# Taxonomic revision of the rock-dwelling door snail genus *Montenegrina* Boettger, 1877 (Mollusca, Gastropoda, Clausiliidae)

**DOI:** 10.3897/zookeys.599.8168

**Published:** 2016-06-16

**Authors:** Zoltán Fehér, Miklós Szekeres

**Affiliations:** 1 3rd Zoological Department, Natural History Museum Vienna, A-1010 Burgring 7, Vienna, Austria; 2 Department of Zoology, Hungarian Natural History Museum, H-1088 Baross u 13, Budapest, Hungary; 3 Institute of Plant Biology, Biological Research Centre of the Hungarian Academy of Sciences, Temesvári krt. 62, H-6726, Szeged, Hungary

**Keywords:** Balkan, Albania, Greece, Montenegro, Macedonia, new taxa, distribution data, types, polytypic species, limestone habitat

## Abstract

The genus *Montenegrina* is revised on the basis of material available at the Hungarian Natural History Museum (Budapest), Naturhistorisches Museum Wien (Vienna), and the Naturmuseum Senckenberg (Frankfurt am Main), as well as newly discovered populations. The following new taxa are described: *Montenegrina haringae* **sp. n.**, *Montenegrina lillae* **sp. n.**, *Montenegrina prokletiana* **sp. n.**, *Montenegrina sturanyana* **sp. n.**, *Montenegrina grammica erosszoltani* **ssp. n.**, *Montenegrina grammica improvisa* **ssp. n.**, *Montenegrina hiltrudae desaretica* **ssp. n.**, *Montenegrina hiltrudae selcensis* **ssp. n.**, *Montenegrina laxa delii* **ssp. n.**, *Montenegrina nana barinai* **ssp. n.**, *Montenegrina prokletiana kovacsorum* **ssp. n.**, *Montenegrina rugilabris golikutensis* **ssp. n.**, *Montenegrina rugilabris gregoi* **ssp. n.**, *Montenegrina skipetarica danyii* **ssp. n.**, *Montenegrina skipetarica gurelurensis* **ssp. n.**, *Montenegrina skipetarica pifkoi* **ssp. n.**, *Montenegrina skipetarica puskasi* **ssp. n.**, *Montenegrina sporadica tropojana* **ssp. n.**, *Montenegrina sturanyana gropana* **ssp. n.**, *Montenegrina sturanyana ostrovicensis* **ssp. n.**, and *Montenegrina tomorosi hunyadii* **ssp. n.** A neotype is designated for *Montenegrina helvola* (Küster, 1860), and *Montenegrina cattaroensis antivaricostata* **nom. n.** was introduced to replace the junior homonym *Clausilia umbilicata costata* Boettger, 1907 (non Pfeiffer, 1928). Of each taxon types or specimens from the type localities are figured, and distribution maps are provided.

## Introduction

*Montenegrina* is an obligate rock-dwelling door snail genus, associated with habitats of limestone outcrops. According to Nordsieck (2007), the genus belongs to the subfamily Alopiinae and, within that, the tribe Montenegrinini, together with *Protoherilla* Wagner, 1921. A family-wide molecular phylogenetic reconstruction confirmed its belonging to the Alopiinae, and found *Alopia* Adams & Adams, 1855 and *Herilla* Adams & Adams, 1855 the closest related genera (Uit de Weerd and Gittenberger 2013).

**Figure 1.**
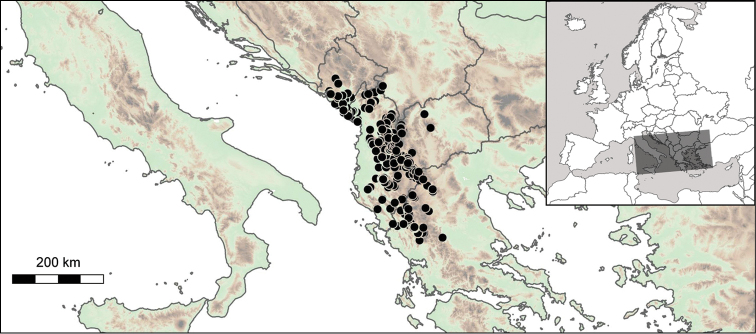
Geographic distribution of *Montenegrina*.

The geographic range of *Montenegrina* includes the coastal regions of Montenegro south of the Bay of Kotor, Albania, western Macedonia, and northwestern Greece (Fig. 1). In geographical terms it extends from the southernmost parts of the Dinaric Mountains to the northern part of the Pindos Mountains, and is separated from the eastern Balkans (Moesia) by the Vardar Basin and the Kosovo Plain. Biogeographically this area can be classified as part of the South Adriatic–Ionian province (Griffiths et al. 2004: 262).

The region is rich in limestone, which occurs in discrete karstic patches that are separated by intervening non-limestone (e.g. Quaternary fluvial deposits, flysch, volcanic and metamorphic) regions. For obligate limestone-dwelling gastropods with limited capacity of active dispersal these karst outcrops function as ‘islands’ that offer home to isolated populations (Gittenberger 2007). Nevertheless, in some rare events, local forms can be subject to jump-dispersal, allowing colonization at remote locations (Uit de Weerd et al. 2005). Allopatric distribution, in combination with the restricted dispersal, provides favourable conditions for non-adaptive speciation (e.g. via founder effect, genetic drift), and can give rise to radiation into multiple, morphologically diverse and geographically separated forms (Gittenberger 1991).

Unambiguous application of species and subspecies concepts under these circumstances can be quite challenging. In Balkan species of the rock-dwelling alopiinid door snails related morphotype groups often show higher divergence between than within their isolated populations and, when groups of populations show morphological diversity, their features tend to follow discernible geographical partitioning. In the taxonomical practice such species have been regarded polytypic, in the sense of Mayr’s subspecies concept (Mallet 2007). In *Montenegrina* the number of traditionally recognized subspecies is high, representing about three-quarters of the described taxa in the genus (Nordsieck 2007). Occasionally the unambiguous delimitation of species can also be problematic. As different morphs only rarely co-occur or meet in a contact zone, reproductive and competitive relationships under natural conditions cannot be ascertained. Consequently, in some cases it can be difficult to judge objectively which forms can or cannot be regarded conspecific (Gittenberger 2007).

During the 19th century the range of *Montenegrina*, except for the Montenegrin coast, belonged to the reclusive Ottoman Empire and, as a result, remained mostly inaccessible to zoological exploration. Therefore, the first taxa of the genus became known from the Adriatic coast of Montenegro, part of which then belonged to the Austrian Empire. The description of the first species, *Clausilia cattaroensis* Rossmässler, 1835, was followed by those of *Clausilia subcristata* Pfeiffer, 1848, Clausilia cattaroensis var. gracilior Küster, 1850, *Clausilia helvola* Küster, 1860, *Clausilia laxa* Küster, 1861 (the latter two likely from marine flotsam, see: Nordsieck 1972), *Clausilia umbilicata* Boettger, 1879, and Clausilia (Herilla) klecaki Westerlund, 1881. Exceptions were *Clausilia janinensis* Mousson, 1859 and *Clausilia rugilabris* Mousson, 1859 from the Turkish-occupied region, which Mousson (1859) described from material collected by the Schaefli Expedition to the Ioannina region.

After the turn of the century, Rudolf Sturany and Otto Wohlberedt began exploring the Ottoman-ruled northern Albanian area (Sturany 1905, Wohlberedt 1907). During the 1st World War, wide-scoped Austrian, Hungarian and German expeditions were organized to the northern territories of present-day Albania, eastern Montenegro, Kosovo and western Macedonia, which were then occupied by the armies of the Central Powers (Doflein 1921, Kümmerle 1926, Teleki and Csiki 1922–1940). The molluscs collected on these expeditions were studied by Wagner (1919), Soós (1924) and Hesse (1928). There were only few follow-up malacological studies (e.g. those of Anton Fuchs) between the two World Wars. Molluscs were typically collected as by-products by experts focusing on other groups (e.g. Bischoff, Dabović, Weigner and Winkler). Since the 1960s and 1970s the exploration of Greek and former Yugoslavian areas became intense due to the contributions of Wolfgang Fauer, Hartmut Nordsieck, László Pintér, Péter Subai and, somewhat later, Ivailo Dedov, Peter Reischütz, and Helmut Sattmann. By contrast, Albania was inaccessible for naturalists until the last decade of the 20th century, when finally a substantial set of malacological data from Albania could be published (Dhora and Welter-Schultes 1996, Welter-Schultes 1996).

As Albania and the neighbouring regions still remained the least explored parts of Europe, it became one of the focus areas in the research of the Hungarian Natural History Museum (HNHM). From the late 1990s the HNHM organized nearly a hundred zoological and botanical field expeditions to this area (Barina et al. 2014, Erőss and Fehér 2009, Fehér et al. 2004, Murányi 2007, Murányi et al. 2011), which sampled at thousands of localities that have never been surveyed by naturalists before. These field trips resulted in significant accessions of the Mollusc Collection, providing several undescribed taxa, as well as new distribution records for little-known species of *Montenegrina*.

This in itself could have justified the need for a taxonomic revision of this genus. But there are also other reasons why *Montenegrina* became the focus of our interest. The size of its range and the number of known populations deemed large enough, but still accessible to almost comprehensive sampling. Furthermore, its patchy distribution makes this genus an attractive system for molecular phylogenetic, phylogeographic and evolutionary studies that can help better understanding the general mechanisms of speciation and spatial distribution of rock-dwelling gastropods. Such studies, however, require a thorough, morphology-based revision of the taxa.

## Methods

In the text common geographic names and terms are given according to their current English usage. However, in the sections listing the available material these names and their spelling often follow those of the literature records or original labels. These, as well as historic or obsolete names, are given between quotation marks. To allow unambiguous identification of locality names geographical objects are often defined by locally used terms. These are as follows:

brana dam (Macedonian)


chuka summit (Macedonian)


çuka summit (Albanian)


farangi canyon (Greek)


gora mountain (Serbian)


izvor spring (Serbian)


lumi river (Albanian)


maja summit (Albanian)


mali mountain (Albanian)


pečina cave (Serbian)


përroi stream, creek (Albanian)


planina mountain (Serbian)


potok stream (Serbian)


qafa mountain pass (Albanian)


shpella cave (Albanian)


shkëmb cliff (Albanian)


ura bridge (Albanian)


In some of the countries different names are used for transboundary objects (e.g. Crni Drin/Drin i Zi/Black Drin, Vjosë/Aoós, Skadar/Shkodër/Skutari, Nemerçkë/Dousko, etc.). In such cases these are mentioned in the form used in the country where the actual site belongs.

The studied samples are in the following collections:

HNC
Haus der Natur, Cismar 


HNHM
Hungarian Natural History Museum, Budapest 


MIZPAS
Museum and Institute of Zoology, Polish. Academy of Sciences, Warsaw 


MMM
 Munkácsy Mihály Museum, Békéscsaba 


NHMUK
Natural History Museum, London



NHMW
Naturhistorishes Museum, Vienna 


NHMW-E
 Edlauer collection in NHMW



NHMW-K
 Klemm collection in NHMW



NMBE
Natural History Museum Bern 


RMNH
Naturalis Biodiversity Center, Leiden 


SMF
 Naturmuseum Senckenberg, Frankfurt am Main 


SMNS
 Stuttgart State Museum of Natural History, Stuttgart 


SMNS-N
 Nordsieck collection in SMNS



ZMH
Zoological Museum, Hamburg 


ZSM
Bavarian State Collection of Zoology, München 


DED
 private collection of Ivailo Dedov, Sofia 


ER
 private collection of Zoltán Erőss, Budapest 


GR
 private collection of Jozef Grego, Banská Bystrica 


HU
 private collection of András Hunyadi, Budapest 


SZ
 private collection of Miklós Szekeres, Szeged 


Private collections are referred to only when they include type material of the newly described taxa. Numbers of the type specimens are given separately for the dry and alcohol-preserved specimens. Among the latter ‘a’ and ‘aj’ denote adult and recognizable juvenile individuals, respectively.

The names of the frequently mentioned collectors are abbreviated as follows: AH – András Hunyadi, AR – Alexander Reischütz, AS – Anna Szigethy, CN – Csaba Németh, DA – Dorottya Angyal, DM – Dávid Murányi, DP – Dániel Pifkó, EH – Elisabeth Haring, EM – Edvárd Mizsei, GP – Gellért Puskás, HS – Helmut Sattmann, ID – Ivailo Dedov, JG – Jozef Grego, JK – Jenő Kontschán, KJ – Katharina Jaksch, KK – Kornél Kovács, LD – László Dányi, LP – László Pintér, LT – Lilla Tamás, MD – Michael Duda, NR – Nicole Reischütz, PJ – Péter Juhász, PR – Peter L. Reischütz, PS – Péter Subai, TD – Tamás Deli, TK – Tibor Kovács, TH – Tamás Huszár, TN – Tamás Németh, ZB – Zoltán Barina, ZE – Zoltán Erőss, ZF – Zoltán Fehér, ZU – Zsolt Ujvári.

Georeferenced distribution records used in this paper are deposited at the Global Biodiversity Information Facility (GBIF), http://ipt.pensoft.net/resource?r=montenegrina

**Figure 2.**
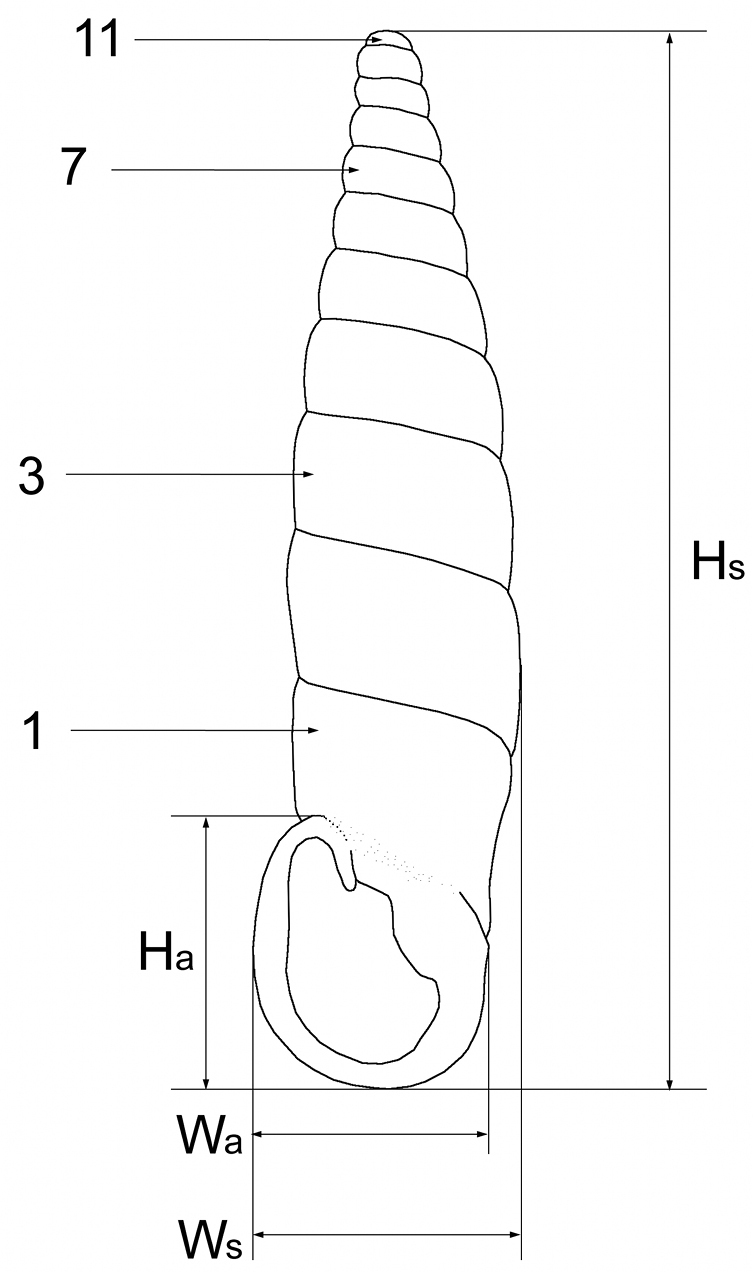
Shell dimensions and whorls counts. H_a_: apertute height, H_s_: shell height, W_a_: aperture width, W_s_: shell width.

Dimensions and whorl numbers are given as shown in Fig. 2. For most of the taxa size data are taken from the literature, preferentially from the original descriptions (unless indicated otherwise). For the new taxa the measurements of the holotypes and 10–12 randomly selected paratypes are given. These were taken from front view images by ImageJ software (Schneider et al. 2012) using landmarks placed on the pictures. The dimensions of the shells are given by size ranges, as well as means and standard deviation values, whereas those of the apertures only by size ranges. In the diagnoses shell height is given simply as large (>20 mm), medium (15–20 mm), small (10–15 mm), or very small (<10 mm).

**Figure 3.**
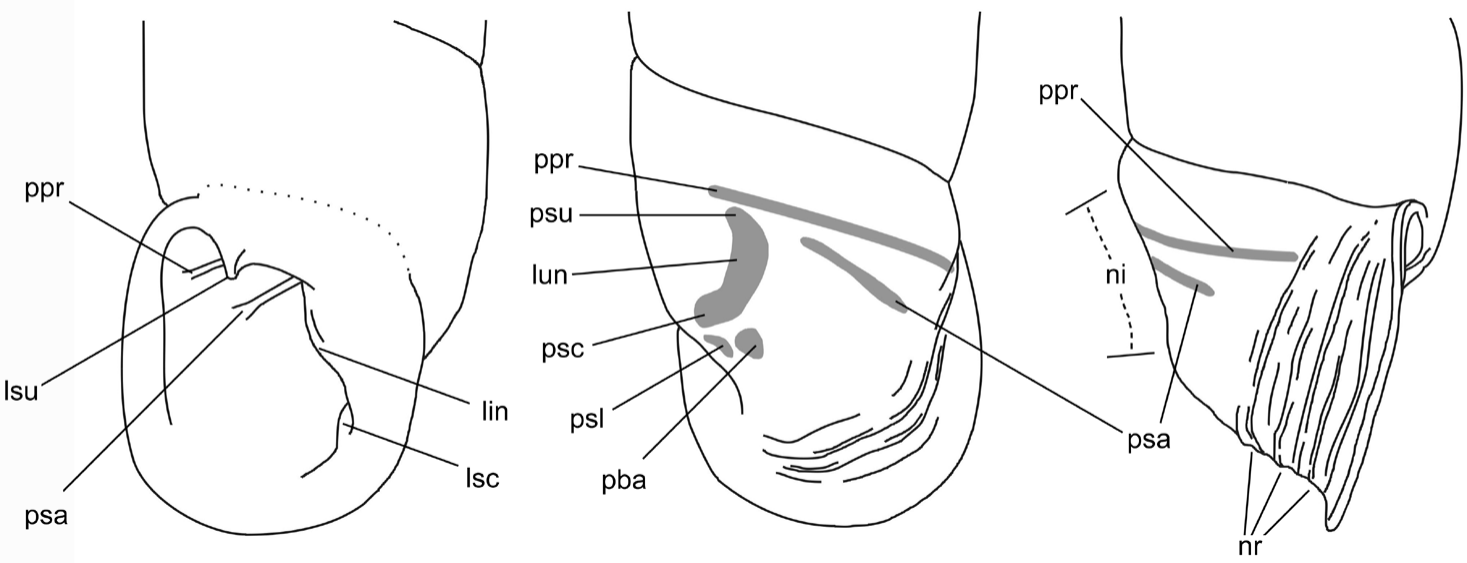
Shell structures of the last whorl. lin: lamella inferior, lsc: lamella subcolumellaris, lsu: lamella superior, lun: lunella, ni: neck inflection, nr: neck ribs, pba: plica basalis, ppr: plica principalis, psa: anterior plica superior, psc: plica subclaustralis, psl: plica sulcalis, psu: plica superior.

**Figure 4.**
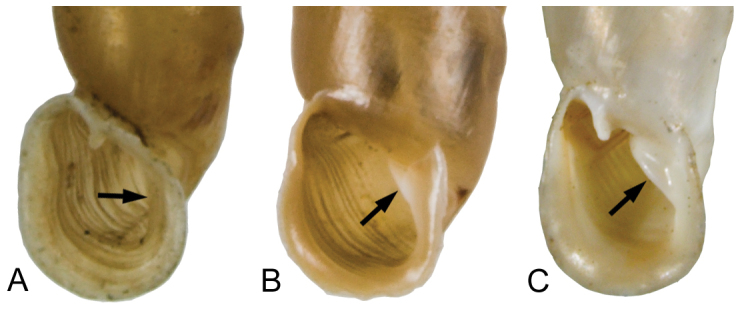
Different exposures of the lamella inferior. **A** hidden (*Montenegrina helvola*) **B** weakly emerged (*Montenegrina okolensis*) **C** well emerged (*Montenegrina subcristata*).

**Figure 5.**
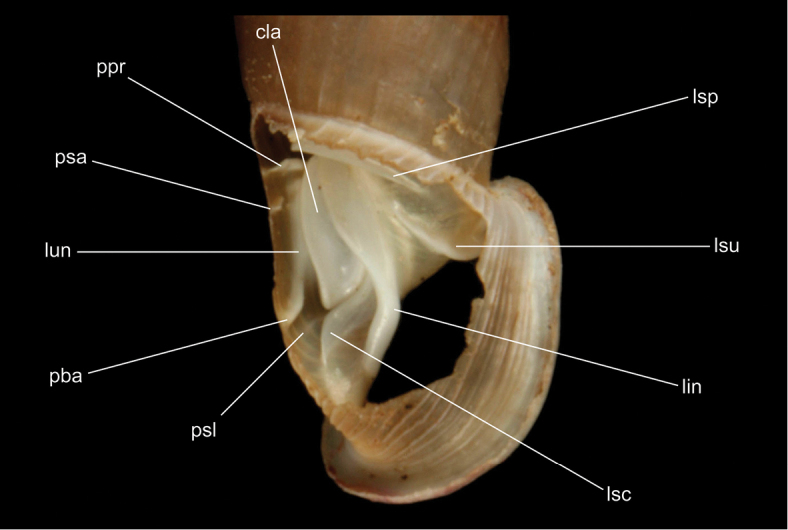
Components of the clausiliar apparatus. cla: clausilium plate, lin: lamella inferior, lsc: lamella subcolumellaris, lsp: lamella spiralis, lsu: lamella superior, lun: lunella, pba: plica basalis, ppr: plica principalis, psa: anterior plica superior, psl: plica sulcalis.

**Figure 6.**
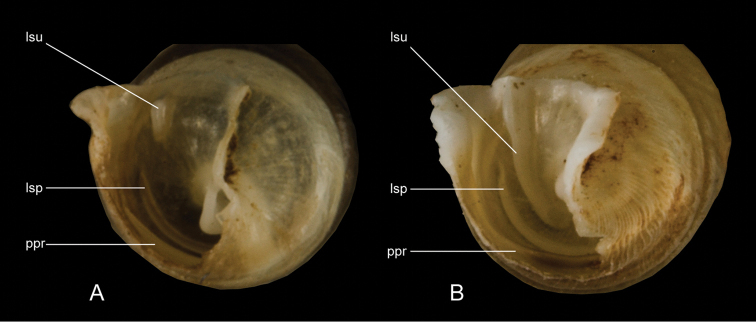
Relation betwen the lamellae superior and spiralis. **A** non-overlapping (*Montenegrina dofleini fagorum*) **B** overlapping (*Montenegrina perstriata callistoma*). lsp: lamella spiralis, lsu: lamella superior, ppr: plica principalis.

**Figure 7.**
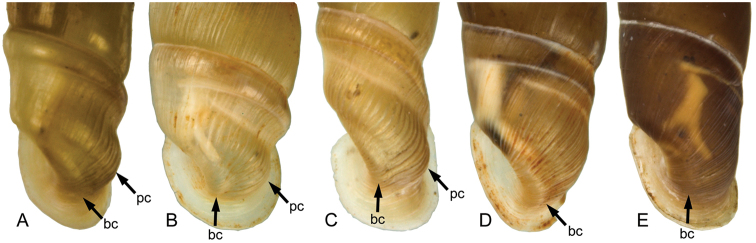
Variable strengths of the basal (bc) and peripheral crests (pc). **A** *Montenegrina prokletiana prokletiana* ssp. n. **B** *Montenegrina drimmeri* **C** *Montenegrina helvola helvola* **D** *Montenegrina perstriata ochridensis* **E** *Montenegrina rugilabris irmengardis*.

**Figure 8.**
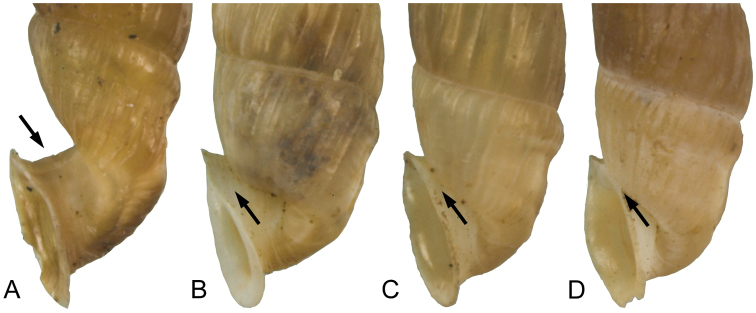
Positions of the peristome. **A** strongly projected (*Montenegrina fuchsi fuchsi*) **B** projected (*Montenegrina fuchsi pallida*) **C** detached (*Montenegrina fuchsi klemmi*) **D** attached (*Montenegrina fuchsi klemmi*).

**Figure 9.**
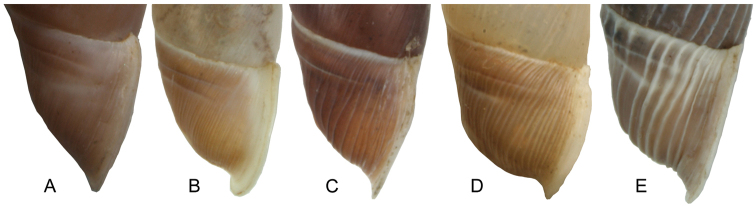
Different strengths of the neck sculpture. **A** finely striate (*Montenegrina subcristata*) **B** striate (*Montenegrina hiltrudae sattmanni*) **C** wrinkled-costate (*Montenegrina skipetarica remota*) **D** costate (*Montenegrina hiltrudae costulata*) **E** strongly costate (*Montenegrina skipetarica thysi*).

The terminology of distinctive shell structures is used according to the detailed descriptions provided by Likharev (1962) and Nordsieck (1982), and illustrated in Figs 3–9. The term lunella complex designates the palatal adduct formed by the fusion of the lunella with the plicae superior and subclaustralis (Fig. 3).

## Systematic part

### Family Clausiliidae Gray, 1855 Subfamily Alopiinae Wagner, 1913

#### Genus Montenegrina

Taxon classificationAnimaliaStylommatophoraClausiliidae

Boettger, 1877

Montenegrina [Clausilia, Sect. X. Delima, gruppe der cattaroensis] Boettger 1877a: 37.
Clausilia (Heteroptycha) Westerlund, 1884: 5.
Montenegrina [Clausilia (Delima) gruppe A.] – Westerlund 1884: 50.
Montenegrina [Delima, group i.] – Kennard and Woodward 1923: 307.
Heteroptychia (sic!) – Kennard and Woodward 1923: 308.
Delima (Albanodelima) Wagner, 1924: 118. – Wagner 1925: 59.
Montenegrina [Alopia (Delima) a.] – Lindholm 1924: 57.
Heteroptycha [Alopia (Delima) b.] – Lindholm 1924: 57.
Delima (Montenegrina) – Zilch in Wenz 1960: 429–430.
Delima (Heteroptycha) – Zilch in Wenz 1960: 429–430.
Montenegrina (Heteroptycha) – Brandt 1961: 1.
Montenegrina (Montenegrina) s. str. – Brandt 1961: 3.
Montenegrina (Beieriella) Klemm, 1962: 242.
Montenegrina – Brandt 1962: 141. – Nordsieck 1969: 259. – Nordsieck 1972: 26. – Nordsieck 1974: 149. – Gittenberger 2002: 131. – Welter-Schultes 2012: 326.

##### Type species.

[*Montenegrina*]: *cattaroensis*—to our best knowledge, Kennard and Woodward (1923) were the first mentioning this as type species, but without pointing out subsequent designation; [*Heteroptycha*]: *helvola*—by monotypy, see Lindholm (1924); [*Albanodelima*]: *umbilicata*—subsequent designation by Nordsieck (2001: 20); [*Beieriella*]: *irmengardis*—by original designation.

##### Distribution.

Coastal regions of Montenegro south of the Bay of Kotor, Albania, western Macedonia, and northwestern Greece (Fig. 1.).

##### Ecology.

**Figure 10.**
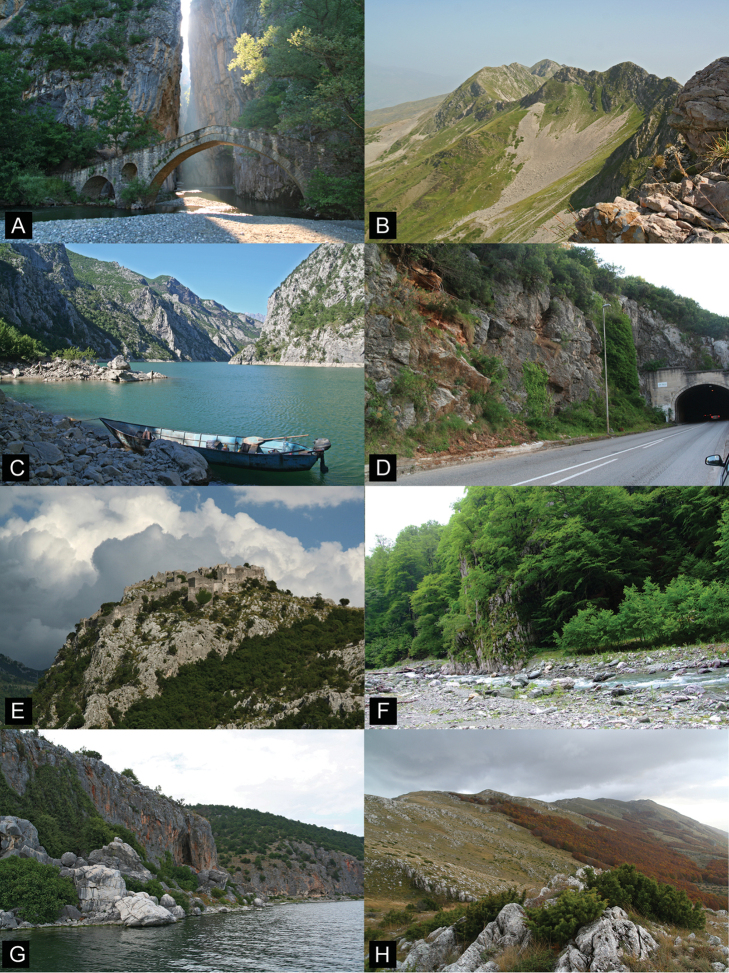
Typical rocky habitats preferred by *Montenegrina* species. **A** Portitsa Gorge of the Venetikos River (type locality of *Montenegrina hiltrudae maasseni*) **B** rocky ridge of the Ostrovica Mts north of the Faqekuq Summit (type locality of *Montenegrina sturanyana ostrovicensis* ssp. n.) **C** valley of the Drin upstream of the Koman Dam (near the type locality of *Montenegrina prokletiana kovacsorum* ssp. n.) **D** roadside cliff near Zaljevo (*Montenegrina subcristata*) **E** ruins of the medieval fortress at Haj-Nehaj (*Montenegrina cattaroensis umbilicata*) **F** rocky forest in the valley of the Tropojë Stream (type locality of *Montenegrina sporadica tropojana* ssp. n.) **G** cliffs along the shore of Lake Prespa near the Sv. Marena church (*Montenegrina dofleini prespaensis*) **H** rocky grassland in the alpine region of the Galičica Mts south of the Bugarska Summit (*Montenegrina dofleini dofleini*).

*Montenegrina* species inhabit rocky habitats, where they feed on the microflora and find hiding place in crevices or among and under boulders. All known populations are associated with limestone environments. The formation and shape of the habitable outcrops can vary considerably even within a species. Suitable habitats include small to large cliffs, gorges, rocky forests, rocky alpine grasslands, or even artificial stone walls along roads (Fig. 10).

##### Historical background.

The first few described taxa of *Montenegrina* were initially classified within the genus *Clausilia* Draparnaud, 1805 and, later on, in the subgenus Clausilia (Delima) Hartmann, 1842. In his *Clausilia* monograph Schmidt (1868) already distinguished *Clausilia cattaroensis*, *Clausilia helvola*, *Clausilia laxa*, and *Clausilia subcristata* as the group of *Clausilia cattaroensis*. The name *Montenegrina* was first used by Boettger (1877a) to designate the *cattaroensis* group within the subgenus Clausilia (Delima). A formal description of *Montenegrina* was provided by Westerlund (1884), who included here all aforementioned taxa except *Clausilia helvola*. For this species, based on its special aperture structure, he established the subgenus Clausilia (Heteroptycha), which was then elevated to genus rank by Kennard and Woodward (1923). Later Zilch (1960) considered *Montenegrina* and *Heteroptycha* as subgenera of *Delima*, whereas *Albanodelima* Wagner, 1924 as a synonym of *Heteroptycha*.

Based on genital morphology, Wagner (1924) was the first to unite all *Montenegrina* species known at that time within one subgenus, Delima (Albanodelima) Wagner, 1924. A description of this taxon, originally outlined only by a list of the involved species, was given in a subsequent publication of Wagner (1925). In this he defined three species groups within Delima (Albanodelima). The *umbilicata* group included Delima (Albanodelima) umbilicata, Delima (Albanodelima) cattaroensis, Delima (Albanodelima) interior (Boettger, 1907), Delima (Albanodelima) kleciaki, Delima (Albanodelima) skipetarica (Soós, 1924), Delima (Albanodelima) subcristata, Delima (Albanodelima) weigneri Poliński, 1924, and Delima (Albanodelima) wohlberedti (Möllendorff, 1899). Wagner pointed out that he regarded part of *Montenegrina* (as used by Kennard and Woodward, 1923) synonymous with this species group. The *attemsi* group comprised Delima (Albanodelima) attemsi Wagner, 1914, Delima (Albanodelima) janinensis, Delima (Albanodelima) ochridensis Wagner, 1925, Delima (Albanodelima) perstriata Wagner, 1919, and Delima (Albanodelima) rugilabris. Finally, the group of *helvola* included Delima (Alpidelima) helvola and Delima (Albanodelima) apfelbecki (Sturany, 1907), with a note that *Heteroptycha* was considered a synonym of this group. In contrast to this concept, Zilch (1960) referred to *Albanodelima* as if it had been used by Wagner only for the *helvola* group, and thus would have been a synonym of *Heteroptycha*.

*Montenegrina* was first regarded a distinct genus by Brandt (1961). In the following year Klemm (1962) erected the subgenus Montenegrina (Beieriella), which was later rejected by Nordsieck (1972), claiming that it had been based merely on distinctive characters of its type species, Montenegrina (Beieriella) irmengardis Klemm, 1962. In the same article Nordsieck also dismissed *Heteroptycha* as subgenus. Subsequent publications also do not use subgeneric division of *Montenegrina* (Nordsieck 1969, 1972, 1974, 2009; Erőss et al. 1999, 2006; Welter-Schultes 2012), only species groups are distinguished in Nordsieck’s works.

Assessments of the genital morphology of *Montenegrina* were provided by Nordsieck (1969, 1972, 2009), based on examinations of *Montenegrina cattaroensis*, *Montenegrina janinensis*, *Montenegrina rugilabris*, *Montenegrina irmengardis*, *Montenegrina stankovici* (Urbański 1960), *Montenegrina subcristata* and *Montenegrina umbilicata* samples (Nordsieck, 2009). Genitalia data were also given on Delima (Albanodelima) apfelbecki, Delima (Albanodelima) kleciaki and Delima (Albanodelima) subcristata by Wagner (1925), Clausilia (Delima) skipetarica by Soós (1924), Montenegrina (Beieriella) irmengardis by Klemm (1962), Delima (Montenegrina) perstriata ochridensis by Loosjes (1966), *Montenegrina janinensis maasseni* Gittenberger, 2002 by Uit de Weerd and Gittenberger (2004) and *Montenegrina dofleini sinosi* Páll-Gergely, 2010 in its description (Páll-Gergely 2010). Data on the microarmature of the clausilium plate were published by Gittenberger (2002).

The most recent revision of the genus was provided by Nordsieck (2009). In this he distinguished eight species groups, namely those of *Montenegrina cattaroensis*, *Montenegrina drimmeri* Fehér & Szekeres, 2006, *Montenegrina fuchsi* Brandt, 1961, *Montenegrina janinensis*, *Montenegrina laxa*, *Montenegrina perstriata*, *Montenegrina rugilabris*, and *Montenegrina skipetarica*. By and large this concept was adopted in the Fauna Europaea checklist (Bank 2013), which mentions 22 species of *Montenegrina* with 87 subspecies. In the present paper a revised system of *Montenegrina* is provided on the basis of the material available at the HNHM, NHMW and SMF collections, as well as that of newly discovered populations.

#### Montenegrina apfelbecki

Taxon classificationAnimaliaStylommatophoraClausiliidae

(Sturany, 1907)

Fig. 11A

Clausilia apfelbecki Sturany, 1907: 233. – Wohlberedt 1909: 101.
Delima (Delima) apfelbecki – Sturany and Wagner 1915: 74, plate 16, fig. 91.
Delima (Albanodelima) apfelbecki – Wagner 1924: 120. – Wagner 1925: 67, plate 1, fig. 12. (genital anatomy).
Montenegrina apfelbecki – Nordsieck 1972: 28. – Zilch 1981: 126, plate 14, fig. 30. – Nordsieck 2009: 75.

##### Diagnosis.

Shell small, light corneous. All whorls smooth, glossy. Neck inflexed, densely striate. Basal and peripheral crests well visible. Peristome detached, ovoid, with simple margin. Lamellae superior and spiralis do not overlap. In front view lamella inferior moderately emerged, weakly-bent subcolumellaris not visible. Lunella dorsal-dorsolateral. Basalis mostly absent, rarely residual and separate from the lunella. Subclaustralis very short, sulcalis well developed. Anterior plica superior absent. Clausilium plate almost entirely visible through the aperture.

##### Dimensions

(in mm). H_s_: 12.6–16.0, W_s_: 3.4–4.2 (syntypes NHMW 41166).

**Figure 11.**
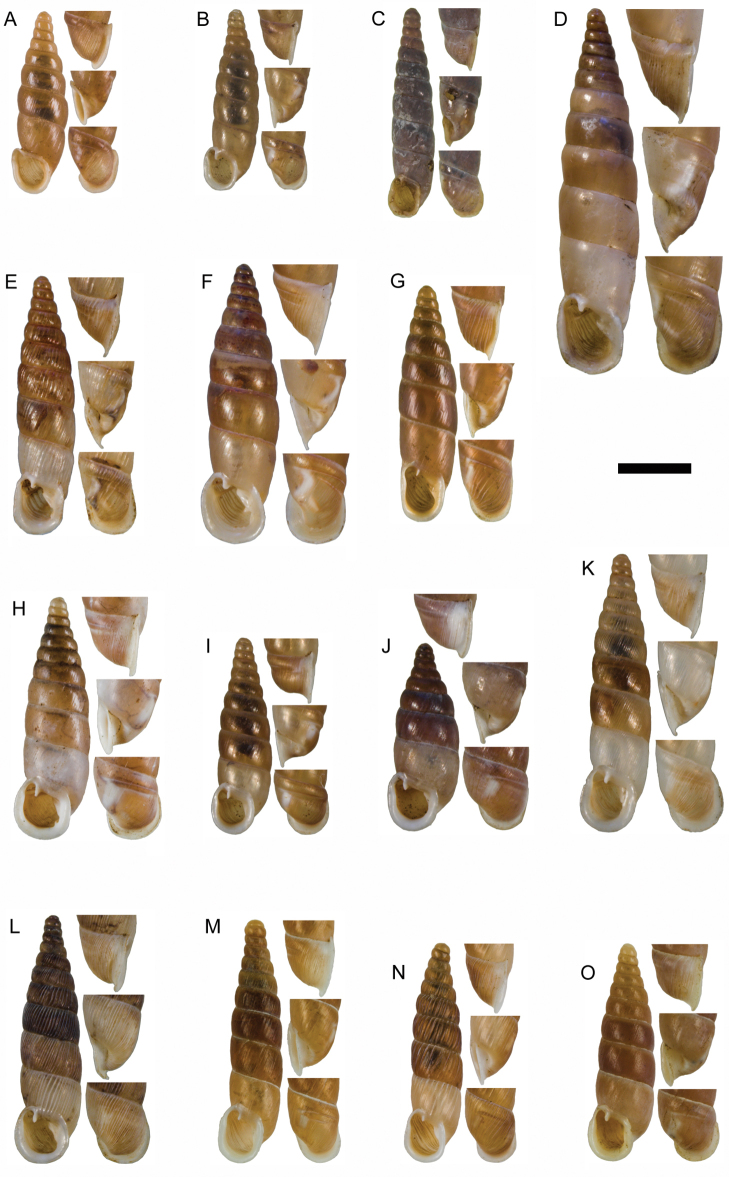
**A** *Montenegrina apfelbecki* (Sturany, 1907), syntype, NHMW 41166 **B** *Montenegrina attemsi attemsi* (Wagner, 1914), syntype, SMF 167038 **C** *Montenegrina attemsi jakupicensis* Fauer, 1993, holotype, SMF 309239 **D** *Montenegrina cattaroensis cattaroensis* (Rossmässler, 1835), lectotype, SMF 156967 **E** *Montenegrina cattaroensis antivaricostata* nom. n., lectotype of *costata* (Boettger, 1907), SMF 176320 **F** *Montenegrina cattaroensis umbilicata* (Boettger, 1879), lectotype, SMF 176318 **G** *Montenegrina chiasma* Nordsieck, 1972, paratype, NHMW 75000/-E 32343 **H** *Montenegrina dofleini dofleini* (Wagner, 1928), Tomoros, original series, ZSM 20150453 **I** *Montenegrina dofleini fagorum* Nordsieck, 1974, holotype, SMF 227680 **J** *Montenegrina dofleini dofleini* (Wagner, 1928), paratype of *kaiseri*, SMF 201563 **K** *Montenegrina dofleini kastoriae* Nordsieck, 1972, holotype, 221042 **L** *Montenegrina dofleini pinteri* Nordsieck, 1974, holotype, SMF 227684 **M** *Montenegrina dofleini prespaenis* Nordsieck, 1988, NHMW 84019 **N** *Montenegrina dofleini sinosi* Páll-Gergely, 2010, holotype, HNHM 97145 **O** *Montenegrina dofleini wagneri* Szekeres, 2006, holotype, NHMW 103375. Scale bar: 5 mm.

##### Type locality.

“Mal i Shêit bei Oroshi, Merdita, in einer Höhe von zirka 1500 m” = Albania, Mt. Shënt, ca. 1500 m.

##### Type material.

Type locality, leg. Buljubašić, 1905, syntypes (NHMW 41166/pl., NHMW-E 38290/2, NHMW-K 9368/3, SMF 32771/2, SMF 201619); same data, ex Oberwimmer, syntypes (NHMW 65490/2), same data, ex Käufel, syntypes (NHMW 70409/3).

##### Distribution.

**Figure 12.**
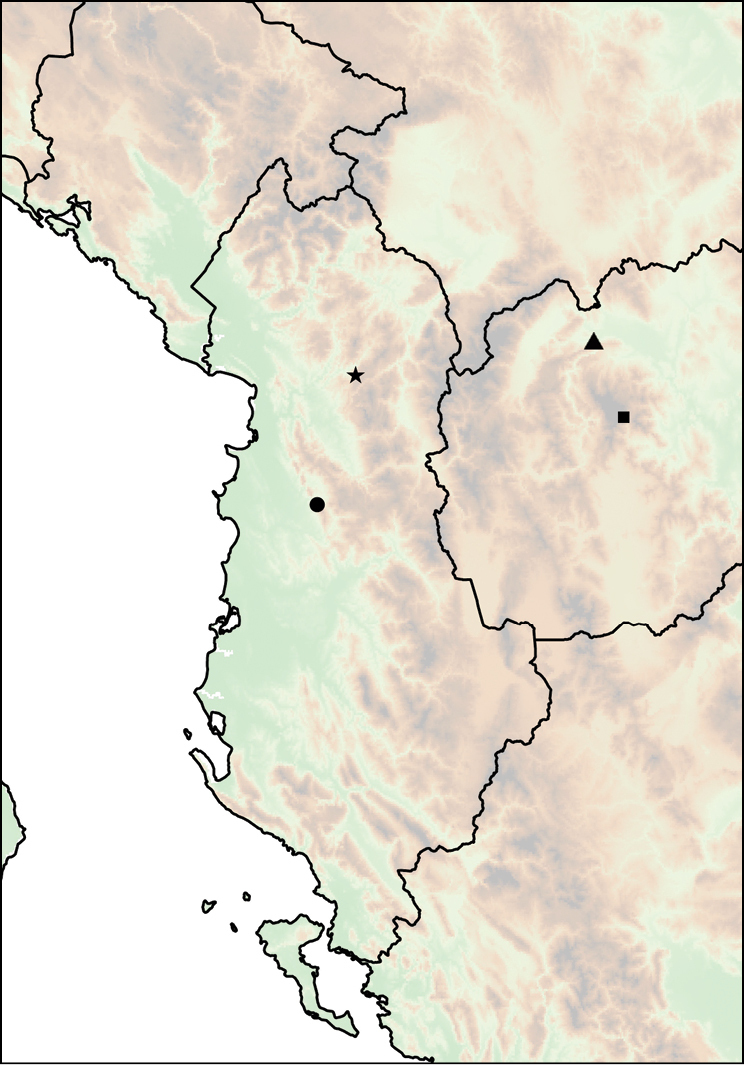
Distribution of *Montenegrina apfelbecki*. *Montenegrina attemsi* and *Montenegrina chiasma*. *Montenegrina apfelbecki* (star); *Montenegrina chiasma* (circle); *Montenegrina attemsi attemsi* (triangle); *Montenegrina attemsi jakupicensis* (square).

Mount Shënt (Mali i Shëntit) in Northern Albania (Fig. 12).

##### Remarks.

Of this taxon only the type series are known. The material was collected during the 1905 expedition organized by Rudolf Sturany, then curator of the Mollusc Collection at NHMW. Sturany was accompanied by Viktor Apfelbeck, curator of the entomological collection at the National Museum of Sarajevo, and Latif Buljubašić, Apfelbeck’s assistant. After a few days of joint work in the Oroshi area Buljubašić was left alone for two months to explore the surrounding Shënt, Munellë and Koritnik mountains (Sturany 1905). During that period Buljubašić acquired a large and diverse invertebrate material, but *Montenegrina apfelbecki* was found only at one site. This suggests that the species was highly local, although the discovered population could have been quite large. In 2014 a thorough search by the first author over the plateau of the Shënt Mountain, along its western slope and northern side toward the Mount Zebë, failed to re-discover the type locality of this species (Angyal et al. 2015). Apparently it is not frequent in the Shënt Mountain, and may only have a very narrow range.

#### Montenegrina attemsi

Taxon classificationAnimaliaStylommatophoraClausiliidae

(Wagner, 1914)

##### Diagnosis.

Shell small, slender, light yellowish-corneous. All whorls smooth. Peristome attached, ovoid to angular, with somewhat swollen margin. Lamellae superior and spiralis overlap. In front view lamella inferior hidden or weakly emerged, subcolumellaris broadly-bent. Lunella dorsolateral to lateral, diffuse, not connected to the basalis. Subclaustralis absent or residual, sulcalis present. Anterior plica superior not connected to the lunella complex. Clausilium plate only barely visible through the aperture. Differs from similar-looking *Montenegrina janinensis* by the smaller, more elongate, entirely smooth shell, as well as the less emerged lamella inferior.

#### Montenegrina attemsi attemsi

Taxon classificationAnimaliaStylommatophoraClausiliidae

(Wagner, 1914)

Fig. 11B

Delima (Delima) attemsi Wagner, 1914 in Sturany and Wagner 1915: 73–74, plate 16, fig. 92.
Delima (Albanodelima) attemsi – Wagner 1924: 120.
Montenegrina janinensis attemsi – Nordsieck 1974: 151, plate 5, fig. 28. – Zilch 1981: 129. – Nordsieck 2009: 75.

##### Diagnosis.

Neck striate to striate-costate. Basal crest strong, peripheral crest weak. In front view lamella inferior weakly emerged, broadly-bent subcolumellaris mostly visible. Basalis short, subclaustralis weak. Anterior plica superior mostly present.

##### Dimensions

(in mm). H_s_: 12.0–14.8, W_s_: 3.0–3.5 (Nordsieck 1974).

##### Type locality.

“Treska bei Üsküb” = Macedonia, Matka Gorge near Skopje.

##### Type material.

Type locality, leg. Apfelbeck, ex Oberwimmer [10525], syntypes (NHMW 65491/3); same locality, ex Wagner, ex Käufel, syntypes (NHMW-E 9407/3); same locality, ex Käufel, syntypes (NHMW-K 9366/4); same locality, ex Wagner, ex Fuchs, ex Rušnov, ? syntypes (NHMW 70410/3); same locality, leg. Apfelbeck, ex Wagner, ex Brandt, syntype (SMF 167038/1); same locality, ex Käufel, ex Jaeckel, syntype (SMF 201603).

##### Other material.

Macedonia, gorge of the Treska near Skopje, leg. Penther, 11.v.1918 (NHMW 52313); Skopje District, Matka Gorge near the Sv. Andreja Church, 300 m, 41.9610°N, 21.2943°E, leg. ZE, ZF, AH, 10.iv.2004. (HNHM 94429); same locality, leg, TD, 2008. (HNHM 99519).

##### Distribution.

The subspecies is known only from the valley of the Treska River (Matka Gorge) in northwestern Macedonia (Fig. 12).

##### Remarks.

Although the description was formally published in Sturany and Wagner (1915), a reprint of the paper had already been printed and circulated in 1914, and the authorship of the taxon has been unequivocally attributed to Wagner.

#### Montenegrina attemsi jakupicensis

Taxon classificationAnimaliaStylommatophoraClausiliidae

Fauer, 1993

Fig. 11C

Montenegrina janinensis jakupicensis Fauer, 1993: 56–57, plate 1, fig. 8. – Nordsieck 2009: 75.

##### Diagnosis.

Neck smooth, basal and peripheral crests weak. In front view lamella inferior mostly hidden, broadly-bent subcolumellaris also not visible. Basalis mostly absent, subclaustralis absent or residual. Anterior plica superior mostly absent.

##### Dimensions

(in mm). H_s_: 12.4–16.9 (holotype 14.2), W_s_: 2.7–3.4 (holotype 2.9).

##### Type locality.

Macedonia, Jakupica Mts, Nežilovo, 6.8 km to Dom Čeplez, ca. 1170 m.

##### Type material.

Type locality, leg. PS, 12.vii.1990, holotype (SMF 309239), paratypes (NMBE 535051/6, ZMH 91629/3); Nežilovo, 6.4 km towards Dom Čeplez, ca. 1120 m, leg. PS, 12.vii.1990, paratypes (SMF 309240/1, NMBE 535050/4, ZMH 91628/1, ZHM 91630/1).

##### Other material.

Macedonia, Nežilovo, quarry ca. 10 km on the road to Dom Čeplez, 1230 m, 41.6810°N, 21.4303°E, leg. ZE, ZF, AH, 4.iv.2004. (HNHM 95358); Babuna Spring NW of Nežilovo, 1280 m, 41.6903°N, 21.4162°E, leg. DM, TK, 3.x.2013 (HNHM 99520).

##### Distribution.

Southern part of the Jakupica Mts in Macedonia. Known only from a few sites, within a narrow range northwest of Nežilovo village (Fig. 12).

#### Montenegrina cattaroensis

Taxon classificationAnimaliaStylommatophoraClausiliidae

(Rossmässler, 1835)

##### Diagnosis.

Shell medium to large, light corneous. Whorls smooth or indistinctly costate. Neck weakly inflexed, basal and peripheral crests weak to well recognizable. Peristome attached, ovoid to angular, with somewhat swollen margin. Lamellae superior and spiralis mostly overlap. In front view lamella inferior well emerged, medium-bent subcolumellaris mostly visible. Lunella dorsal to dorsolateral, separate from the strong basalis. Subclaustralis well developed, sulcalis present. Anterior plica superior long, separate from the lunella complex. Clausilium plate partly visible through the aperture.

#### Montenegrina cattaroensis cattaroensis

Taxon classificationAnimaliaStylommatophoraClausiliidae

(Rossmässler, 1835)

Fig. 11D

Clausilia cattaroensis Rossmässler, 1835: Heft 2, pp. 8–9, plate 7, fig. 100. – Küster 1844–1862: 40–41, plate 4, figs 14–17. – Pfeiffer 1848: 437–438. – Schmidt 1868: 68.
Clausilia cattaroensis var. gracilior Küster, 1850 in Küster 1844–1862: 40.
Clausilia cattaroënsis (partim) – Walderdorff 1864: 509.
Clausilia (Delima) cattaroensis – Westerlund 1884: 53–54. – Wohlberedt 1907: 551. – Wohlberedt 1909: 673–674, plate 14, figs 148–151.
Clausilia (Delima) cattaroensis f. parvula Westerlund, 1884: 54.
Clausilia (Delima) catarvensis (sic!) – Westerlund 1884: 54.
Clausilia (Delima) lævigata Mhlf. – Westerlund 1884: 54.
Clausilia (Delima) lesinacensis Parr. – Westerlund 1884: 54.
Clausilia (Delima) cattaroensis var. gracilior – Westerlund 1884: 54.
Delima (Delima) cattaroensis – Sturany and Wagner 1915: 73–74, plate 16, fig. 92.
Delima (Albanodelima) cattaroensis – Wagner 1924: 118.
Delima (Montenegrina) cattaroensis – Zilch in Wenz 1960: 429–430, fig. 1526.
Montenegrina cattaroensis – Nordsieck 1969: 259. (genital anatomy) – Zilch 1981: 127, plate 12, fig. 16. – Nordsieck 2009: 73.

##### Diagnosis.

Shell mostly large, elongate. All whorls smooth. Neck finely costate. Basal and peripheral crests well recognizable. Lamellae superior and spiralis overlap. Lunella dorsolateral. Plica principalis often connected to the superior. Sulcalis well developed.

##### Dimensions

(in mm). H_s_: 16.8–26.5 (lectotype 26.0); W_s_: 4.3–5.9 (lectotype 5.8) (Kotor NHMW 110430/MN/0068, SMF 156967, SMF 221221). According to Westerlund (1884) the height and width of the smallest specimens (forma *parvula*) can be 15 mm and 3 mm, respectively.

##### Type locality.

“Cattaro” = Montenegro, Kotor.

##### Type material.

“Dalmatien, Cattaro”, ex Rossmässler, lectotype (SMF 156967), paralectotype (SMF 5021/3, SMF 176307/5 SMF 221221/9)

##### Other material.

Montenegro, Kotor, above the town, leg. Kiss, LP, 9.vii.1985 (HNHM 43127); Kotor, bus terminal, 42.4197°N, 18.7718°E, leg. ZE, ZF, 29.vi.1996 (HNHM 91037); Kotor, Škurda Gorge and N slope of the fortress hill, 80 m, 42.4269°N, 18.7756°E, leg. TD, ZE, ZF (HNHM 99601, NHMW 110430/MN/0068); Bay of Kotor, N of Dobrota, gorge of the Ljuta, 20 m, 42.4862°N, 18.7670°E, leg. LD, ZF, JK, DM, 8.x.2008 (HNHM 99599); Mt. Lovćen, gorge ca. 8 km from Kotor on the Kotor to Njeguši road, 400 m, 42.4024°N, 18.7750°E, leg. LD, ZF, JK, DM, 8.x.2008 (HNHM 99600); same locality, leg. ZE, ZF, 21.iv.2000 (HNHM 95412); same locality, leg. TD, ZE, ZF (HNHM 99602, NHMW 110430/MN/0069); 9 km from Kotor on the Kotor to Njeguši road, 450 m, 42.4095°N, 18.7787°E, leg. TD, ZE, ZF (HNHM 99603, NHMW 110430/MN/0070); “Risano über Bocche di Cattaro” (= Risan, above the Bay of Kotor) ex Käufel, ex Rušnov (NHMW Kau-70412); Bay of Kotor, Orahovac, 30 m, 42.4894°N, 18.7641°E, leg. TD, Domokos, Nacsa, Páll-Gergely, 5.iv.2006 (MMM-B01329).

##### Distribution.

**Figure 13.**
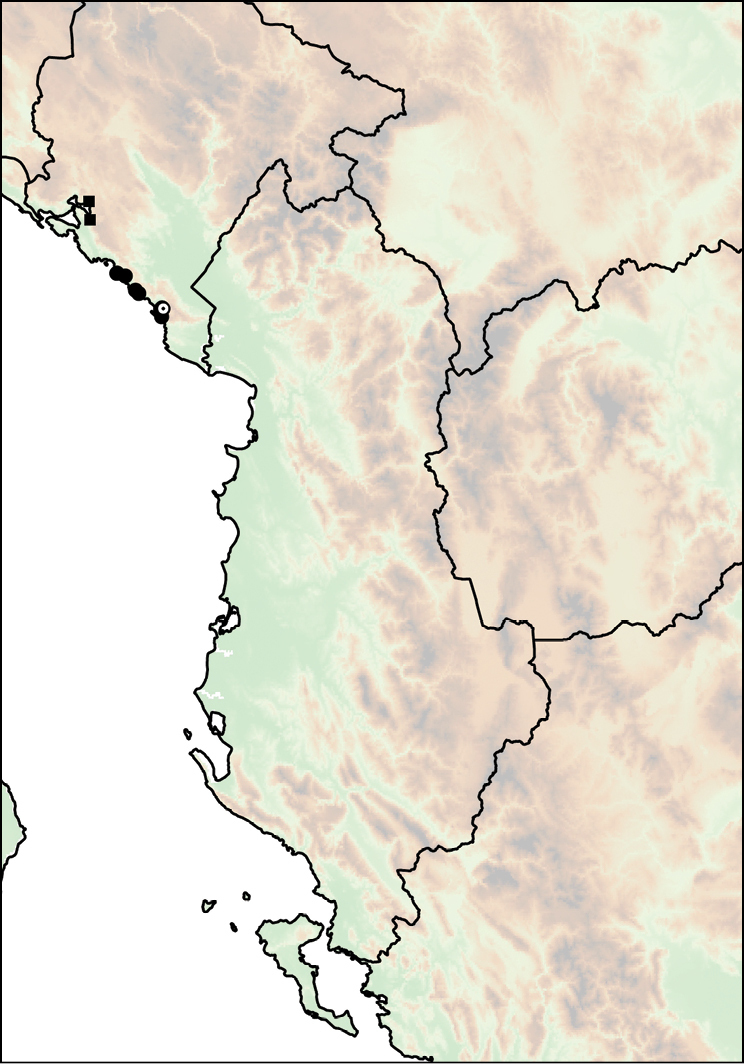
Distribution of *Montenegrina cattaroensis*. *Montenegrina cattaroensis cattaroensis* (square); *Montenegrina cattaroensis antivaricostata* (empty circle with dot); *Montenegrina cattaroensis umbilicata* (circle).

This taxon lives at the foot and slopes of the mountains surrounding the Bay of Kotor between Risan and Kotor. We found it along the serpentine road from Kotor to the Mt. Lovćen up to 450 m. It is not syntopic with *Montenegrina subcristata*, which occurs somewhat farther on the Njeguši Plateau, above 800–900 m (Fig. 13).

##### Remarks.

The monograph of Küster (1844-1862) was published in parts over several years. Page 40, where the name *gracilior* was first mentioned, dates to 1850 (Welter-Schultes 1999).

The distribution record from Herceg-Novi (“Castelnuovo”) by Schmidt (1868) is probably erroneous, as during recent field trips no *Montenegrina* could be found in that area. Gluhi do, mentioned by Wohlberedt (1901), is a locality of *Montenegrina subcristata*.

#### Montenegrina cattaroensis antivaricostata nom. n.

Taxon classificationAnimaliaStylommatophoraClausiliidae

Fig. 11E

Montenegrina cattaroensis antivaricostata non Clausilia costata Pfeiffer, 1828: 42, plate 7, figs 17–18.
Clausilia (Delima) umbilicata var. costata Boettger, 1907 in Wohlberedt 1907: 551. – Wohlberedt 1909: 674, plate 14, figs 156–157.
Delima (Albanodelima) umbilicata costata – Wagner 1924: 118.
Montenegrina umbilicata costata – Zilch 1981: 132, plate 12, fig. 18. – Nordsieck 2009: 73.

##### Diagnosis.

Shell medium, tumid. All whorls and the neck with widely-spaced and indistinct wrinkle-ribs. Basal and peripheral crests well recognizable. Lamellae superior and spiralis mostly overlap. Lunella dorsal-dorsolateral. Weak sulcalis usually present.

##### Dimensions

(in mm). H_s_: 14.4–23.1, W_s_: 4.5–6.0 (topotypes HNHM 83482).

##### Type locality.

“Am Saumpfade zwischen Antivari und Mikulić” = Montenegro, along the path between Stari Bar and Mikulić [*costata* (Boettger, 1907)].

##### Type material.

Type locality, ex Boettger, ex Wohlberedt, 1906, lectotype (SMF 176320), paralectotype (SMF 176321) [*costata* (Boettger, 1907)].

##### Other material.

Montenegro, Rumija Mts, 3 km above Stari Bar, on the footpath to Veliki Mikulići, 340 m, 42.0973°N, 19.1458°E, leg. LD, ZF, JK, DM, 14.x.2008 (HNHM 99612); same locality, leg. ZE, ZF, 19.iv.2000 (HNHM 83482); same locality, leg. MD, EH, KJ, HS, 7.vii.2015 (NHMW 110430/MN/0155).

##### Etymology.

The new replacement name refers to the historical name of Stari Bar (Antivari).

##### Distribution.

Western side of the Rumija Mts in Montenegro. This taxon was found only at the type locality, parapatrically with *Montenegrina cattaroensis umbilicata*. (There is a very narrow transitional contact zone between the two taxa). *Montenegrina cattaroensis umbilicata* is wider distributed in the area, and can be found along a large section of the footpath from Stari Bar to Veliki Mikulići (Fig. 13).

##### Remarks.

Although the description of *Clausila umbilicata costata* was formally published in Wohlberedt (1907), the authorship is unequivocally attributed to Boettger. This name is a primary junior homonym (ICZN Art. 53.3) of *Clausilia costata* Pfeiffer, 1828 (currently *Cochlodina costata*), therefore, it must be rejected and replaced by a new substitute name (ICZN Art. 60.3, 72.7).

#### Montenegrina cattaroensis umbilicata

Taxon classificationAnimaliaStylommatophoraClausiliidae

(Boettger, 1879)

Fig. 11F

Clausilia umbilicata Boettger, 1879: 102, plate 2, fig. 3.
Clausilia (Delima) umbilicata – Westerlund 1884: 53. – Wohlberedt 1907: 551. – Wohlberedt 1909: 674, plate 14, figs 152–155.
Delima (Albanodelima) umbilicata – Wagner 1924: 118.
Montenegrina umbilicata – Nordsieck 1969: 259. (genital anatomy).
Montenegrina umbilicata umbilicata – Zilch 1981: 132, plate 12, fig. 17. – Nordsieck 2009: 73.

##### Diagnosis.

Shell medium to large, tumid. All whorls smooth. Neck finely costate. Basal and peripheral crests weak. Lamellae superior and spiralis mostly overlap. Lunella dorsal-dorsolateral. Subclaustralis fused to lunella as its almost straight continuation. Plica principalis often connected to the superior. Weak sulcalis usually present.

##### Dimensions

(in mm). H_s_: 18.5–25.3 (lectotype 19.4), W_s_: 4.8–6.6 (lectotype 5.4) (lectotype SMF 176318, paralectotypes SMF 176319, topotypes HNHM 99604, Petrovac NHMW 110430/MN/0073, Haj Nehaj NHMW 110430/MN/0075).

##### Type locality.

“Antivari” = Montenegro, Stari Bar.

##### Type material.

Type locality, ex Boettger, ex Benoit, lectotype (SMF 176318), paralectotype (SMF 176319/5).

##### Other material.

Montenegro, Stari Bar, fortress, 130 m, 42.0939°N, 19.1342°E, leg. LD, ZF, JK, DM, 14.x.2008 (HNHM 99604); Rumija Mts, 2 km above Stari Bar, along the footpath to Veliki Mikulići, 320 m, 42.0978°N, 19.1447°E, leg. LD, ZF, JK, DM, 14.x.2008 (HNHM 99606); 1 km above Stari Bar, both sides of the gorge, 220 m, 42.0996°N, 19.1426°E, leg. LD, ZF, JK, DM, 14.x.2008 (HNHM 99605, HNHM 99607); Mt. Menka near Zaljevo, leg. Papp, 6.iv.1972 (HNHM 31109); Rijeka Reževići, along the Budva to Petrovac road, 100 m, 42.2270°N, 18.9117°E, leg. TD, ZE, ZF, 26.v.2015 (HNHM 99608, NHMW 110430/MN/0072); S of Novoselje, 3.5 km from Petrovac toward Virpazar, 400 m, 42.2178°N, 18.9545°E, leg. TD, ZE, ZF, 26.v.2015 (HNHM 99609, NHMW 110430/MN/0073); Čanj, valley of the Suvi Potok, road to the beach, 40 m, 42.1649°N, 19.0069°E, leg. TD, ZE, ZF, 26.v.2015 (HNHM 99610, NHMW 110430/MN/0074); Haj Nehaj (near Sutomore), quarry, 140 m, 42.1530°N, 19.0237°E, leg. TD, ZE, ZF, 26.v.2015 (HNHM 99611, NHMW 110430/MN/0075).

##### Distribution.

This taxon is distributed sporadically along the southern Montenegrin coast between Budva and Zaljevo (Fig. 13).

##### Remarks.

Earlier *Montenegrina cattaroensis umbilicata* used to be classified as a distinct species, but its geographical vicinity and very similar shell characters indicate close relationship with *Montenegrina cattaroensis*.

Wohlberedt (1909) mentions a more southern occurrence, in the Mužura Mts near Ulcinj. This record, however, corresponds to *Montenegrina subcristata* (leg. Winneguth, 1904, NHMW 41258/2).

#### Montenegrina chiasma

Taxon classificationAnimaliaStylommatophoraClausiliidae

Nordsieck, 1972

Fig. 11G

Montenegrina laxa chiasma Nordsieck, 1972: 30, plate 5, fig. 42.
Montenegrina chiasma – Nordsieck 2009: 73.

##### Diagnosis.

Shell medium, light violet-brown with whitish tint. Whorls flattened, smooth, only weakly inflexed neck costate. Basal and peripheral crests weak. Peristome broadly attached, ovoid, with somewhat swollen margin. Lamellae superior and spiralis do not overlap. In front view lamella inferior moderately emerged, medium-bent subcolumellaris hidden. Lunella dorsolateral, fused to the short basalis. Subclaustralis also short, sulcalis residual. Anterior plica superior mostly absent, if present short and separate from the lunella complex. Clausilium plate almost entirely visible through the aperture.

##### Dimensions

(in mm). H_s_: 16.1–18.0 (holotype 18.0), W_s_: 4.2–4.6 (holotype 4.5).

##### Type locality.

Albania, Tiranë District, N slope of Mt. Dajti, 1600 m.

##### Type material.

Type locality, ex Edlauer, paratypes (NHMW 75000/-E 16652/4); Mal i Dajtit, N-slope, 100 m beneath the summit, ex Edlauer, paratypes (NHMW 75000/-E 32343/4). The holotype (NHMW 76600) could not be found in the NHMW collection.

##### Distribution.

This taxon is known from the summit region of the Mt. Dajti (1613 m), east of Tiranë (Fig. 12).

##### Remarks.

The type material is from the collection of Edlauer and, according to the label information, it has been determined as *weigneri* by Käufel. Therefore, it could have been collected in the 1930s, possibly by Fuchs or Bischoff. Although the type locality seems to be limited to the summit region of the Mt. Dajti, we were unable to find this species there.

#### Montenegrina dofleini

Taxon classificationAnimaliaStylommatophoraClausiliidae

(Wagner, 1928)

##### Diagnosis.

Shell small to medium, light corneous. Neck variably inflexed, basal and peripheral crests weak. Peristome attached, its margin somewhat swollen. Lamellae superior and spiralis separate to overlapping. In front view lamella inferior visible, medium-bent subcolumellaris variably exposed. Lunella dorsal to dorsolateral. Basalis and subclaustralis weak to absent. If present, basalis not connected to the lunella. Anterior plica superior, if present, close and parallel to plica principalis, often connected to the lunella complex. Clausilium plate partly or entirely visible through the aperture.

#### Montenegrina dofleini dofleini

Taxon classificationAnimaliaStylommatophoraClausiliidae

(Wagner, 1928)

Fig. 11H, J

Delima (Montenegrina) dofleini Wagner, 1928 in Hesse 1928: 19.
Montenegrina dofleini dofleini – Nordsieck 1974: 153–154, plate 6, figs 31–32. – Zilch 1981: 127. – Nordsieck 2009: 74.
Montenegrina (Montenegrina) kaiseri Brandt, 1961: 4–5, plate 1, fig. 3.
Montenegrina dofleini kaiseri – Nordsieck 1974: 154, plate 6, fig. 34. – Zilch 1981: 127. – Nordsieck 2009: 74.

##### Diagnosis.

Shell medium, occasionally with whitish tint. Lower whorls smooth, upper ones very densely striate-costate. Neck weakly inflexed, densely striate-costate. Peristome rounded to ovoid. Lamellae superior and spiralis mostly overlap. In front view lamella inferior more or less well emerged, subcolumellaris mostly visible. Lunella dorsolateral, separate from the short basalis. Subclaustralis and sulcalis absent. Anterior plica superior mostly connected to the lunella complex.

##### Dimensions

(in mm). H_s_: 16.6–21.1, W_s_: 4.2–5.5 (Nordsieck 1974).

##### Type locality.

“Tomoros, 1500 m” = Macedonia, Galičica Mts, Tomoros Summit [*dofleini*]; Macedonia, Galičica Mts, W of Peštani, ca. 1800 m [*kaiseri*].

##### Type material.

“Tomoros, 1500 m”, leg. Doflein, 24.vii.1918, original series [*dofleini*], syntype status doubtful (ZSM 20150453/2); Peštani, E of the Tomoros Summit, 1800 m, leg. Kaiser, viii.1958, holotype [*kaiseri*] (ZMH 2926, ex SMF 163942), paratypes [*kaiseri*] (SMF 163943/4, SMF 201563, ZHM 2278/8, NMBE 9827/2, NHMW-K 44014/2).

##### Other material.

Macedonia, Ohrid District, Galičica Mts, ca. 4 km N of the junction at the Sveti Naum to Carina road, 1590 m, 40.9934°N, 20.8569°E, leg. ZF, EH, KJ, HS, 16.x.2014 (NHMW 110430/MN/0038); ca. 600 m E of the Bugarska Chuka, 1650 m, 41.0026°N, 20.8534°E, leg. ZF, EH, KJ, HS, 16.x.2014 (NHMW 110430/MN/0039); ca. 500 m E of the Bugarska Chuka, 1680 m, 41.0019°N, 20.8523°E, leg. ZF, EH, KJ, HS, 16.x.2014 (NHMW 110430/MN/0040); ca. 250 m E of the Bugarska Chuka, 1750 m, 41.0033°N, 20.8488°E, leg. ZF, EH, KJ, HS, 16.x.2014 (NHMW 110430/MN/0041); Bugarska Chuka, E side, 1800 m, 41.0037°N, 20.8470°E, leg. ZF, EH, KJ, HS, 16.x.2014 (NHMW 110430/MN/0042); ca. 300 m W of the Bugarska Chuka, 1740 m, 41.0046°N, 20.8432°E, leg. ZF, EH, KJ, HS, 16.x.2014 (NHMW 110430/MN/0043); ca. 200 m N of the Bugarska Chuka, 1800 m, 41.0051°N, 20.8462°E, leg. ZF, EH, KJ, HS, 16.x.2014 (NHMW 110430/MN/0044); N of the Magaro Summit, 1820 m, 40.9444°N, 20.8270°E, leg. ZE, ZF, JG, 29.vi.2015 (HNHM 99567, NHMW 110430/MN/0046); N of the Magaro Summit, 1870 m, 40.9439°N, 20.8283°E, leg. ZF, 29.vi.2015 (HNHM 99568, NHMW 110430/MN/0047); N of the Magaro Summit, 1900 m, 40.9429°N, 20.8252°E, leg. ZF, 29.vi.2015 (HNHM 99569, NHMW 110430/MN/0048); SE slope of the Galičica Mts, rocky forest along the Carina to Sveti Naum road, 1000–1200 m, leg. LP, PS, AS, 14.vii.1972 (HNHM 36717, SMF 227682); Elšani, above the Elešec Camp, 760 m, 41.0345°N, 20.8105°E, leg. ZF, KJ, HS, 17.x.2014 (NHMW 110430/MN/0045); same locality, leg. LP, PS, AS, 15.vii.1972 (HNHM 36737, HNHM 54405, HNHM 67012, SMF 22768); same locality, leg. ZE, ZF, AH, 7.iv.2004 (HNHM 94433).

##### Distribution.

**Figure 14.**
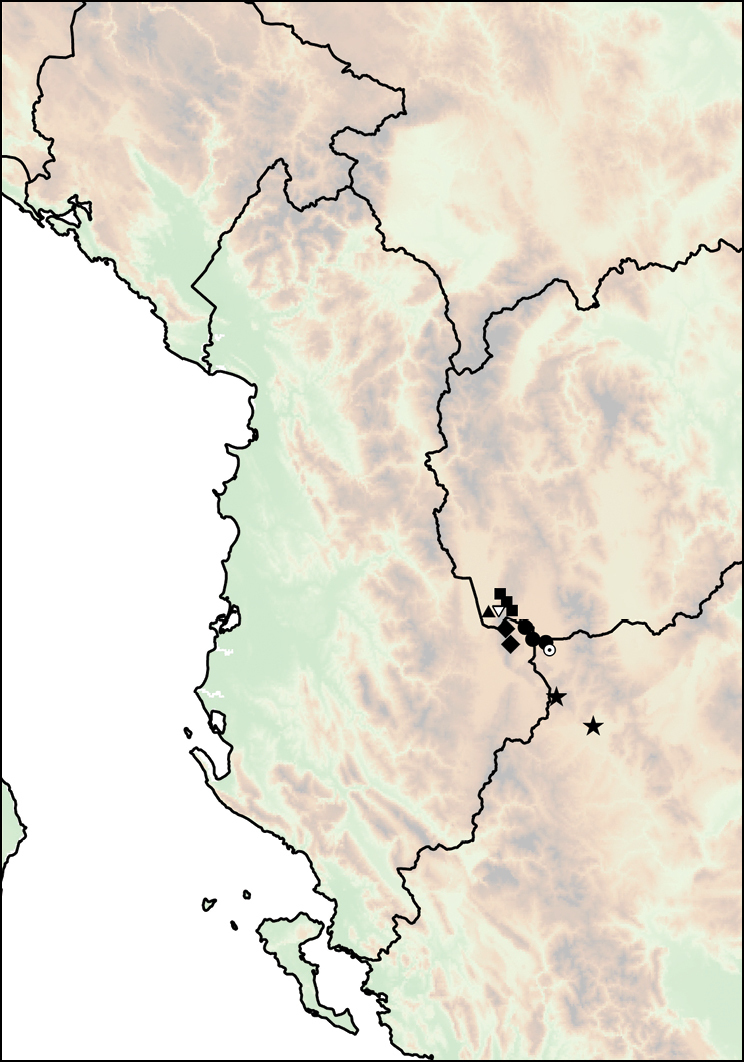
Distribution of *Montenegrina dofleini*. *Montenegrina dofleini dofleini* (square); *Montenegrina dofleini fagorum* (inverted empty triangle); *Montenegrina dofleini kastoriae* (star); *Montenegrina dofleini pinteri* (triagle); *Montenegrina dofleini prespaensis* (circle); *Montenegrina dofleini sinosi* (empty circle with dot); *Montenegrina dofleini wagneri* (diamond).

This taxon is frequent in the alpine region of the Galičica Mountain. We have found it on the eastern slope of the Bugarska Summit above 1600 m, and on the northern slope of the Magaro Summit above 1800 m. Although sporadically there are occurrences at lower altitudes (e.g. in Elšani at 750 m), on the western slopes of the Galičica, below 1600 m, it is replaced by *Montenegrina dofleini fagorum* Nordsieck, 1974 (Fig. 14).

##### Remarks.

According to Wagner (see: Hesse 1928), the material he studied consisted of six shells, only one of them in good condition, which had been dead-collected from the Tomoros, 1500 m, on July 24, 1918. In the ZSM collection there are only two shells, one of these relatively well preserved (Fig. 11H). These are apparently original specimens collected by Doflein. Although no material could be found in the MIZPAS collection (Dominika Mierzwa, personal communication), there is no evidence that the ZSM specimens are part of the material that had been studied by Wagner. Therefore, the syntype status of this sample is doubtful.

Although not synonymizing formally, Nordsieck (2009) has already questioned the subspecific status of *kaiseri*. Two arguments seem to support Nordsieck’s opinion that *kaiseri* could be regarded as junior synonym of *dofleini*. First, according to the label information, the type series of *kaiseri* were collected in the vicinity of the Tomoros Summit, which is the type locality of *dofleini*. Second, the paratypes of *kaiseri* show high variation in the traits defined by Brandt as distinguishing features.

In the original description the type locality of *kaiseri* was erroneously defined as ‘W of Peštani’ (Brandt 1961), because the Galičica range is east of this village.

The type series of *kaiseri* was collected by Kaiser, the curator of the ZMH Mollusc Collection at that time, and the entire lot was originally housed in the ZMH. As indicated in the description, Brandt (1961) intended depositing the holotype in the SMF. As this would have been done without authorization, the type was returned to the ZMH (Zilch 1981, Bernhard Hausdorf, personal communication).

#### Montenegrina dofleini fagorum

Taxon classificationAnimaliaStylommatophoraClausiliidae

Nordsieck, 1974

Fig. 11I

Montenegrina janinensis fagorum Nordsieck, 1974: 152, plate 5, fig. 30. – Zilch 1981: 129, plate 13, fig. 27. – Nordsieck 2009: 75.

##### Diagnosis.

Shell small, tumid, with whitish tint. All whorls smooth and glossy. Neck inflexed, behind the aperture finely striate. Peristome rounded to ovoid. Lamellae superior and spiralis mostly do not overlap. In front view lamella inferior moderately emerged, subcolumellaris not or only slightly barely visible. Lunella dorsal-dorsolateral, basalis and subclaustralis absent. Sulcalis mostly absent or residual. Anterior plica superior absent or weak, not connected to the lunella complex.

##### Dimensions

(in mm). H_s_: 11.8–14.4 (holotype 13.5), W_s_: 3.4–4.0 (holotype 3.7).

##### Type locality.

Macedonia, Galičica Mts, above Trpejca, 10 km toward Carina along the road crossing the Galičica Mts, 1400 m.

##### Type material.

Type locality, leg. Nordsieck, 26.viii.1971, holotype (SMF 227680), paratypes (SMF 227681, NHMW-K 65049/10, SMNS-N 5483), same locality, leg. Fauer, 17.viii.1971, paratypes (SMNS-N 5571, NMBE 535049/3), same locality, leg. LP, PS, AS, 14.vii.1972, paratypes (NMBE 535047/8, HNHM 11915/15, HNHM 67026/2, HNHM 36721/8).

##### Other material.

Macedonia, near the type locality, Korita Spring, 40.9674°N, 20.8149°E, leg. ZF, EH, KJ, HS, 17.x.2014 (NHMW 110430/MN/0053); same locality, leg. ZE, ZF, AH, 6.iv.2004 (HNHM 94244); Galičica Mts, 12 km from the Sveti Naum to Ohrid road toward Carina, 1540 m, 40.9627°N, 20.8171°E, leg. A. Fehér, T. Fehér, ZF, LT (NHMW 110430/MN/0052); same locality, leg. ZE, ZF, AH, 6.iv.2004 (HNHM 94450); 14 km from the Sveti Naum to Ohrid road toward Carina, Baba observation point, 1600 m, 40.9541°N, 20.8126°E, leg. ZE, ZF, JG, 29.vi.2015 (NHMW 110430/MN/0054); same locality, leg. ZE, ZF, AH, 6.iv.2004 (HNHM 94451).

##### Distribution.

This taxon inhabits the western slopes of the Galičica Mountain. It was found along the road crossing the mountain from ca. 1300 m to the mountain pass at 1600 m. Nordsieck (1974) mentions this taxon from the Mt. Thatë (southern extension of the Galičica), however those records correspond to another taxon, *Montenegrina dofleini wagneri* Szekeres, 2006. Our data suggest that the ranges of *fagorum* and *wagneri* are disjunct, as we found typical *dofleini* populations between them in the Magaro Summit area (NHMW 110430/MN/0046–48).

#### Montenegrina dofleini kastoriae

Taxon classificationAnimaliaStylommatophoraClausiliidae

Nordsieck, 1972

Fig. 11K

Montenegrina kastoriae Nordsieck, 1972: 34, plate 4, fig. 33.
Montenegrina dofleini kastoriae – Nordsieck 1974: 153. – Zilch 1981: 127, plate 14, fig. 35.
Montenegrina kastoriae kastoriae – Nordsieck 2009: 74.

##### Diagnosis.

Shell medium, elongate, whitish-corneous. Lower whorls smooth to indistinctly ribbed, upper ones very densely striate to costate. Neck weakly inflexed, behind the aperture striate to costate. Peristome ovoid to angular. Lamellae superior and spiralis overlap. In front view lamella inferior moderately emerged, subcolumellaris not or barely visible. Lunella dorsal to dorsolateral, weaker toward the basis. Basalis rarely recognizable, never connected to the lunella. Subclaustralis and sulcalis absent. Anterior plica superior mostly does not reach the lunella complex.

##### Dimensions

(in mm). H_s_: 14.4–20.4 (holotype 19.0), W_s_: 3.8–4.6 (holotype 4.5).

##### Type locality.

Greece, Western Macedonia, Kastoria, S side of the peninsula.

##### Type material.

Type locality, leg. H. Nordsieck, 24.viii.1971, holotype (SMF 221042), paratypes (SMF 221043, NHMW-K 65048/10, SMNS-N 5479).

##### Other material.

Greece, Western Macedonia, Kastoria, N side of the peninsula, leg. H. Nordsieck, 24.viii.1971 (SMNS-N 5477, NHMW 77938/5); same locality, behind Yacht Club, 640 m, 40.5233°N, 21.2747°E, leg. ZE, ZF, JG, 28.vi.2013 (HNHM 99570); same locality, leg. ZF, EH, KJ, HS, 17.x.2014 (NHMW 110430/MN/0050); Florina District, Krystallopigi, above the cemetery, 1120 m, 40.6357°N, 21.0903°E, leg. ZE, ZF, JG, 29.vi.2013 (HNHM 99571); same locality, leg. ZF, EH, KJ, HS, 17.x.2014 (NHMW 110430/MN/0049).

##### Distribution.

Western part of the Verno Mts in Western Macedonia Province, Greece. Originally this taxon was known only from Kastoria, but recently it has also been found at Krystallopigi, ca. 20 km northwest of the type locality (Fig. 14).

#### Montenegrina dofleini pinteri

Taxon classificationAnimaliaStylommatophoraClausiliidae

Nordsieck, 1974

Fig. 11L

Montenegrina janinensis pinteri Nordsieck, 1974: 154, plate 6, fig. 33.
Montenegrina dofleini pinteri – Zilch 1981: 127, plate 15, fig. 39. – Nordsieck 2009: 74.

##### Diagnosis.

Shell medium, elongate, whitish-corneous. Lower and upper whorls with dense, sharp ribs. Neck not or barely inflexed, ribs behind the aperture denser and finer. Basal and peripheral crests very weak. Peristome rounded to somewhat angular. Lamellae superior and spiralis long overlap. In front view lamella inferior moderately emerged, subcolumellaris mostly not visible. Lunella dorsal-dorsolateral, separate from the short basalis. Subclaustralis and sulcalis absent. Anterior plica superior mostly connected to the lunella complex.

##### Dimensions

(in mm). H_s_: 14.8–18.9 (holotype 16.9), W_s_: 4.1–5.0 (holotype 4.4).

##### Type locality.

Macedonia, Trpejca, north of boat harbor.

##### Type material.

Type locality, leg. LP, PS, AS, 16.vii.1972, holotype (SMF 227684), paratypes (SMF 227685, SMF 265700/4, SMNS-N 6146, NMBE 535039/118, ZMH 91605/3, HNHM 11960/272, HNHM 37763/207, HNHM 37762/3, HNHM 36708/3, HNHM 52687/10, HNHM 67011/6).

##### Other material.

Macedonia, Ohrid District, N of Trpejca, shore of the Lake Ohrid, 700 m, 40.9656°N, 20.7858°E, leg. ZF, 9.viii.2014 (NHMW 110430/MN/0051); same locality, leg. ZE, ZF, AH, 6.iv.2004 (HNHM 94438); Sv. Zaum, shore of Lake Ohrid, 40.949°N, 20.774°E (DED).

##### Distribution.

This taxon is known only from the vicinity of Tpejca at the shore of the Lake Ohrid (Fig. 14). At the type locality it occurs in the immediate vicinity of *Montenegrina stankovici*. The two taxa can often be found on the same cliffs, with *Montenegrina stankovici* preferring the lowest regions directly above the water surface, and *Montenegrina dofleini pinteri* inhabiting higher parts.

##### Remarks.

See considerations on niche segregation at *Montenegrina stankovici*.

#### Montenegrina dofleini prespaensis

Taxon classificationAnimaliaStylommatophoraClausiliidae

Nordsieck, 1988

Fig. 11M

Montenegrina dofleini prespaensis Nordsieck, 1988: 199–200, fig. 4.
Montenegrina dofleini ssp. – Nordsieck 1974: 153.
Montenegrina kastoriae prespaensis – Nordsieck 2009: 74.

##### Diagnosis.

Shell medium, elongate. Lower whorls smooth to indistinctly wrinkled, upper ones almost smooth to bluntly costate. Neck very weakly inflexed, behind the aperture striate to finely costate. Peristome attached, ovoid. Lamella superior weak, with spiralis does not overlap. In front view lamella inferior moderately emerged, subcolumellaris mostly not visible. Lunella dorsal-dorsolateral, weaker toward the basis. Basalis and subclaustralis mostly absent, residual sulcalis occasionally recognizable. Anterior plica superior absent or, if present, occasionally connected to the lunella complex.

##### Dimensions

(in mm). H_s_: 13.9–19.6 (holotype 16.7), W_s_: 3.6–4.5 (holotype 3.9).

##### Type locality.

Greece, Western Macedonia, Psarades, opposite to the village.

##### Type material.

Type locality, leg. HS, 1986, holotype (NHMW 84019), paratypes, (NHMW 84020/46); Psarades, peninsula NW of the village, leg. HS, 1985, paratypes (NHMW 84021/6, SMNS-N 9519).

##### Other material.

Greece, Psarades, N of the village, 860 m, 40.8315°N, 21.0288°E, leg. PS, 10.v.1995 (HNHM 12040); Psarades, E side of the bay, 870 m, 40.8316°N, 21.0287°E, leg. ZE, ZF, JG, 28.vi.2013 (HNHM 99573); same locality, leg. ZF, EH, KJ, HS, 18.x.2014 (NHMW 110430/MN/0056); SW of Psarades, Panagia Eleousa cave temple, 850 m, 40.8087°N, 20.9979°E, leg. ZF, EH, KJ, HS, 18.x.2014 (NHMW 110430/MN/0057); Albania, Korçë District, Kallamas, 850 m, 40.8924°N, 20.9394°E, leg. ZF, EH, KJ, HS, 17.x.2014 (NHMW 110430/MN/0055); same locality, leg. ZE, ZF, JG, 26.vi.2015 (HNHM 99575); Sveta Marena cave temple ca. 4 km E of Kallamas, 850 m, 40.8872°N, 20.9729°E, leg. ZE, ZF, JG, 29.6.2015 (NHMW 110430/MN/0134); 2 km SE of Kallamas, 850 m, 40.8809°N, 20.9590°E, leg. ZE, ZF, JG, 29.6.2015 (NHMW 110430/MN/0135).

##### Distribution.

Lake Prespa area, with sporadical occurrences are along the western and southern shores. Occasinally sympatric, but not syntopic, with *Montenegrina hiltrudae sattmanni* Nordsieck, 1988 and *Montenegrina hiltrudae desaretica* ssp. n. (Fig. 14).

##### Remarks.

From the confluence of the Great and Small Prespa Lakes Nordsieck (1988) mentions a further lot of paratypes (NHMW 84022/3) which, in fact, belongs to *Montenegrina dofleini sinosi* (see there).

#### Montenegrina dofleini sinosi

Taxon classificationAnimaliaStylommatophoraClausiliidae

Páll-Gergely, 2010

Fig. 11N

Montenegrina dofleini sinosi Páll-Gergely, 2010: 150–152, figs 1–2. (genital anatomy, clausilium plate).

##### Diagnosis.

Shell medium, elongate. Lower and upper whorls strongly wrinkled or ribbed. Neck very weakly inflexed, behind the aperture densely striate-costate. Peristome ovoid. Lamella superior weak, does not overlap spiralis. In front view lamella inferior moderately emerged, subcolumellaris mostly hidden. Lunella dorsal-dorsolateral, weaker toward the basis. Basalis and subclaustralis mostly absent, residual sulcalis occasionally recognizable. Anterior plica superior absent or, if present, occasionally connected to the lunella complex.

##### Dimensions

(in mm). H_s_: 13.9-16.3 (holotype 15.0), W_s_: 3.4-3.7 (holotype 3.5 mm).

##### Type locality.

Greece, Western Macedonia, southern shore of the Prespa Lake, Agios Achillios, near the junction to Psarades, 850 m, 40.8105°N, 21.0702°E.

##### Type material.

Type locality, leg. Páll-Gergely, 25.v.2009, holotype (HNHM 97145), paratypes (HNHM 97146/3, SMF 333885/3, NMBE 28607/2, NHMW 107878/2); at the confluence of the Great and Small Prespa Lakes, leg. HS, 21.v.1986, paratypes [*prespaensis*] (NHMW 84022/3).

##### Other material.

Type locality, leg. ZE, ZF, JG, 28.vi.2013 (HNHM 99580); same locality, leg. ZF, EH, KJ, HS, 18.x.2014 (NHMW 110430/MN/0058).

##### Distribution.

The subspecies is known only from its type locality, which is only at 4 km distance from the nearest occurrence of *Montenegrina dofleini prespaensis*. Potentially suitable intermediate localities have not been searched, therefore it is not clear whether there are transitional populations between the two taxa (Fig. 14).

##### Remarks.

Morphologically *Montenegrina dofleini sinosi* might be an extremely ribbed morph of the closely related *Montenegrina dofleini prespaensis*. Its distinct subspecific status remains questionable until the finer distribution pattern and population genetic relationship of the two taxa becomes better known.

#### Montenegrina dofleini wagneri

Taxon classificationAnimaliaStylommatophoraClausiliidae

Szekeres, 2006

Fig. 11O

Montenegrina apfelbecki wagneri Szekeres, 2006 in Erőss et al. 2006: 186–188, fig. 6.

##### Diagnosis.

Shell small, with whitish tint. All whorls smooth and glossy. Neck very weakly inflexed, behind the aperture finely striate. Basal and peripheral crests very weak. Peristome ovoid. Lamellae superior and spiralis do not overlap. In front view lamella inferior barely emerged. Deep subcolumellaris visible only in obliquely through the aperture. Lunella dorsolateral. Basalis mostly, subclaustralis always absent, sulcalis strong. Anterior plica superior usually absent or, if present, residual and connected to the lunella complex.

##### Dimensions

(in mm). W_s_: 13.2–14.9 (holotype 14.9), W_s_: 3.4–4.3 (holotype 4.2).

##### Type locality.

Albania, Mt. Thatë.

##### Type material.

“Mal i That”, leg. Fuchs, holotype (NHMW 103375), paratypes (NHMW 47961/2).

##### Other material.

Albania, Korçë District, Mt. Thatë, E of Podgorije, ca. 1.3 km E of the Bregu i Stanit Summit, 1830 m, 40.8255°N, 20.8528°E, leg. ZB, CN, DP, 20.v.2007 (HNHM 99576); ca. 4.3 km NW of Liqenas, ca. 700 m south of a summit, 1910 m, 40.8183°N, 20.8728°E, leg. ZB, DP, 22.v.2007 (HNHM 99577); ca. 4.3 km NW of Liqenas, 400 m ESE of a summit, 1780 m, 40.8220°N, 20.8789°E, leg. ZB, CN, DP, 22.v.2007 (HNHM 99578); along the path from Goricë to the ridge, 40.8485°N, 20.8624°E, leg. ZB, CN, Schmotzer, 11.viii.2008 (HNHM 99579); near the ‘Tower of the war’, alpine rocky grassland, 1750 m, 40.9122°N, 20.8498°E, leg. ID, 25.v.2013 (DED); same locality, 1810–1830 m, 40.9136°N, 20.8419°E, leg. ID, 24.ix.2013 (DED); Plaja e Pusit Summit, 2200 m, 40.8799°N, 20.8419°E, leg. ID, 25.vi.2013 (DED).

##### Distribution.

Alpine region (above 1700 m) of the Mt. Thatë in southern Albania. Dedov found this taxon syntopically with *Montenegrina hiltrudae fusca* Fehér & Szekeres, 2006 in the ‘Tower of the war’ area, near the Albanian–Macedonian border (Fig. 14).

##### Remarks.

Due to misinterpretation of the label information, the original description erroneously indicated the type locality as “Maja e Madhe (1796 m), WNW of Peshkopi”.

In Nordsieck’s view (Nordsieck 2009) *Montenegrina dofleini wagneri* is synonymous with *Montenegrina dofleini fagorum*. However, in our opinion their distinct subspecies status is well supported by stable differences in shell morphology.

#### Montenegrina drimmeri

Taxon classificationAnimaliaStylommatophoraClausiliidae

Fehér & Szekeres, 2006

Fig. 15N

Montenegrina drimmeri Fehér & Szekeres, 2006 in Erőss et al. 2006: 182–184, fig. 1. – Nordsieck 2009: 73.

##### Diagnosis.

Shell small, tumid. Lower whorls very pale, almost colorless. All whorls costate, ribs stronger, sharper and wider spaced toward the apex. Neck deep inflexed, densely costate. Basal and peripheral crests strong. Peristome attached, rounded to somewhat angular, with wide, simple margin. Lamellae superior and spiralis mostly do not overlap. In front view lamella inferior barely emerged, medium-bent subcolumellaris hidden, often not at all visible through the aperture. Plica principalis fused to the superior. Lunella dorsolateral, connected to the basalis. Subclaustralis shorter than the basalis, sulcalis residual or absent. Anterior plica superior separate from the lunella complex. Clausilium plate only barely visible through the aperture.

##### Dimensions

(in mm). H_s_: 11.1–15.4 (holotype 12.6), W_s_: 3.8–4.7 (holotype 4.5).

##### Type locality.

Albania, Dibrë District, Lunarë, at the bridge of the Lumi i Murrës (22 km W of Fushë-Muhurr along the Peshkopi to Burrel road) 730 m, 41.6256°N, 20.2498°E.

##### Type material.

Type locality, leg. ZE, ZF, JK, DM, 26.vi.2003, holotype (HNHM 94829), paratypes (HNHM 94830/18, NHMW 103271, RMNH 100312, SMF 328075, NMBE 534905).

##### Other material.

Type locality, leg. ZF, DM, ZU, 18.v.2010 (HNHM 99636).

##### Distribution.

Southern part of the Lura-Dejë mountain group in northern Albania. Known only from the type locality (Fig. 16).

##### Remarks.

Nordsieck (2009) placed *Montenegrina drimmeri* into the same species-group with *Montenegrina fuchsi*.

**Figure 15.**
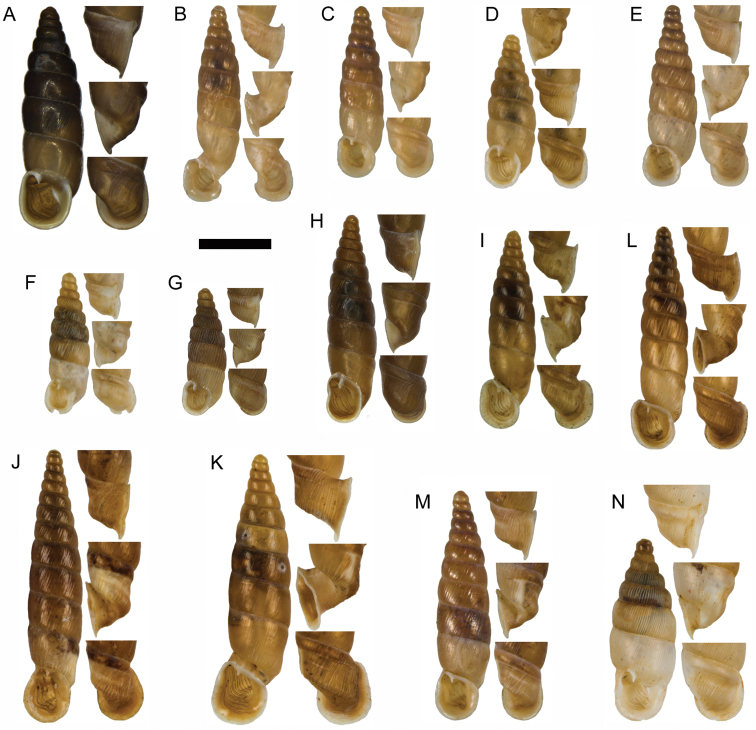
**A** *Montenegrina haringae* sp. n., holotype, NHMW 111219 **B** *Montenegrina fuchsi fuchsi*, Brandt, 1961, holotype, SMF 163939 **C** *Montenegrina fuchsi klemmi* Brandt, 1962, holotype, SMF 167014 **D** *Montenegrina fuchsi muranyii* Fehér & Szekeres, 2006, holotype, HNHM 94846 **E** *Montenegrina fuchi pallida* Fauer, 1993, holotype, SMF 309235 **F** *Montenegrina grammica grammica*, Nordsieck, 1988, holotype, NHMW 84026 **G** *Montenegrina grammica erosszoltani* ssp. n., holotype, HNHM 99512 **H** *Montenegrina grammica improvisa* ssp. n., holotype, HNHM 99518 **I** *Montenegrina helvola helvola* (Küster, 1860), Mt. Krujë, HNHM 86052 **J** *Montenegrina helvola carinata* Erőss & Szekeres, 1996, holotype, HNHM 70836 **K** *Montenegrina helvola magna* Fehér & Szekeres, 2006, holotype, HNHM 94848 **L** *Montenegrina helvola ornata* Erőss & Szekeres, 1996, holotype, HNHM 70838 **M** *Montenegrina helvola pageti* Brandt, 1962, holotype, SMF 167016 **N** *Montenegrina drimmeri* Fehér & Szekeres, 2006, holotype, HNHM 94829. Scale bar: 5 mm.

#### Montenegrina fuchsi

Taxon classificationAnimaliaStylommatophoraClausiliidae

Brandt, 1961

##### Diagnosis.

Shell very small to small, light corneous, whorls smooth to indistinctly wrinkled-costate. Neck deep inflexed, behind the aperture costate. Basal crest strong, peripheral crest well visible to very strong. Peristome with simple margin, narrowly attached to detached and projected. In front view lamella inferior moderately emerged, broadly-bent subcolumellaris not or only barely visible. Lunella dorsal to dorsolateral, subclaustralis short. Anterior plica superior not connected to the lunella complex. Clausilium plate partly visible through the aperture.

#### Montenegrina fuchsi fuchsi

Taxon classificationAnimaliaStylommatophoraClausiliidae

Brandt, 1961

Fig. 15B

Montenegrina (Heteroptycha) fuchsi Brandt, 1961: 2–3, plate 1, fig. 1.
Montenegrina fuchsi fuchsi – Zilch 1981: 128, plate 13, fig. 25. – Nordsieck 2009: 73.

##### Diagnosis.

Shell surface indistinctly wrinkled, neck densely costate. Peripheral crest very strong. Peristome detached, strongly projected, rounded to pear-shaped. Lamella subcolumellaris visible through the aperture. Plica principalis often fused to the superior. Lunella dorsal, sulcalis well developed.

##### Dimensions

(in mm). H_s_: 13.6–13.7, W: 2.9–3.0 (width of the penultimate whorl).

##### Type locality.

“An der Straße von Tepelene nach Korzyra” = Albania, valley of the Vjosë River between Kelcyrë and Tepelenë.

##### Type material.

Type locality, ex Käufel, ex Zilch, holotype (SMF 163939); Type locality, leg. Fuchs, 1936, paratypes (NHMW-K 4491/3, NMBE 9825/2, ZMH 113581/3).

##### Other material.

Albania, right bank of the Vjosë at the bridge along the Tepelenë to Kelcyrë road, leg. Fuchs, 1936 (NHMW-K 47960, NHMW-K 32317, NHMW-K 48293, NHMW 21443, ZSM 20150454); “Shendeli bei Tepelenë, 1000 m”, leg. Fuchs (NHMW-K 3238, NHMW-K 48292); Tepelenë District, 7 km W of Këlcyre, near the bridge at the Peshtan junction, 170 m, 40.2961°N, 20.1076°E, leg. ZE, ZF, 6.vii.1996 (HNHM 86048); same locality, leg. ZE, ZF, KK, 13.iv.2001 (HNHM 85949), same locality, leg. PJ, TK, DM, GP, 14.x.2013 (HNHM 99563); same locality, leg. DA, ZE, ZF, JG, 29.vi.2014 (HNHM 99333, NHMW 110430/MN/0036).

##### Distribution.

Southern part of the Mt. Shëndel in southern Albania.

**Figure 16.**
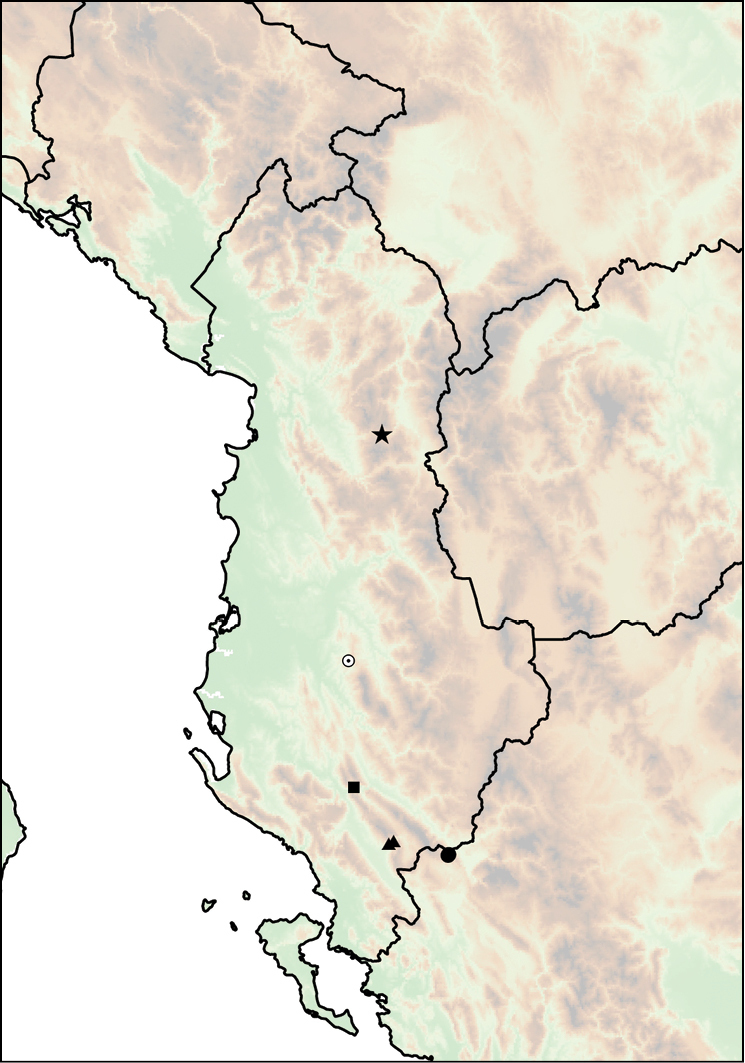
Distribution of *Montenegrina drimmeri* and *Montenegrina fuchsi*. *Montenegrina drimmeri* (star); *Montenegrina fuchsi fuchsi* (square); *Montenegrina fuchsi klemmi* (triangle); *Montenegrina fuchsi muranyii* (empty circle with dot); *Montenegrina fuchsi pallida* (circle).

Most of the museum lots of this taxon are from the valley of the Vjosë River, west of Kelcyrë, at the foot of the Mt. Shëndel. All of these are probably from the same locality, near the bridge to Peshtan. However, the record by Fuchs (“Shendeli bei Tepelene, 1000 m”) indicates a presumably wider distribution in the Shëndel, north of the type locality (Fig. 16).

##### Remarks.

Franz Käufel apparently intended to describe this as a new species, and started to distribute material as types. The HNMW 21443 lot contains a handwritten label from Käufel with the name *fuchsi*, and the indication “Paratypen”. But Käufel’s manuscript has never been published and this taxon was formally described only by Brandt in 1961. Therefore, only the lots designated in Brandt’s (1961) description are considered type material.

#### Montenegrina fuchsi klemmi

Taxon classificationAnimaliaStylommatophoraClausiliidae

Brandt, 1962

Fig. 15C

Montenegrina (Heteroptycha) fuchsi klemmi Brandt, 1962: 143, plate 5, fig. 13.
Montenegrina fuchsi klemmi – Zilch 1981: 128, plate 13, fig. 26. – Nordsieck 2009: 73.

##### Diagnosis.

Shell surface smooth to indistinctly wrinkled, neck irregularly costate. Peripheral crest strong. Peristome narrowly attached or detached, rounded. Lamella subcolumellaris barely visible through the aperture. Lunella dorsal-dorsolateral, sulcalis well developed.

##### Dimensions

(in mm). H_s_: 10.4–14.0, W_s_: 2.6–2.9.

##### Type locality.

“Suhe, Tal d. Lum i Suhes ö. Argyrokastro” = Albania, Gjirokastër District, Suhë, valley of Lumi i Suhës.

##### Type material.

Type locality, leg. Fuchs, 1931, holotype (SMF 167014), paratypes (SMF 167015/2, NHMW-E 32491/23, NMBE 9826/4, ZMH 113579/2).

##### Other material.

Type locality, ex Klemm (SMF 201621/2) [although mentioned as paratypes by Zilch (1981), the original description does not mention this lot]; same locality, without date (NHMW-K 48294/7) [although indicated on the label as type, the original description does not mention it]; Gjirokastër District, between Suhë and Poliçan, leg. Fuchs, 1936. (NHMW-E 32441, NHMW-K 48295); 3 km NE of Suhë, along the Libohovë to Sheper road, 430 m, 40.0882°N, 20.2716°E, leg. ZF, JK, DM, 12.x.2004 (HNHM 95049); same locality, leg. DA, ZE, ZF, JG, 27.vi.2014 (HNHM 99272, NHMW 110430/MN/0037); 5 km NE of Suhë, 420 m, 40.0915°N, 20.2922°E, leg. ZF, JK, DM, 12.x.2004 (HNHM 95050).

##### Distribution.

Lunxhëri Mts in southern Albania. Known only from the gorge of the Suhë Creek crossing the mountain east to west between Suhë and Poliçan (Fig. 16).

#### Montenegrina fuchsi muranyii

Taxon classificationAnimaliaStylommatophoraClausiliidae

Fehér & Szekeres, 2006

Fig. 15D

Montenegrina fuchsi muranyii Fehér & Szekeres, 2006 in Erőss et al. 2006: 189–190, fig. 9. – Nordsieck 2009: 73.

##### Diagnosis.

Shell very small, light corneous. All whorls smooth or very finely, indistinctly wrinkled. Neck weakly inflexed, densely wrinkled-costate. Basal and peripheral crests well recognizable. Peristome rounded to ovoid, with simple margin. Lamellae superior and spiralis do not overlap. In front view lamella inferior moderately emerged, subcolumellaris not visible. Lunella dorsolateral. Basalis absent or weak and separate from the lunella. Subclaustralis absent, sulcalis weak to residual. Anterior plica superior mostly present, not connected to the lunella complex. Clausilium plate not or only barely visible through the aperture.

##### Dimensions

(in mm). H_s_: 8.9–11.9 (holotype 10.9), W_s_: 2.8–3.4 (holotype 3.4).

##### Type locality.

Albania, Berat District, Tomorr Mts, Kalaja e Tomorrit, 1100 m, 40.7025°N, 20.1093°E.

##### Type material.

Type locality, leg. Harmos, DM, 26.v.2004, holotype (HNHM 94846), paratypes (NHMUK 20050219, HNC 63183, HNHM 94847/39, NHMW 103277, RMNH 100307, SMF 328081).

##### Distribution.

Tomorr Mts in southern Albania. This taxon is known only from the type locality, which is a western extension of the Tomorr (Fig. 16).

#### Montenegrina fuchsi pallida

Taxon classificationAnimaliaStylommatophoraClausiliidae

Fauer, 1993

Fig. 15E

Montenegrina fuchsi pallida Fauer, 1993: 54–55, plate 1, fig. 6. – Nordsieck 2009: 73.

##### Diagnosis.

Shell surface indistinctly wrinkled-costate, stronger at the upper whorls. Neck with sharp ribs and well visible recognizable peripheral crest. Peristome detached, projected, ovoid. Lamella subcolumellaris visible through the aperture. Lunella dorsal, sulcalis weak to residual.

##### Dimensions

(in mm). H_s_: 10.7–15.5 (holotype 12.5), W_s_: 2.8–4.1 (holotype 3.4).

##### Type locality.

Greece, Epirus, Pogonisko junction on the Konitsa to Molivdokepastos road, 500 m.

##### Type material.

Type locality, leg. Fauer, PS, 26.ix.1989, holotype (SMF 309235), paratypes (SMF 309236/2, NMBE 534897/115, SMNS-N 9792, NHMW 103730/1); same locality, leg. PS, 16.vii.1990, paratypes (NMBE 534896/95).

##### Other material.

Type locality, 40.0468°N, 20.5648°E, leg. ZE, ZF, JG, 26.vi.2013 (HNHM 99566).

##### Distribution.

Southern part of the Dousko (Nemërçkë) Mountain, in Epirus, northwestern Greece. Known only from the type locality (Fig. 16).

#### Montenegrina grammica

Taxon classificationAnimaliaStylommatophoraClausiliidae

Nordsieck, 1988

##### Diagnosis.

Shell very small to small, neck inflexed, with strong peripheral crest. Peristome attached, with swollen margin. Lamellae superior and spiralis do not or only occasionally overlap. In front view lamella inferior barely to moderately emerged, medium-bent subcolumellaris mostly not visible. Lunella broad, dorsolateral to lateral. Basalis absent or short, if present fused to or separate from lunella. Subclaustralis and sulcalis residual or absent. Anterior plica superior absent or weak and separate from the lunella complex. Differs from *Montenegrina janinensis* by the smaller, more conical and stronger sculptured shell, as well as the deeper inflexed neck and broadly-bent lamella subcolumellaris.

#### Montenegrina grammica grammica

Taxon classificationAnimaliaStylommatophoraClausiliidae

Nordsieck, 1988

Fig. 15F

Montenegrina janinensis grammica Nordsieck, 1988: 198–199, fig. 2. – Nordsieck 2009: 75.

##### Diagnosis.

Shell light corneous. Lower whorls smooth to indistinctly costate-weinkled, upper ones with strong ribs. Neck smooth to weakly wrinkled, with weak basal crest. Peristome rounded. Lamellae superior and spiralis do not overlap. In front view lamella inferior barely emerged, subcolumellaris mostly not visible. Lunella dorsolateral. Basalis absent or residual and fused to the lunella. Anterior plica superior mostly absent, rarely weak and separate from the lunella complex.

##### Dimensions

(in mm). H_s_: 9.8–10.3 (holotype 9.8), W_s_: 2.9–3.4 (holotype 3.1).

##### Type locality.

Greece, Gramos Mts, mountain ridge NNW of the Epano Arena, ca. 2000 m.

##### Type material.

Type locality, leg. HS, 3.vii.1986, holotype (NHMW 84026), paratypes (NHMW 84027/8).

##### Other material.

Greece, Gramos Mts, W of Epano Arena summit, 2000 m, 40.3090°N, 20.8981°E, leg. ZE, ZF, JG, 27.vi.2013 (HNHM 99628).

##### Distribution.

**Figure 17.**
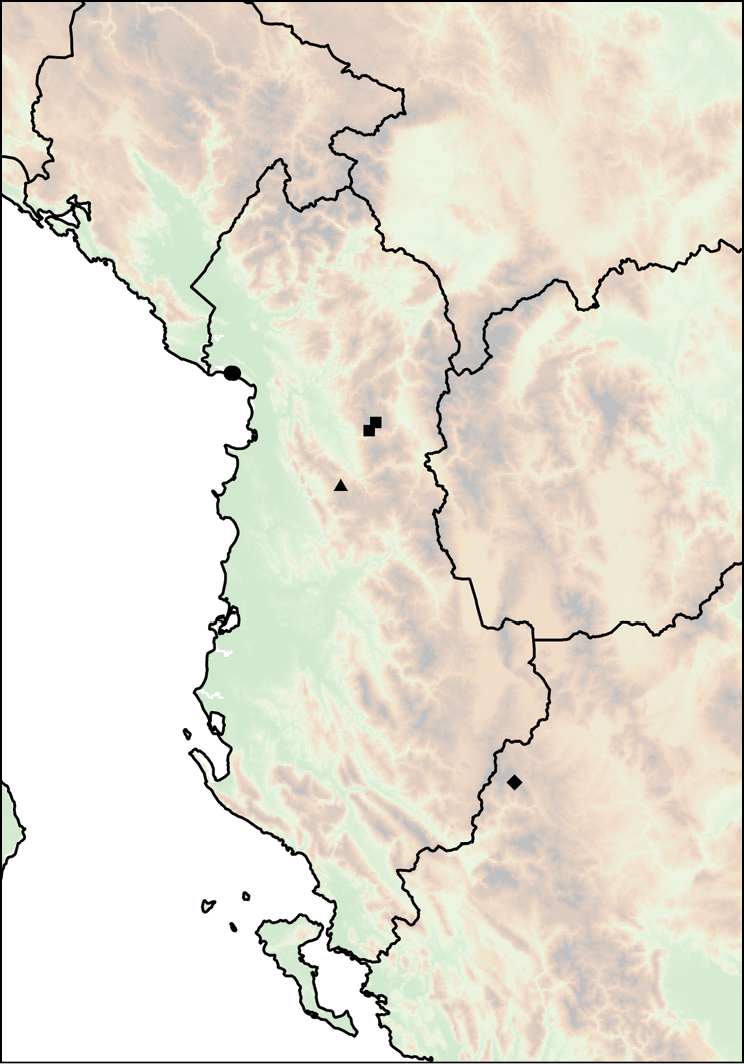
Distribution of *Montenegrina grammica* and *Montenegrina haringae* sp. n. *Montenegrina grammica erosszoltani* ssp. n. (square); *Montenegrina grammica grammica* (diamond); *Montenegrina grammica improvisa* ssp. n. (triangle); *Montenegrina haringae* sp. n. (circle).

Gramos Mts in northwestern Greece. Known only form the northern side of the Epano Arena. In that area it lives syntopically with the more frequent and wider distributed *Montenegrina skipetarica pindica* Nordsieck, 1988 (Fig. 17).

#### Montenegrina grammica erosszoltani ssp. n.

Taxon classificationAnimaliaStylommatophoraClausiliidae

http://zoobank.org/783B036F-077D-48CE-82F4-326AD5EC5B10

Fig. 15G

##### Diagnosis.

Very small subspecies with dorsolateral lunella, missing basalis and non-connected anterior plica superior.

##### Description.

The very small, somewhat conical, greyish-corneous shell consists of 7½ to 9 whorls. The entire surface is strongly costate, with denser ribs behind the aperture and toward the apex. The deep neck inflection extends to the lateral side. The basal and peripheral crests are well developed. The ovoid to circular aperture has broadly attached, simple margin. The lamellae superior starts at the same depth as that of the end of the spiralis, occasionally with a slight overlap. The lamella inferior and medium-bent subcolumellaris are only rarely and slightly visible in front view. The broad lunella is dorsolateral. The basalis and subclaustralis are missing, the sulcalis is present. The weak anterior part of the plica superior initiates far from the lunella complex. Only the parietal edge of the clausilium plate is visible through the aperture.

##### Dimensions

(in mm). Holotype H_s_: 8.9, W_s_: 3.0, H_a_: 2.4, W_a_: 2.1; paratypes (HNHM 99513, n = 12): H_s_: 8.5–11.4 (mean 9.5, S.D. 0.77), W_s_: 2.6–3.3 (mean 2.9, S.D. 0.21), H_a_: 2.3–2.9, W_a_: 1.9–2.4.

##### Differential diagnosis.

The new subspecies differs from *Montenegrina grammica grammica* by its strongly costate whorls and the presence of the anterior plica superior, whereas from *Montenegrina grammica improvisa* by its smaller size, costate surface, deeper ending lamella subcolumellaris, and missing basalis.

##### Type locality.

Albania, Mat District, W of the Shkëmb i Skanderbeut (1 km from the Peshkopi to Burrel road toward Fushë-Lurë), 1160 m, 41.6461°N, 20.1807°E.

##### Type material.

Type locality, leg. ZE, ZF, AH, DM, 13.iv.2006, holotype (HNHM 99512), paratypes (HNHM 99513/52, ER/52, HU/52, SZ/5); same locality, leg. ZE, ZF, 11.x.2005, paratypes (HNHM 99514/138+5aj, MMM-B01327/138, ER/138, SZ/10); same locality, leg. ZF, DM, ZU, 18.v.2010, paratypes (HNHM 99515/3a+75, NHMW 111233/10); on the Peshkopi to Burrel road, 1 km W of the Fushë-Lurë junction, 1090 m, 41.6451°N, 20.1823°E, leg. ZE, ZF, AH, DM, 13.iv.2006, paratypes (HNHM 99516/27, ER/27, HU/27, SZ/5); Mali i Dejës, limestone hill of 1700 m height above the headwaters of the Lumi i Varoshit, 1540 m, 41.6746°N, 20.2112°E, leg. ZF, DM, ZU, 18.v.2010, paratypes (HNHM 99517/3+1a, SZ/2).

##### Etymology.

The new subspecies is named after our friend and colleague Zoltán Erőss, who accompanied the first author on several Balkan field trips, including the one during which this taxon was discovered.

##### Distribution.

Southern part of the Lura-Dejë mountain group, within the catchment area of the Varosh Stream (Fig. 17).

##### Remarks.

At one of the localities it was found syntopically with *Montenegrina skipetarica puskasi* ssp. n.

#### Montenegrina grammica improvisa ssp. n.

Taxon classificationAnimaliaStylommatophoraClausiliidae

http://zoobank.org/05856D4B-F29C-423F-897F-0C5C90B4B854

Fig. 15H

##### Diagnosis.

Small subspecies with almost smooth shell, dorsolateral to lateral lunella, short, non-connected basalis, and missing anterior plica superior.

##### Description.

The medium-size, light brownish-corneous shell consists of 9½ to 11 whorls. The surface is smooth at the lower whorls and becomes weakly, indistinctly costate toward the apex. The neck is irregularly and finely wrikled-costate, and behind the aperture densely striate. The basal and peripheral crests are well developed. The peristome is attached, ovoid to somewhat angular, with slightly swollen margin. The lamellae superior and spiralis occasionally overlap. In front view the straight descending end of the lamella inferior is visible, the subcolumellaris is hidden. The lunella is broad, dorsolateral to lateral, separate from the short basalis. The subclaustralis is absent, the sulcalis is residual or also missing. The clausilium plate cannot be viewed through the aperture.

##### Dimensions

(in mm). Holotype H_s_: 14.4, W_s_: 3.8, H_a_: 3.6, W_a_: 2.4; paratypes (HNHM 99019, n = 12): H_s_: 12.8–14.4 (mean 13.7, S.D. 0.50), W_s_: 3.3–4.2 (mean 3.6, S.D. 0.26), H_a_: 3.0–3.6, W_a_: 2.0–2.6.

##### Differential diagnosis.

The new taxon can be distinguished from *Montenegrina grammica grammica* and *Montenegrina grammica erosszoltani* by its larger and smoother shell, deep-ending lamella subcolumellaris, deeper lunella, and the presence of a short basalis.

##### Type locality.

Albania, Mat District, Gropa Mts, 3.5 km W of Gurri i Bardhë, N slope of the Maja e Bastarit, 1160 m, 41.4361°N, 20.0485°E.

##### Type material.

Type locality, leg. ZF, TN, EM, 15.iv.2014, holotype (HNHM 99518), paratypes (HNHM 99019/29+18a+8aj, NHMW 111234/5, SZ/3).

##### Etymology.

The name *improvisa* refers to the unexpected, accidental discovery of the new subspecies.

##### Distribution.

Gropa Mts in Central Albania. Known only from the type locality (Fig. 17).

#### Montenegrina haringae sp. n.

Taxon classificationAnimaliaStylommatophoraClausiliidae

http://zoobank.org/6C3DD1FC-600F-4684-9AB3-1DE517A47F78

Fig. 15A

##### Diagnosis.

Small to medium-size species with broadly attached peristome, broadly-bent lamella subcolumellaris, dorsal lunella, and weak, separate basalis.

##### Description.

The small to medium-size, tumid, light corneous shell consists of 8 to 9½ whorls. The entire surface is opaque and smooth, except for the inflexed neck that is weakly costate behind the aperture. The basal and peripheral crests are weak. The aperture is ovoid, somewhat angular, broadly attached, with simple peristome margin. The long lamella superior only barely overlaps with the spiralis. In front view the lamella inferior is moderately emerged, the end of the broadly-bent subcolumellaris is usually visible. The dorsal lunella is separate from the weak, lump-like basalis. The subclaustralis is absent, the sulcalis is residual or also absent. The anterior part of the plica superior is absent or weak, non-parallel to the principalis, separate from the lunella complex. The narrow clausilium plate is partly visible through the aperture, its proximal half is obscured by the deep-emerged lamella inferior.

##### Dimensions

(in mm). Holotype H_s_: 14.8, W_s_: 4.3, H_a_: 3.6, W_a_: 2.9; paratypes (NHMW 111220, n =12): H_s_: 12.5–17.4 (mean 14.7, S.D. 1.44), W_s_: 3.7–4.8 (mean 4.4, S.D. 0.26), H_a_: 3.3–4.1, W_a_: 2.9–3.7 mm.

##### Differential diagnosis.

Compared to species of the surrounding coastal regions, the new taxon can be distinguished from both *Montenegrina cattaroensis* and *Montenegrina subcristata* by its smaller size, less deep lunella, missing subclaustralis and weak or absent anterior plica superior. From all other species of the genus differs by the combination of a dorsal lunella and the absence of subclaustralis.

##### Type locality.

Albania, Shkodër District, Mt. Renc, dry gorge S of Baks-Rjollë, (SW of the Maja e Zezë), 30 m, 41.8577°N, 19.4923°E.

##### Type material.

Type locality, leg. TD, ZE, ZF, 27.v.2015, holotype (NHMW 111219), paratypes (NHMW 111220/65, HNHM 99483/61, MMM-B01324/61, SZ/5); above Baks-Rjollë, S slope of the Maja e Zezë, 100 m, 41.8569°N, 19.5044°E, leg. ZB, DP, GP, 4.v.2014, paratypes (HNHM 99061/4, SZ/1).

##### Etymology.

The new species is named after Elisabeth Haring, head of the Central Research Laboratories at NHMW, who provided support and inspiration to this study.

##### Distribution.

Mt. Renc in northwestern Albania (Fig. 17).

#### Montenegrina helvola

Taxon classificationAnimaliaStylommatophoraClausiliidae

(Küster, 1860)

##### Diagnosis.

Shell small to large, light corneous. Whorls smooth to indistinctly costate. Neck deep inflexed, costate. Basal and peripheral crests strong. Peristome detached, projected, rounded to pear-shaped, with simple margin. Lamellae superior and spiralis long overlap. In front view lamella inferior high-ending, hidden to moderately emerged. Broadly-bent subcolumellaris retracted, not visible through the aperture. Lunella dorsolateral to ventrolateral. Basalis absent to long and fused to the lunella as its almost straight continuation. Subclaustralis absent or short, perpendicular to the lunella. Sulcalis absent or residual. Anterior plica mostly present, separate from the lunella complex. Clausilium plate not or only barely visible through the aperture.

#### Montenegrina helvola helvola

Taxon classificationAnimaliaStylommatophoraClausiliidae

(Küster, 1860)

Fig. 15I

**Figure 18.**
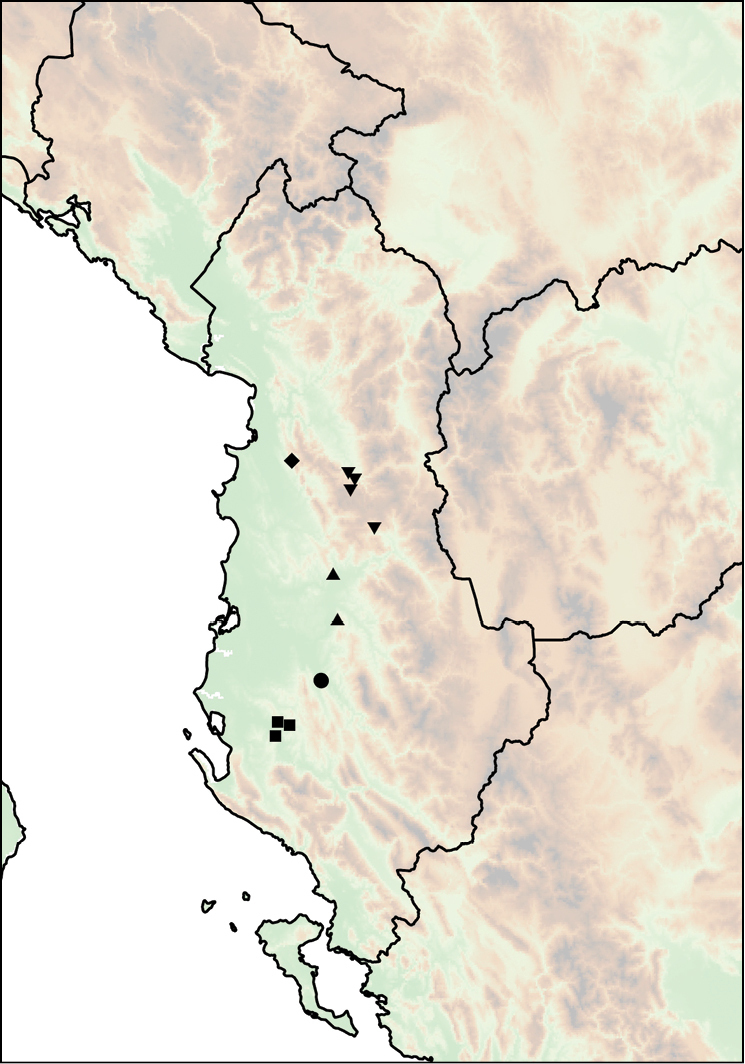
Distribution of *Montenegrina helvola*. *Montenegrina helvola carinata* (square); *Montenegrina helvola helvola* (diamond); *Montenegrina helvola magna* (inverted triangle); *Montenegrina helvola ornata* (triangle); *Montenegrina helvola pageti* (circle).

Clausilia helvola Küster, 1860 – Küster 1844-1862: 176, plate 19, figs 15–18. – Schmidt 1868: 70–71.
Clausilia (Heteroptycha) helvola – Westerlund 1884: 40.
Delima (Alpidelima) helvola – Wagner 1924: 120.
Delima (Heteroptycha) helvola – Zilch in Wenz 1960: 429–430, fig. 1527.
Montenegrina helvola helvola – Zilch 1981: 128. – Nordsieck 2009: 73.

##### Diagnosis.

Shell small to medium. Lower whorls smooth, upper ones indistinctly wrinkled-costate. Neck wrinkled-costate. Peristome rounded to pear-shaped. In front view lamella inferior very high, barely emerged. Lunella dorsolateral. Basalis absent or residual and fused to the lunella. Subclaustralis weak and short, sulcalis residual. Anterior plica superior short, occasionally absent. Clausilium plate barely visible through the aperture.

##### Dimensions

(in mm). H_s_: 12.5–19 (neotype: 18.5), W_s_: 3.5–4.6 (neotype: 4.5) (SMF 94110a, HNHM 86051).

##### Type locality.

Albania, Krujë, Skanderbeg Fortress (due to the present neotype designation). In the original description the locality was given as “Dalmatien”. According to Wagner (1924), the original material was collected in Slano (southern Dalmatia) from marine flotsam.

##### Type material.

Albania, Mali i Krujës, inside the Skanderbeg Castle, ex K. L. Pfeiffer, ex Fuchs, 1941, neotype (SMF 94110a) (Zilch in Wenz 1960: fig. 1527.).

##### Other material.

Same as that of the neotype (NHMW-E, NHMW-E 33201, NHMW-E 32547, NHMW-K 50602, NHMW-K 48291 NHMW-K 54388); Krujë, castle, 600 m, 41.5107°N, 19.7947°E, leg. ZE, ZF, KK, 10.iv.2001 (HNHM 86054); Mali i Krujës, S slope at 1200 m, “zum Derwischgrab”, leg. Fuchs (NHMW-E 16727/6); same locality at 1000 m, leg. Fuchs (NHMW-E 16501/3); Mali i Krujës, above Krujë, ca. 1000 m, 41.5222°N, 19.8055°E, leg. ZF, Kónya, 14.ix.1994 (HNHM 86051); same locality, leg. ZE, ZF, KK, 10.iv.2001 (HNHM 86052); Krujë, road to the Qafa e Shtamës, 41.5220°N, 19.7939°E, leg. AR, NR, PR, ix.2013 (NHMW 10.430/MN/0076); Mali i Krujës above Noi, along the Burrel to Krujë road, 700 m, 41.5229°N, 19.8164°E, leg. ZE, ZF, JK, DM, 26.x.2002 (HNHM 91606).

##### Distribution.

Mt. Krujë in central Albania. Known from the fortress of Krujë and some other sites on the Mt. Krujë, above and east of the town (Fig. 18).

##### Remarks.

The first volume of the Systematisches Conchylien-Cabinet (Küster 1844–1862) was published in parts. Plate 19, with a figure of *Montenegrina helvola* but without description, was printed in 1853. The description (at p. 176), however, was published only in 1860 (Welter-Schultes 1999).

Without specific type locality it was not possible to ascertain where Küster’s material originated from. Zilch (1960) depicted a specimen from Krujë, which was subsequently used as reference for the taxon without any nomenclatural relevance. The original type material could not be found in the public collections that currently hold remnants of Küster’s collection. Therefore, we can reasonably assume that it has been lost. In order to clarify and fix the taxonomic status and the type locality of *helvola* it is necessary to designate a neotype, in accordance with ICZN Art. 75.3. To preserve nomenclatural stability, we think it is justified to designate as neotype the specimen that has been used as reference for the taxon since Zilch (1960).

Wagner (1924) mentioned *helvola* as a species of *Alpidelima*. But in the same paper he listed it under *Albanodelima*, suggesting *Alpidelima* could have been merely a typographic error.

#### Montenegrina helvola carinata

Taxon classificationAnimaliaStylommatophoraClausiliidae

Erőss & Szekeres, 1999

Fig. 15J

Montenegrina helvola carinata Erőss & Szekeres, 1999 in Erőss et al. 1999: 449–450, fig. 4.

##### Diagnosis.

Shell small to large, slender. Lower whorls smooth to finely, indistinctly costate, upper ones wrinkled-costate. Neck strongly costate. Peripheral crest particularly strong, extends to the entire last whorl. Peristome pear-shaped. In front view lamella inferior hidden or only barely emerged. Lunella ventrolateral, fused to the long basalis. Subclaustralis short, sulcalis absent or residual. Anterior plica superior long. Clausilium plate not visible through the aperture.

##### Dimensions

(in mm). H_s_: 15.4–21.2 (holotype 18.9), W_s_: 3.4–4.2 (holotype 3.7).

##### Type locality.

Albania, Mallakastër District, 4 km SSE of Greshicë, gorge of the Përroi i Povlit (in the description erroneously given as Konakut Valley), 150 m, 40.5356°N, 19.7964°E.

##### Type material.

Type locality, leg. ZE, 17.viii.1993, holotype (HNHM 70836), paratypes (HNHM 70837/2, NHMW 100077/2, SMF 312506/2, NMBE 534907/1, NMBE 22595/1).

##### Other material.

Type locality, leg. ZF, JK, DM, 11.x.2004 (HNHM 95051); Byllis, 40.5447°N, 19.7389°E, leg. AR, NR, PR, ix.2003 (NHMW 110430/MN/0078); Poçem, bank of the Vjosë, 50 m, 40.4926°N, 19.7261°E, leg. ZB, DP, GP, 11.v.2014 (HNHM 99618).

##### Distribution.

Mallakastër Hills in southern Albania, in the lower valley of the Vjosë River. Known localities are within 5 km from each other (Fig. 18).

##### Remarks.

Nordsieck (2009) mentions this taxon from Mollas, which record presumably refers to *Montenegrina helvola ornata* Erőss & Szekeres, 1999.

#### Montenegrina helvola magna

Taxon classificationAnimaliaStylommatophoraClausiliidae

Fehér & Szekeres, 2006

Fig. 15K

Montenegrina helvola magna Fehér & Szekeres, 2006 in Erőss et al. 2006: 190, fig. 10. – Nordsieck 2009: 73.

##### Diagnosis.

Shell medium to large, ventricose. Lower whorls smooth, upper ones smooth to indistinctly wrinkled-costate. Neck wrinkled-costate. Peristome pear-shaped to somewhat angular. In front view lamella inferior barely emerged. Lunella lateral, fused to the short basalis. Subclaustralis and sulcalis residual. Anterior plica superior long, rarely connected to the lunella complex. Clausilium plate not visible through the aperture.

##### Dimensions

(in mm). H_s_: 14.5–20.6 (holotype 18.7), W_s_: 3.7–5.0 (4.2).

##### Type locality.

Albania, Mat District, Ura e Vashës, in the gorge of the Lumi i Matit, at its confluence with the Përroi i Gurri i Bardhit, 350 m, 41.4677°N, 20.1048°E.

##### Type material.

Type locality, leg. ZF, JK, DM, 9.x.2004, holotype (HNHM 94848), paratypes (HNHM 94849/26, NHMW 103278, SMF 328082).

##### Other material.

Type locality, leg. ZF, TN, EM, 15.iv.2014 (HNHM 99010); between Fshat and Shkallë, N slope of the Mt. Kjuteti, 450 m, 41.4794°N, 20.0814°E, leg. ZB, DP, B. Pintér, 27.v.2007 (HNHM 99619); 6 km S of Gurri i Bardhë along the Klos to Elbasan road, gorge of the Lumi i Guisës, 1030 m, 41.4306°N, 20.0920°E, leg. LD, ZE, ZF, AH, DM, 30.vi.2007 (HNHM 99622); Librazhd District, Orenjë, 720 m, 41.2802°N, 20.2100°E, leg. ZF, EM, 13.iv.2014 (HNHM 98962).

##### Distribution.

Gropa-Bizë-Martanesh mountain group in central Albania. The most distant sites are ca. 25 km from each other (Fig. 18).

#### Montenegrina helvola ornata

Taxon classificationAnimaliaStylommatophoraClausiliidae

Erőss & Szekeres, 1999

Fig. 15L

Montenegrina helvola ornata Erőss & Szekeres, 1999 in Erőss et al. 1999: 450–451, fig. 5. – Nordsieck 2009: 73.
Montenegrina n. sp. – Dhora and Welter-Schultes 1999: 17.
Montenegrina helvola carinata – Nordsieck 2009: 77, plate 2, figs 6–7.

##### Diagnosis.

Shell small to medium. All whorls indistinctly wrinkled-costate. Neck wrinkled-costate. Peristome pear-shaped. In front view lamella inferior barely emerged. Lunella lateral-ventrolateral, fused to the short basalis. Subclaustralis residual, sulcalis residual or absent. Anterior plica superior long. Clausilium plate not visible through the aperture.

##### Dimensions

(in mm). H_s_: 13.2–16.9 (holotype 15.7), W_s_: 3.0–3.9 (holotype 3.3).

##### Type locality.

Albania, Elbasan District, Petresh (= 5 km SSE of Graçen), along the Tiranë to Elbasan road, S of the village, 440 m, 41.1030°N, 20.0064°E.

##### Type material.

Type locality, leg. ZE, 19.viii.1993, holotype (HNHM 70838), paratypes (HNHM 70839/2, NHMW 100079/2, SMF 312507/2, NMBE 534906/1, NMBE 22594/1).

##### Other material.

Type locality, leg. ZF, TK, DM, 22.vi.2012 (HNHM 99617); “Elbasan Paß”, leg. Jaeckel (NHMW-K 57868/1, NHMW-K 47293/1); 1 km SE of Shkamë (near Mollas), W side of the Mt. Sulovë, 480 m, 40.9280°N, 20.0305°E, leg. ZE, ZF, JK, DM, 29.vi.2003 (HNHM 94904).

##### Distribution.

Krabba and Sulovë Mts in central Albania. The type locality is in the southern part of the Krraba Mountain, and presumably the site “Elbassan-paß, 800 m” given by Jaeckel and Schmidt (1961) can be in the vicinity. We found this subspecies in the Mt. Sulovë, at ca. 20 km from the type locality, and isolated from it by a wide, non-rocky flysch zone. Some other locality records in the literature from the Mt. Sulovë (Dhora and Welter-Schultes 1999, Nordsieck 2009) presumably also correspond to this taxon (Fig. 18).

#### Montenegrina helvola pageti

Taxon classificationAnimaliaStylommatophoraClausiliidae

Brandt, 1962

Fig. 15M

Montenegrina (Heteroptycha) pageti Brandt, 1962: 143–144, plate 5, fig. 14.
Montenegrina helvola pageti – Zilch 1981: 128, plate 13, fig. 24. – Nordsieck 2009: 73.

##### Diagnosis.

Shell small to medium. Lower whorls smooth, upper ones indistinctly wrinkled-costate. Neck densely costate. Basal crest strong, peripheral crest very strong. Peristome pear-shaped. In front view lamella inferior more or moderately emerged. Lunella lateral, fused to the short basalis. Subclaustralis and sulcalis residual or absent. Anterior plica superior long. Clausilium plate not visible through the aperture.

##### Dimensions

(in mm). H_s_: 13.2–16.9, W_s_: 3.4–4.2.

##### Type locality.

“Berat (Perat) in S-Albanien, am Abhang des Festungsberges” = Albania, Berat district, Berat, slope of the castle hill.

##### Type material.

“Perat, Festungberg”, ex Zilch, leg. Fuchs, 1936, holotype (SMF 167016), paratypes (SMF 167017/2, SMF 201625/2, NHMW-E 16552/20, NHMW-K 48296/5, ZMH 85772/3); “Berat, Festungsberg”, leg. Fuchs, ex Brandt, ? paratypes (type status not indicated on the label) (ZSM 20150455/3).

##### Other material.

Berat, SW side of the castle hill, 70 m, 40.7041°N, 19.9479°E, leg. DA, ZE, ZF, JG, 27.vi.2014 (HNHM 99616, NHMW 110430/MN/0077).

##### Distribution.

Known only from the castle hill of Berat (type locality), which is one of the westernmost foothills of the Tomorr Mts (Fig. 18).

#### Montenegrina hiltrudae

Taxon classificationAnimaliaStylommatophoraClausiliidae

Nordsieck, 1972

##### Diagnosis.

Shell small to large, light corneous to light brown. Lower whorls smooth to costate, sculpture stronger toward the apex. Neck variably inflexed. Basal crest weak to strong, peripheral crest weak to not recognizable. Peristome circular to ovoid, with simple to swollen margin. Lamellae superior and spiralis with no to long overlap. In front view lamella inferior more or less well emerged, broadly-bent subcolumellaris variably exposed. Lunella dorsal to dorsolateral. Basalis absent to strong, fused to or separate from the lunella. Subclaustralis absent to weak, sulcalis rudimentary to strong. Anterior plica superior present, connected to or separate from the lunella complex. Clausilium plate partly visible through the aperture.

#### Montenegrina hiltrudae hiltrudae

Taxon classificationAnimaliaStylommatophoraClausiliidae

Nordsieck, 1972

Fig. 19A

Montenegrina hiltrudae Nordsieck, 1972: 33–34, plate 4, fig. 32.
Montenegrina hiltrudae hiltrudae – Zilch 1981: 128, plate 14, fig. 35. – Nordsieck 2009: 74.

##### Diagnosis.

Shell small to medium, tumid, light corneous. Lower and upper whorls distinctly costate, ribs sharper behind the aperture. Neck weakly inflexed. Basal crest strong, peripheral one not recognizable. Peristome detached, circular to ovoid, with swollen, reflexed margin. Lamellae superior and spiralis mostly overlap. In front view lamella inferior well emerged, medium-bent subcolumellaris mostly visible. Lunella dorsal-dorsolateral, weakens toward the basis. Basalis absent or weak and separate from the lunella. Subclaustralis very short, sulcalis present. Anterior plica superior occasionally connected to the lunella complex.

##### Dimensions

(in mm). H_s_: 13.9–17.4 (holotype 15.9), W_s_: 4.0–5.0 (holotype 4.3).

**Figure 19.**
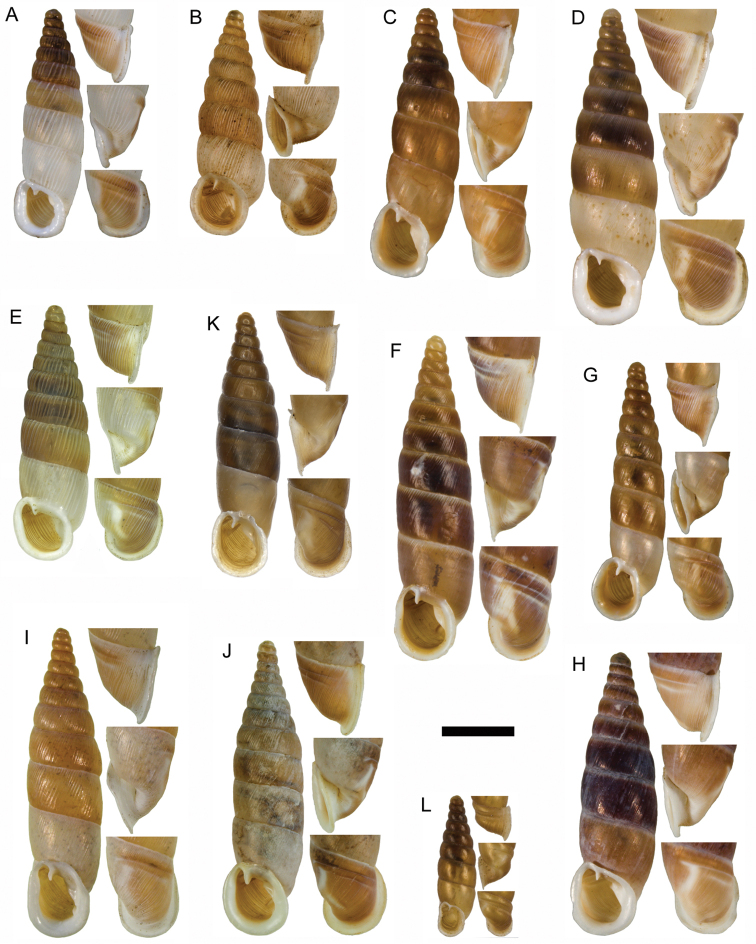
**A** *Montenegrina hiltrudae hiltrudae* Nordsieck, 1972, holotype, SMF 221046 **B** *Montenegrina hiltrudae costulata* Erőss & Szekeres, 2006, holotype, HNHM 94893 **C** *Montenegrina hiltrudae dennisi* Gittenberger, 2002, paratype, SMF 331782 **D** *Montenegrina hiltrudae densicostulata* Nordsieck, 1974, holotype, SMF 227686 **E** *Montenegrina hiltrudae desaretica* ssp. n., holotype, NHMW 111250 **F** *Montenegrina hiltrudae fusca* Fehér & Szekeres, 2006, holotype, HNHM 94895 **G** *Montenegrina hiltrudae maasseni* Gittenberger, 2002, paratype, SMF 331784 **H** *Montenegrina hiltrudae protruda* Gittenberger, 2002, paratype, SMF 331780 **I** *Montenegrina hiltrudae robusta* Nordsieck, 2009, SMF 328778 **J** *Montenegrina hiltrudae sattmanni* Nordsieck, 1988, holotype, NHMW 84028 **K** *Montenegrina hiltrudae selcensis* ssp. n., holotype, NHMW 111216 **L** *Montenegrina minuscula* Erőss & Szekeres, 2006, holotype, HNHM 94831. Scale bar: 5 mm.

##### Type locality.

Greece, Western Macedonia, near Vogatsikon, on the road to Germas (= Yermas).

##### Type material.

Type locality, leg. H. Nordsieck, 23.viii.1971, holotype (SMF 221046), paratypes (SMF 221047, SMNS-N 5476, NHMW 77935/5, NHMW-K-65052/11).

##### Distribution.

**Figure 20.**
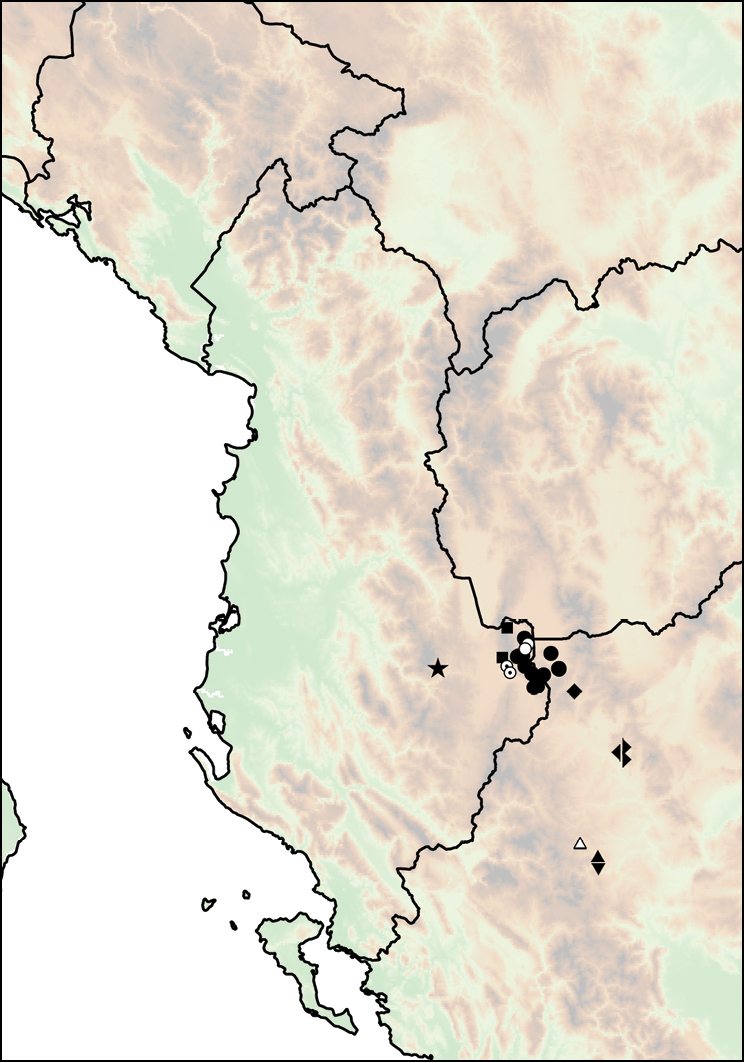
Distribution of *Montenegrina hiltrudae*. *Montenegrina hiltrudae costulata* (empty circle with dot); *Montenegrina hiltrudae dennisi* (triangle); *Montenegrina hiltrudae densicostulata* (triangle pointing right); *Montenegrina hiltrudae desaretica* ssp. n. (empty circle); *Montenegrina hiltrudae fusca* (square); *Montenegrina hiltrudae hiltrudae* (triangle pointing left); *Montenegrina hiltrudae maasseni* (inverted triangle); *Montenegrina hiltrudae protruda* (empty triangle); *Montenegrina hiltrudae robusta* (diamond); *Montenegrina hiltrudae sattmanni* (circle); *Montenegrina hiltrudae selcensis* ssp. n. (star).

Southern part of the Verno Mts in Western Macedonia (Greece). Known only from the type locality (Fig. 20).

#### Montenegrina hiltrudae costulata

Taxon classificationAnimaliaStylommatophoraClausiliidae

Erőss & Szekeres, 2006

Fig. 19B

Montenegrina sattmanni costulata Erőss & Szekeres, 2006 in Erőss et al. 2006: 204, fig. 27. – Nordsieck 2009: 73.

##### Diagnosis.

Shell small to medium, conical, whitish-corneous. Surface distinctly costate, ribs sharper on the upper whorls, denser at the neck. Neck very weakly inflexed. Basal crest strong, peripheral crest not recognizable. Peristome detached, ovoid, with somewhat swollen margin. Lamellae superior and spiralis long overlap. In front view lamella inferior more or less well emerged, broadly-bent subcolumellaris mostly not visible. Lunella dorsal, short and broad, mostly separate from the strong basalis. Subclaustralis short, sulcalis strong. Anterior plica superior mostly connected to the lunella complex.

##### Dimensions

(in mm). H_s_: 14.1–20.4 (holotype 16.0), W_s_: 3.7–5.3 (holotype 4.8).

##### Type locality.

Albania, Korçë District, Qafa e Zvezdës, 4 km from Zvezdë along the road to the Prespa Lake, 1030 m, 40.7330°N, 20.8729°E.

##### Type material.

Type locality, leg. ZE, ZF, JK, DM, 2.vii.2003, holotype (HNHM 94893), paratypes (NHMUK 20050231, HNC 63195, HNHM 94894/77, NHMW 103293, RMNH 100323, SMF 328097).

##### Other material.

Type locality, leg. ZE, ZF, JG, 29.vi.2013 (HNHM 99590); transitional forms to *Montenegrina hiltrudae sattmanni*: Korçë District, Mt. Thatë, ca. 2.7 km NNW of Zvezdë, ca. 500 m SW of the Zvezdë Summit, 1720 m, 40.7565°N, 20.8494°E, leg. ZB, CN, DP, 25.v.2007 (HNHM 99589); Zaroshka, 870 m, 40.7668°N, 20.9277°E, leg. ID, 27.v.2013 (DED).

##### Distribution.

Southern part of the Mt. Thatë, in the vicinity of the Zvezdë Pass that connents the Prespa and Korçë Basins (Fig. 20).

#### Montenegrina hiltrudae dennisi

Taxon classificationAnimaliaStylommatophoraClausiliidae

Gittenberger, 2002

Fig. 19C

Montenegrina dennisi dennisi Gittenberger, 2002: 134, figs 7–8. – Nordsieck 2009: 73, plate 2, fig. 10.

##### Diagnosis.

Shell medium, light corneous. Lower whorls smooth, upper ones moderately wrinkled-costate. Neck weakly inflexed, finely costate. Basal and peripheral crests weak. Peristome attached, ovoid, with somewhat swollen margin. Lamellae superior and spiralis mostly overlap. In front view lamella inferior well emerged, broadly-bent subcolumellaris usually not visible. Lunella dorsal, short and broad. Basalis absent or residual, separate from the lunella. Subclaustralis absent, sulcalis well developed. Anterior plica superior weak, not connected to the lunella complex.

##### Dimensions

(in mm). H_s_: 15.7–21.2 (holotype 18.7), W_s_: 4.3–5.2 mm.

##### Type locality.

Greece, Western Macedonia, 1.5 km SE of Zakas, 2.5 km along the road to Spileo, N slope, 900 m.

##### Type material.

Type locality, leg. Gittenberger, Uit de Weerd, holotype (RMNH 94939), paratypes (RMNH 94940/50); same locality, leg. Gittenberger, Maassen, 23.v.2001, paratypes (RMNH 82021/30, SMF 331782/3, NMBE 534890/3); Spileo, west side, 3.25 km S of Zakas, SSW slope, 930 m, leg. Gittenberger, Maassen, 24.v.2001, paratypes (RMNH 82027/32, SMF 331783/3, NMBE 534889/3).

##### Other material.

Greece, 0.5 km N of Spileo, toward Zakas, 930 m, 40.0080°N, 21.2847°E, leg. ZE, ZF, JG, 22.vi.2013 (HNHM 99591); 3 km E of Zakas, 900 m, 40.0422°N, 21.2823°E, leg. ZE, AH, 15.vii.2004 (HNHM 95393); 0.5 km W of Spileo, toward Portitsa Farangi, 940 m, 40.0077°N, 21.2805°E, leg. ZE, ZF, JG, 22.vi.2013 (HNHM 99592).

##### Distribution.

Western Macedonia (Greece). All known localities are within a range of a few km on the Mt. Orliakas, reaching to the Venetikos River in the the north (Fig. 20).

##### Remarks.

Originally *Montenegrina hiltrudae dennisi* was described as a separate species (Gittenberger 2002). However, its shell features (and also those of the closely related *Montenegrina hiltrudae protruda* Gittenberger, 2002) fit well in the varability range of *Montenegrina hiltrudae* subspecies.

#### Montenegrina hiltrudae densicostulata

Taxon classificationAnimaliaStylommatophoraClausiliidae

Nordsieck, 1974

Fig. 19D

Montenegrina hiltrudae densicostulata Nordsieck, 1974: 155, plate 6, fig. 37. – Zilch 1981: 128, plate 14, fig. 36. – Nordsieck 2009: 74.
Montenegrina hiltrudae (partim) – Nordsieck 1972: 33–34.

##### Diagnosis.

Shell small to medium, tumid, light corneous. Lower whorls striate to densely costate, upper ones with stronger ribs. Neck weakly inflexed. Basal crest strong, peripheral crest not recognizable. Peristome detached, circular to ovoid, with swollen, reflexed margin. Lamellae superior and spiralis mostly overlap. In front view lamella inferior well emerged, medium-bent subcolumellaris usually visible. Lunella dorsal-dorsolateral, weakens toward the basis. Basalis absent or short, separate from the lunella. Subclaustralis very short, sulcalis well developed. Anterior plica superior occasionally connected to the lunella complex.

##### Dimensions

(in mm). H_s_: 15.8–22.4 (holotype 21.9), W_s_: 4.3–6.1 (holotype 6.1).

##### Type locality.

Greece, Western Macedonia, Srianovon, along the Neapolis to Vogatsikon road.

##### Type material.

Type locality, leg. H. Nordsieck, 23.viii.1971, holotype (SMF 227686), paratypes (SMF 227687, SMNS-N 5475, NMBE 534967/2, NHMW-K-65051/10).

##### Other material.

Greece, Kastoria District, above a quarry along the Vogatsiko to Driovouno road, 680 m, 40.3782°N, 21.4160°E, leg. ZE, ZF, JG, 28.vi.2013 (HNHM 99584); 1 km W of Germas, dry gorge, 840–880 m, 40.4430°N, 21.4111°E, leg. ZE, ZF, JG, 28.vi.2013 (HNHM 99582, HNHM 99583).

##### Distribution.

Southern part of the Verno Mountain in Western Macedonia (Greece). Found at some sites around Vogatsiko, southeast of the Kastoria Lake (Fig. 20).

##### Remarks.

Originally Nordsieck (1972) classified *Montenegrina hiltrudae densicostulata* with the nominotypical subspecies, but only two years later he described it as a distinct taxon (Nordsieck 1974).

#### Montenegrina hiltrudae desaretica ssp. n.

Taxon classificationAnimaliaStylommatophoraClausiliidae

http://zoobank.org/BF6871BD-EC85-4947-8847-8A7ABD1B53BD

Fig. 19E

Montenegrina sattmanni sattmanni – Erőss et al. 2006: 206.

##### Diagnosis.

Medium-size subspecies with costate shell, attached peristome, and non-overlapping lamellae superior and spiralis.

##### Description.

The light greyish-corneous shell of 8½ to 10½ whorls is tumid, with relatively large aperture. All whorls are more or less costate, more distinctly toward the apex. The neck is not or only very weakly inflexed, its ribs become sharper and near the aperture. The basal crest is weak, the peripheral one is not recognizable. The aperture is almost circular, its broadly attached margin is somewhat swollen and deflexed. The lamella superior is low but long, it does not overlap with the spiralis. In front view the lamella inferior is moderately emerged, the end of the broadly-bent subcolumellaris is mostly visible. The short and broad lunella is dorsal-dorsolateral, it is connected to the often weak basalis. The subclaustralis is absent, the sulcalis is rudimentary. The anterior part of the plica superior is weak, not connected to the lunella complex. The clausilium plate is partly visible through the aperture.

##### Dimensions

(in mm). Holotype H_s_: 17.6, W_s_: 5.5, H_a_: 4.5, W_a_: 4.1; paratypes H_s_: 15.0–18.9 (mean 16.9, S.D. 1.33), W_s_: 4.4–5.9 (mean 5.3, S.D. 0.39), H_a_: 3.8–5.2, W_a_: 3.3–4.3.

##### Differential diagnosis.

The new subspecies resembles the nearby occurring *Montenegrina hiltrudae sattmanni*, but can be distinguished from it by the costate shell, broadly attached peristome, non-overlapping lamellae superior and spiralis, as well as the reduced subclaustralis, sulcalis and anterior plica superior. From all other *Montenegrina hiltrudae* subspecies differs by the combination of an attached peristome and costate lower whorls.

##### Type locality.

Albania, Korçë District, cave temple ca. 2 km S of Glloboçeni, at the Prespa Lake, 850 m, 40.8422°N, 20.9616°E.

##### Type material.

Type locality, leg. ZE, ZF, JG, 30.vi.2015, holotype (NHMW 111250), paratypes (NHMW 111251/20+5a+11aj, HNHM 99690/25, GR/25, SZ/5); same locality, leg ID, 23.ix.2013, paratypes (DED/17).

##### Other material.

Albania, Korçë District, 1 km NE of Liqenas, Sveti Atanas i Veliki Antoni Church, 850 m, 40.7919°N, 20.9152°E, leg. ZE, ZF, JG, 28.vi.2013 (HNHM 99587); same locality, leg. ZE, ZF, JK, DM, 2.vii.2003 (HNHM 94901); Liqenas, 860 m, 40.7891°N, 20.9072°E, leg. ZE, ZF, JK, DM, 2.vii.2003 (HNHM 94898); Ishull i Vogël, E of Liqenas, 860 m, 40.7915°N, 20.9324°E, leg. ZF, LT, 17.viii.2007 (HNHM 99572).

##### Etymology.

The new taxon is named after Desaretia, the ancient Roman name of the Ohrid–Prespa region.

##### Distribution.

Prespa Lake area. Apart from the type locality, it was also found in the vicinity of Liqenas (Fig. 20).

##### Remarks.

Formerly the Liqenas population was mentioned by Erőss et al. (2006) as differing from the typical *Montenegrina hiltrudae sattmanni* by the stronger apical sculpture. However, more recent morphological and distribution data suggest that the populations around Liqenas belong to *Montenegrina hiltrudae desaretica*, but differ from its typical form by the weaker sculpture and the occasionally connected anterior plica superior.

#### Montenegrina hiltrudae fusca

Taxon classificationAnimaliaStylommatophoraClausiliidae

Fehér & Szekeres, 2006

Fig. 19F

Montenegrina sattmanni fusca Fehér & Szekeres, 2006 in Erőss et al. 2006: 204–206, fig. 28.
Montenegrina skipetarica fusca – Nordsieck 2009: 73.

##### Diagnosis.

Shell medium, light brown, paler along the suture. Lower whorls smooth, upper ones indistinctly striate-costate. Neck weakly inflexed, densely striate behind the aperture. Basal crest weak, peripheral crest not recognizable. Peristome attached, ovoid, with somewhat swollen margin. Lamellae superior and spiralis overlap. In front view lamella inferior well emerged, broadly-bent subcolumellaris not visible. Lunella dorsal-dorsolateral, short and broad, mostly fused to the basalis. Subclaustralis short, sulcalis strong. Anterior plica superior connected to the lunella complex, forward reaches as far as the plica principalis.

##### Dimensions

(in mm). H_s_: 18.3–22.4 (holotype 22.4), W_s_: 4.5–5.3 (holotype 5.3).

##### Type locality.

Albania, Korçë District, Korit e Bregas, 6 km S of Podgorje along the Pogradec to Zvezdë road, 900 m, 40.7688°N, 20.8275°E.

##### Type material.

Type locality, leg. ZE, ZF, JK, DM, 2.vii.2003, holotype (HNHM 94895), paratypes (HNHM 94896/9, NHMW 103294, RMNH 100324, SMF 328098).

##### Other material.

Type locality, leg. ZE, ZF, JG, 29.vi.2013 (HNHM 99596); Veliterna, leg. Megyeri, 7.vii.1960 (HNHM 99597); Mt. Thatë, near the ‘Tower of the war’, 1830 m, 40.9136°N, 20.8419°E, leg. ID, 24.ix.2013 (DED).

##### Distribution.

Mt. Thatë in southern Albania. The type locality is at the western foot of the mountain, and this taxon was also found in the alpine region at the ‘Tower of the war’, near the Albanian–Macedonian border (Dedov, unpublished data). At this locality it occurs syntopically with *Montenegrina dofleini wagneri* (Fig. 20).

#### Montenegrina hiltrudae maasseni

Taxon classificationAnimaliaStylommatophoraClausiliidae

Gittenberger, 2002

Fig. 19G

Montenegrina janinensis maasseni Gittenberger, 2002: 131–134, figs 5–6. – Nordsieck 2009: 75.
Montenegrina janinensis maasii (sic! typographic error) – Uit de Weerd and Gittenberger 2004: 307, fig. 1d.

##### Diagnosis.

Shell small to medium, light corneous. Lower whorls smooth, upper ones indistinctly wrinkled-costate. Neck inflexed, costate. Basal and peripheral crests weak. Peristome attached, ovoid, with simple margin. Lamellae superior and spiralis mostly overlap. In front view lamella inferior more or less well emerged, broadly-bent subcolumellaris occasionally visible. Lunella dorsal, short and broad. Basalis residual, separate from the lunella. Subclaustralis absent, sulcalis well developed. Anterior plica superior mostly not connected to the lunella complex.

##### Dimensions

(in mm). H_s_: 14.5–18.6 (holotype 16.0), W_s_: 3.6–4.1.

##### Type locality.

Greece, Western Macedonia, Portitsa bridge, 4 km SE of Zakas, 625 m.

##### Type material.

Type locality, leg. Gittenberger, Maassen, 24.v.2001, holotype (RMNH 94949), paratypes (RMNH 82024/28, NMBE 535056/2, SMF 331784/2).

##### Other material.

Greece, Portitsa Farangi, near Spileo, 39.9965°N, 21.2855°E, leg. ZE, ZF, JG, 22.vi.2013 (HNHM 99595); same locality, leg. ZE, AH, 15.vii.2004 (HNHM 99594).

##### Distribution.

Western Macedonia (Greece). Known only from its type locality, the gorge of the Venetikos River. *Montenegrina hiltrudae dennisi* occurs only a few few km farther north, at the slope of the Mt. Orliakas, bordered by the Venetikos Valley (Fig. 20).

##### Remarks.

*Montenegrina hiltrudae maasseni* was described by Gittenberger (2002) as a subspecies of *Montenegrina janinenis*, but its shell morphology suggests a close relationship to *Montenegrina hiltrudae dennisi* (see: Erőss et al. 2006).

#### Montenegrina hiltrudae protruda

Taxon classificationAnimaliaStylommatophoraClausiliidae

Gittenberger, 2002

Fig. 19H

Montenegrina dennisi protruda Gittenberger, 2002: 135, figs 9–10, 16 (clausilium plate), 17–19 (clausilium plate microarmature). – Nordsieck 2009: 73.

##### Diagnosis.

Shell medium, light to brownish-corneous. Lower whorls smooth, upper ones finely striate. Neck very weakly inflexed, finely costate. Basal and peripheral crests weak. Peristome detached, ovoid, with somewhat swollen margin. Lamellae superior and spiralis mostly overlap. In front view lamella inferior well emerged, broadly-bent subcolumellaris not visible. Lunella dorsal, short and broad, not fused to the basalis. Subclaustralis not recognizable. Sulcalis well developed. Anterior plica superior weak, not connected to the lunella complex. Lunella dorsal-dorsolateral to dorsolateral, short and broad, mostly fused to the basalis. Subclaustralis weak or absent, sulcalis well developed. Anterior plica superior not connected to the lunella complex.

##### Dimensions

(in mm). H_s_: 16.0–20.6 (holotype 19.0), W_s_: 4.8–5.4 mm.

##### Type locality.

Greece, Western Macedonia, 2.3 km before Aetia along the Aetia to Anavrita road, along the path to the Nymphoon Cave, W slope, 1000 m.

##### Type material.

Type locality, leg. Gittenberger, Uit de Weerd, holotype (RMNH 94942), paratypes (RMNH 94941/21); same locality, leg. Gittenberger, Maassen, 23.v.2001, paratypes (RMNH 82016/19, SMF 331780/3, NMBE 534891/3); hill 1.75 km E of Aetia, E slope, 940 m, paratypes (RMNH 82017/7).

##### Other material.

Type locality, 990 m, 40.0741°N, 21.2017°E, leg. ZE, ZF, JG, 22.vi.2013 (HNHM 99593).

##### Distribution.

Western Macedonia (Greece). Known only from the type locality (Fig. 20).

#### Montenegrina hiltrudae robusta

Taxon classificationAnimaliaStylommatophoraClausiliidae

Nordsieck, 2009

Fig. 19I

Montenegrina skipetarica robusta Nordsieck, 2009: 77–78, plate 2, fig. 8.

##### Diagnosis.

Shell medium to large, brownish-corneous. Very weak and indistinct ribs of the lower whorls become stronger and better defined toward the apex. Weakly inflexed neck has more robust ribs. Basal and peripheral crests weak. Peristome ovoid, mostly attached, with swollen, reflexed margin. Lamellae superior and spiralis overlap. In front view lamella inferior more or less well emerged, broadly-bent subcolumellaris visible. Lunella short and broad, dorsal-dorsolateral to dorsolateral, mostly fused to the basalis. Subclaustralis residual or absent, sulcalis well developed. Anterior plica superior long, often connected to the lunella complex.

##### Dimensions

(in mm). H_s_: 18.0–25.1 (holotype 21.4), W_s_: 4.55–5.8 (holotype 5.2).

##### Type locality.

Greece, Western Macedonia, 4 km from Gavros toward Kotas along the Kastoria to Florina road, 850 m.

##### Type material.

Type locality, leg. Hemmen, 13.iv.1995, holotype (SMF 328778), paratypes (SMNS-N 10260/5).

##### Other material.

Same as types (NHMW 110430/MN/0117); type locality, 40.6532°N, 21.1789°E, leg. ZF, EH, KJ, HS, 18.x.2014 (NHMW 110430/MN/0118).

##### Distribution.

Western part of the Verno Mts in Western Macedonia (Greece). Known only from the type locality, located between Kastoria and the Prespa Lakes (Fig. 20).

##### Remarks.

Some specimens of the original series (e.g. those of NHMW 110430/MN/0117) were not designated as types.

#### Montenegrina hiltrudae sattmanni

Taxon classificationAnimaliaStylommatophoraClausiliidae

Nordsieck, 1988

Fig. 19J

Montenegrina sattmanni Nordsieck, 1988: 200–201, fig. 5.
Montenegrina sattmanni sattmanni – Nordsieck 2009: 73, plate 2, fig. 9.

##### Diagnosis.

Shell medium, light corneous. Lower whorls smooth, upper ones finely striate to indistinctly costate. Neck not or very weakly inflexed, densely striate. Basal crest very weak, peripheral crest not recognizable. Peristome attached, ovoid, with somewhat swollen margin. Lamellae superior and spiralis overlap. In front view lamella inferior well emerged, broadly-bent subcolumellaris mostly visible. Lunella dorsal-dorsolateral, short and broad, separate from the basalis. Subclaustralis short, sulcalis strong. Anterior plica superior long, mostly connected to the lunella complex.

##### Dimensions

(in mm). H_s_: 17.3–21.4 (holotype 20.1), W_s_: 4.6–5.5 (holotype 5.3).

##### Type locality.

Greece, Western Macedonia, Mikrolimni, near the Biological Station.

##### Type material.

Type locality, leg. HS, 24.v.1986, holotype (NHMW 84028), paratypes, (NHMW 84029/7, SMNS-N 9520/2).

##### Other material.

Type locality, 40.7429°N, 21.1102°E, leg. ZE, ZF, JG, 28.vi.2013 (HNHM 99585); 500 m N of the Agios Achillios junction (Psarades to Vrondero road), 870 m, 40.7998°N, 21.0715°E, leg. ZF, EH, KJ, HS, 18.x.2014 (NHMW 110430/MN/0065); 3 km SE of Vrondero, Petros Kokkalis Cave, 1000 m, 40.7192°N, 21.0341°E, leg. ZF, EH, KJ, HS, 18.x.2014 (NHMW 110430/MN/0066); Albania, Korçë District, Tren, Shpella e Trenit, bank of the Small Prespa Lake, 860 m, 40.6723°N, 20.9871°E, leg. ZE, ZF, JG, 28.vi.2013 (HNHM 99586); Mt. Thatë, ca. 1.7 km N of Zvezdë, 2.1 km southeast of the Zvezdë Summit, 1180 m, 40.7419°N, 20.8635°E, leg. ZB, CN, DP, 25.v.2007 (HNHM 99588); Zagradets, 870 m, 40.6767°N, 21.0039°E, leg. ID, 24.vi.2013 (DED); Cerie, 1130 m, 40.7523°N, 20.9434°E, leg. ID, 24.vi.2013 (DED); Rakitsko, 1100 m, 40.7245°N, 20.9768°E, leg. ID, 26.v.2013 (DED); Glloboçeni, S side, boat harbor, 850 m, 40.8584°N, 20.9474°E, leg. ZE, ZF, JG, 30.vi.2013 (NHMW 110430/MN/0067); N of Glloboçeni, 860 m, 40.8580°N, 20.9426°E, leg. ZF, EH, KJ, HS, 17.x.2014 (NHMW 110430/MN/0064);

##### Distribution.

The area of the Prespa Lakes (Fig. 20).

##### Remarks.

Originally *Montenegrina hiltrudae sattmanni* was described as a distinct species (Nordsieck 1988), but its shell characteristics and distribution range indicate its belonging to *Montenegrina hiltrudae*.

The paratype lot NHMW 84030/4 could not be found in the NHMW collection.

#### Montenegrina hiltrudae selcensis ssp. n.

Taxon classificationAnimaliaStylommatophoraClausiliidae

http://zoobank.org/726612D6-C5CD-4049-A9F3-3A115643F617

Fig. 19K

##### Diagnosis.

Medium-size subspecies with large, very narrowly attached peristome and in front view well visible lamella subcolumellaris.

##### Description.

The medium-size, light brownish-corneous shell of 9 to 10½ whorls is tumid, with brad basis. The surface is smooth at the lower whorls but becomes indistinctly and densely striate-costate toward the apex. The neck is only very weakly inflexed, behind the peristome it is finely and densely striate. The basal crest is weak, the peripheral crest is not recognizable. The aperture is very large, with the peristome its height reaches one quarter of that of the entire shell. The light whitish-brown peristome is wide, narrowly attached, its margin is swollen, deflexed. The lamellae superior and spiralis overlap. The lamella inferior is strongly emerged, its inner part bends close to the spiralis. The broadly-bent lamella subcolumellaris is visible in front view. The lunella is dorsal-dorsolateral, short and broad, mostly separate from the basalis. The subclaustralis short, the sulcalis is well developed. The anterior part of the plica superior is long, ends in a small lump behind the aperture. Its inner end occasionally extends to the lunella complex. The clausilium plate is entirely visible through the aperture.

##### Dimensions

(in mm). Holotype H_s_: 19.9, W_s_: 6.2, H_a_: 5.5, W_a_: 4.4; paratypes H_s_: 16.5–21.4 (mean 19.4, S.D. 1.40), W_s_: 5.1–6.2 (mean 5.7, S.D. 0.39), H_a_: 4.6–5.8, W_a_: 4.0–4.9 (NHMW 111217, NHMW 111218, HNHM 99481, HNHM 99482, n = 12).

##### Differential diagnosis.

The new subspecies can be distinguished from *Montenegrina hiltrudae dennisi* and *Montenegrina hiltrudae maasseni* by its much finer neck sculpture, from *Montenegrina hiltrudae fusca* by its more conical shell and stronger lamella inferior, from *Montenegrina hiltrudae sattmanni* by the finer apical and neck sculpture, weaker basalis and more dorsal lunella, whereas from the rest of *Montenegrina hiltrudae* subspecies by the attached peristome.

##### Type locality.

Albania, Korçë District, NE of Strelcë, in the limestone gorge of the Lumi i Verbës at the foot of the Shkëmb i Selcës, 990 m, 40.7480°N, 20.5219°E.

##### Type material.

Type locality, leg. ZF, JG, 30.vi.2014, holotype (NHMW 111216), paratypes (NHMW 111218/1+2a, HNHM 99482/3, GR/2); between Strelcë and Selcë, 1090 m, 40.7462°N, 20.5123°E, leg. DA, ZE, 30.vi.2014, paratypes (NHMW 111217/19+2a, HNHM 99481/20, GR/20, SZ/5).

##### Etymology.

The new taxon is named after the Selcë Cliff (Shkëmb i Selcës), its type locality.

##### Distribution.

Southern part of the Mokër Mts. Two nearby localities are known in the vicinity of Strelcë. At the type locality this taxon is found syntopically with *Montenegrina laxa errans* Erőss & Szekeres, 2006 (Fig. 20).

#### Montenegrina janinensis

Taxon classificationAnimaliaStylommatophoraClausiliidae

(Mousson, 1859)

Fig. 21J, K

Clausilia janinensis Mousson, 1859: 276.
Clausilia (Delima) janinensis – Westerlund 1884: 54–55.
Delima (Albanodelima) janinensis – Wagner 1924: 120.
Montenegrina janinensis janinensis – Zilch 1981: 129. – Nordsieck 2009: 75.
Montenegrina janinensis crassilabris Fauer, 1993: 55–56, plate 1, fig. 7. – Nordsieck 2009: 75.

##### Diagnosis.

Shell small, light corneous. Lower whorls smooth, upper ones with fine, widely-spaced, indistinct ribs. Neck inflexed, wrinkled-costate. Basal and peripheral crests well visible. Peristome attached, rounded to somewhat angular, with slightly to strongly swollen, reflexed margin. Lamellae superior and spiralis overlap. In front view lamella inferior moderately emerged, broadly-bent subcolumellaris visible. Lunella dorsolateral, mostly separate from the weak basalis. Subclaustralis residual, sulcalis well developed. Anterior plica superior not connected to the lunella complex. Clausilium plate partly visible through the aperture.

##### Dimensions

(in mm). H_s_: 12.2–17.1, W_s_: 3.2–4.2 (Perama, HNHM 99560).

**Figure 21.**
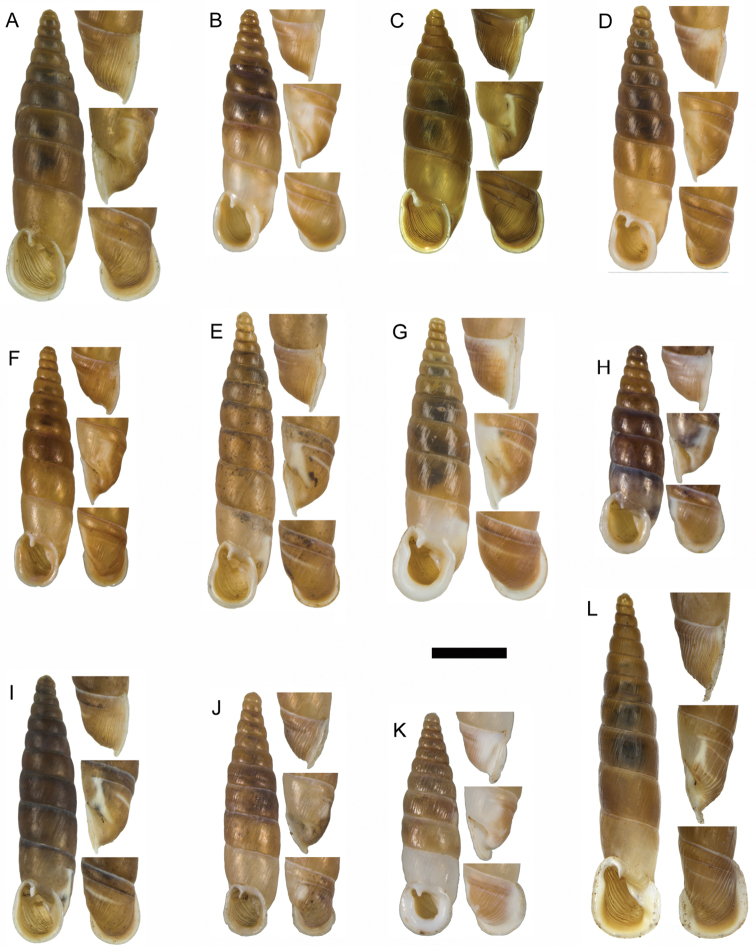
**A** *Montenegrina laxa laxa* (Küster, 1861), Tërkuza Valley HNHM 99033 **B** *Montenegrina laxa dedovi* Nordsieck, 2009, holotype, SMF 332465 **C** *Montenegrina laxa delii* ssp. n., holotype, NHMW 111237 **D** *Montenegrina laxa disjuncta* Fehér & Szekeres, 2006, holotype, HNHM 94871 **E** *Montenegrina laxa errans* Erőss & Szekeres, 2006, holotype, HNHM 94874 **F** *Montenegrina laxa iba* Nordsieck, 1972, holotype, SMF 201629a **G** *Montenegrina laxa kontschani* Erőss & Szekeres, 2006, holotype, HNHM 94878 **H** *Montenegrina laxa lakmosensis* Nordsieck, 2009, paratype, SMF 332354 **I** *Montenegrina laxa miraka* Nordsieck, 1996, Mirakë, HNHM 99630 **J** *Montenegrina janinensis* (Mousson, 1859), syntype, SMF 176316 **K** *Montenegrina janinensis* (Mousson, 1859), holotype of *crassilabris*, SMF 309237 **L** *Montenegrina lillae* sp. n., holotype, HNHM 99496. Scale bar: 5 mm.

##### Type locality.

“environs de Ianina” = Greece, Epirus, Ioannina [*janinensis*]; Greece, Epirus, Kria near Perama, above the southeastern cemetery, 600 m [*crassilabris*].

##### Type material.

“Janina, Epirus”, ex Schaefli, ex Mousson, syntypes [*janinensis*] (SMF 176316/6); same locality, ex Boettger, syntypes [*janinensis*] (SMF 176317/3); same locality, ex Shuttleworth, syntypes [*janinensis*] (NMBE 21041/6); Kria near Perama, leg. Fauer, 10.viii.1971, holotype [*crassilabris*] (SMF 309237), paratypes [*crassilabris*] (SMF 309238/3, HNHM 39968/3, SMNS-N 9791, ZMH 91624/pl.); same locality, leg. PS, Fauer, 27.ix.1989, paratypes [*crassilabris*] (NMBE 535019, NMBE 22589/3, NHMW 103731, ZMH 91623/pl.); Kria, above the church, leg. Hausdorf, 31.v.1985, paratypes [*crassilabris*] (ZMH 91622/pl.); Kria, above the southeastern cemetery, 600 m, leg. Fauer, 22.iv.1988, paratypes [*crassilabris*] (ZMH 91627/pl.); 1.2 km from Kria toward Kranoula, ex Fauer, 10.viii.1971, paratypes [*crassilabris*] (ZMH 91625/6); Perama, exit of the stalactite cave, leg. PS, 29.vii.1975 paratypes [*crassilabris*] (HNHM 67025/4, NMBE 535017/60, ZMH 91626/9).

##### Other material.

Greece, Epirus, Amfithea–Spothi road N of Lake Pamvotis, 2 km E of Ligkiades junction, 640 m, 39.6802°N, 20.9052°E, leg. ZE, ZF, JG, 24.vi.2013 (HNHM 99559); Perama, near entrance of the cave, 500 m, 39.6947°N, 20.8463°E, leg. ZE, ZF, JG, 24.vi.2013 (HNHM 99560); Mitsikeli Mts, S of Dikórifo, 1070 m, 39.7828°N, 20.8041°E, leg. PS, 23.v.1991 (NMBE); Kria, dry gorge above the cemetery, 660 m, 39.7245°N, 20.8429°E, leg. ZE, ZF, JG, 24.vi.2013 (HNHM 99562); Perama, near the exit of the stalactite cave, 520 m, 39.6971°N, 20.8433°E, leg. ZE, ZF, JG, 24.vi.2013 (HNHM 99561).

##### Distribution.

**Figure 22.**
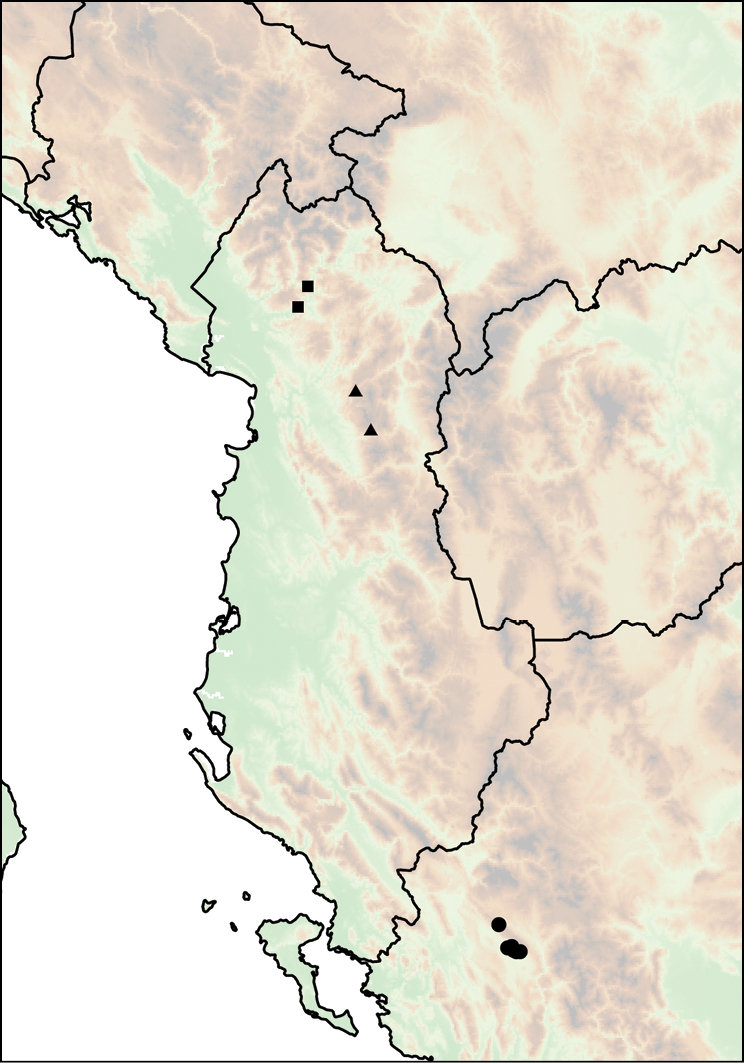
Distribution of *Montenegrina janinensis*; *Montenegrina lillae* sp. n. and *Montenegrina minuscula*. *Montenegrina janinensis* (circle); *Montenegrina lillae* sp. n. (square); *Montenegrina minuscula* (triangle).

Southwestern part and foothills of the Mitsikeli Mts in northwestern Greece. Most of the known occurrences are along the northern shore of the Pamvotis Lake (between Kria and Spothi), but a record from Dikórifo indicates that the species may be wider distributed (Fig. 22).

##### Remarks.

Although not synonymized formally, Nordsieck (2009) already questioned the distinct taxonomic status of *crassilabris*, assuming that it might only be a local form of *Montenegrina janinensis* with thickened peristome. This morphotype is known from Kria and Perama, but its range is not clearly distinct from that of the typical *janinensis*. Moreover, on the Goritsa Hill at Perama they live in parapatry: at the entrance of the stalactite cave typical *janinensis*, whereas at the exit (only a few hundred meters farther) the form with the thickened peristome (Fauer 1993, and also our personal observation).

Based mainly on their small shell size, originally several non-related *Montenegrina* taxa were described as subspecies of *Montenegrina janinensis*. These include *Montenegrina soosi* Erőss & Szekeres, 2006, and some subspecies currently classified with *Montenegrina attemsi*, *Montenegrina dofleini*, *Montenegrina grammica*, *Montenegrina hiltrudae*, *Montenegrina sporadica* Nordsieck, 1974, and *Montenegrina tomorosi* Brandt, 1961.

#### Montenegrina laxa

Taxon classificationAnimaliaStylommatophoraClausiliidae

(Küster, 1861)

##### Diagnosis.

Shell small to large, yellowish- to brownish-corneous. Lower whorls smooth, upper ones smooth to finely wrinkled-costate. Neck variably inflexed, finely striate to wrinkled-costate. Basal and peripheral crests recognizable. Peristome attached, ovoid to angular, with simple to swollen and reflexed margin. Lamellae superior and spiralis distant to long overlapping. In front view lamella inferior mostly well emerged. Weakly-bent subcolumellaris and the clausilium plate not or only barely visible through the aperture. Lunella short, broad and diffuse, dorsolateral to ventrolateral. Basalis is fused to the lunella as its almost straight continuation. Lunella complex also fused to the plica principalis. Subclaustralis short, sulcalis mostly residual or absent. Anterior plica superior usually long, distant and diverging from the principalis, in most subspecies not connected to the lunella complex.

#### Montenegrina laxa laxa

Taxon classificationAnimaliaStylommatophoraClausiliidae

(Küster, 1861)

Fig. 21A

Clausilia laxa Küster, 1861 in Küster 1844–1862: 276, plate 31, figs 14–16. – Schmidt 1868: 68–69.
Delima (Albanodelima) weigneri Poliński, 1924: 143–145, plate 4, figs 7–8. – Wagner 1924: 119.
Clausilia (Delima) laxa – Westerlund 1884: 54.
Montenegrina laxa laxa – Nordsieck 1972: 29–30, plate 5, fig. 40. – Zilch 1981: 130. – Nordsieck 2009: 73.

##### Diagnosis.

Shell medium to large, yellowish-corneous. All whorls smooth. Neck weakly inflexed, wrinkled-costate. Basal and peripheral crests weak. Peristome ovoid to somewhat angular, with simple margin. Lamellae superior and spiralis mostly do not overlap. Subcolumellar lamella barely, clausilium plate not visible through the aperture. Lunella lateral. Subclaustralis shorter than the basalis, sulcalis residual or absent. Anterior plica superior long, almost parallel to the plica principalis, not connected to the lunella complex.

##### Dimensions

(in mm). H_s_: 16.3–21.8, W_s_: 4.6–5.9 mm (Nordsieck 1972).

##### Type locality.

“wahrscheinlich aus der Gegend von Castel nuovo” = Montenegro, probably from the area of Herceg-Novi [*laxa*]; “monts Mali Dajtit à l’Est de la ville de Tirana, près de la route de Tuffina à Safa Muzizes” = Albania, Mt. Dajti east of Tiranë, route from Tufinë to the Qafa e Murrizës [*weigneri*]; “la vallée de la rivière Terküza” = Albania, N of Tiranë, gorge of the Lumi i Tërkuzës [*weigneri*].

##### Type material.

Syntypes of *weigneri* are thought to be in the MIZPAS (not seen).

##### Other material.

Albania, Tirana, “Römerbrücke” (= Roman bridge), leg. Fuchs (SMF 94109a [Nordsieck 1972: fig. 40], NHMW-E 32318, NHMW-K 54402, NHMW-K 50578, SMF 94109); “Mal i Krujë” (likely erroneous locality record), leg. Fuchs (NHMW-E 32479, NHMW-K 50575); Tiranë District, NE of Herraj, gorge of the Lumi i Tërkuzës near the dam of the Lumi i Bovillës, 330 m, 41.4446°N, 19.8661°E, leg. ZF, TN, EM, 16.iv.2014 (HNHM 99032); gorge of the Lumi i Tërkuzës 0.5 km beneath the dam, 240 m, 41.4412°N, 19.8636°E, leg. ZF, TN, EM, 16.iv.2014 (HNHM 99033); gorge of the Lumi i Tiranës, 5 km NE of Ferraj, 290 m, 41.3948°N, 19.9000°E, leg. ZF, TN, EM, 16.iv.2014 (HNHM 99035); Shkalla e Tiranit, gorge of the Lumi i Tiranës, 400 m, 41.4022°N, 19.9012°E, leg. ZE, ZF, KK, 10.iv.2001 (HNHM 86044); between Ferraj and Shtish (NE of Tiranë), at the bridge of the Lumi i Tiranës, 200 m, 41.3798°N, 19.8592°E, leg. ZE, ZF, KK, 10.iv.2001 (HNHM 86043).

##### Distribution.

**Figure 23.**
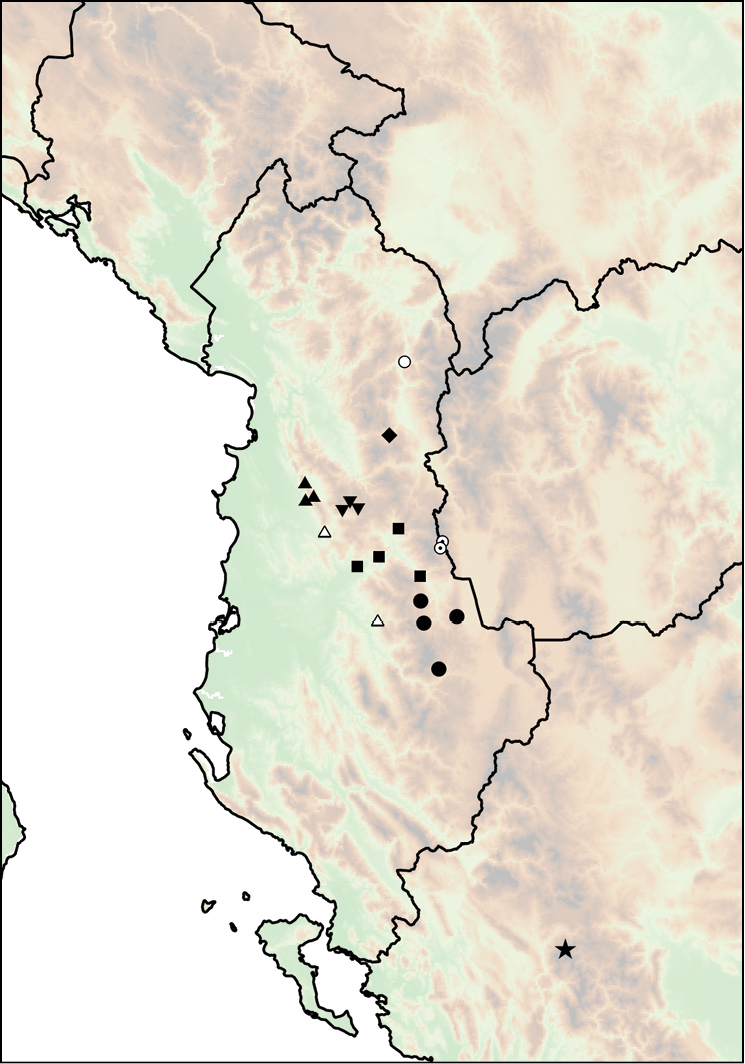
Distribution of *Montenegrina laxa*. *Montenegrina laxa dedovi* (empty circle with dot); *Montenegrina laxa delii* ssp. n. (empty circle); *Montenegrina laxa disjuncta* (diamond); *Montenegrina laxa errans* (circle); *Montenegrina laxa iba* (empty triangle); *Montenegrina laxa kontschani* (inverted triangle); *Montenegrina laxa lakmosensis* (star); *Montenegrina laxa laxa* (triangle); *Montenegrina laxa miraka* (square).

Dajti-Kruja mountain range in central Albania. Known from the gorges of the Tiranë and Terkuzë Rivers, north of Tiranë (Fig. 23).

##### Remarks.

The illustration with the name *laxa* was published in 1860, but a description of the species only in 1861 (both in Küster 1844–1862). Therefore the latter is considered the year of description (Welter-Schultes 1999).

*Montenegrina* species do not occur in the area of Herceg-Novi, which was given by Küster (1844–1862) as the type locality. This record is either incorrect or based on marine flotsam (Nordsieck 1972). Without type material and reliable type locality this taxon could be identified using the precise description of Schmidt (1868), which was based on the type series. In the view of Wagner (1924), *laxa* is a synonym of *wohlberedti* and not identical with *weigneri*. By contrast, Nordsieck (1972) claims that the Terküza Valley population fits well Schmidt’s description, therefore he considers *laxa* a senior synonym of *weigneri*. The latter view has been widely accepted in the past 40 years. In order to preserve nomenclatural stability, we stick to this practice.

#### Montenegrina laxa dedovi

Taxon classificationAnimaliaStylommatophoraClausiliidae

Nordsieck, 2009

Fig. 21B

Montenegrina dedovi dedovi Nordsieck, 2009: 75–77, plate 2, fig. 4. – Dedov and Neubert 2009: 92, plate 1, fig. 5.

##### Diagnosis.

Shell small to medium, light corneous. All whorls smooth. Neck weakly inflexed, finely and densely striate. Basal and peripheral crests weak. Peristome, ovoid to somewhat angular, with simple margin. Lamellae superior and spiralis do not overlap. Subcolumellar lamella not or barely, clausilium plate not at all visible through the aperture. Lunella lateral. Subclaustralis short to residual, sulcalis very weak or absent. Anterior plica superior distant and diverging from the plica principalis, not connected to the lunella complex.

##### Dimensions

(in mm). H_s_: 13.6–20.3 (holotype 16.1), W_s_: 3.7–4.4 (holotype 4.15) (Nordsieck 2009, Dedov and Neubert 2009).

##### Type locality.

Macedonia, Jablanica Mts, Vartop region, after the Čuma Summit, 1868 m.

##### Type material.

Type locality, leg. ID, 15.vii.2006, holotype (SMF 332465), paratypes (SMF 331838/4).

##### Other material.

Macedonia, Jablanica Mts, up to Gorna Belitsa, alpine limestone meadow, ca. 1600–1700 m, 41.22°N, 20.54°E, leg. ID, Minkov, 10.vii.2009 (DED-580); near the Kokal Summit, 1800–1928 m, 41.203°N, 20.532°E, leg. ID, 15.vii.2009 (DED-560); Tchumata area (up to the Krustets area), alpine limestone meadows, 1980 m, leg. ID, 12.vii.2009 (DED-582).

##### Distribution.

This taxon is known from the alpine region of the Jablanica Mts (Fig. 23).

##### Remarks.

Originally *Montenegrina laxa dedovi* was described as a separate species (Nordsieck 2008), but its shell structure (and particularly its palatal plicae) positions it among the subspecies of *Montenegrina laxa*.

#### Montenegrina laxa delii ssp. n.

Taxon classificationAnimaliaStylommatophoraClausiliidae

http://zoobank.org/B122E609-F6CB-4690-81B3-E1BE35FB0827

Fig. 21C

##### Diagnosis.

Large subspecies with tumid shell, long overlapping lamellae superior and spiralis.

##### Description.

The large, tumid, brownish-corneous shell consists of 8½ to 10½ whorls. The surface is smooth at the lower whorls, whereas toward the apex it becomes indistinctly stiate-costate. The neck is also indistinctly costate with riblets that become crowded behind the aperture. The basal and peripheral crests are well recognizable. The light brownish, wide ovoid peristome is broadly attached, with simple or only slightly swollen margin. The lamellae superior and spiralis long overlap. The lamella inferior is well emerged. The weakly-bent subcolumellaris is retracted, its end is barely visible in slanted view through the aperture. The lateral lunella is short and broad, with diffuse outline. It is fused, through the plica superior, to the plica principalis, and downward in an almost straight line also to the long basalis. In most cases the subclaustralis is short and weak, the reduced sulcalis is fused to the lunella complex. The anterior plica superior of variable strength starts diverging from, then becomes parallel to, the plica principalis. It is not connected to the lunella complex. The clausilium plate is not visible through the aperture.

##### Dimensions

(in mm). Holotype H_s_: 16.7, W_s_: 4.9, H_a_: 4.4, W_a_: 3.7; paratypes (NHMW 111235, n = 12): H_s_: 16.1–20.7 (mean 18.0, S.D. 1.31), W_s_: 4.9–6.0 (mean 5.3, S.D. 0.39), H_a_: 4.4–5.5, W_a_: 3.6–4.6.

##### Differential diagnosis.

Differs from nearest occurring *Montenegrina laxa laxa* and *Montenegrina laxa disjuncta* Fehér & Szekeres, 2006 by its larger, stronger sculptured shell and weaker, shorter anterior plica superior, whereas from all other *Montenegrina laxa* subspecies by the combination of a large, tumid shell and long overlapping lamellae superior and spiralis.

##### Type locality.

Albania, Kukës District, S of Draj-Reç, entrance of the Vilë Gorge, 430 m, 41.8830°N, 20.3370°E.

##### Type material.

Type locality, leg. ZF, JG, 2.vii.2005, holotype (NHMW 111237), paratypes (NHMW 111235/58+5a+4aj, HNHM 99645/63, GR/63); Draj-Reç (11 km S of the Përroi i Bushtricës mouth), 550 m, 41.8908°N, 20.3370°E, leg. TD, ZE, ZF, DM, 9.x.2005, paratypes (HNHM 99646/15, MMM-B01328/15, ER/15, SZ/4).

##### Etymology.

The new taxon is named after Tamás Deli, malacologist (MMM-B), who participated in several Balkan field trips, including the one that led to the discovery of this subspecies.

##### Distribution.

Western foothills of the Korab Mts in northeastern Albania. Known from two nearby sites in the valley of the Black Drin (Drin i Zi), around Draj-Reç. This is the northernmost subspecies of this polytypic species (Fig. 23).

#### Montenegrina laxa disjuncta

Taxon classificationAnimaliaStylommatophoraClausiliidae

Fehér & Szekeres, 2006

Fig. 21D

Montenegrina laxa disjuncta Fehér & Szekeres, 2006 in Erőss et al. 2006: 197–198, fig. 19. – Nordsieck 2009: 73.

##### Diagnosis.

Shell medium, light corneous. All whorls smooth. Neck inflexed, finely striate-costate. Basal and peripheral crests well recognizable. Peristome angular, with broad, somewhat swollen margin. Lamellae superior and spiralis do not overlap. Subcolumellaris barely, clausilium plate not visible through the aperture. Lunella lateral to ventrolateral, basalis strong. Subclaustralis short, sulcalis residual or absent. Anterior plica superior long, starts diverging from, then turns parallel to the plica principalis. It is occasionally connected to the lunella complex.

##### Dimensions

(in mm). H_s_: 16.1–21.3 (holotype 17.7), W_s_: 3.6–4.5 (holotype 4.1).

##### Type locality.

Albania, Dibrë District, 4 km E of Selishtë, along the Peshkopi to Burrel road, 13 km W of the bridge of Fushë-Muhurr, 760 m, 41.6308°N, 20.2983°E.

##### Type material.

Type locality, leg. ZE, ZF, JK, DM, 26.vi.2003, holotype (HNHM 94871), paratypes (NHMUK 20050225, HNC 63189, HNHM 94872/25, NHMW 103286, RMNH 100326, SMF 328090).

##### Other material.

Type locality, leg. ZF, TN, EM, 14.iv.2014 (HNHM 98995); Selishtë, 770 m, 41.6280°N, 20.2741°E, leg. ZF, TN, EM, 14.iv.2014 (HNHM 98996); same locality, leg. ZE, ZF, JK, DM, 26.vi.2003 (HNHM 94873); same locality, leg. ZE, ZF, AH, DM, 13.iv.2006 (HNHM 96808); 2 km E of Selishtë, 760 m, 41.6228°N, 20.2886°E, leg. ZF, TN, EM, 14.iv.2014 (HNHM 99000); 3 km E of Selishtë, along the road to Gjuras, 1000 m, 41.6186°N, 20.2895°E, leg. TD, ZE, ZF, DM, 11.x.2005 (HNHM 96810).

##### Distribution.

This taxon is known from the vicinity of Selishtë (west of Peshkopi in Central Albania) (Fig. 23).

#### Montenegrina laxa errans

Taxon classificationAnimaliaStylommatophoraClausiliidae

Erőss & Szekeres, 2006

Fig. 21E

Montenegrina laxa errans Erőss & Szekeres, 2006 in Erőss et al. 2006: 198, fig. 20. – Nordsieck 2009: 73.

##### Diagnosis.

Shell slender, medium to large, yellowish-corneous. Lower whorls smooth, upper ones finely striate. Neck inflexed, striate. Basal and peripheral crests weak. Peristome angular, with somewhat swollen margin. Lamellae superior and spiralis do not overlap. Subcolumellaris and clausilium plate not visible through the aperture. Lunella lateral to ventrolateral, basalis strong. Subclaustralis short, sulcalis residual or absent. Anterior plica superior long, starts diverging from, then turns parallel to the plica principalis. It is not connected to the lunella complex.

##### Dimensions

(in mm). H_s_: 16.0–19.9 (holotype 19.9), W_s_: 3.6–4.3 (holotype 4.0).

##### Type locality.

Albania, Pogradec District, Çervenakë, 6 km from the Lin to Pogradec road toward the TV transmission tower, 1150 m, 40.9444°N, 20.6109°E.

##### Type material.

Type locality, leg. ZE, ZF, JK, DM, 2.vii.2003, holotype (HNHM 94874), paratypes (NHMUK 20050226, HNC 63190, HNHM 94875/66, NHMW 103287, RMNH 100314, SMF 328091, NMBE 22599/3).

##### Other material.

Type locality, leg. ZE, ZF, JG, 29.vi.2013 (HNHM 99631); 4 km SW of Bishnicë, Shkemb i Qytetit, 1140 m, 40.9210°N, 20.4491°E, leg. ZF, TN, EM, 12.iv.2014 (HNHM 98950); Librazhd District, Stravaj, 790 m, 41.0042°N, 20.4343°E, leg. ZF, TN, EM, 12.iv.2014 (HNHM 98952); Korçë District, NE of Strelcë, limestone gorge of the Lumi i Verbës at the foot of the Shkëmb i Selcës, 990 m, 40.7480°N, 20.5219°E, leg. ZF, JG, 30.vi.2013 (NHMW 110430/MN/0103); same locality, leg. ZB, CN, DP, 23.v.2007 (HNHM 99632).

##### Distribution.

This is the southernmost *Montenegrina laxa* subspecies, found sporadically around the Mokër Mts in southeastern Albania (Fig. 23).

#### Montenegrina laxa iba

Taxon classificationAnimaliaStylommatophoraClausiliidae

Nordsieck, 1972

Fig. 21F

Montenegrina laxa iba Nordsieck, 1972: 30, plate 5, fig. 41. – Zilch 1981: 130, plate 13, fig. 23. – Nordsieck 2009: 73.

##### Diagnosis.

Shell medium, yellowish-corneous. Lower whorls smooth, upper ones finely striate. Neck weakly inflexed, densely costate. Basal and peripheral crests well recognizable. Peristome ovoid to somewhat angular, with simple margin. Lamellae superior and spiralis mostly do not overlap. Subcolumellaris and clausilium plate barely visible through the aperture. Lunella dorsolateral to lateral, basalis short. Subclaustralis shorter than the basalis, sulcalis residual or absent. Anterior plica superior weak, not connected to the lunella complex, rarely absent.

##### Dimensions

(in mm). H_s_: 12.3–17.8 (holotype 16.2), W_s_: 3.4–4.2 (holotype 3.7).

##### Type locality.

“Arzen-Durchbruch bei Ibë” = Albania, Tiranë District, gorge of the Erzen near Ibë.

##### Type material.

Type locality (“beim Krabapaß”), leg. Fuchs, holotype (SMF 201629a), paratypes (SMF 201629b/3, NHMW-E 28704/pl., NHMW-E 32270/30).

##### Other material.

Original series but not type (NHMW-E 34152, NHMW 87658, NHMW-K 54386); Albania, gorge of the Lumi i Erzenit, 290 m, 41.2598°N, 19.9676°E, leg. ZF, TN, EM, 17.iv.2014 (HNHM 99036); same locality, leg. ZE, ZF, KK, 11.iv.2001 (HNHM 85891); Gramsh District, Tërvol, gorge of the Përroi i Holtit, 250 m, 40.9261°N, 20.2232°E, leg. ZF, AH, TH, DM, 26.viii.2006 (HNHM 99629).

##### Distribution.

Central Albania. For a long time this taxon has been known only from the type locality, but a recently discovered population more than 30 km farther south indicates that it may have a wider, sporadic distribution (Fig. 23).

#### Montenegrina laxa kontschani

Taxon classificationAnimaliaStylommatophoraClausiliidae

Erőss & Szekeres, 2006

Fig. 21G

Montenegrina laxa kontschani Erőss & Szekeres, 2006 in Erőss et al. 2006: 199–200, fig. 21. – Nordsieck 2009: 73.

##### Diagnosis.

Shell medium to large, yellowish-corneous. All whorls smooth. Neck inflexed, striate. Basal and peripheral crests well recognizable. Peristome rounded to somewhat angular, with swollen, reflexed margin. Lamellae superior and spiralis overlap. Subcolumellar lamella not or barely, clausilium plate not at all visible through the aperture. Lunella lateral. Subclaustralis shorter than the basalis, sulcalis present. Anterior plica superior very long, starts diverging from, then turns parallel or even converging to, the plica principalis. It is mostly separate from the lunella complex.

##### Dimensions

(in mm). H_s_: 14.8–19.3 (holotype 19.0), W_s_: 3.7–4.8 mm (holotype 4.1).

##### Type locality.

Albania, Tiranë District, 6 km S of the Qafa e Shtyllës, 7.7 km S of the Elbasan junction along the Tiranë to Klos road, 1420 m, 41.3512°N, 20.0546°E.

##### Type material.

Type locality, leg. ZE, ZF, JK, DM, 22.x.2002, holotype (HNHM 94878), paratypes (NHMUK 20050227, HNC 63191, HNHM 94879/70, NHMW 103288, RMNH 100325, SMF 328092, NMBE 534925/4).

##### Other material.

Albania, Tiranë District, Gropa Mts, ca. 3 km E of the Shën Mëri junction along the Tiranë to Bizë road, 1400 m, 41.3512°N, 20.0502°E, leg. ZF, TK, DM, 20.vi.2012 (HNHM 99633); ca. 3.5 km E of the Shën Mëri junction, 1400 m, 41.3507°N, 20.0583°E, leg. ZF, TK, DM, 20.vi.2012 (HNHM 99634); Shtyllë Pass, 1.3 km N of the Elbasan junction at the Tiranë to Bizë road, 1520 m, 41.3705°N, 20.0855°E, leg. ZF, TK, DM, 20.vi.2012 (HNHM 99635); same locality, leg. ZE, ZF, JK, DM, 22.x.2002 (HNHM 94880); along the Klos to Elbasan road, 3 km E of the Tiranë junction, 1380 m, 41.3515°N, 20.1163°E, leg. ZF, JK, DM, 9.x.2004 (HNHM 94882).

##### Distribution.

Southwestern part of the Gropa-Bizë-Martanesh mountain group in Central Albania (Fig. 23).

#### Montenegrina laxa lakmosensis

Taxon classificationAnimaliaStylommatophoraClausiliidae

Nordsieck, 2009

Fig. 21H

Montenegrina janinensis – Sattmann and Reischütz 1994: 46.
Montenegrina dedovi lakmosensis Nordsieck, 2009: 77, plate 2, fig. 5.

##### Diagnosis.

Shell small, tumid, yellowish-corneous. All whorls smooth. Neck weakly inflexed, densely striate. Basal and peripheral crests weak. Peristome ovoid to somewhat angular, with slightly swollen margin. Lamella superior weak and short, distant from the spiralis. In front view lamella inferior moderately emerged. Subcolumellaris and clausilium plate not or barely visible through the aperture. Lunella dorsolateral to lateral, basalis short. Subclaustralis shorter than the basalis, sulcalis residual or absent. Anterior plica superior mostly absent, if present separate from the lunella complex.

##### Dimensions

(in mm). H_s_: 12.8–15.0 (holotype 14.1), W_s_: 3.8–4.1 (holotype 4.0).

##### Type locality.

Greece, Lakmos Mts, Epirus–Thessalia border, near Metsovon, S of the Peristeri Summit, ca. 2000 m.

##### Type material.

Type locality, leg. HS, 2.vii.1988, holotype (SMNS 70544), paratypes (SMNS-N 9776/12, SMF 332354/1).

##### Other material.

Original series but not type (NHMW 102868)

##### Distribution.

Lakmos (= Peristeri) Mts in northwestern Greece. Known only from the type locality (Fig. 23).

##### Remarks.

Originally *Montenegrina laxa lakmosensis* was described by Nordsieck (2008) as a subspecies of *dedovi*, which is now also classified with *Montenegrina laxa* (see above).

#### Montenegrina laxa miraka

Taxon classificationAnimaliaStylommatophoraClausiliidae

Nordsieck, 1996

Fig. 21I

Montenegrina laxa miraka Nordsieck, 1996: 8–9, plate 2, fig. 2. – Nordsieck 2009: 73.

##### Diagnosis.

Shell medium to large, yellowish-corneous. All whorls smooth. Neck inflexed, densely striate-costate. Basal and peripheral crests well recognizable. Peristome ovoid to somewhat angular, with simple to weakly swollen margin. Lamellae superior and spiralis long overlap. In front view lamella inferior mostly well emerged. Subcolumellaris and clausilium plate not visible through the aperture. Lunella ventrolateral-lateral to ventrolateral, basalis long. Subclaustralis shorter than the basalis, sulcalis residual or absent. Anterior plica superior very long, starts diverging from, then turns parallel or even converging to the principalis. It is not connected to the lunella complex.

##### Dimensions

(in mm). H_s_: 16.6–22.5 (holotype 22.1), W_s_: 4.0–5.4 (holotype 5.1).

##### Type locality.

Albania, Librazhd District, Mirakë, 5 km W of Librazhd = 1 km NW Ura e Kamarës, S slope, 300 m.

##### Type material.

Type locality, leg. Welter-Schultes, 17.ix.1995, holotype (HNC 40580), paratypes (HNC 40577, SMNS-N 10305, NHMW 89037/2, SMF 311198/2).

##### Other material.

Albania, Mirakë, Ura e Kamarës, 190 m, 41.1634°N, 20.2301°E, leg. ZF, TK, DM, 22.vi.2012 (HNHM 99630); Mirakë, right bank of Lumi i Shkumbinit, 210 m, 41.1657°N, 20.2377°E, leg. ZE, ZF, JK, DM, 24.x.2002 (HNHM 91605); 1 km S of Lunik, along the Librazhd to Peshkopi road, 700 m, 41.2661°N, 20.3181°E, leg. ZE, ZF, JK, DM, 24.x.2002 (HNHM 94905); same locality, leg. ZF, TN, EM, 13.iv.2014 (HNHM 98964); E of Lunik, 750 m, 41.2768°N, 20.3352°E, leg. ZE, ZF, AH, DM, 11.iv.2006 (HNHM 96805); same locality, leg. ZF, TN, EM, 13.iv.2014 (HNHM 98969); Qukës-Shkumbin, above a quarry, 500 m, 41.0937°N, 20.4402°E, leg. ZE, ZF, JK, DM, 30.vi.2003 (HNHM 94902); 6 km W of Librazhd, 230 m, 41.1676°N, 20.2498°E, leg. ZE, ZF, JK, DM, 30.vi.2003 (HNHM 94897); Elbasan District, Mengli, small gorge near a quarry, 250 m, 41.1335°N, 20.1261°E, leg. ZF, TN, EM, 17.iv.2014 (HNHM 99046).

##### Distribution.

Çermenikë Mountain in central Albania. Known from a few localities around this mountain within a circle of 10–15 km radius around Librazhd (Fig. 23).

#### Montenegrina lillae sp. n.

Taxon classificationAnimaliaStylommatophoraClausiliidae

http://zoobank.org/1EE8B181-9952-4632-A116-B6BF1392BD43

Fig. 21L

##### Diagnosis.

Large, elongate species with wide, broade-lipped aperture, lateral to ventrolateral lunella and long, fused basalis.

##### Description.

The large, elongate shell of 10½ to 12 whorls is light brown. The surface of the whorls is opaque, very finely wrikled, almost smooth. The weakly inflexed neck has strong whitish ribs that become dense toward the aperture. The basal crest is strong, the peripheral one is weak. The peristome is large, angular, broadly attached. It has wide, whitish, somewhat swollen margin, which is missing at the upper columellar side. The lamella superior is weak, it does not overlap with the spiralis. The terminal part of the lamella inferior is well emerged, descends seeply and ends low at the columellar side of the peristome. The weakly-bent lamella subcolumellaris is retracted, its end is not visible through the aperture. The plica superior often becomes fused to the principalis. The broad, lateral to ventrolateral lunella is connected to the long basalis. The subclaustralis is short, diffuse, the sulcalis is well developed. The anterior part of the plica superior is long, often separate from the lunella complex. The clausilium plate is not visible through the aperture.

##### Dimensions

(in mm). Holotype H_s_: 23.0, W_s_: 5.0, H_a_: 5.6, W_a_: 4.7; paratypes (HNHM 99497, n = 12): H_s_: 19.7–23.4 (mean 21.4, S.D. 1.09), W_s_: 4.8–5.6 (mean 5.2, S.D. 0.22), H_a_: 5.0–5.7, W_a_: 4.1–4.8.

##### Differential diagnosis.

Differs from all other species of the genus by the combination of a broadly attached peristome with dissolved upper margin, weak lamella superior, and deep subcolumellaris. From *Montenegrina subcristata*, the only other species of the genus with lateral to ventrolateral lunella, it is distinguished by the basalis that is fused to the lunella complex.

##### Type locality.

Albania, Shkodër District, Koman, ferry harbor near the dam, 180 m, 42.1087°N, 19.8264°E.

##### Type material.

Type locality, leg. ZE, ZF, AH, DM, 14.iv.2006, holotype (HNHM 99496), paratypes (HNHM 99497/67+9aj, ER/67, HU/67, SZ/10); type locality, leg. ZF, TK, DM, 19.vi.2012, paratypes (HNHM 99498/5, NHMW 111227/6+3a).

##### Other material.

Albania, Koman Lake, right bank, ca. 12 km upstream of the Koman Dam, 170 m, 42.1872°N, 19.8696°E, leg. ZE, ZF, AH, DM, 15.iv.2006 (HNHM 99499).

##### Etymology.

This species is named after Lilla Tamás, the wife of the first author.

##### Distribution.

Lower part of the Drin Valley. Known from the Koman Dam and 12 km upstream of that (Fig. 22).

#### Montenegrina minuscula

Taxon classificationAnimaliaStylommatophoraClausiliidae

Erőss & Szekeres, 2006

Fig. 19L

Montenegrina janinensis – Dhora and Welter-Schultes 1999: 16.
Montenegrina minuscula Erőss & Szekeres, 2006 in Erőss et al. 2006: 184–185, fig. 2. – Nordsieck 2009: 75.

##### Diagnosis.

Shell very small, light corneous. All whorls smooth. Neck finely striate, deep inflexed, with strong basal and peripheral crests. Peristome detached, projected, pear-shaped, with simple margin. Lamellae superior and spiralis overlap. In front view lamella inferior hidden or barely emerged. Subcolumellaris and clausilium plate barely visible through the aperture. Lunella dorsolateral-lateral, separate from the short to rudimentary basalis. Subclaustralis absent, sulcalis residual or absent. Anterior plica superior only exceptionally present, either fused to or separate from the lunella complex.

##### Dimensions

(in mm). H_s_: 9.1–11.5 (holotype 9.9), W_s_: 2.3–2.9 (holotype 2.7).

##### Type locality.

Albania. Mat District, 3 km W of the Qafa e Murrës, gorge of the Lumi i Varoshit near the Shkëmb i Skanderbeut, 970 m, 41.6465°N, 20.1898°E.

##### Type material.

Type locality, leg. ZE, ZF, JK, DM, 26.vi.2003, holotype (HNHM 94831), paratypes NHMUK 20050214/2, HNC 63178/2, HNHM 94832/229, NHMW 103272/2, RMNH 100319/2, SMF 328076/2, NMBE 534918/6).

##### Other material.

Type locality, leg. TD, ZE, ZF, DM, 11.x.2005 (HNHM 96824); Mirditë District, 2 km NE of Kurbnesh, 800 m, 41.7952°N, 20.1117°E, leg. ZF, DM, ZU, 20.v.2010 (HNHM 99625); 1 km N of Kurbnesh, 41.7883°N, 20.1068°E, leg. ZE, ZF, JK, DM, 27.vi.2003 (HNHM 94833).

##### Distribution.

Lura-Dejë mountain group in northern Albania. Known only from two sites: the type locality at the southern foothills of the Mt. Lura in the gorge of the Varosh Stream, and the locality in the valley of the Urakë River, at the western foot of the Dejë. Although the elevation seems different, the record “Kurbnesh, 400 m” (Dhora and Welter-Schultes 1999) most probably refers to the latter occurrence (Fig. 22).

##### Remarks.

In the gorge of the Varosh Stream this species occurs syntopically with *Montenegrina skipetarica skipetarica*.

#### Montenegrina nana

Taxon classificationAnimaliaStylommatophoraClausiliidae

Fehér & Szekeres, 2006

##### Diagnosis.

Shell small to medium, smooth to strongly wrinkled-costate. Neck weakly inflexed to inflexed, basal and peripheral crests well recognizable. In front view lamella inferior moderately emerged, medium-bent subcolumellaris not or only barely visible. Lunella dorsolateral to lateral, mostly fused to the basalis. Subclaustralis and sulcalis present. Anterior plica superior absent or weak. Clausilium plate not or barely visible through the aperture.

##### Remarks.

*Montenegrina nana*, which was formerly classified as a subspecies of *Montenegrina perstriata*, differs from this species by its smaller size, oblique peristome, and deeper inflexed neck. One of the subspecies, *Montenegrina nana barinai* ssp. n., occurs syntopically and does not hybridize with *Montenegrina perstriata ochridensis*, indicating that they are distinct species.

#### Montenegrina nana nana

Taxon classificationAnimaliaStylommatophoraClausiliidae

Fehér & Szekeres, 2006

Fig. 24A

Montenegrina perstriata nana Fehér & Szekeres, 2006 in Erőss et al. 2006: 202–203, fig. 24. – Nordsieck 2009: 74, plate 3, fig. 17.

##### Diagnosis.

Shell small to medium, light corneous. All whorls smooth. Neck weakly inflexed, smooth to very finely striate. Basal and peripheral crests well recognizable. Lamellae superior and spiralis mostly overlap. Lamella subcolumellaris not or only barely visible through the aperture. Lunella lateral, fused to the short basalis. Subclaustralis residual or absent, sulcalis present. Anterior plica superior absent or weak, parallel to the principalis, separate from the lunella complex. Clausilium plate not visible through the aperture.

##### Dimensions

(in mm). Hs: 14.3–16.1 (holotype 15.1), Ws: 3.3–3.7 (holotype 3.7).

**Figure 24.**
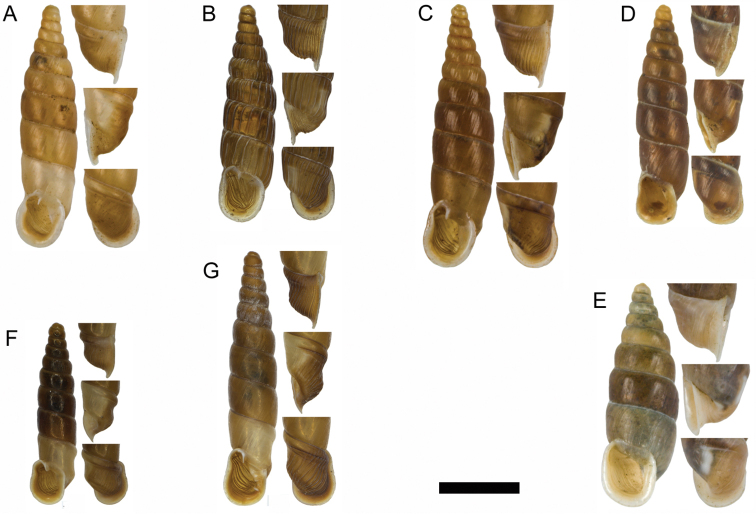
A. *Montenegrina nana nana* Fehér & Szekeres, 2006, holotype, HNHM 94889 **B** *Montenegrina nana barinai* ssp. n., holotype, HNHM 99508 **C** *Montenegrina nana gracilis* Erőss & Szekeres, 2006, holotype, HNHM 94886 **D** *Montenegrina okolensis okolensis* Szekeres, 2006, holotype, HNHM 94839 **E** *Montenegrina okolensis caesia* Fehér & Szekeres, 2009, holotype, HNHM 94835 **F** *Montenegrina prokletiana prokletiana* ssp. n., holotype, HNHM 99490 **G** *Montenegrina prokletiana kovacsorum* ssp. n., holotype, HNHM 99485. Scale bar: 5 mm.

##### Type locality.

Albania, Librazhd District, 3 km NE of Lunik, on the Librazhd–Peshkopi road, 1050 m, 41.2966°N, 20.3741°E.

##### Type material.

Type locality, leg. ZE, ZF, JK, DM, 24.ix.2002, holotype (HNHM 94889), paratypes (HNHM 94890/5, NHMW 103291, SMF 328095).

##### Other material.

Type locality, leg. ZF, TN, EM, 13 Apr. 2014 (HNHM 98973); 8.3 km S of Steblevë, at the mountain pass, 41.322°N, 20.394°E, leg. AR, NR, PR, ix.2013 (NHMW 110430/MN/0085); SW of Zabzun, 2 km toward Sebisht from the Librazhd to Peshkopi road, 1240 m, 41.3339°N, 20.3958°E, leg. ZF, 13.iv.2014 (HNHM 98978).

##### Distribution.

**Figure 25.**
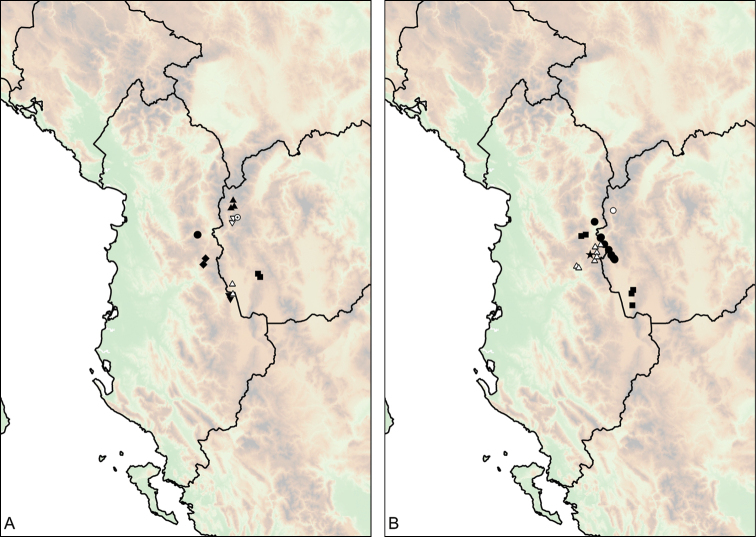
Distribution of *Montenegrina nana* and *Montenegrina perstriata*. **A** *Montenegrina nana gracilis* and *Montenegrina nana barinai* ssp. n. (circle); *Montenegrina nana nana* (diamond); *Montenegrina perstriata diminuta* (square); *Montenegrina perstriata mavrovoensis* (empty circle with dot); *Montenegrina perstriata occidentalis* (empty triangle); *Montenegrina perstriata perstriata* (empty inverted triangle); *Montenegrina perstriata plenostoma* (inverted triangle); *Montenegrina perstriata subcristatula* (triangle) **B** *Montenegrina perstriata callistoma* (empty triangle); *Montenegrina perstriata drimica* (circle); *Montenegrina perstriata ochridensis* (square); *Montenegrina perstriata radikae* (empty circle); *Montenegrina perstriata tenebrosa* (star); *Montenegrina perstriata steffeki* is not shown.

Gollobordë Plateau in northeastern Albania (Fig. 25A).

#### Montenegrina nana barinai ssp. n.

Taxon classificationAnimaliaStylommatophoraClausiliidae

http://zoobank.org/D38AA9A0-A189-49C2-974E-CF52094AA241

Fig. 24B

##### Diagnosis.

Small subspecies with strongly costate shell, widely separate lamellae inferior and spiralis, missing anterior plica superior.

##### Description.

The small shell of 9½ to 10½ whorls is light brownish-corneous. All whorls are strongly costate. The sharp whitish ribs become crowded behind the aperture. The neck is inflexed, the basal and peripheral crests are well recognizable. The peristome is ovoid, attached, its wide, light brown margin is slightly deflexed. The weak lamella superior ends far from the outer end of the spiralis. The weakly emerged, straight descending end of the lamella inferior is barely visible in front view. The deep-ending, medium-bent lamella subcolumellaris cannot be viewed through the aperture. The broad lunella is dorsolateral. It is fused, via the superior, to the plica principalis, and at its basal end to the short or residual basalis. The subclaustralis is weak, the sulcalis is present. The anterior part of the plica superior is missing. The parietal edge of the clausilium plate is often visible through the aperture.

##### Dimensions

(in mm). Holotype Hs: 13.2, Ws: 3.6, Ha: 3.5, Wa: 2.9; paratypes (HNHM 99008, n = 12): Hs: 13.0–15.8 (mean 13.8, S.D. 0.95), Ws: 3.4–4.6 (mean 3.9, S.D. 0.34), Ha: 3.1–3.9, Wa: 2.3–3.3.

##### Differential diagnosis.

Distinguishable from the two other subspecies of *Montenegrina nana* by its smaller, strongly costate shell, wide separate lamellae superior and spiralis, dorsolateral lunella, and the absence of the anterior plica superior.

##### Type locality.

Albania, Bulqizë District, Valikardhë, S slope of the Maja e Temlishit, 770 m, 41.514°N, 20.316°E.

##### Type material.

Type locality, leg. ZF, TN, EM, 15.iv.2014, holotype (HNHM 99508), paratypes (HNHM 99008/39+20a+1aj, NHMW 111231/35a, SZ/4).

##### Other material.

Type locality, 880 m, 41.5138°N, 20.3142°E, leg. ZB, DP, 29.v.2008, (HNHM 99509).

##### Etymology.

The new taxon is named after Zoltán Barina, botanist (HNHM), who collected invaluable zoological material during his field trips in Albania.

##### Distribution.

Known only from its type locality at the southern slope of the Mt. Temlishi in northern Albania. (Fig. 25).

##### Remarks.

The type locality of the new subspecies is sepatated only by a few hundred meters from that of *Montenegrina nana gracilis* Erőss & Szekeres, 2006. *Montenegrina nana barinai* ssp. n. occurs syntopically with *Montenegrina perstriata ochridensis*.

#### Montenegrina nana gracilis

Taxon classificationAnimaliaStylommatophoraClausiliidae

Erőss & Szekeres, 2006

Fig. 24C

Montenegrina perstriata gracilis Erőss & Szekeres, 2006 in Erőss et al. 2006: 202, fig. 23. – Nordsieck 2009: 74.

##### Diagnosis.

Shell small to medium, light to dark corneous. Lower whorls smooth, upper ones indistinctly costate. Inflexed neck with sharp, irregular ribs. Basal crest strong, peripheral crest well recognizable. Lamellae superior and spiralis mostly do not overlap. Lamella subcolumellaris not visible through the aperture. Lunella dorsolateral-lateral to lateral, mostly fused to the short basalis. Subclaustralis and sulcalis well developed. Anterior plica superior absent or weak, parallel to the principalis, separate from the lunella complex. Clausilium plate not visible through the aperture.

##### Dimensions

(in mm). Hs: 14.2–20.1 mm (holotype 16.5), Ws: 3.5– 4.7 mm (holotype 3.9).

##### Type locality.

Albania, Bulqizë District, 10 km E of Bulqizë, bank of Lumi i Zalli i Qytetit, 620 m, 41.5096°N, 20.3149°E.

##### Type material.

Type locality, leg. ZE, ZF, JK, DM, 25.x.2002, holotype (HNHM 94886), paratypes (NHMUK 20050229, HNC 63193, HNHM 94887/90, NHMW 103290, RMNH 100322, SMF 328094, NMBE 535066/4).

##### Other material.

Type locality, leg. LD, ZE, ZF, AH, DM, 30.vi.2007 (HNHM 99624).

##### Distribution.

Mt. Temlishi in northern Albania. Known only from the type locality at the foot of this mountain (Fig. 25A).

##### Remarks.

Erroneously this taxon was also mentioned from the vicinity of the Tre Çesmë Spring near Zerqan (Erőss et al. 2006), a locality of *Montenegrina perstriata ochridensis* (see also there).

#### Montenegrina okolensis

Taxon classificationAnimaliaStylommatophoraClausiliidae

Szekeres, 2006

##### Diagnosis.

Shell small to medium, elongate, light to dark corneous. All whorls smooth. Neck inflexed, almost smooth, basal and peripheral crests weak. Peristome attached, ovoid to somewhat angular, with somewhat swollen margin. Lamella superior absent to residual and distant from the spiralis. In front view lamella inferior hidden to weakly emerged. Medium-bent subcolumellaris only obliquely visible. Lunella dorsal-dorsolateral to dorsolateral. Basalis and subclaustralis absent. Sulcalis present or only residual. Anterior plica superior mostly absent. Clausilium plate visible through the aperture. Distinguishable from *Montenegrina apfelbecki* by the attached peristome and reduced lamellae.

#### Montenegrina okolensis okolensis

Taxon classificationAnimaliaStylommatophoraClausiliidae

Szekeres, 2006

Fig. 24D

Montenegrina apfelbecki okolensis Szekeres, 2006 in Erőss et al. 2006: 186, fig. 5.
Montenegrina janinensis caesia (partim) – Nordsieck 2009: 75.

##### Diagnosis.

Shell small to medium, elongate. In front view lamella inferior weakly emerged. Lunella dorsolateral. Sulcalis present. Anterior plica superior mostly absent. Clausilium plate partly visible through the aperture.

##### Dimensions

(in mm). H_s_: 13.9–15.6 (holotype 13.9), W_s_: 3.4–4.4 mm (holotype 3.4) (see type and other material).

##### Type locality.

“Theth valley” = Albania, Prokletije Mts, one of the mountains surrounding the Theth Valley (probably Mt. Jezercë).

##### Type material.

“Theth valley”, ex Nádai, ex Holzinger, collected by an unknown mountaineer, vii.1996, holotype (HNHM 94839).

##### Other material.

Albania, Malesia District, above the N side of the Qafa e Valbonës, along the footpath between Rragam and Theth, 1850 m, 42.4068°N, 19.8122°E, leg. TD, ZE, ZF, DM, 06.x.2005 (HNHM 99613); same locality, leg. Ádám, GP, Somay, 5.ix.2005 (HNHM 99614).

##### Distribution.

In the central part of the Prokletije Mts in northern Albania.

**Figure 26.**
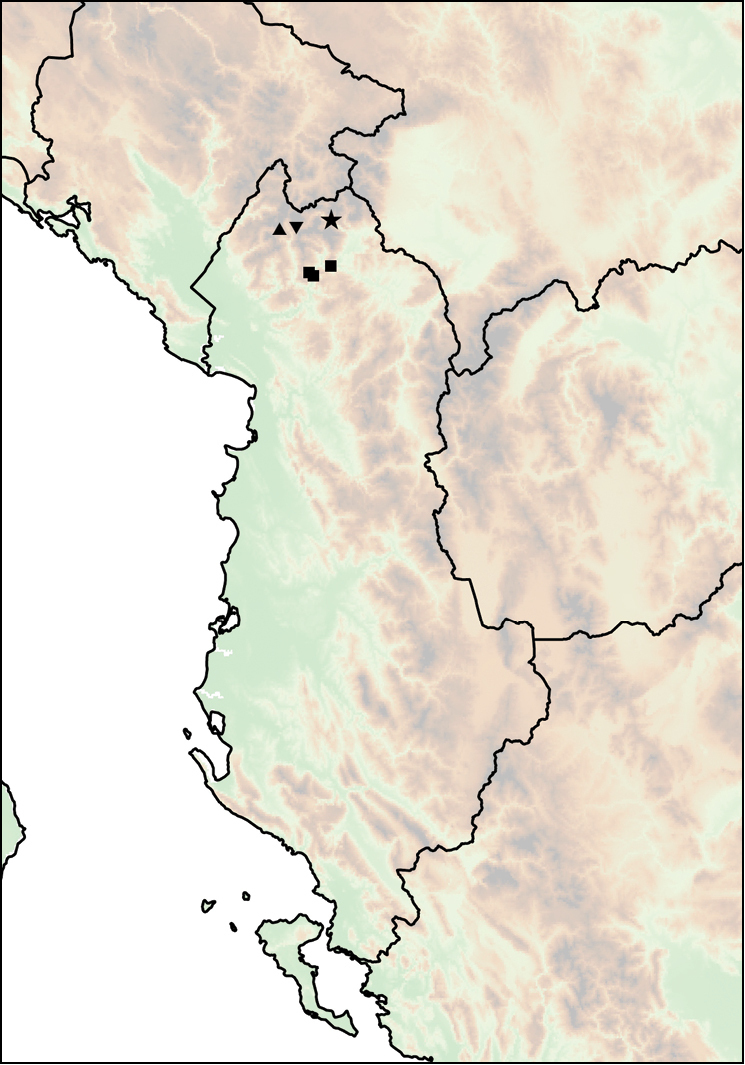
Distribution of *Montenegrina okolensis* and *Montenegrina prokletiana* sp. n. *Montenegrina okolensis caesia* (triangle); *Montenegrina okolensis okolensis* (inverted triangle),; *Montenegrina prokletiana prokletiana* ssp. n. (star); *Montenegrina prokletiana kovacsorum* ssp. n. (square).

The description of this subspecies was based on a single shell without precise locality and collector information. Accordingly, type locality was defined only as “Maja e Jezerces region” (Erőss et al. 2006). Recently the subspecies has been re-discovered near the Valbona Pass (Qafa e Valbonës), south of the Jezercë Summit. Notably, along the Theth to Rragam footpath that crosses the main mountain ridge, *Montenegrina okolensis okolensis* could be found only around its highest point, near to and above the pass, between 1760 and 1850 m (see also: Fehér and Erőss 2009) (Fig. 26).

##### Remarks.

Nordsieck (2009) considered *okolensis* a synonym of *caesia* Fehér & Szekeres, 2006. New *okolensis* samples confirm the stability of its morphological differences from *caesia*, namely the elongate shell, deeper lunella and better developed sulcalis. Therefore we maintain it as a distinct subspecies.

#### Montenegrina okolensis caesia

Taxon classificationAnimaliaStylommatophoraClausiliidae

Fehér & Szekeres, 2006

Fig. 24E

Montenegrina apfelbecki caesia Fehér & Szekeres, 2006 in Erőss et al. 2006: 185, fig. 3.
Montenegrina janinensis caesia (partim) – Nordsieck 2009: 75.

##### Diagnosis.

Shell small, tumid. In front view lamella inferior hidden. Lunella dorsal-dorsolateral. Sulcalis residual. Anterior plica superior absent. Clausilium plate almost entirely visible through the aperture.

##### Dimensions

(in mm). H_s_: 12.2–16.3 (holotype 14.3), W_s_: 3.9–4.8 (holotype 4.5 mm).

##### Type locality.

Albania, Prokletije Mts, N side of the Qafa e Tërthorës, 11 km from Bogë toward Theth, 1800 m, 42.392°N, 19.730°E.

##### Type material.

Type locality, leg. ZE, ZF, JK, DM, 20.x.2002, holotype (HNHM 94835), paratypes (NHMUK 20050215, HNC 63179, HNHM 94836/53, NHMW 103273, RMNH 100313, SMF 328077, NMBE 534893/4).

##### Distribution.

Central part of the Prokletije Mts in northern Albania. Known only from the type locality (Fig. 26).

##### Remarks.

Originally *Montenegrina okolensis okolensis* and *Montenegrina okolensis caesia* were described as subspecies *Montenegrina apfelbecki*, with which they share similar plicae. However, their reduced clausiliar apparatus and isolated occurrence supports their classification within a separate species, which seems more closely related to other north Albanian representatives of the genus (e.g. *Montenegrina prokletiana* sp. n.).

#### Montenegrina perstriata

Taxon classificationAnimaliaStylommatophoraClausiliidae

(Wagner, 1919)

##### Diagnosis.

Shell medium to large, light to dark corneous. Lower whorls in some subspecies flattened and nearly of the same width. Whorls smooth to indistinctly costate. Neck weakly inflexed, basal and peripheral crests well recognizable. Peristome attached, angular, with simple to strongly swollen margin. Lamellae superior and spiralis distant to overlapping. In front view lamella inferior moderately emerged, medium-bent subcolumellaris mostly not visible. Lunella dorsolateral to ventrolateral, mostly not connected to the basalis. Subclaustralis often short, sulcalis present. Plica superior frequently fused to the principalis. Anterior plica superior absent or weak. Clausilium plate not or only barely visible through the aperture.

#### Montenegrina perstriata perstriata

Taxon classificationAnimaliaStylommatophoraClausiliidae

(Wagner, 1919)

Fig. 27A

Delima laxa perstriata Wagner, 1919: 71–72.
Delima (Albanodelima) perstriata – Wagner 1924: 120. – Wagner 1925: 61, plate 14, figs 97a–b.
Montenegrina perstriata perstriata – Nordsieck 1977: 84, plate 4, fig. 13. – Zilch 1981: 130. – Nordsieck 2009: 74.

##### Diagnosis.

Shell medium, tumid, dark corneous. Lower whorls smooth, flattened, nearly of the same width. Upper whorls very finely, indistinctly wrinkled-costate. Neck densely costate. Peristome with simple, somewhat reflexed margin. Lamellae superior and spiralis overlap. In front view lamella subcolumellaris not visible. Lunella dorsolateral, not connected to the basalis. Subclaustralis short, sulcalis present. Anterior plica superior absent or weak, separate from the lunella complex. Clausilium plate barely visible through the aperture.

##### Dimensions

(in mm). H_s_: 21.2, W_s_: 5.5 (holotype according to Nordsieck 1977).

**Figure 27.**
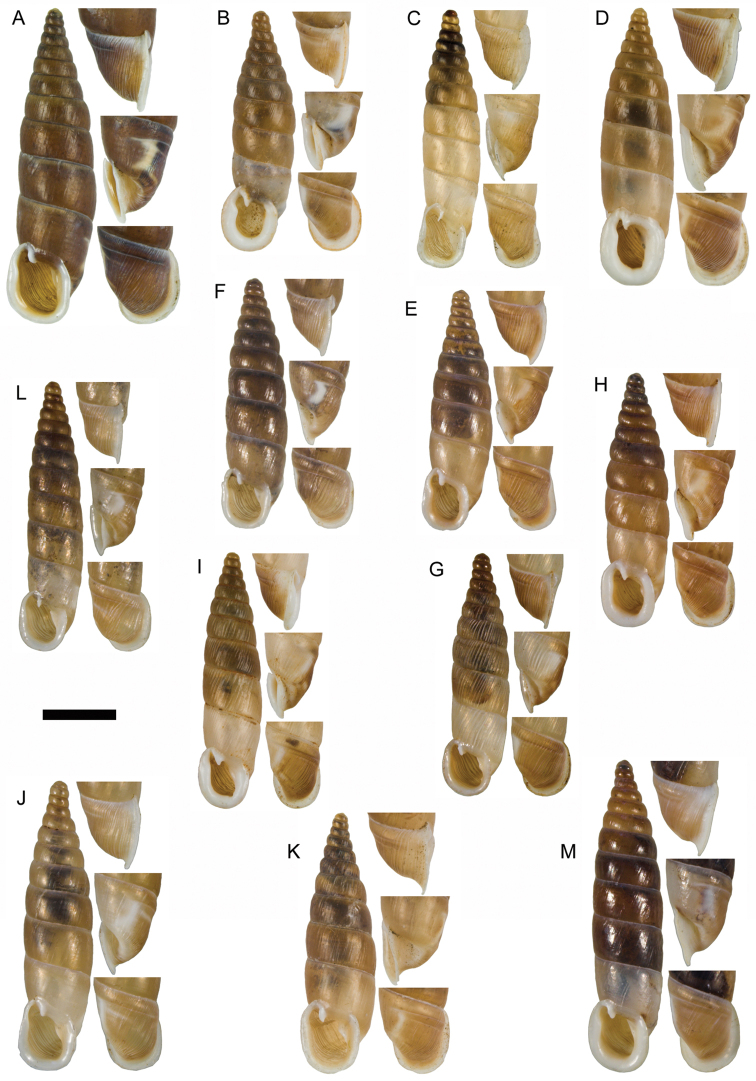
**A** *Montenegrina perstriata perstriata* (Wagner, 1919), Galičnik, NHMW 110430/MN/0105 **B** *Montenegrina perstriata callistoma* Fehér & Szekeres, 2006, holotype, HNHM 94883 **C** *Montenegrina perstriata diminuta* Fehér & Szekeres, 1999, holotype, HNHM 70832 **D** *Montenegrina perstriata drimica* Nordsieck, 1972, holotype of *crassa*, HNHM 70834 **E** *Montenegrina perstriata drimica* Nordsieck, 1972, holotype, SMF 221048 **F** *Montenegrina perstriata mavrovoensis* Nordsieck, 2009, paratype, SMF320048 **G** *Montenegrina perstriata occidentalis* Nordsieck, 1977, holotype, SMF 237804 **H** *Montenegrina perstriata ochridensis* (Wagner, 1925), Ohrid, SMF 167047 **I** *Montenegrina perstriata plenostoma* Fehér & Szekeres, 2006, holotype, HNHM 94840 **J** *Montenegrina perstriata radikae* Nordsieck, 1972, holotype, SMF 221050 **K** *Montenegrina perstriata steffeki* Erőss & Szekeres, 2006, holotype, HNHM 94891 **L** *Montenegrina perstriata subcristatula* Nordsieck, 1977, holotype, SMF 221318 **M** *Montenegrina perstriata tenebrosa* Nordsieck, 2009, paratype, SMF 328780. Scale bar: 5 mm.

##### Type locality.

Macedonia, Mavrovo District, Galičnik.

##### Type material.

According to Nordsieck (1977), the type specimen is in Warsaw (MIZPAS 10114) (not seen).

##### Other material.

Macedonia, 1 km E of Galičnik, 1440 m, 41.5942°N, 20.6716°E, leg. ZF, EH, KJ, HS, 14.x.2014 (NHMW 110430/MN/0104); same locality, leg. ZE, ZF, AH, 9.iv.2004 (HNHM 94436); Galičnik, 1440 m, 41.5936°N, 20.6575°E, leg. ZF, EH, KJ, HS, 14.x.2014 (NHMW 110430/MN/0105); same locality, leg. ZE, ZF, AH, 9.iv.2004 (HNHM 94437); 10 km SW from Mavrovo toward Galičnik and 1 km W on a minor road, 1740 m, 41.6240°N, 20.6768°E, leg. ZF, EH, KJ, HS, 14.x.2014 (NHMW 110430/MN/0106).

##### Remarks.

Wagner (1925) clarified that the unintentional assignement of this taxon to *laxa* in the original description was due to an editorial error during publication.

##### Distribution.

Western part of the Bistra Mts in the vicinity of Galičnik (Fig. 25).

#### Montenegrina perstriata callistoma

Taxon classificationAnimaliaStylommatophoraClausiliidae

Fehér & Szekeres, 2006

Fig. 27B

Montenegrina perstriata callistoma Fehér & Szekeres, 2006 in Erőss et al. 2006: 200–202, fig. 22. – Nordsieck 2009: 74, plate 3, fig. 16.
Montenegrina perstriata crassa – Fehér and Erőss 2009: 11.
Montenegrina perstriata “*crassa*” – Nordsieck 2009: 75, plate 3, fig. 15.

##### Diagnosis.

Shell medium, light corneous. Lower whorls smooth, upper ones very finely and densely striate. Neck densely striate. Peristome rounded to angular, with strongly swollen, reflexed margin. Lamellae superior and spiralis overlap to long overlap. Subcolumellaris not or barely, clausilium plate not at all visible through the aperture. Lunella lateral, separate from the basalis. Sulcalis residual to weak. Anterior plica superior separate from the lunella complex.

##### Dimensions

(in mm). H_s_: 14.3–18.1 (holotype 16.0), W_s_: 3.2–4.3 (holotype 4.2).

##### Type locality.

Albania, Bulqizë District, Klenjë, near a cave along the Librazhd to Peshkopi road, 1220 m, 41.3646°N, 20.4685°E.

##### Type material.

Type locality, leg. ZE, ZF, JK, DM, 24.x.2002, holotype (HNHM 94883), paratypes (NHMUK 20050228, HNC 63192, HNHM 94884/29, NHMW 103289, RMNH 100320, SMF 328093).

##### Other material.

Type locality, leg. ZF, TN, EM, 13.iv.2014 (HNHM 98982); 1.5 km S of Steblevë, along the Librazhd to Peshkopi road, 1220 m, 41.3250°N, 20.4457°E, leg. ZF, TN, EM, 13.iv.2014 (HNHM 98980); same locality, leg. AR, NR, PR, ix.2013 (NHMW 110430/MN/0082); 1 km S of Steblevë, 1250 m, 41.3239°N, 20.4511°E, leg. ZE, ZF, JK, DM, 24.x.2002 (HNHM 94885); 8.5 km N of Steblevë, leg. AR, NR, PR, ix.2013 (NHMW 110430/MN/0083); rocky grassland between Klenjë and Steblevë, 1190 m, 41.3575°N, 20.4623°E, leg. PJ, TK, Magos, GP, 31.v.2013 (HNHM 99623); Bulqizë District, W of Zabzun, 4 km from the Librazhd to Peshkopi road toward Sebisht, 1230 m, 41.3462°N, 20.3904°E, leg. ZF, 13.iv.2014 (HNHM 98977); Tuçep, 1.5 km N of Ostreni i Vogël, 700 m, 41.4404°N, 20.5052°E, leg. ZF, TN, EM, 14.iv.2014 (HNHM 98987); same locality, leg. ZE, ZF, JK, DM, 25.x.2002 (HNHM 91915); 1.2 km S of Ostreni i Madh, 870 m, 41.4199°N, 20.4633°E, leg. ZF, TN, EM, 14.iv.2014 (HNHM 98986); same locality, leg. ZE, ZF, JK, DM, 25.x.2002 (HNHM 94900); 1.5 km N of Klenjë, along the Librazhd to Peshkopi road, 1220 m, 41.3792°N, 20.4687°E, leg. ZF, TN, EM, 13.iv.2014 (HNHM 98984); 16.3 km N of Steblevë, leg. AR, NR, PR, ix.2013 (NHMW 110430/MN/0084); Librazhd District, W of Funarës, 580 m, 41.2719°N, 20.2839°E, leg. ZF, TN, EM, 12.iv.2014 (HNHM 98953); 3.5 km W of Funarës, 660 m, 41.2748°N, 20.2679°E, leg. ZE, ZF, AH, DM, 11.iv.2006 (HNHM 96814); same locality, leg. ZF, TN, EM, 12.iv.2014 (HNHM 98954).

##### Distribution.

Gollobordë and Çermenikë Plateaus in eastern central Albania (Fig. 25).

##### Remarks.

Shell sizes show unusually large variability between the different localities. They are smallest at the type locality, but at other sites can be as large as those of the closely related *Montenegrina perstriata tenebrosa* Nordsieck, 2009.

#### Montenegrina perstriata diminuta

Taxon classificationAnimaliaStylommatophoraClausiliidae

Fehér & Szekeres, 1999

Fig. 27C

Montenegrina perstriata diminuta Fehér & Szekeres, 1999 in Erőss et al. 1999: 446–448, fig. 2. – Nordsieck 2009: 74, plate 3, fig. 12.

##### Diagnosis.

Shell medium, slender, light corneous. Lower whorls flattened, nearly of the same width. All whorls indistinctly wrinkled-costate, more prominently toward the apex. Neck densely costate. Peristome with simple margin. Lamellae superior and spiralis mostly overlap. In front view lamella subcolumellaris not visible. Lunella lateral to ventrolateral, not connected to the basalis. Subclaustralis residual, sulcalis present. Anterior plica superior absent or weak, not connected to the lunella complex. Clausilium plate not visible through the aperture.

##### Dimensions

(in mm). H_s_: 14.0–18.5 (holotype 17.4), W_s_: 3.0–3.8 (holotype 3.7).

##### Type locality.

Macedonia, Galičica Mts, 8 km NW of the Bukovo Pass (= 1 km E of the Zavoj junction) on the Ohrid to Resen road, 950 m, 41.1978°N, 20.9173°E.

##### Type material.

Type locality, leg. ZE, ZF, 11.viii.1996, holotype (HNHM 70832), paratypes (HNHM 70833/7, NHMW 100075/2, SMF 312510/2, NMBE 535061/1, NMBE 22593/2).

##### Other material.

Type locality, leg. ZE, ZF, AH, 7.iv.2004 (HNHM 94431); same locality, leg. ZF, EH, KJ, HS, 15.x.2014 (NHMW 110430/MN/0102); 2.2 km toward Rečica from the Ohrid to Resen road, 940 m, 41.2202°N, 20.9115°E, leg. ZF, EH, KJ, HS, 15.x.2014 (NHMW 110430/MN/0101); same locality, leg. ZE, ZF, JG, 30.vi.2015 (NHMW 110430/MN/0111).

##### Distribution.

Northern extensions of the Galičica Mts (Fig. 25).

#### Montenegrina perstriata drimica

Taxon classificationAnimaliaStylommatophoraClausiliidae

Nordsieck, 1972

Fig. 27D, E

Montenegrina perstriata drimica Nordsieck, 1972: 32, plate 4, fig. 38. – Zilch 1981: 130, plate 14, fig. 31. – Nordsieck 2009: 74, plate 1, fig. 3. – Dedov and Neubert 2009:91, plate 1, figs 3–4.
Montenegrina perstriata crassa Erőss & Szekeres, 1999 in Erőss et al. 1999: 448, fig. 3. – Nordsieck 2009: 75.

##### Diagnosis.

Shell small to large, tumid, light to dark corneous. Lower whorls smooth, flattened, nearly of the same width. Upper whorls finely striate-costate. Neck densely striate-costate. Peristome with weakly to very strongly swollen, reflexed margin. Lamellae superior and spiralis overlap. In front view lamella subcolumellaris often visible. Lunella lateral, separate from the weak basalis. Subclaustralis short, sulcalis present. Anterior plica superior absent or weak, separate from the lunella complex. Clausilium plate not visible through the aperture.

##### Dimensions

(in mm). H_s_: 14.0–21.5 (holotype 16.5), W_s_: 3.8–5.0 (holotype 4.3).

##### Type locality.

Macedonia, valley of the Crni Drin, Lukovo near Struga [*drimica*]; Macedonia, valley of the Crni Drin, left side, 2 km N of Lukovo and 1 km S of the road junction to Modrić, 41.3654°N, 20.6048°E [*crassa*].

##### Type material.

Lukovo near Struga, leg. H. Nordsieck, 27.viii.1971, holotype [*drimica*] (SMF 221048), paratypes [*drimica*] (SMF 221049, SMNS-N 5500/30); same locality, leg. H. Nordsieck, 19.viii.1971, paratype [*drimica*] (SMNS-N5596); 2 km N of Lukovo, leg. ZE, ZF, 13.viii.1996, holotype [*crassa*] (HNHM 70834), paratypes [*crassa*] (HNHM 70835/6, NHMW 100076/2, SMF 312509/2, NMBE 535059/1, NMBE 22592/3).

##### Other material.

Macedonia, Debar District, S of Debar, at the dam of the Crni Drin, 590 m, 41.4948°N, 20.5052°E, leg. ZF, EH, KJ, HS, 14.x.2014 (NHMW 110430/MN/0087); between Lukovo and Džepište, leg. Sipos, AS, Topál, 13.x.1975 (HNHM 10139/35); 1 km SE of Džepište, leg. LP, PS, AS, 17.vii.1972 (HNHM 36712); Struga District, valley of the Crni Drin, 3.7 km S of Lukovo, 700 m, 41.3299°N, 20.6369°E, leg. ZF, EH, KJ, HS, 15.x.2014 (NHMW 110430/MN/0088); same locality, leg. ZE, ZF, AH, 8.iv.2004 (HNHM 94448); 3 km S of Lukovo, Brana Globočica, 41.3355°N, 20.6345°E, leg. ZE, ZF, AH, 8.iv.2004 (HNHM 94447); 2 km S of Lukovo, S of the viaduct, 690 m, 41.3397°N, 20.6273°E, leg. ZF, EH, KJ, HS, 15.x.2014 (NHMW 110430/MN/0089); same locality, N of the viaduct, 690 m, 41.3406°N, 20.6266°E, leg. ZF, EH, KJ, HS, 15.x.2014 (NHMW 110430/MN/0090); same locality, leg. ZE, ZF, AH, 8.iv.2004 (HNHM 94446); Lukovo, S edge of the village, 640 m, 41.3545°N, 20.6124°E, leg. ZF, EH, KJ, HS, 15.x.2014 (NHMW 110430/MN/0091); same locality, leg. ZE, ZF, AH, 8.iv.2004 (HNHM 94445); 1 km N of Lukovo, 630 m, 41.3623°N, 20.6036°E, leg. ZF, EH, KJ, HS, 15.x.2014 (NHMW 110430/MN/0092); 1.3 km N of Lukovo, 620 m, 41.3638°N, 20.6049°E, leg. ZF, EH, KJ, HS, 15.x.2014 (NHMW 110430/MN/0093); 1.5 km N of Lukovo, 620 m, 41.3666°N, 20.6049°E, leg. ZF, EH, KJ, HS, 15.x.2014 (NHMW 110430/MN/0094); same locality, leg. ZE, ZF, AH, 8.iv.2004 (HNHM 94449); 1.7 km N of Lukovo, 620 m, 41.3672°N, 20.6046°E, leg. ZF, EH, KJ, HS, 15.x.2014 (NHMW 110430/MN/0095); 2 km N of Lukovo, 620 m, 41.3695°N, 20.6021°E, leg. ZF, EH, KJ, HS, 15.x.2014 (NHMW 110430/MN/0096); same locality, leg. ZE, ZF, AH, 8.iv.2004 (HNHM 94589); 2.4 km N of Lukovo, S of the Modrić junction, 610 m, 41.3705°N, 20.5979°E, leg. ZF, EH, KJ, HS, 15.x.2014 (NHMW 110430/MN/0097); 2.5 km N of Lukovo, at the Modrić junction, 610 m, 41.3713°N, 20.5971°E, leg. ZF, EH, KJ, HS, 15.x.2014 (NHMW 110430/MN/0098); 6 km N of Lukovo, side road to Lokov, 620 m, 41.3978°N, 20.5899°E, leg. ZF, EH, KJ, HS, 15.x.2014 (NHMW 110430/MN/0099); 4 km N of Lukovo, 600 m, 41.3816°N, 20.5881°E, leg. ZF, EH, KJ, HS, 15.x.2014 (NHMW 110430/MN/0100).

##### Distribution.

Upper valley of the Crni Drin between the Globočica Lake and Debar (Fig. 25).

##### Remarks.

This taxon shows considerable intra- and inter-population heterogeneity in terms of shell size and peristome thickness. Nordsieck (2009) questioned the distinct subspecific status of *crassa*, which he regarded “a local form of *Montenegrina perstriata drimica* with thickened peristome, anterior upper palatal plica continuous with upper palatal plica…”. He argued that “there are further forms of *Montenegrina perstriata drimica* with thickened peristome occurring farther in the south” (Nordsieck 1972: 32–33). Based on these arguments, Dedov and Neubert (2009) already treated *crassa* as the junior synonym of *drimica*, and we also accept this view.

#### Montenegrina perstriata mavrovoensis

Taxon classificationAnimaliaStylommatophoraClausiliidae

Nordsieck, 2009

Fig. 27F

Montenegrina perstriata mavrovoensis Nordsieck, 2009: 78, plate 3, fig. 11.

##### Diagnosis.

Shell medium, elongate, light corneous. Lower whorls nearly of the same width, almost smooth to finely, indistinctly costate. Upper whorls indistinctly wrinkled-costate. Neck costate. Peristome with simple margin. Lamellae superior and spiralis mostly overlap. Lamella subcolumellaris deep, even in oblique view barely visible. Lunella dorsolateral to lateral, not connected to the weak basalis. Subclaustralis residual, sulcalis present. Anterior plica superior absent or weak and separate from the lunella complex. Clausilium plate mostly not visible through the aperture.

##### Dimensions

(in mm). H_s_: 15.4–18.4 (holotype 17.7), W_s_: 3.9–4.5 (holotype 4.3).

##### Type locality.

Macedonia, Bistra Mts, Mavrovo 2 km towards Galičnik.

##### Type material.

Type locality, leg. H. Nordsieck, 4.viii.1974, holotype (SMNS 70545), paratypes (SMNS-N 6922/15, SMF320048/8).

##### Distribution.

Bistra Mts in western Macedonia. Known only from the type locality (Fig. 25).

##### Remarks.

Nordsieck (1977) noticed the peculiarity of this taxon and mentioned it as ‘a morph standing close to *subcristatula*’. Later, in the description, he suggested that it is probably closer to *Montenegrina perstriata diminuta* (Nordsieck 2009).

#### Montenegrina perstriata occidentalis

Taxon classificationAnimaliaStylommatophoraClausiliidae

Nordsieck, 1977

Fig. 27G

Montenegrina dofleini occidentalis Nordsieck, 1977: 85, plate 4, fig. 15. – Zilch 1981: 127, plate 15, fig. 38. – Nordsieck 2009: 74.

##### Diagnosis.

Shell small to medium, elongate, light corneous. Lower whorls flattened, nearly of the same width. Surface costate, ribs sharper and wider spaced over the upper whorls and the neck. Peristome rounded to angular, with somewhat swollen margin. Lamellae superior and spiralis long overlap. In front view lamella subcolumellaris not visible. Lunella dorsolateral to lateral, separate from the weak basalis. Subclaustralis residual, sulcalis strong. Anterior plica superior mostly absent, if present separate from the lunella complex. Clausilium plate occasionally visible through the aperture.

##### Dimensions

(in mm). H_s_: 12.8–17.8 (holotype 16.7), W_s_: 3.6–4.4 (holotype 4.1).

##### Type locality.

Macedonia, Kališta near Struga.

##### Type material.

Type locality, leg. Nordsieck, 5.viii.1974, holotype (SMF 237804), paratypes (SMF 237805, SMF 320060/15, SMNS-N 6937, NMBE 534974/2); type locality, leg. Nordsieck, 26.viii.1976, paratypes (ZMH 91617/6).

##### Other material.

Macedonia, Kališta, orthodox cemetery, 710 m, 41.1493°N, 20.6493°E, leg. ZE, ZF, AH, 7.iv.2004 (HNHM 94441); Albania, Pogradec District, Lin, 710 m, 41.0693°N, 20.6468°E, leg. ZE, ZF, JG, 30.vi.2013 (HNHM 99615); same locality, leg. ZE, ZF, JK, DM, 23.x.2003 (HNHM 94903).

##### Distribution.

Western shore of the Ohrid Lake. Known only from two localities (Fig. 25). A third record, mentioned in the original description (Radožda near Struga), was later reconsidered as corresponding to *Montenegrina perstriata plenostoma* Fehér & Szekeres, 2006 (Nordsieck 1977, 2009).

#### Montenegrina perstriata ochridensis

Taxon classificationAnimaliaStylommatophoraClausiliidae

(Wagner, 1925)

Fig. 27H

Delima (Albanodelima) perstriata ochridensis – Wagner 1924: 120. (*nomen nudum*).
Delima (Delima) perstriata ochridensis Wagner, 1925: 62, plate 14, figs 98a–c.
Delima (Montenegrina) perstriata ochridensis – Loosjes 1966: 122, fig. 1. (genital anatomy).
Montenegrina perstriata ochridensis – Nordsieck 1972: 32–33, plate 4, fig. 39. – Zilch 1981: 130. – Erőss et al. 1999: 448. – Nordsieck 2009: 74.
Montenegrina ochridensis – Nordsieck 1988: 201.

##### Diagnosis.

Shell medium, light corneous. Lower whorls smooth to very densely striate, upper ones densely costate. Neck densely costate. Peristome angular, with swollen, reflexed margin. Lamellae superior and spiralis do not overlap. Subcolumellaris not or only barely, clausilium plate not at all visible through the aperture. Lunella lateral, fused to the basalis. Sulcalis absent or residual. Anterior plica superior separate from the lunella complex.

##### Dimensions

(in mm). H_s_: 16.8–20.7, W_s_: 4.4–5.1 (Nordsieck 1972).

##### Type locality.

Macedonia, Ohrid.

##### Type material.

Syntypes are thought to be in the MIZPAS (not seen).

##### Other material.

Macedonia, Ohrid, Izvor Studeničiśta, 700 m, 41.1030°N, 20.8143°E, leg. ZE, ZF, AH, 7.iv.2004 (HNHM 94428); Ohrid, ex Kuščer (NHMW-E 48139); Ohrid, Biological Station, leg. Kaiser, viii.1958 (NHMW-K 44011, SMF 167047 [Nordsieck 1972: fig. 37]); Ohrid District, Šipokno, Hotel Gorica, 730 m, 41.0843°N, 20.7973°E, leg. ZF, EH, KJ, HS, 15.x.2014 (NHMW 110430/MN/0079); Gradište, Zaliv na Koskite (“Bay of the Bones”), 700 m, 40.9937°N, 20.7992°E, leg. ZF, EH, KJ, HS, 16.x.2014 (NHMW 110430/MN/0080); Peštani, 41.0092°N, 20.8056°E, leg. ZF, 17.x.2014 (NHMW 110430/MN/0081); gorge S of Peštani, 760 m, 41.0087°N, 20.8092°E, leg. ZE, ZF, AH, 6.iv.2004 (HNHM 94434); shore of the Lake Ohrid ca. 2.5 km S of Peštani, 40.999°N, 20.802°E, leg. LP, PS, AS, 16.vii.1972 (HNHM 36714); Sveti Stefan, 6 km S of Ohrid, leg. H. Nordsieck, 26.viii.1971 (SMNS-N 5491). Albania, Bulqizë District, Zerqan, Tre Çesmë Spring, 570 m, 41.5160°N, 20.3911°E, leg. ZE, ZF, JK, DM, 25.x.2002 (HNHM 94888); same locality, leg. ZF, TN, EM, 15.iv.2014 (HNHM 99006); Valikardhë, S slope of the Maja e Temlishit, 770 m, 41.5140°N, 20.3160°E, leg. ZF, TN, EM, 15.iv.2014 (HNHM 99007).

##### Distribution.

Originally this taxon was known only from the eastern coastline of the Ohrid Lake, between Ohrid and Gradište (see: Brandt 1962; Loosjes 1966). Recently it was also found in Central Albania, east of Bulqizë (Fig. 25). On the Mt. Temlishi it lives syntopically with *Montenegrina nana barinai* ssp. n.

##### Remarks.

First *ochridensis* was regarded by Nordsieck (1972) a subspecies of *Montenegrina perstriata* but later, pointing out the differences in shell morphology, he classified it as a distinct species (Nordsieck 1988). More recently he again mentioned this taxon as *Montenegrina perstriata ochridensis* (Nordsieck 2009).

In the original description the subgenus name *Delima* is likely a typographic error (Wagner 1925: 70), because earlier and in the plate caption of the same paper he used *Albanodelima* (Wagner 1924, 1925).

#### Montenegrina perstriata plenostoma

Taxon classificationAnimaliaStylommatophoraClausiliidae

Fehér & Szekeres, 2006

Fig. 27I

Montenegrina dofleini plenostoma Fehér & Szekeres, 2006 in Erőss et al. 2006: 188, fig. 7. – Nordsieck 2009: 74.

##### Diagnosis.

Shell medium to large, slender, light corneous. Lower whorls flattened, nearly of the same width. Surface costate, ribs shaper and denser over the upper whorls and the neck. Peristome rounded to angular, with strongly swollen, reflexed margin. Lamellae superior and spiralis overlap. In front view lamella subcolumellaris mostly visible. Lunella dorsolateral, separate from the basalis. Subclaustralis short, sulcalis strong. Anterior plica superior well developed, often connected to the lunella complex. Clausilium plate occasionally visible through the aperture.

##### Dimensions

(in mm). H_s_: 14.8–18.9 (holotype 17.6), W_s_: 3.7–4.4 (4.0).

##### Type locality.

Albania, Pogradec District, 2 km E of the Qafa e Thanës, along the Librazhd to Pogradec road, 860 m, 41.0632°N, 20.6253°E.

##### Type material.

Type locality, leg. ZE, ZF, JK, DM, 23.x.2002, holotype (HNHM 94840), paratypes (NHMUK 20050217, HNC 63181, HNHM 94841/20, NHMW 103275, RMNH 100308, SMF 328079); 1 km E of Qafa e Thanës, leg. ZF, 23.vii.1993, paratypes (HNHM 90882/29).

##### Other material.

Type locality, leg. ZF, TN, EM, 12.iv.2014 (HNHM 98945); 1 km N of Pishkupat, 700 m, 41.0402°N, 20.6346°E, leg. ZE, ZF, JK, DM, 30.vi.2003 (HNHM 94843); Macedonia, Radožda near Struga, leg. H. Nordsieck, 6.viii.1974 (SMF 320061).

##### Distribution.

West of the Ohrid Lake (see Erőss et al. 2006), sympatric but not syntopic with *Montenegrina dofleini occidentalis* (Fig. 25).

#### Montenegrina perstriata radikae

Taxon classificationAnimaliaStylommatophoraClausiliidae

Nordsieck, 1972

Fig. 27J

Montenegrina perstriata radikae Nordsieck, 1972: 32, plate 4, fig. 37. – Zilch 1981: 130, plate 14, fig. 32. – Nordsieck 2009: 74.

##### Diagnosis.

Shell medium to large, tumid, light corneous. All whorls smooth, lower ones nearly of the same width. Neck wrinkled-costate. Basal crest stronger than the peripheral. Peristome with swollen margin. Lamella superior weak, distant from the spiralis. In front view lamella subcolumellaris not or only barely visible. Lunella lateral, fused to the short basalis. Subclaustralis and sulcalis residual. Anterior plica superior absent or short and mostly fused to the lunella complex. Clausilium plate not visible through the aperture.

##### Dimensions

(in mm). H_s_: 17.8–23.1 (holotype 19.7), W_s_: 4.6–5.5 (holotype 4.9).

##### Type locality.

Macedonia, Radika gorge near Debar, at the bridge.

##### Type material.

Type locality, leg. H. Nordsieck, 29.viii.1971, holotype (SMF 221050), paratypes (221051/1, SMNS-N 5510, SMNS-N 5511, NHMW-K 65045/7).

##### Other material.

Macedonia, Mavrovo District, Radika gorge, bridge 1 km N of the Sence junction along the Debar to Gostivar road, 880 m, 41.6994°N, 20.6478°E, leg. ZF, EH, KJ, HS, 14.x.2014 (NHMW 110430/MN/0086); same locality, leg. ZE, ZF, AH, 8.iv.2004 (HNHM 94917); Radika valley, 6 km from Trnica toward Debar, leg. LP, PS, AS, 12.vii.1972 (HNHM 31107).

##### Distribution.

Radika Valley in western Macedonia. Known to occur within a small range. All known occurrences are in the immediate vicinity of the type locality. A few kilometers farther upstream in the Radika Valley this taxon is replaced by *Montenegrina perstriata subcristatula* Nordsieck, 1977 (Fig. 25).

#### Montenegrina perstriata steffeki

Taxon classificationAnimaliaStylommatophoraClausiliidae

Erőss & Szekeres, 2006

Fig. 27K

Montenegrina perstriata steffeki Erőss & Szekeres, 2006 in Erőss et al. 2006: 203, fig. 25.
Montenegrina dofleini steffeki – Nordsieck 2009: 74.

##### Diagnosis.

Shell medium to large, tumid and somewhat conical, light corneous. Lower whorls almost smooth to variably wrinkled, upper ones indistinctly wrinkled-costate. Neck strongly, irregularly costate. Relatively large peristome with simple margin. Lamellae superior and spiralis overlap. In front view lamella subcolumellaris occasionally visible. Lunella dorsal-dorsolateral to dorsolateral, mostly separate from the basalis. Subclaustralis short, sulcalis strong. Anterior plica superior weak, not connected to the lunella complex. Clausilium plate partly visible through the aperture.

##### Dimensions

(in mm). H_s_: 14.9–21.7 (holotype 17.7), W_s_: 3.9–5.4 (holotype 4.9).

##### Type locality.

Macedonia, valley of the Crni Drin, gorge above and W of Jablanica, about 25 km NNW of Struga.

##### Type material.

Type locality, leg. Šteffek, 28.vii.1985, holotype (HNHM 94891), paratypes (NHMUK 20050230, HNC 63194, HNHM 94892/2, NHMW 103292, RMNH 100321, SMF 328096).

##### Distribution.

Upper valley of the Crni Drin Valley in western Macedonia. The subspecies was collected only once at the type locality. Due to the vague locality data, during later attempts the site could not be found.

##### Remarks.

In Nordsieck’s (2009) opinion this taxon is closely related to “*Montenegrina dofleini occidentalis*”.

#### Montenegrina perstriata subcristatula

Taxon classificationAnimaliaStylommatophoraClausiliidae

Nordsieck, 1977

Fig. 27L

Montenegrina perstriata perstriata – Nordsieck 1972: 31, plate 4, fig. 36.
Montenegrina perstriata subcristatula Nordsieck, 1977: 84, plate 4, fig. 14. – Zilch 1981: 130, plate 14, fig. 33. – Nordsieck 2009: 74, plate 3, fig. 13.

##### Diagnosis.

Shell medium, slender, light corneous. Lower whorls smooth, flattened, nearly of the same width. Upper whorls indistinctly wrinkled-costate. Neck strongly wrinkled-costate. Peristome with simple margin. Lamellae superior and spiralis overlap. In front view lamella subcolumellaris often visible. Lunella lateral, mostly connected to the basalis. Subclaustralis as long as the basalis, sulcalis present. Anterior plica superior absent or very weak and separate from the lunella complex.

##### Dimensions

(in mm). H_s_: 15.6–18.6 (holotype 18.6), W_s_: 3.8–4.2 (holotype 4.2) (Nordsieck 1972, 1977).

##### Type locality.

Macedonia, gorge of the Radika near Debar, 1.5 km upstream of the bridge.

##### Type material.

Type locality, leg. Nordsieck, 28.viii.1971, holotype (SMF 221318), paratypes (SMNS-N 5509, ZMH 91590/8); Radika gorge above Ničpur junction, leg. Nordsieck, 5.viii.1974, paratype status doubtful (SMNS-N 6933, SMF 247211/5).

##### Other material.

Macedonia, Gostivar District, upper valley of the Radika, 6 km from the Debar to Gostivar road toward Ničpur, 1100 m, 41.7655°N, 20.6666°E, leg. ZF, EH, KJ, HS, 14.x.2014 (NHMW 110430/MN/0108); Mavrovo District, Radika Valley, along the Debar to Gostivar road at the Ničpur junction, 930 m, 41.7203°N, 20.6682°E, leg. ZF, EH, KJ, HS, 14.x.2014 (NHMW 110430/MN/0107); same locality, leg. ZE, ZF, AH, 8.iv.2004 (HNHM 94919); 500 m E of the Nistrovo junction along the Debar to Gostivar road, 900 m, 41.7102°N, 20.6564°E, leg. ZF, EH, KJ, HS, 14.x.2014 (NHMW 110430/MN/0109); at the Nistrovo junction, 890 m, 41.7079°N, 20.6500°E, leg. ZF, EH, KJ, HS, 14.x.2014 (NHMW 110430/MN/0110); same locality, leg. ZE, ZF, AH, 8.iv.2004 (HNHM 94918).

##### Distribution.

Valley of the Radika in western Macedonia. This taxon is known to occur within a few km section of the valley. Downstream it is replaced by *Montenegrina perstriata radikae* (Fig. 25).

##### Remarks.

In the description, Nordsieck (1972) mentions material from from three sites, without specifying which of these are designated as paratypes. Sample SMF 247211 is labelled as paratypes and Zilch (1981) lists it accordingly. But SMNS-N 6933, the other part of the same lot, is without indication of type status.

#### Montenegrina perstriata tenebrosa

Taxon classificationAnimaliaStylommatophoraClausiliidae

Nordsieck, 2009

Fig. 27M

Montenegrina perstriata – Dhora and Welter-Schultes 1999: 16.
Montenegrina perstriata tenebrosa Nordsieck, 2009: 78–79, plate 3, fig. 14.

##### Diagnosis.

Shell medium to large, dark corneous. Lower whorls smooth, upper ones very finely and densely striate. Neck densely striate. Peristome rounded to angular, with strongly swollen, reflexed margin. Lamellae superior and spiralis mostly do not overlap. Subcolumellaris mostly not, clausilium plate not visible through the aperture. Lunella dosolateral-lateral, fused to the basalis. Sulcalis well developed. Anterior plica superior frequently connected to the lunella complex.

##### Dimensions

(in mm). H_s_: 18.3–21.4 (holotype 20.9), W_s_: 4.75–5.6 (holotype 5.3).

##### Type locality.

Albania, Mali i Gurit, Qafa e Zylit, 1800 m.

##### Type material.

Type locality, leg. Dhora, 16.ix.1996, holotype (SMNS 70546), paratypes (SMNS-N 10698/4, SMF 328780/3).

##### Other material.

Albania, Bulqizë District, Maja a Zyllit (= Maja e Shullanit), 6 km from the Librazhd to Peshkopi road toward Sebisht, 1360 m, 41.3611°N, 20.3967°E, leg. ZF, 13.iv.2014 (HNHM 98975).

##### Distribution.

Gollobordë Plateau in northeastern Albania. The precise location of the type locality is unknown, but the material from the road to Sebisht is thought to be from its vicinity (Fig. 25).

##### Remarks.

Nordsieck (2009) noted that *Montenegrina perstriata tenebrosa* is close to the ‘*crassa*’ mentioned by Fehér and Erőss (2009) from central Albania. Based on a large set of recently obtained samlpes we are of the opinion that the form mentined as *crassa* (Fehér and Erőss 2009) belongs to *Montenegrina perstriata callistoma*. We regard *Montenegrina perstriata tenebrosa* an extremely large member of this heterogeneous group, but maintain its status as a separate subspecies.

#### Montenegrina prokletiana sp. n.

Taxon classificationAnimaliaStylommatophoraClausiliidae

http://zoobank.org/3223E4A9-37AB-4ECD-B4F2-FDA88CC135B9

##### Diagnosis.

Shell very small to small, elongate, light corneous. Whorls flat, smooth to indistinctly wrinkled, lower whorls nearly of same width. Neck costate, its strong inflection extends to the lateral side. Basal crest weak, peripheral crest strong. Peristome attached, ovoid to somewhat angular, with more or less swollen margin. Lamellae superior and spiralis overlap. In front view lamella inferior moderately, medium-bent subcolumellaris strongly emerged. Lunella dosal-dorsolateral to dorsolateral, separate from the basalis. Subclaustralis of variable strength, sulcalis present. Anterior plica superior mostly absent. Clausilium plate not or only barely visible through the aperture. Differs fom all other species of the genus by its very prominent, horizontally ending lamella subcolumellaris.

#### Montenegrina prokletiana prokletiana ssp. n.

Taxon classificationAnimaliaStylommatophoraClausiliidae

http://zoobank.org/0B60F163-FD4B-496B-AFA1-872F9409C4C5

Fig. 24F

##### Diagnosis.

Very small, slender subspecies with strongly inflexed neck, weak basalis, but long subclaustralis.

##### Description.

The light brown shell is very small and slender, its 8½ to 10½ whorls are separated by a deep suture. The lower three whorls are almost of the same width. Except the finely costate neck the entire shell surface is smooth. The strong inflection of the neck extends to the ventral side. The basal crest is weak, the peripheral one is strong. The light brown peristome is inflexed at the parietal side. The swollen and deflexed peristome margin is absent at the upper columellar side. The strong, projected lamella superior barely overlaps with the spiralis. The lamella inferior is weakly emerged, it turns horizontal before ending. In front view the medium-bent subcolumellaris is visible. Neither the inferior, nor the subcolumellaris reach the peristome margin. The dorsolateral lunella is not connected to the residual basalis. The subclaustralis is long and well developed, the sulcalis is present. The anterior plica superior is missing. The clausilium plate is not or only barely visible through the aperture.

##### Dimensions

(in mm). Holotype H_s_: 10.8, W_s_: 2.7, H_a_: 2.6, W_a_: 2.1; paratypes (HNHM 99491, n = 12): H_s_: 9.1–11.0 (mean 9.9, S.D. 0.66), W_s_: 2.4–2.8 (mean 2.6, S.D. 0.12), H_a_: 2.3–2.7, W_a_: 1.9–2.2.

##### Differential diagnosis.

The new subspecies differs from *Montenegrina prokletiana kovacsorum* ssp. n. by its smaller size, non-marginal lamellae inferior and subcolumellaris, as well as its well developed subclaustralis.

##### Type locality.

Albania, Tropojë District, Dragobi (14 km N of Bajram Curri), gorge of the Përroi i Thatë, 540 m, 42.4364°N, 19.9846°E.

##### Type material.

Type locality, leg. TD, ZE, ZF, DM, 6.x.2005, holotype (HNHM 99490), paratypes (HNHM 99491/3a+24, NHMW 111223/5a, MMM-B01326/25, ER/24, SZ/4); same locality, leg. ZB, Somogyi, 12.viii.2012, paratypes (HNHM 99492/13a+3aj).

##### Etymology.

The new taxon is named after the Prokletije Mts, the area where this species is distributed.

##### Distribution.

Northern Albania, Valbona Valley in the southern part of the Prokletije Mts. Known only from the type locality (Fig. 26).

#### Montenegrina prokletiana kovacsorum ssp. n.

Taxon classificationAnimaliaStylommatophoraClausiliidae

http://zoobank.org/B90D1DFC-978D-4C40-A920-4B070662DCE9

Fig. 24G

##### Diagnosis.

Small, very slender subspecies with strongly inflexed neck and emerged, almost marginal, swollen end of the lamella subcolumellaris.

##### Description.

The small, very slender, light brown shell consists of 8½ to 10½ whorls, which are separated by a deep suture. The lower three whorls are almost of the same width. The surface is smooth over the entire shell except the neck, which is strongly wrinkled-costate from the dorsal side. The neck inflection is very strong and extends to the ventral side. The basal crest is weak, the peripheral one is strong. The light brown peristome is inflexed at the parietal side. The peristome margin is narrow, swollen and deflexed, but it is entirely absent over the upper columellar side. The strong lamella superior emerges from the plane of the peristome. Inward it does not overlap the spiralis. The lamella inferior is emerged, its end abruptly turns toward the aperture. Both the inferior and the medium-bent subcolumellaris terminate in thick, horizontal plicae at the peristome margin. The dorsal-dorsolateral to dorsolateral lunella is separate from the basalis. The subclaustralis is absent, the sulcalis is present. The anterior part of the plica superior is mostly missing, rarely a weak, separately standing residue of it is visible. The clausilium plate is not or only barely visible through the aperture.

##### Dimensions

(in mm). Holotype H_s_: 15.5, W_s_: 3.5, H_a_: 3.5, W_a_: 2.7; paratypes (HNHM 99486, n = 12) H_s_: 10.7–16.2 (mean 13.8, S.D. 1.59), W_s_: 2.5–3.5 (mean 3.1, S.D. 0.30), H_a_: 2.7–3.5, W_a_: 1.9–2.8.

##### Differential diagnosis.

Differs from *Montenegrina prokletiana prokletiana* ssp. n. by the larger size, marginally ending lamellae inferior and subcolumellaris, and the absence of a subclaustralis.

##### Type locality.

Albania, Shkodër District, Toplanë, Drin Valley 20.5 km upstream of the Koman Dam, limestone gorge and a cave on the right bank of Koman Lake, 180 m, 42.2339°N, 19.8741°E.

##### Type material.

Type locality, leg. ZF, TK, DM, 18.vi.2012, holotype (HNHM 99485), paratypes (HNHM 99486/26+17a+4aj, NHMW 111221/11, SZ/2).

##### Other material.

Albania, Drin Valley ca. 17.5 km upstream of the Koman Dam, right bank, 42.2258°N, 19.8924°E, leg. ZE, ZF, AH, DM, 15.iv.2006 (HNHM 99487); ca. 18 km upstream of the Koman Dam, left side-valley, 170 m, 42.2269°N, 19.9050°E, leg. ZE, ZF, AH, DM, 15.iv.2006 (HNHM 99488); Tropojë District, 3 km W of the Lumi i Valbonës mouth along the Dushaj to Tetaj road, 240 m, 42.2623°N, 19.9911°E, leg. TD, ZE, ZF, DM, 7.x.2005 (HNHM 97074, MMM-B01325); same locality, leg. DA, ZE, ZF, JG, 26.vi.2014 (HNHM 99258, NHMW 111222).

##### Etymology.

The new taxon is named after Tibor Kovács, entomologist (HNHM), and Kornél Kovács, malacologist, both participants of several field trips to the Balkans.

##### Distribution.

Lower Drin Valley in northern Albania. Known to occur upstream of the Koman Dam up to the mouth of the Valbonë (Fig. 26). Along this section of the river we found *Montenegrina* shells in fluvial flotsam, which likely belong to this subspecies, but their poor condition does not allow unambiguous identification.

##### Remarks.

The specimens near the mouth of the Valbonë are usually smaller (H_s_: 9.0–12.0 mm) with fewer (8–9) whorls. Some flotsam specimens have strongly wrinkled surface, but otherwise very similar shell structure.

#### Montenegrina rugilabris

Taxon classificationAnimaliaStylommatophoraClausiliidae

(Mousson, 1859)

##### Diagnosis.

Shell medium to large, yellowish- to purplish-brown. Lower whorls mostly smooth, upper ones smooth to indistinctly costate. Neck not or weakly inflexed. Basal crest well recognizable, peripheral crest weak. Peristome rounded to ovoid, with wide, swollen margin. Lamellae superior and spiralis mostly overlap. In front view lamella inferior variably emerged, broadly-bent subcolumellaris usually visible. Lunella dorsal to dorsal-dorsolateral, fused to or separate from the basalis. Lunella complex with the basalis can form a lambda-like structure. Subclaustralis always shorter than the basalis, sulcalis strong. Anterior plica superior fused to or separate from the lunella complex. Clausilium plate well visible through the aperture. Distinguishable from the similar *Montenegrina skipetarica* by the shorter subclaustralis and well developed sulcalis.

#### Montenegrina rugilabris rugilabris

Taxon classificationAnimaliaStylommatophoraClausiliidae

(Mousson, 1859)

Fig. 28A

Clausilia rugilabris Mousson, 1859: 275–276.
Clausilia (Delima) rugilabris – Westerlund 1884: 53.
Delima (Albanodelima) rugilabris – Wagner 1924: 120.
Montenegrina rugilabris – Zilch 1981: 130, plate 14, fig. 34. – Nordsieck 2009: 74.

##### Diagnosis.

Shell medium, tumid, yellowish-brown. Lower whorls smooth, upper ones very finely, indistinctly costate. Neck not to weakly inflexed, finely and densely costate. Basal crest well recognizable, peripheral crest weak. Peristome attached, with strongly swollen, reflexed margin. Lamellae superior and spiralis overlap. In front view lamella inferior moderately emerged, broadly-bent subcolumellaris visible. Lunella dorsal, separate from the short basalis. Subclaustralis absent or residual. Anterior plica superior often connected to the lunella complex. Clausilium plate almost entirely visible through the aperture.

##### Dimensions

(in mm). H_s_: 16.5–20.4, W_s_: 5.1–6.3 (near Ligkiades junction HNHM 99549).

**Figure 28.**
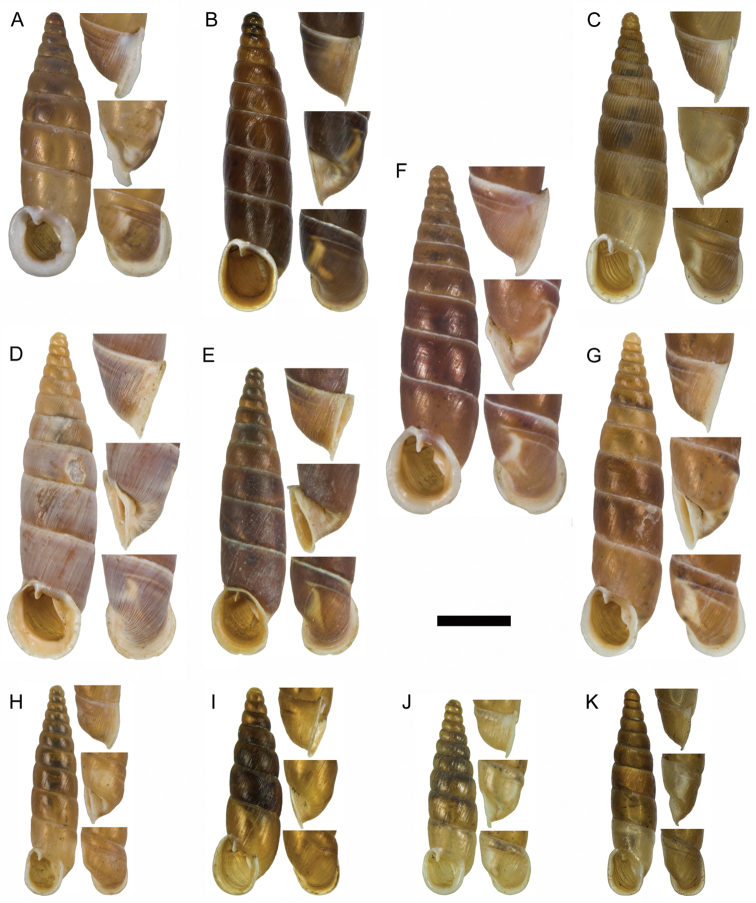
**A** *Montenegrina rugilabris rugilabris* (Mousson, 1859), syntype, SMF 176313 **B** *Montenegrina rugilabris golikutensis* ssp. n., holotype, NHMW 111214 **C** *Montenegrina rugilabris gregoi* ssp. n., holotype, HNHM 99474 **D** *Montenegrina rugilabris edmundi* Szekeres, 2006, HNHM 94850 **E** *Montenegrina rugilabris lambdaformis* Reischütz & Sattmann, 1990, holotype, NHMW 84367 **F** *Montenegrina rugilabris irmengardis* Klemm, 1962, paratype, SMF 165015 **G** *Montenegrina rugilabris welterschultesi* Fehér & Szekeres, 1999, holotype, HNHM 70840 **H** *Montenegrina soosi* Erőss & Szekeres, 2006, holotype, HNHM 94868 **I** *Montenegrina stankovici* (Urbański, 1960), Sveti Naum, HNHM 90879 **J** *Montenegrina sporadica sporadica* Nordsieck, 1974, holotype, SMF 227678 **K** *Montenegrina sporadica tropojana* ssp. n., holotype, NHMW 111224. Scale bar: 5 mm.

##### Type locality.

“Ianina” = Greece, Epirus, Ioannina.

##### Type material.

“Janina”, ex Mousson, ex Schaefli, syntypes (SMF 176313/7); same locality, ex Rossmässler, syntype (SMF 176314/1); same locality, ex Boettger, syntypes (SMF 176315/3), same locality, ex Jetschin, syntypes (SMF 94048/2); same locality, ex Shuttleworth, syntypes (NMBE 21042/6).

##### Other material.

Greece, Epirus, Mitsikeli Mts, N of Ligkiades, 1220 m, 39.7019°N, 20.8946°E, leg. JK, DM, Szederjesi, ZU, 4.v.2011 (HNHM 99546); Amfithea to Spothi road N of the Lake Pamvotis, 0.5 km E of the Ligkiades junction, 600 m, 39.6828°N, 20.8887°E, leg. ZE, ZF, JG, 24.vi.2014 (HNHM 99547); 1.5 km W of the Ligkiades junction, 530 m, 39.6895°N, 20.8714°E, leg. ZE, ZF, JG, 24.vi.2014 (HNHM 99548); 1 km W of the Ligkiades junction, 550 m, 39.6870°N, 20.8740°E, leg. ZE, ZF, JG, 24.vi.2014 (HNHM 99549); 0.5 km W of the Ligkiades junction, 570 m, 39.6849°N, 20.8795°E, leg. ZE, ZF, JG, 24.vi.2014 (HNHM 99550); near Restaurant Drabatova SE of Amfithea, 480 m, 39.6832°N, 20.8779°E, leg. AR, NR, PR, ix.2013 (NHMW 110430/MN/0034); Strouni, E of Perama, 480 m, 39.6860°N, 20.8727°E, leg. LP, PS, 30.vii.1976 (HNHM 31110); Lapsista near Ioannina, leg. Nordsieck, 21.viii.1971 (NHMW-K 65044)

##### Distribution.

**Figure 29.**
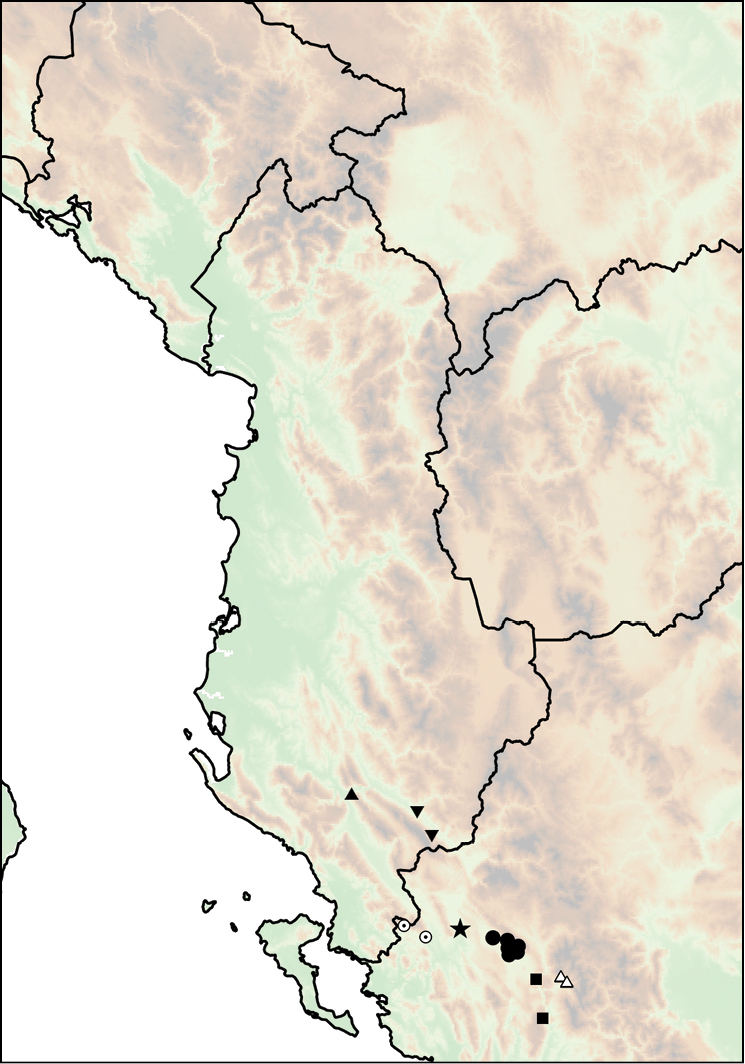
Distribution of *Montenegrina rugilabris*. *Montenegrina rugilabris edmundi* (empty circle with dot); *Montenegrina rugilabris golikutensis* ssp. n. (triangle); *Montenegrina rugilabris gregoi* ssp. n. (empty triangle); *Montenegrina rugilabris irmengardis* (square); *Montenegrina rugilabris lambdaformis* (star); *Montenegrina rugilabris rugilabris* (circle); *Montenegrina rugilabris welterschultesi* (inverted triangle).

Southwestern part and foothills of the Mitsikeli Mts, north of the Lake Pamvotis. Its range overlaps with that of *Montenegrina janinensis* (Fig. 29).

#### Montenegrina rugilabris edmundi

Taxon classificationAnimaliaStylommatophoraClausiliidae

Szekeres, 2006

Fig. 28D

Montenegrina irmengardis edmundi Szekeres, 2006 in Erőss et al. 2006: 190–192, fig. 11.
Montenegrina skipetarica edmundi – Nordsieck 2009: 73.

##### Diagnosis.

Shell large, purplish-brown with strong whitish overlay. Lower whorls are opaque and densely striate, upper ones increasingly glossy with diffuse striae. Neck not or barely inflexed, basal and peripheral crests very weak. Peristome attached, with somewhat swollen margin. Lamellae superior and spiralis overlap. In front view lamella inferior well emerged, subcolumellaris mostly hidden. Lunella dorsal-dorsolateral, fused to the long basalis. Subclaustralis short. Anterior plica superior mostly separate from the lunella complex.

##### Dimensions

(in mm). H_s_: 20.2–23.9 (holotype 22.2), W_s_: 4.8–6.1 (holotype 5.3).

##### Type locality.

Greece, Epirus, 6 km from Lia toward Lista, NE of Filiates, 460 m, 39.7358°N, 20.4537°E.

##### Type material.

Type locality, leg. PS, Szekeres, 16.v.1997, holotype (HNHM 94850), paratypes (NHMUK 20050220, HNHM 94851, NHMW 103279, RMNH 100707, SMF 328083, NMBE 534996/7); same locality, leg. ZE, AH, 18.vii.2004, paratypes (HNC 63184, HNHM 94852); Thesprotia, 8.5 km ESE of Tsamantas, between Lista and the junction to Keramitsa, 470 m, UTM DK50, leg. Gittenberger, 27.v.1991, paratypes (RMNH 100706/14).

##### Other material.

Type locality, 39.7358°N, 20.4537°E, leg. ZE, ZF, JG, 25.vi.2013 (HNHM 99697, NHMW 110430/MN/0173); near the waterfall at the SE end of Tsamantas, 620 m, leg. PS, 17.vii.1990 (MNBE)

##### Distribution.

Southeastern part of the Mt. Mourgana in Epirus, northwestern Greece. In addition to the type locality a site is also known near Tsamantas (Fig. 29).

##### Remarks.

In a recent assessment Nordsieck (2008) classified *Montenegrina rugilabris edmundi* (as well as the Montenegrina rugilabris subspecies irmengardis, *lambdaformis* Reischütz & Sattmann, 1990 and *welterschultesi* Fehér & Szekeres, 1999) with *Montenegrina skipetarica*.

#### Montenegrina rugilabris golikutensis ssp. n.

Taxon classificationAnimaliaStylommatophoraClausiliidae

http://zoobank.org/1543A07D-169C-4499-AE2F-B8C99B46E33A

Fig. 28B

##### Diagnosis.

Medium-size, light to darker purplish-brown subspecies with smooth whorls, not overlapping lamellae superior and spiralis, in front view not visible lamella subcolumellaris, and mostly separate anterior plica superior and lunella complex.

##### Description.

The medium-size, dark to lighter purplish-brown shell consists of 9 to 10 whorls, which are separated by a whitish suture. The entire surface is smooth, except that of the weakly inflexed, very finely and densely costate neck. The basal and peripheral crests are weak. The light brown, ovoid to somewhat quadrangular, narrowly attached peristome has slightly swollen margin. The lamella superior initiates at the end of the spiralis, without overlap. The lamella inferior is moderately emerged, its end part descends straight. The broadly bent subcolumellaris is not visible in front view. The dorsal to dorsolateral lunella is short and wide, fused to the short subclaustralis but not to the much longer basalis. The sulcalis is strong. The anterior par of the plica superior is long, often connected to the lunella complex. The clausilium plate is partly visible through the aperture.

##### Dimensions

(in mm). Holotype H_s_: 17.6, W_s_: 4.7, H_a_: 4.3, W_a_: 3.5; paratypes H_s_: 16.2–20.1 (mean 18.3, S.D. 1.17), W_s_: 4.6–5.8 (mean 5.0, S.D. 0.33), H_a_: 4.3–5.3, W_a_: 3.3–4.2 (NHMW 111215, n = 12).

##### Differential diagnosis.

Differs from *Montenegrina rugilabris irmengardis* and *Montenegrina rugilabris lambdaformis* Reischütz & Sattmann, 1990 by the non-overlapping lamellae superior and spiralis, deeper lunella, and the separate basalis, whereas from all other *Montenegrina rugilabris* subspecies by its purplish-brown shell surface.

##### Type locality.

Albania, Tepelenë District, Kendrevicë Mts, N slope of the Maja e Golikut, 1350 m, 40.2758°N, 20.0947°E.

##### Type material.

Type locality, leg. DA, ZF, JG, 28.vi.2014, holotype (NHMW 111214), paratypes (HNHM 99363/35, NHMW 111215/23+12a+18aj, GR/36, SZ/4); Mali i Golikut, E of Kodër, 1270 m, 40.2758°N, 20.0940°E, leg. ZB, DP, Schmidt, 11.viii.2006, paratypes (HNHM 99479/18, SZ/4); Mali i Golikut, 2.2 km E of Kodër, 1380 m, 40.2733°N, 20.0943°E, leg. ZB, DP, 11.viii.2006, paratype (HNHM 99480).

##### Etymology.

The new taxon is named after the Mt. Goliku (Mali i Golikut), its type locality.

##### Distribution.

Kendrevicë Mts in southern Albania. Known only from the type locality and its vicinity (Fig. 29).

#### Montenegrina rugilabris gregoi ssp. n.

Taxon classificationAnimaliaStylommatophoraClausiliidae

http://zoobank.org/F0F4FB6D-CBF9-4DB3-AE23-BF448965DD91

Fig. 28C

##### Diagnosis.

Medium-size, tumid subspecies with costate apical whorls, long overlapping lamellae superior and spiralis, and in front view visible lamella subcolumellaris.

##### Description.

The medium-size, tumid, light corneous shell consists of 9½ to 11½ whorls. The surface of the lower whorls varies between almost smooth to indistinctly costate with widely spaced, wrinkle-like ribs. The apex is and the weakly inflexed neck are strongly costate. The basal and peripheral crests are weak. The wide-rimmed peristome is almost circular, its upper margin is narrowly attached. There is a long overlap between the lamellae superior and spiralis. The moderately emerged lamella inferior and the broadly-bent subcolumellaris are both well visible in front view. The dorsal lunella is fused to the basalis, which is much longer than the subclaustralis. The sulcalis is strong. The anterior part of the plica superior is not connected to the lunella complex. Part of the clausilium plate is visible through the aperture.

##### Dimensions

(in mm). Holotype H_s_: 19.7, W_s_: 5.7, H_a_: 4.9, W_a_: 4.0; paratypes H_s_: 15.3–19.9 (mean 17.8, S.D. 1.34), W_s_: 4.5–5.7 (mean 5.1, S.D. 0.35), H_a_: 3.9–4.9, W_a_: 3.5–4.1 (HNHM 99475, n = 12).

##### Differential diagnosis.

Distinguishable from all other *Montenegrina rugilabris* subspecies by its costate apical whorls.

##### Type locality.

Greece, Epirus, Ioannina District, 3 km NE of Prosilio, along the road to Syrrako, 1130 m, 39.5768°N, 21.0969°E.

##### Type material.

Type locality, leg. ZE, ZF, JG, 23.vi.2013, holotype (HNHM 99474), paratypes (HNHM 99475/21+27a, NHMW 111211/10a+39aj, ER/44, GR/44, SZ/8).

##### Other material.

Greece, Epirus, 1 km W of Kipina Monastery, 620 m, 39.5668°N, 21.1231°E, leg. ZE, ZF, JG, 23.vi.2013 (HNHM 99476, NHMW 111212); Kipina Monastery, 700 m, 39.5681°N, 21.1313°E, leg. ZE, ZF, JG, 23.vi.2013 (HNHM 99477, NHMW 111213).

##### Etymology.

The new taxon is named after Jozef Grego, malacologist, and also to his son Maroš Grego, who accompanied the first author on several Balkan field trips, including the one during which when this subspecies was discovered.

##### Distribution.

Lakmos Mts in northwestern Greece. Known from two nearby localities in the western part of the Lakmos (Fig. 29).

##### Remarks.

The specimens from the vicinity of the Kipina Monastery have weaker sculpture than those of the type locality.

#### Montenegrina rugilabris irmengardis

Taxon classificationAnimaliaStylommatophoraClausiliidae

Klemm, 1962

Fig. 28F

Montenegrina (Beieriella) irmengardis Klemm, 1962: 242–244, plate 3, figs 2 (genital anatomy), 8 (shell).
Montenegrina irmengardis irmengardis – Nordsieck 1972: 35, plate 4, fig. 34. – Zilch 1981: 128, plate 15, fig. 40.
Montenegrina skipetarica irmengardis – Nordsieck 2009: 73.

##### Diagnosis.

Shell large, dark purplish-brown with whitish suture. Lower whorls smooth, upper ones smooth to finely wrinkled-costate. Neck not to slightly inflexed, finely and densely striate. Basal crest well recognizable, peripheral crest weak. Peristome attached, with somewhat swollen margin. Lamellae superior and spiralis overlap. In front view lamella inferior well emerged, subcolumellaris mostly visible. Lunella dorsal-dorsolateral, fused to the basalis. Subclaustralis short. Anterior plica superior mostly separate from the lunella complex.

##### Dimensions

(in mm). H_s_: 20.1–26.2, W_s_: 5.2–6.5 (Nordsieck 1972).

##### Type locality.

Greece, Epirus, “Platanusa” (= Platanoussa), 750–800 m.

##### Type material.

Type locality, leg. Beier, 3–10.vi.1933, holotype (NHMW 74164), paratypes (NHMW 74165/pl., NHMW-K 47162/pl. NHMW-K 47163/pl., NHMW-K 47165/pl., NHMW-E 58051/10, NHMW-E 58052/15, SMF 165015/5, SMF 201606/5, SMF 265701/10, NMBE 535001/1, NMBE 535000/2, SMNS-N 1277, HNHM 36719/2).

##### Other material.

Type locality, 600 m, 39.4279°N, 21.0106°E, leg. ZE, ZF, JG, 23.vi.2013 (HNHM 99554); Epirus, W of Kedros, 2 km toward Charakopi, 730 m, 39.5728°N, 20.9861°E, leg. ZE, ZF, JG, 24.vi.2013 (HNHM 99552); 4 km E of Charakopi, bridge of the Arachtos River, 380 m, 39.5763°N, 20.9772°E, leg. ZE, ZF, JG, 24.vi.2013 (HNHM 99553).

##### Distribution.

Arachtos Valley west of the Lakmos and Tzoumerka mountains in northwestern Greece. In addition to the type locality and its surroundings, this taxon is also known from another site ca. 17 km N of the type locality (Fig. 29).

#### Montenegrina rugilabris lambdaformis

Taxon classificationAnimaliaStylommatophoraClausiliidae

Reischütz & Sattmann, 1990

Fig. 28E

Montenegrina irmengardis lambdaformis Reischütz & Sattmann, 1990: 260, plate 3, fig. 7.
Montenegrina skipetarica lambdaformis – Nordsieck 2009: 73.

##### Diagnosis.

Shell medium to large, dark purplish-brown with whitish suture. All whorls smooth, lower ones separated by whitish suture. Neck not to slightly inflexed, densely striate. Basal crest well recognizable, peripheral crest weak. Peristome mostly detached, with somewhat swollen margin. Lamellae superior and spiralis overlap. In front view lamella inferior well emerged, subcolumellaris mostly visible. Lunella dorsal-dorsolateral, fused to the basalis. Subclaustralis short. Anterior plica superior separate from the lunella complex.

##### Dimensions

(in mm). H_s_: 17–20 (holotype 19.5), W_s_: 4.0–5.0 (holotype 4.5 mm).

##### Type locality.

Greece, Epirus, Pateron Monastery, W of Zitsa.

##### Type material.

Type locality, leg. PR, vii.1987, holotype (NHMW 84367), paratypes (NHMW 84368/3).

##### Other material.

Greece, Epirus, E of Lithino, ca. 1 km N of the Pateron Monastery, 360 m, 39.7720°N, 20.6180°E, leg. ZE, ZF, JG, 25.vi.2013 (HNHM 99555).

##### Distribution.

Epirus in northwestern Greece. Known only from the vicinity of the Pateron Monastery (Fig. 29).

#### Montenegrina rugilabris welterschultesi

Taxon classificationAnimaliaStylommatophoraClausiliidae

Fehér & Szekeres, 1999

Fig. 28G

Montenegrina irmengardis welterschultesi Fehér & Szekeres, 1999 in Erőss et al. 1999: 450, fig. 6.
Montenegrina skipetarica welterschultesi – Nordsieck 2009: 73.

##### Diagnosis.

Shell medium to large, purplish-brown with whitish suture. All whorls smooth, lower ones separated by whitish suture. Neck barely inflexed, densely striate. Basal and peripheral crests weak. Peristome attached, with somewhat swollen margin. Lamellae superior and spiralis long overlap. In front view lamella inferior moderately emerged, subcolumellaris mostly hidden visible. Lunella dorsal-dorsolateral, fused to the basalis. Subclaustralis short. Anterior plica superior separate from the lunella complex.

##### Dimensions

(in mm). H_s_: 16.0–22.7 (holotype 22.1), W_s_: 4.0–5.0 (holotype 4.8).

##### Type locality.

Albania, Përmet District, Petran, at the confluence of the Vjosë and Lengaricë Rivers, 300 m, 40.2094°N, 20.4126°E.

##### Type material.

Type locality, leg. ZE, ZF, 7.vii.1996, holotype (HNHM 70840), paratypes (HNHM 70841/7, NHMW 100080/2, SMF 312508/2, NMBE 535015/1, NMBE 22596/2).

##### Other material.

Type locality, leg. ZE, ZF, KK, 13.iv.2001 (HNHM 86127); same locality, leg. ZF, LT, 18.viii.2007 (HNHM 99556); same locality, leg. PJ, TK, DM, GP, 13.x.2012 (HNHM 99557); Përmet District, Nemerçkë Mts, gorge ca. 2 km SW of Draçovë, 450 m, 40.1226°N, 20.4805°E, leg. DA, ZE, ZF, JG, 29.vi.2014 (HNHM 99558, NHMW 110430/MN/0035).

##### Distribution.

Nemerçkë Mts in southern Albania. Welter-Schultes (1996) found this taxon 6 km southeast of Përmët, in the vicinity of the type locality. Recently another population has been found near Draçovë, indicating that this taxon might be wider distributed in the Nemerçkë Mts (Fig. 29).

#### Montenegrina skipetarica

Taxon classificationAnimaliaStylommatophoraClausiliidae

(Soós, 1924)

##### Diagnosis.

Shell small to large, light corneous to violet-brown. Neck variably inflexed. Peristome mostly attached. In front view lamella inferior moderately to well emerged. Subcolumellaris broadly-bent. Lunella short and broad, dorsal-dorsolateral to dorsolateral. Lunella complex with the basalis can form a lambda-like structure. Plica superior fused to the principalis. Basalis and subclaustralis of comparable length. Sulcalis weak or residual. Anterior plica superior absent to strong. Clausilium plate well visible through the aperture. Distinguishable from *Montenegrina rugilabris* by the longer subclaustralis and weak sulcalis.

#### Montenegrina skipetarica skipetarica

Taxon classificationAnimaliaStylommatophoraClausiliidae

(Soós, 1924)

Fig. 30E

Clausilia (Delima) skipetarica Soós, 1924: 181–183, figs 2 (shell), 3 (genital anatomy).
Delima (Albanodelima) skipetarica – Wagner 1924: 120.
Montenegrina perstriata skipetarica – Erőss et al. 2006: 203, fig. 26. – Nordsieck 2009: 73, plate 1, fig. 1.

##### Diagnosis.

Shell large, tumid, reddish-brown with whitish suture. Lower whorls smooth, upper ones finely costate. Neck weakly inflexed, wrinkled-costate. Basal crest well recognizable, peripheral crest weak. Peristome attached, ovoid to somewhat quadrangular, with simple margin. Lamellae superior and spiralis overlap. In front view lamella inferior well emerged, subcolumellaris mostly not visible. Lunella dorsal-dorsolateral, usually connected to the short basalis. Subclaustralis short, sulcalis residual. Anterior plica superior absent or week and separate from the lunella complex.

##### Dimensions

(in mm). H_s_: 18.5–23.4, W_s_: 5.6–6.8 (topotypes HNHM 99521).

**Figure 30.**
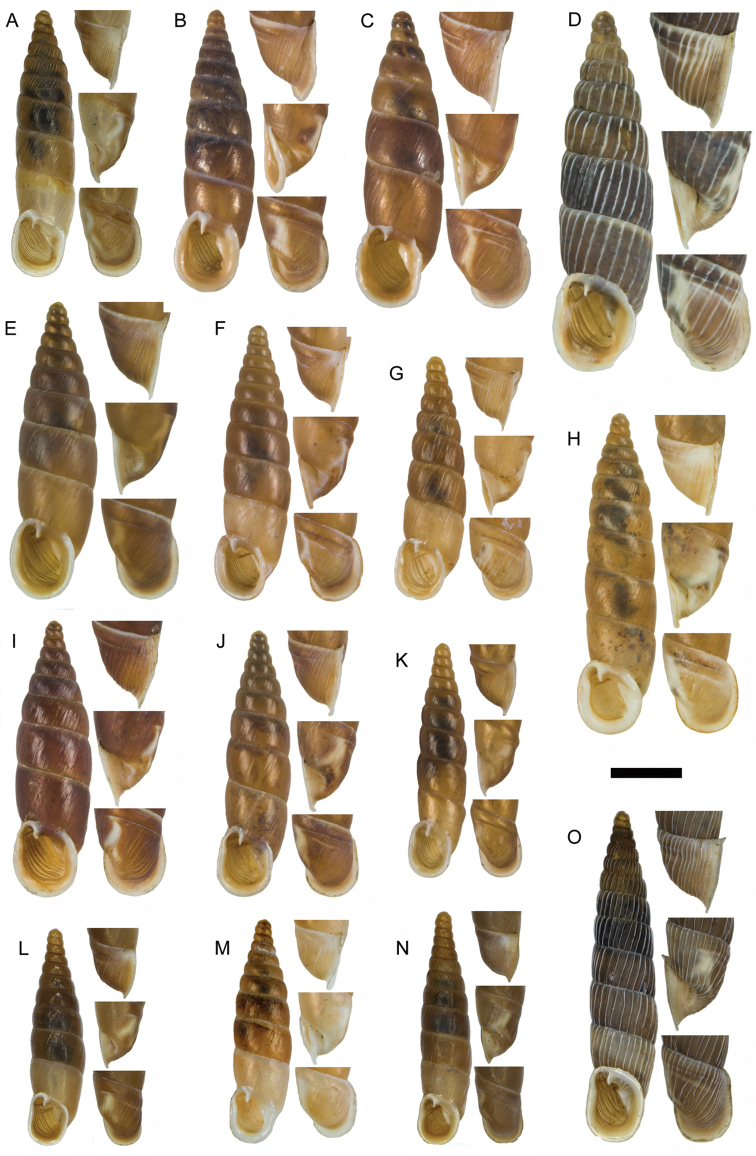
**A** *Montenegrina skipetarica puskasi* ssp. n., holotype, HNHM 99448 **B** *Montenegrina skipetarica konitsae* Nordsieck, 1972, holotype, SMF 221044 **C** *Montenegrina skipetarica voidomatis* Nordsieck, 1974, holotype, SMF 227688 **D** *Montenegrina skipetarica thysi* Loosjes & Loosjes-van Bemmel, 1988, Vikos Gorge, Agia Paraskevi, HNHM 99532 **E** *Montenegrina skipetarica skipetarica* (Soós, 1924), Ura e Lapavës, HNHM 99521 **F** *Montenegrina skipetarica ersekensis* Nordsieck, 1996, 4.5 km S of Barmash, HNHM 99533 **G** *Montenegrina skipetarica rugosa* Fehér & Szekeres, 2006, holotype, HNHM 94862 **H** *Montenegrina skipetarica nobilis* Erőss & Szekeres, 2006, holotype, HNHM 94844 **I** *Montenegrina skipetarica remota* Fehér & Szekeres, 2006, holotype, HNHM 94859 **J** *Montenegrina skipetarica flava* Erőss & Szekeres, 2006, holotype, HNHM 94853 **K** *Montenegrina skipetarica csikii* Erőss & Szekeres, 2006, holotype, HNHM 94837 **L** *Montenegrina skipetarica pifkoi* ssp. n., holotype, HNHM 99464 **M** *Montenegrina skipetarica pindica* Nordsieck, 1988, holotype, NHMW 84024a **N** *Montenegrina skipetarica danyii* ssp. n., holotype, HNHM 99471 **O** *Montenegrina skipetarica gurelurensis* ssp. n., holotype, HNHM 99459. Scale bar: 5 mm.

##### Type locality.

“Ura i Lopez” = Albania, Kukës District, 2.5 km N of Bushtricë along the Kukës to Peshkopi road, Ura e Lapavës at the gorge of the Përroi i Vaut të Çajës, 600 m.

##### Type material.

The original series, collected by Csiki, were destroyed in 1956 by a fire at the HNHM (Erőss et al. 2006).

##### Other material.

Type locality, 41.8949°N, 20.4169°E, leg. ZE, ZF, JK, DM, 25.vi.2003 (HNHM 93813); type locality, leg. LD, ZE, ZF, AH, DM, 26.vi.2007 (HNHM 99521); Mat District, 3 km W of the Qafa e Murrës, Shkëmb i Skanderbeut, gorge of the Lumi i Varoshit, 970 m, 41.6465°N, 20.1901°E, leg. ZE, ZF, JK, DM, 26.vi.2003 (HNHM 94914); same locality, leg. TD, ZE, ZF, DM, 11.x.2005 (HNHM 96806); same locality, leg. ZE, ZF, AH, DM, 13.iv.2006 (HNHM 96809); same locality, leg. ZB, ZF, DM, DP, ZU, 18.v.2010 (HNHM 99522); Mat District, 5 km toward Fushë-Lurë from the Peshkopi to Burrel road, upper valley of the Lumi i Varoshit, 1360 m, 41.6651°N, 20.2083°E, leg. ZF, DM, ZU, 18.v.2010 (HNHM 99523); 5 km E of Lis along the Peshkopi to Burrel road, 850 m, 41.6361°N, 20.1228°E, leg. ZE, ZF, JK, DM, 26.vi.2003 (HNHM 94842); same locality, leg. ZE, ZF, AH, DM, 13.iv.2006 (HNHM 96815); W of the Shkëmb i Skanderbeut, 2 km from the Peshkopi to Burrel road toward Fushë-Lurë, 1180 m, 41.6502°N, 20.1861°E, leg. ZE, ZF, AH, DM, 13.iv.2006 (HNHM 96807).

##### Distribution.

**Figure 31.**
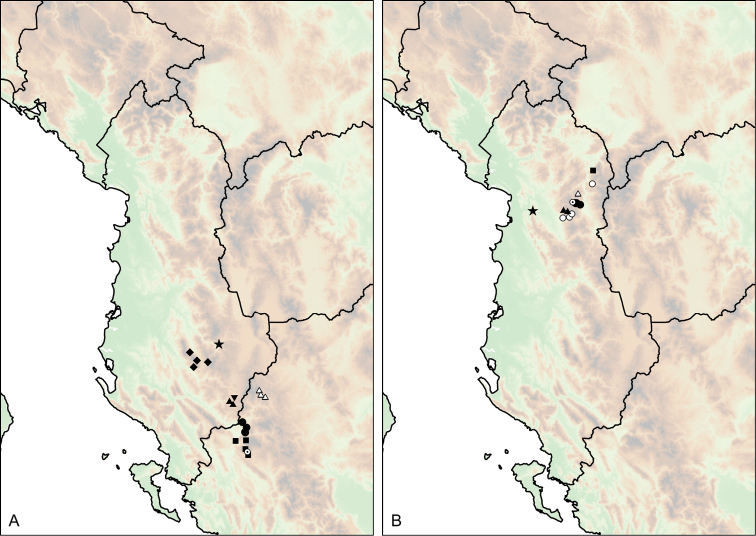
Distribution of *Montenegrina skipetarica*. **A** *Montenegrina skipetarica ersekensis* (triangle); *Montenegrina skipetarica flava* (star); *Montenegrina skipetarica konitsae* (circle); *Montenegrina skipetarica nobilis* (inverted triangle); *Montenegrina skipetarica pindica* (empty triangle); *Montenegrina skipetarica rugosa* (diamond); *Montenegrina skipetarica thysi* (empty circle with dot); *Montenegrina skipetarica voidomatis* (square) **B** *Montenegrina skipetarica csikii* (square); *Montenegrina skipetarica danyii* ssp. n. (empty triangle); *Montenegrina skipetarica gurelurensis* ssp. n. (empty circle with dot); *Montenegrina skipetarica pifkoi* ssp. n. (triangle); *Montenegrina skipetarica puskasi* ssp. n. (circle); *Montenegrina skipetarica remota* (star); *Montenegrina skipetarica skipetarica* (empty circle).

Lura-Dejë mountain group and the Korab Mts in northern Albania. Most of the known occurrences are in the southern part of the Lura-Dejë mountain group. The type locality, however, is 30–40 km northeast of this area, and an isolated occurrence is in the northwestern part of the Korab Mts (Fig. 31B).

##### Remarks.

Soós’ paper (1924) describing *Montenegrina perstriata skipetarica* is a chapter of a larger study assessing the results of Csiki’s zoological explorations in northern Albania. Whereas the complete volume was published only in 1940, the chapter written by Soós had already been printed and circulated in 1924. Therefore, in the case of *skipetarica* this earlier date is to be regarded as the year of description (Erőss et al. 2006).

#### Montenegrina skipetarica csikii

Taxon classificationAnimaliaStylommatophoraClausiliidae

Erőss & Szekeres, 2006

Fig. 30K

Montenegrina apfelbecki csikii Erőss & Szekeres, 2006 in Erőss et al. 2006: 185–186, fig. 4.
Montenegrina janinensis csikii – Nordsieck 2009: 75.

##### Diagnosis.

Shell medium, elongate, light yellowish-brown. Whorls smooth. Neck inflexed, with dense, sharp ribs. Basal and peripheral crests well recognizable. Peristome attached, ovoid to angular, with simple margin. Lamellae superior and spiralis do not overlap. In front view lamella inferior moderately emerged, subcolumellaris hidden. Lunella dorsolateral, mostly fused to the strong basalis. Subclaustralis and sulcalis present. Anterior plica superior mostly absent, if present occasionally connected to the lunella complex.

##### Dimensions

(in mm). H_s_: 14.4–19.2 (holotype 16.8), W_s_: 3.3–3.9 (holotype 3.5).

##### Type locality.

Albania, Kukës District, Bicaj, gorge of the Përroi i Tershanës, 500 m, 41.9897°N, 20.4202°E.

##### Type material.

Type locality, leg. ZE, ZF, JK, DM, 25.vi.2003, holotype (HNHM 94837), paratypes (NHMUK 20050216, HNC 63180, HNHM 94838/33, NHMW 103274, RMNH 100316, SMF 328078, NMBE 534892/4).

##### Other material.

Type locality, leg. LD, ZE, ZF, AH, DM, 25.vi.2007 (HNHM 96845).

##### Distribution.

Southern part of the Mt. Gjalica e Lumës in northern Albania. Known only from the type locality (Fig. 31B).

#### Montenegrina skipetarica danyii ssp. n.

Taxon classificationAnimaliaStylommatophoraClausiliidae

http://zoobank.org/55989E67-BECD-45BE-B94B-60E59497A0F4

Fig. 30N

##### Diagnosis.

Small, slender subspecies with costate apical whorls and neck, overlapping lamellae superior and spiralis, and in front view mostly visible lamella subcolumellaris.

##### Description.

The small, slender, light brownish-corneous shell consists of 10 to 11½ whorls. The lower whorls are smooth, the upper ones are increasingly costate toward the tip. The weakly inflexed neck is densely costate. The basal crest is weak, the peripheral one is well visible. The ovoid to somewhat quadrangular peristome is broadly attached, its margin is somewhat swollen. The lamella superior is long, its frontal half is of even height. Inward it overlaps with the lamella spiralis. The moderately emerged lamella inferior ends with a straight descent. The broadly-bent lamella subcolumellaris is mostly visible in front view. The inner end of the plica principalis is often fused to the superior. The dorsolateral lunella is short and broad, it is mostly connected to the plica basalis. The basalis and subclaustralis are of the same length. The sulcalis is week. The anterior part of the plica superior is weak, separate from the lunella complex, occasionally absent. The clausilium plate is only barely visible through the aperture.

##### Dimensions

(in mm). Holotype H_s_: 16.2, W_s_: 4.1, H_a_: 3.6, W_a_: 3.0; paratypes H_s_: 13.1–16.2 (mean 14.7, S.D. 0.77), W_s_: 3.5–4.1 (mean 3.8, S.D. 0.24), H_a_: 2.9–3.7, W_a_: 2.4–3.0 (HNHM 99472, n = 12).

##### Differential diagnosis.

The new subspecies can be distinguished from *Montenegrina skipetarica pifkoi* ssp. n. by its more slender shell and overlapping lamellae superior and spiralis, whereas from all other *Montenegrina skipetarica* subspecies by its considerably smaller size.

##### Type locality.

Albania, Dibrë District, 9 km N of Cidhnë along the road from Cidhnë to Fushë-Lurë, 1330 m, 41.8149°N, 20.2775°E.

##### Type material.

Type locality, leg. TD, ZE, ZF, DM, 10.x.2005, holotype (HNHM 99471), paratypes (HNHM 99472/48, MMM-B01323/49, ER/49, SZ/4); type locality, leg. LD, ZE, ZF, AH, DM, 29.vi.2007, paratypes (HNHM 99473/64, NHMW 111210/2+1a, ER/69, HU/69, SZ/3).

##### Etymology.

The new taxon is named after László Dányi, entomologist and pedozoologist (HNHM), who was the companion to the first author on several Balkan field trips, including the one during which this subspecies was collected.

##### Distribution.

Eastern part of the Lura Mountain in northern Albania. Known only from the type locality (Fig. 31B).

#### Montenegrina skipetarica ersekensis

Taxon classificationAnimaliaStylommatophoraClausiliidae

Nordsieck, 1996

Fig. 30F

Montenegrina ersekensis Nordsieck, 1996: 9–10, plate 2, fig. 3.
Montenegrina skipetarica ersekensis – Nordsieck 2009: 73.

##### Diagnosis.

Shell medium, light corneous. Lower whorls smooth, upper ones very finely, indistinctly costate. Neck inflexed, with fine and dense ribs. Basal and peripheral crests well recognizable. Peristome attached, somewhat angular, with slightly swollen and reflexed margin. Lamellae superior and spiralis long overlap. In front view lamella inferior well emerged, subcolumellaris mostly visible. Lunella dorsolateral, connected to the strong basalis. Subclaustralis well developed, sulcalis weak. Anterior plica superior strong, connected to the lunella complex.

##### Dimensions

(in mm). H_s_: 16.7–19.0 (holotype 16.7), W_s_: 4.4–5.0 (holotype 4.5 mm).

##### Type locality.

Albania, Ersekë District, 7 km SW of Ersekë along the road to Leskovik, 1100–1200 m.

##### Type material.

Type locality, leg. Welter-Schultes, 15.ix.1995, holotype (HNC 40587), paratypes (HNC 40585, SMNS-N 10311).

##### Other material.

Albania, Ersekë District, 4.5 km S of Barmash along the Leskovik to Ersekë road, 880 m, 40.2688°N, 20.6065°E, leg. ZF, LT, 17.viii.2007 (HNHM 99533); 4 km S of Barmash, 920 m, 40.2656°N, 20.6001°E, leg. DA, ZE, ZF, JG, 29.vi.2014 (HNHM 99375, NHMW 110430/MN/0031); 4.5 km NW of the Gërmenj junction, 890 m, 40.2418°N, 20.6414°E, leg. DA, ZE, ZF, JG, 29.vi.2014 (HNHM 99534, HNMW 110430/MN/0030).

##### Distribution.

Western extensions of the Gramos Mts in southern Albania (Fig. 31A). Known from a few sites of the limestone mountain between Barmash and Gërmenj along the Leskovik to Ersekë main road (see also: Welter-Schultes 1996).

##### Remarks.

The literature record, “2–5 km E of Çorovodë” (Welter-Schultes 1996), most probably corresponds to *Montenegrina skipetarica rugosa* Fehér & Szekeres 2006.

#### Montenegrina skipetarica flava

Taxon classificationAnimaliaStylommatophoraClausiliidae

Erőss & Szekeres, 2006

Fig. 30J

Montenegrina irmengardis flava Erőss & Szekeres, 2006 in Erőss et al. 2006: 192, fig. 12.
Montenegrina skipetarica flava – Nordsieck 2009: 73.

##### Diagnosis.

Shell medium to large, light yellowish-brown. Whorls smooth. Neck weakly inflexed, finely and densely wrinkled-costate. Basal crest well recognizable, peripheral crest weak. Peristome attached, ovoid, with somewhat swollen, reflexed margin. Lamellae superior and spiralis long overlap. In front view lamella inferior well emerged, broadly-bent subcolumellaris not or only barely visible. Lunella dorsolateral, connected to the strong basalis. Subclaustralis well developed, sulcalis weak. Anterior plica superior frequently connected to the lunella complex.

##### Dimensions

(in mm). H_s_: 17.7–20.9 (holotype 18.8), W_s_: 4.1–4.7 (4.6 mm).

##### Type locality.

Albania, Korçë District, gorge of the Lumi i Devollit at the Gjinikas junction, 25 km W of Maliq, along the Korçë to Gramsh road, 750 m, 40.6921°N, 20.5003°E.

##### Type material.

Type locality, leg. ZE, ZF, JK, DM, 4.vii.2003, holotype (HNHM 94853), paratypes (NHMUK 20050221, HNC 63185, HNHM 94854/26, NHMW 103280, RMNH 100309, SMF 328084).

##### Other material.

Type locality, leg. PJ, TK, DM, GP, 16.x.2013 (HNHM 99544); same locality, leg. DA, ZE, ZF, JG, 30.vi.2013 (HNHM 99545, NHMW 110430/MN/0033).

##### Distribution.

Southern parts of the Mokër Mts in southern Albania. Known only from the type locality (Fig. 31A).

#### Montenegrina skipetarica gurelurensis ssp. n.

Taxon classificationAnimaliaStylommatophoraClausiliidae

http://zoobank.org/E28615F7-5E2E-45EE-9C0C-591BD06B827A

Fig. 30O

##### Diagnosis.

Medium-size, strongly costate subspecies with detached peristome, dorsolateral lunella and often uninterrupted plica superior with long anterior part.

##### Description.

The shell is medium- to large-sized, light brownish-corneous, somewhat tumid, of its 9½ to 12½ whorls the lower three are almost of the same width. The surface is strongly costate, with denser ribs at the weakly inflexed neck. The basal crest is weak, the peripheral one is well visible. The light brown, ovoid peristome has simple, detached (rarely very narrowly attached) margin. The lamellae superior and spiralis overlap. The lamella inferior is emerged, well visible in front view. The broadly-bent lamella subcolumellaris is not visible in frontal, only in slanted view at the aperture. The lunella is dorsolateral, broad, mostly fused to the basalis that is as long as the subclaustralis. The sulcalis is week. The anterior part of the plica superior is long, ends in a lump behind the aperture, mostly does not reach the lunella complex. Part of the clausilium plate is visible through the aperture.

##### Dimensions

(in mm). Holotype H_s_: 23.5, W_s_: 5.4, H_a_: 5.5, W_a_: 4.2; paratypes H_s_: 14.4–23.5 (mean 20.1, S.D. 2.36), W_s_: 4.2–5.5 (mean 5.0, S.D. 0.39), H_a_: 4.1–5.5, W_a_: 3.2–4.3 (n = 12).

##### Differential diagnosis.

Distinguishable from *Montenegrina skipetarica thysi* Loosjes & Loosjes-van Bemmel, 1988 by the slender shell and deeper lunella, whereas from all other *Montenegrina skipetarica* subspecies by the costate shell and detached peristome.

##### Type locality.

Albania, Dibrë District, ca. 2 km W of Cidhnë along the footpath to Gurë-Lurë, gorge of the Përroi i Setës, 780 m, 41.7525°N, 20.2484°E.

##### Type material.

Type locality, leg. ZE, ZF, AH, DM, 12.iv.2006, holotype (HNHM 99459), paratypes (HNHM 99460/42+3aj, ER/42, HU/42, SZ/4); same locality, leg. ZE, DM, 10.x.2005, paratypes (HNHM 99461/24+7a+3aj, MMM-B01322/24, ER/24, SZ/6); 1.5–1.9 km W of Cidhnë, leg. ZE, ZF, JG, 1.vii.2015, paratypes (HNHM 99462/45, NHMW 111208/34+26a+12aj, GR/70).

##### Other material.

Albania, Dibrë District, ca. 1.5 km W of Cidhnë, 41.7528°N, 20.2505°E, leg. ZE, ZF, AH, DM, 12.iv.2006 (HNHM 99463).

##### Etymology.

The new taxon is named after the village Gurë-Lurë, as the type locality is along the footpath leading there.

##### Distribution.

Southern part of the Lura Mountain in northern Albania. This taxon is known only from the Setë Gorge, where it was found at a short section of the footpath between Cidhnë and Gurë-Lurë. Towards Cidhnë, at ca. 1.5 km from the hydroelectric station, there is a narrow contact zone between this taxon and *Montenegrina skipetarica puskasi*, and to the east of that zone only *Montenegrina skipetarica puskasi* could be found (Fig. 31B).

#### Montenegrina skipetarica konitsae

Taxon classificationAnimaliaStylommatophoraClausiliidae

Nordsieck, 1972

Fig. 30B

Montenegrina irmengardis konitsae Nordsieck, 1972: 35, plate 4, fig. 35. – Zilch 1981: 129, plate 15, fig. 41.
Montenegrina skipetarica konitsae – Nordsieck 2009: 73.

##### Diagnosis.

Shell large, tumid, light reddish-brown with whitish suture. All whorls smooth. Neck weakly inflexed, finely and densely costate. Basal crest well recognizable, peripheral crest weak. Peristome attached, ovoid, with simple margin. Lamellae superior and spiralis overlap. In front view lamella inferior well emerged, subcolumellaris mostly visible. Lunella dorsolateral, fused to the short basalis. Subclaustralis is short, sulcalis weak. Anterior plica superior short and weak, mostly separate from the lunella complex.

##### Dimensions

(in mm). H_s_: 16.4–23.3 (holotype 21.8), W_s_: 5.0–6.2 (holotype 5.8 mm).

##### Type locality.

Greece, Epirus, Konitsa.

##### Type material.

Type locality, leg. Nordsieck, 22.viii.1971, holotype (SMF 221044), paratypes (SMF 221045, SMNS-N 5467).

##### Other material.

Type locality, leg. Fauer, 11.viii.1972 (marked as paratypes on the NMBE label, but probably not type materaial) (NMBE 535008/6, NHMW-K 65356/5); Konitsa, valley of the Aoos River at the old bridge, 440 m, 40.0355°N, 20.7469°E, leg. ZE, ZF, JG, 26.vi.2013 (HNHM 99527); near the spring at Stomio Monastery, leg. HS, 26.vi.1988 (NHMW 102871/3); Epirus, Sarantaporos Gorge near the Exochi to Agia Varvara road, 530 m, 40.1102°N, 20.7203°E, leg. ZE, ZF, JG, 26.vi.2013 (HNHM 99528); Gamila (= Tymfi) Mts, W side, leg. HS, 27.vi.1988 (NHMW 102869/2); Epirus, NW of Konitsa, gorge of the Topolca Stream, 600 m, 40.0706°N, 20.7608°E, leg. Fauer, PS, 26.ix.1989 (NMBE).

##### Distribution.

Tymfi Mts in Epirus, northwestern Greece. Known from a few localities around Konitsa, from the northern and western sides of the Tymfi Mts (catchment area of the Aoos River), and from the gorge of the Sarantaporos River, ca. 8 km northwest of Konitsa (Fig. 31A).

#### Montenegrina skipetarica nobilis

Taxon classificationAnimaliaStylommatophoraClausiliidae

Erőss & Szekeres, 2006

Fig. 30H

Montenegrina ersekensis nobilis Erőss & Szekeres, 2006 in Erőss et al. 2006: 189, fig. 8.
Montenegrina skipetarica nobilis – Nordsieck 2009: 73.

##### Diagnosis.

Shell large, elongate, light corneous. Lower whorls smooth, upper ones with very fine, indistinct ribs. Neck weakly inflexed, finely and densely costate. Basal and peripheral crests well recognizable. Peristome attached, mostly ovoid, with swollen and reflexed margin. Lamellae superior and spiralis long overlap. In front view lamella inferior well emerged, subcolumellaris mostly visible. Lunella dorsolateral, fused to the long basalis. Subclaustralis and sulcalis well developed. Anterior plica superior strong, connected to the lunella complex.

##### Dimensions

(in mm). W_s_: 18.0–24.2 (holotype 22.5), W_s_: 4.5–5.5 (holotype 5.0).

##### Type locality.

Albania, Ersekë District, 4 km S of Borovë, NE of Barmash, on the Ersekë to Leskovik road, 1040 m, 40.2913°N, 20.6273°E.

##### Type material.

Type locality, leg. EZ, FZ, JK, DM, 3.vii.2003, holotype (HNHM 94844), paratypes (NHMUK 20050218, HNC 63182, HNHM 94845/26, NHMW 103276, RMNH 100327, SMF 328080, NMBE 534895/1).

##### Other material.

Type locality, leg. PJ, TK, DM, GP, 15.x.2013 (HNHM 99536); same locality, leg. DA, ZE, ZF, JG, 29.vi.2014 (HNHM 99382, HNMW 110430/MN/0032).

##### Distribution.

Western extensions of the Gramos Mts in southern Albania. Known only from the type locality (Fig. 31A).

##### Remarks.

Although not synonymized formally, Nordsieck (2009) questioned the distinct taxonomic position of this subspecies.

#### Montenegrina skipetarica pifkoi ssp. n.

Taxon classificationAnimaliaStylommatophoraClausiliidae

http://zoobank.org/4A70F0D7-1163-48C5-A893-914E6A259AA8

Fig. 30L

##### Diagnosis.

Small subspecies with indistinctly costate apical whorls, wrinkled neck, non-overlapping lamellae superior and spiralis, and frontally visible lamella subcolumellaris.

##### Description.

The small, light brownish-corneous shell consists of 9 to 11½ whorls. The lower whorls are smooth, the upper ones are indistinctly wrinkled-costate. The weakly inflexed neck is strongly, irregularly wrinkled. The basal crest is weak, the peripheral one is well recognizable. The ovoid to somewhat quadrangular peristome is broadly attached, its narrow margin is somewhat swollen. The lamella superior initiates at the depth where the spiralis ends, without overlap. The moderately emerged terminal part of the lamella inferior descends in a straight line. In front view the broadly-bent lamella subcolumellaris is mostly well visible. The inner end of the plica principalis is often fused to the superior. The dorsolateral lunella is short and broad, it is connected to the similarly short plica basalis. The basalis and subclaustralis are of the same length. The sulcalis is week. The anterior part of the plica superior is separate from the lunella complex. The clausilium plate is partly visible through the aperture.

##### Dimensions

(in mm). Holotype H_s_: 15.4, W_s_: 4.2, H_a_: 3.7, W_a_: 2.9; paratypes H_s_: 13.2–17.1 (mean 15.4, S.D. 1.24), W_s_: 3.7–4.4 (mean 4.0, S.D. 0.21), H_a_: 3.4–4.0, W_a_: 2.6–3.2 (HNHM 99465, n = 12).

##### Differential diagnosis.

Differs from *Montenegrina skipetarica danyii* ssp. n. by the stouter shell and non-overlapping lamellae superior and spiralis, from *Montenegrina skipetarica pindica* by the separate anterior plica superor, whereas from all other *Montenegrina skipetarica* subspecies by the much smaller shell size.

##### Type locality.

Albania, Mat District, Mali i Dejës, Macukull, rocky forest E (above) of the village, 1280 m, 41.6971°N, 20.1362°E.

##### Type material.

Type locality, leg. ZF, DM, ZU, 19.v.2010, holotype (HNHM 99464), paratypes (HNHM 99465/81, NHMW 111209/11+1a, SZ/7); limestone rocks above and E of Macukull, 1260 m, 41.6962°N, 20.1333°E, leg. ZF, DM, ZU, 19.v.2010, paratypes (HNHM 99466/121+2a+2aj, SZ/6).

##### Other material.

Albania, Mat District, above Macukull, a deep doline in beech forest, 1470 m, 41.6908°N, 20.1468°E, leg. ZB, DP, 8.viii.2009 (HNHM 99467); karst slope above Macukull with beech forest, 1700 m, 41.6886°N, 20.1557°E, leg. ZB, DP, 8.viii.2009 (HNHM 99468); above Macukull, rocky grassland, 1740 m, 41.6878°N, 20.1568°E, leg. ZB, DP, 8.viii.2009 (HNHM 99469); above Macukull, doline near the Shkol-Den Pass, 1940 m, 41.6902°N, 20.1659°E, leg. ZB, DP, 9.viii.2009 (HNHM 99470).

##### Etymology.

The new taxon is named after Dániel Pifkó, botanist (HNHM), who collected invaluable zoological material during his field trips in Albania.

##### Distribution.

Mt. Dejë in northern Albania. This taxon was found at the western slope along the climbing path from Macukull village to the Dejë Summit, between 1250 and 2000 m (Fig. 31B).

#### Montenegrina skipetarica pindica

Taxon classificationAnimaliaStylommatophoraClausiliidae

Nordsieck, 1988

Fig. 30M

Montenegrina dofleini pindica Nordsieck, 1988: 199, fig. 3. – Nordsieck 2009: 74.

##### Diagnosis.

Shell medium, light corneous. Lower whorls smooth, apical ones finely striate. Neck barely inflexed, striate. Basal crest weak, peripheral crest not recognizable. Peristome attached, with swollen margin. Lamellae superior and spiralis do not overlap. In front view lamella inferior moderately emerged, subcolumellaris mostly visible. Lunella dorsolateral, connected to the short basalis. Subclaustralis short, sulcalis weak. Anterior plica superior frequently connected to lunella complex.

##### Dimensions

(in mm). H_s_: 13.9–16.5 (holotype 15.6), W_s_: 3.8–4.3 (holotype 4.3).

##### Type locality.

Greece, Epirus, Gramos Mts, southeastern slope of the Mt. Souflika, ca. 1700 m.

##### Type material.

Type locality, leg. HS, vi.1985, holotype (NHMW 84024a), paratypes (NHMW 84024/3); ridge NNW of the Epano Arena, ca. 2000 m, leg. HS, 4.vii.1985, paratypes (NHMW 84025/9).

##### Other material.

Greece, Epirus, Gramos Mts, W of the Epano Arena summit, 2000 m, 40.3090°N, 20.8981°E, leg. ZE, ZF, JG, 27.vi.2013 (HNHM 99524); NW of the Epano Arena summit, 2060 m, 40.3115°N, 20.8986°E, leg. ZE, ZF, JG, 27.vi.2013 (HNHM 99525); ridge N of the Epano Arena summit, 2120 m, 40.3131°N, 20.8995°E, leg. ZE, ZF, JG, 27.vi.2013 (HNHM 99526).

##### Distribution.

Gramos Mts in northwestern Greece. Known from the Epano Arena area where it is common. Sympatric, and in some sites syntopic, with the much rarer *Montenegrina grammica grammica* (Fig. 31A).

#### Montenegrina skipetarica puskasi ssp. n.

Taxon classificationAnimaliaStylommatophoraClausiliidae

http://zoobank.org/F738111B-2275-4D1F-867D-5A587966819B

Fig. 30A

##### Diagnosis.

Medium-size subspecies with very narrowly attached peristome, dorsolateral lunella and often uninterrupted plica superior with long anterior part.

##### Description.

The shell is medium- to large-sized, light brownish-corneous, somewhat tumid, of its 9^1^/_2_ to 11 whorls the lower three are almost almost of the same width. The surface is smooth at the lower whorls but becomes indistinctly costate over the short apex. The weakly inflexed neck has dense, sharp ribs behind the peristome. The basal crest is weak, the peripheral one is well visible. The light brown, ovoid peristome has simple, narrowly attached (rarely somewhat detached) margin. The lamellae superior and spiralis overlap. The lamella inferior is emerged, well visible in front view. The broadly bent lamella subcolumellaris is not visible in frontal, only in slanted view at the aperture. The lunella is dorsolateral, broad, mostly fused to the basalis that is as long as the subclaustralis. The sulcalis is week. The anterior part of the plica superior is long, ends in a lump behind the aperture and often also reaches the lunella complex. Part of the clausilium plate is visible through the aperture.

##### Dimensions

(in mm). Holotype H_s_: 18.9, W_s_: 5.2, H_a_: 4.8, W_a_: 4.05; paratypes H_s_: 16.8–19.7 (mean 18.7, SD 0.76), W_s_: 4.5–5.4 (mean 4.9, SD 0.28), H_a_: 4.4–5.3, W_a_: 3.5–4.2 (n = 12).

##### Differential diagnosis.

Distinguishable from *Montenegrina skipetarica ersekensis* and *Montenegrina skipetarica nobilis* by the retracted, frontally not visible lamella subcolumellaris, and from all other *Montenegrina skipetarica* subspecies by the cylindical lower whors that are nearly of the same width. From the nearby-occurring and apparently closest related *Montenegrina skipetarica gurelurensis* ssp. n. differs by the smooth shell and attached peristome.

##### Type locality.

Albania, Dibrë District, Arras, 2 km from the Kukës to Peshkopi road toward Cidhnë, 460 m, 41.7391°N, 20.2970°E.

##### Type material.

Type locality, leg. TD, ZE, ZF, DM, 10.x.2005, holotype (HNHM 99448), paratypes (HNHM 99449/32, MMM-B01318/32, ER/32, SZ/4); type locality, leg. ZE, ZF, AH, DM, 12.iv.2006 (HNHM 99450/7, ER/7, HU/7); type locality, leg. ZE, ZF, JG, 1.vii.2015, (HNHM 99451/18, NHMW 111206/18, GR/18).

##### Other material.

Albania, Dibrë District, beneath Cidhnë, gorge of the Setë Stream at the lower hydroelectric station, 510 m, 41.7506°N, 20.2626°E, leg. TD, ZF, 10.x.2005 (HNHM 99452, MMM-B01319); 1 km W of Cidhnë, along the footpath to Gurë-Lurë, gorge of the Setë Stream, 680 m, 41.7518°N, 20.2611°E, leg. TD, ZE, ZF, DM, 10.x.2005 (HNHM 99453, MMM-B01320); same locality, leg. ZE, ZF, AH, DM, 12.iv.2006 (HNHM 99455); ca. 1.5 km W of Cidhnë, 41.7528°N, 20.2505°E, leg. PJ, TK, DM, GP, 8.x.2012, (HNHM 99457); ca. 2 km W of Cidhnë, 730-750 m, leg. ZE, DM, 10.x.2005, (HNHM 99454, MMM-B01321); 0.1–1.5 km W of Cidhnë, leg. ZE, ZF, JG, 1.vii.2015 (HNHM 99458, NHMW 111207); Mat district, Mali i Dejës, limestone hill of 1700 m height above the headwaters of the Lumi i Varoshit, 1540 m, 41.6746°N, 20.2112°E, leg. ZF, DM, ZU, 18.v.2010 (HNHM 99691).

##### Etymology.

The new taxon is named after Gellért Puskás, entomologist (HNHM), who collected invaluable mollusc material during his Balkan field trips.

##### Distribution.

Southern part of the Lura-Dejë mountain group in northern Albania. This taxon was found at some nearby localities along the Setë Stream (Fig. 31B).

#### Montenegrina skipetarica remota

Taxon classificationAnimaliaStylommatophoraClausiliidae

Fehér & Szekeres, 2006

Fig. 30I

Montenegrina irmengardis remota Fehér & Szekeres, 2006 in Erőss et al. 2006: 192–194, fig. 13.
Montenegrina skipetarica remota – Nordsieck 2009: 73, plate 1, fig. 2.

##### Diagnosis.

Shell large, tumid, violet-brown with whitish suture. Lower whorls smooth, upper whorls finely costate. Neck not to barely inflexed, irregularly wrinkled-costare. Basal and peripheral crests weak. Peristome attached, ovoid to somewhat quadrangular, with simple margin. Lamellae superior and spiralis mostly do not overlap. In front view lamella inferior well emerged, broadly-bent subcolumellaris not visible. Lunella dorsal-dorsolateral, connected to the short basalis. Subclaustralis also short, sulcalis weak to residual. Anterior plica superior absent or week and separate from the lunella complex.

##### Dimensions

(in mm). H_s_: 18.0–23.1 (holotype 19.6), W_s_: 4.7–5.6 (holotype 5.4).

##### Type locality.

Albania, gorge of the Mat River, 11 km W of the Ulëz junction, along the Burrel to Milot road, 100 m, 41.6919°N, 19.8318°E.

##### Type material.

Type locality, leg. ZE, ZF, 27.vi.2003, holotype (HNHM 94859), paratypes (HNHM 94860/6, NHMW 103281, SMF 328085); same locality, leg. ZF, 8.x.2004, paratypes (HNHM 94861/3).

##### Other material.

Type locality, leg. ZE, ZF, AH, DM, 14.iv.2006 (HNHM 96812); same locality, leg. ZF, TN, EM, 16.iv.2014 (HNHM 99021).

##### Distribution.

Lower valley of the Mat River. Known only from the type locality (Fig. 31B).

#### Montenegrina skipetarica rugosa

Taxon classificationAnimaliaStylommatophoraClausiliidae

Fehér & Szekeres, 2006

Fig. 30G

Montenegrina irmengardis rugosa Fehér & Szekeres, 2006 in Erőss et al. 2006: 194, fig. 14.
Montenegrina skipetarica rugosa – Nordsieck 2009: 73.

##### Diagnosis.

Shell medium, somewhat conical, light reddish-brown. Whorls with wide-spaced, indistinct wrinkle-ribs. Neck inflexed, sharper and more densely costate. Basal and peripheral crests well recognizable. Peristome attached, ovoid, with simple margin. Lamellae superior and spiralis overlap. In front view lamella inferior well emerged, subcolumellaris mostly visible. Lunella dorsal to dorsolateral, fused to the short basalis. Subclaustralis weak, sulcalis well developed. Anterior plica superior mostly separate from the lunella complex.

##### Dimensions

(in mm). H_s_: 16.0–18.7 (holotype 17.4), W_s_: 4.0–4.4 (holotype 4.4).

##### Type locality.

Albania, Çorovodë District, Mali i Tomorrit, 4.8 km NE of Çorovodë (toward Radesh), gorge of the Përroi i Çorovodës, 480 m, 40.5223°N, 20.2574°E.

##### Type material.

Type locality, leg. ZF, JK, DM, 10.x.2004, holotype (HNHM 94862), paratypes (HNHM 94863/3, NHMW 103282, SMF 328086).

##### Other material.

Type locality, leg. ZF, AH, TH, DM, 22.viii.2006 (HNHM 99540); Qafa e Dëvris, NE of Radesh along the road to Zaloshnje, E side of the gorge, 1150 m, 40.5553°N, 20.2733°E, leg. ZB, ZF, CN, DP, 8.viii.2004 (HNHM 94864); same locality, leg. ZF, AH, TH, DM, 22.viii.2006 (HNHM 99538); 4.5 km NE of Turbehovë, gorge of the Përroi i Krishovës, 1040 m, 40.5590°N, 20.3908°E, leg. ZF, AH, TH, DM, 23.viii.2006 (HNHM 99539); ca. 15 km NE of Çorovodë, at the Gradec-junction, 1000 m, 40.5435°N, 20.2718°E, leg. ZF, AH, TH, DM, 22.viii.2006 (HNHM 99542); 1.5 km N of Qafa e Dëvris, along the road to Zaloshnje, 1110 m, 40.5714°N, 20.2880°E, leg. ZF, AH, TH, DM, 23.viii.2006 (HNHM 99543).

##### Distribution.

Ostrovica, Kulmak and Tomorr Mts in Southern Albania (Fig. 31A).

##### Remarks.

This taxon shows high inter-population variability of the shell sculpture, ranging from indistinctly wrinkled to sharply ribbed.

#### Montenegrina skipetarica thysi

Taxon classificationAnimaliaStylommatophoraClausiliidae

Loosjes & Loosjes-van Bemmel, 1988

Fig. 30D

Montenegrina thysi Loosjes & Loosjes-van Bemmel, 1988: 189–191, fig. 1.
Montenegrina skipetarica thysi – Nordsieck 2009: 73.

##### Diagnosis.

Shell large, tumid, light reddish-brown with whitish tint. Whorls with strong, often whitish ribs, which become less distinct toward the apex. Neck weakly inflexed, densely costate behind the aperture. Basal and peripheral crests are recognizable. Peristome attached, ovoid to angular, with simple margin. Lamellae superior and spiralis mostly overlap. In front view lamella inferior moderately emerged, subcolumellaris not or barely visible. Lunella dorsal-dorsolateral, fused to the strong basalis. Subclaustralis also strong, sulcalis weak, anterior plica superior long, often connected to the lunella complex.

##### Dimensions

(in mm). H_s_: 22.5, W_s_: 6.1 (mean values).

##### Type locality.

Greece, Epirus, Tymfi Mts, Vikos Gorge near the Agia Paraskevi Monastery.

##### Type material.

Type locality, leg. Loosjes, 26–27.vi.1964, holotype and 2 paratypes (RMNH).

##### Other material.

Type locality, 1030 m, 39.8910°N, 20.7538°E, leg. ZE, ZF, JG, 25.vi.2013 (HNHM 99532).

##### Distribution.

Tymfi Mts in Epirus, northwestern Greece. Known only from the type locality (Fig. 31A).

#### Montenegrina skipetarica voidomatis

Taxon classificationAnimaliaStylommatophoraClausiliidae

Nordsieck, 1974

Fig. 30C

Montenegrina irmengardis voidomatis Nordsieck, 1974: 155, plate 6, fig. 38. – Zilch 1981: 129, plate 15, fig. 42.
Montenegrina skipetarica voidomatis – Nordsieck 2009: 73.

##### Diagnosis.

Shell large, tumid, light reddish-brown with whitish suture. All whorls smooth. Neck not or only barely inflexed, finely and densely costate. Basal crest weak, peripheral crest not recognizable. Peristome attached, ovoid, with simple margin. Lamellae superior and spiralis mostly do not overlap. In front view lamella inferior well emerged, subcolumellaris mostly hidden. Lunella dorsal-dorsolateral, fused to the short basalis. Subclaustralis and sulcalis weak. Anterior plica superior mostly separate from the lunella complex.

##### Dimensions

(in mm). H_s_: 19.8–23.0 (holotype 20.6), W_s_: 5.4–6.3 (holotype 5.8).

##### Type locality.

Greece, Epirus, gorge of the Voidomatis near Konitsa, upstream of the Turkish bridge.

##### Type material.

Type locality, leg. Fauer, 12.viii.1972, holotype (SMF 227688), paratypes (SMF 227689, SMNS-N 6242).

##### Other material.

Greece, Epirus, Ioannina District, 2 km S of Kleidonia, old bridge of Voidomatis River, 420 m, 39.9674°N, 20.6646°E, (near type locality), leg. ZE, ZF, JG, 26.vi.2013 (HNHM 99531); Tymfi Mts, Vikos Gorge at the Oxia viewpoint, 1330 m, 39.9067°N, 20.7519°E, leg. ZE, ZF, JG, 25.vi.2013 (HNHM 99529); Kapesovo S 6 km, Kokkori Bridge, 740 m, 39.8620°N, 20.7749°E, leg. ZE, ZF, JG, 26.vi.2013 (HNHM 99530); Kipi, old bridge, leg. HS, 21.viii.1990 (NHMW 102872/2).

##### Distribution.

Southern part of the Tymfi Mts in Epirus, northwestern Greece, within the catchment area of the Voidomatis River (Fig. 31A).

#### Montenegrina soosi

Taxon classificationAnimaliaStylommatophoraClausiliidae

Erőss & Szekeres, 2006

Fig. 28H

Montenegrina janinensis soosi Erőss & Szekeres, 2006 in Erőss et al. 2006: 197, fig. 18. – Nordsieck 2009: 75.

##### Diagnosis.

Shell small, light yellowish corneous. All whorls smooth. Neck inflexed, densely striate-costate. Basal and peripheral crests well recognizable. Peristome attached, ovoid to angular, with simple margin. Lamellae superior and spiralis long overlap. In front view lamella inferior moderately emerged, medium-bent subcolumellaris visible. Lunella broad, dorsolateral-lateral to lateral, mostly not fused to the basalis. Subclaustralis residual, sulcalis strong. Plica superior fused to the principalis. Anterior plica superior long, mostly separate from the lunella complex. Clausilium plate not visible through the aperture.

##### Dimensions

(in mm). H_s_: 11.4-14.6 (holotype 14.6), W_s_: 2.7–3.2 (holotype 3.2).

##### Type locality.

Albania, Kukës District, between Kolesjan and Resk, 1 km N of the Ploshtan junction, along the Kukës to Peshkopi road, 750 m, 41.9598°N, 20.3938°E.

##### Type material.

Type locality, leg. ZE, ZF, JK, DM, 25.vi.2003, holotype (HNHM 94868), paratypes (NHMUK 20050224, HNC 63188, HNHM 94869/23, NHMW 103285, RMNH 100317, SMF 328089).

##### Other material.

Type locality, leg. TD, ZE, ZF, DM, 9.x.2005 (HNHM 96825); 1.8 km S of the Kolesjan junction, 41.9613°N, 20.3955°E, leg. AR, NR, PR, ix.2013 (NHMW 110430/MN/0112); 7.4 km S of the Kolesjan junction, 41.9260°N, 20.3872°E, leg. AR, NR, PR, ix.2013 (NHMW 110430/MN/0113); at the mouth of the Përroi i Bushtricës, 300 m, 41.9352°N, 20.3632°E, leg. TD, ZE, ZF, DM, 9.x.2005 (HNHM 99637); same locality, leg. ZE, ZF, JG, 2.vii.2015 (NHMW 110430/MN/0116); 1.3 km S of the mouth of the Përroi i Bushtricës, 400 m, 41.9254°N, 20.3569°E, leg. ZE, ZF, JG, 2.vii.2015 (NHMW 110430/MN/0115); bridge of the Lumi i Drinit te Zi (between Skavicë and Tej-Drinit), 320 m, 41.9237°N, 20.3539°E, leg. TD, ZE, ZF, DM, 9.x.2005 (HNHM 99644); bridge of the Lumi i Drinit te Zi (between Draj-Reç and Zall-Reç), 310 m, 41.8880°N, 20.3165°E, leg. TD, ZE, ZF, DM, 9.x.2005 (HNHM 99640); same locality, leg. ZE, ZF, JG, 2.vii.2015 (NHMW 110430/MN/0114); Tej-Drinit, 4.5 km from the bridge of the Lumi i Drinit te Zi, 620 m, 41.9423°N, 20.3593°E, leg. TD, ZE, ZF, DM, 9.x.2005 (HNHM 99641); 2.5 km N of Bushtricë, gorge at the Ura e Lapavës, 600 m, 41.8949°N, 20.4169°E, leg. ZE, ZF, JK, DM, 25.vi.2003 (HNHM 94870); same locality, leg. LD, ZE, ZF, AH, DM, 26.vi.2007 (HNHM 99642); 1 km S of Resk along the Kukës to Peshkopi road, 690 m, 41.9260°N, 20.3871°E, leg. LD, ZE, ZF, AH, DM, 26.vi.2007 (HNHM 99643); Arrën, 1150 m, 41.9242°N, 20.2813°E, leg. TD, ZE, ZF, DM, 9.x.2005 (HNHM 99639); Gurri i Shkallës, E of Arrën, 1300 m, 41.9188°N, 20.2693°E, leg. TD, ZE, ZF, DM, 9.x.2005 (HNHM 99638).

##### Distribution.

**Figure 32.**
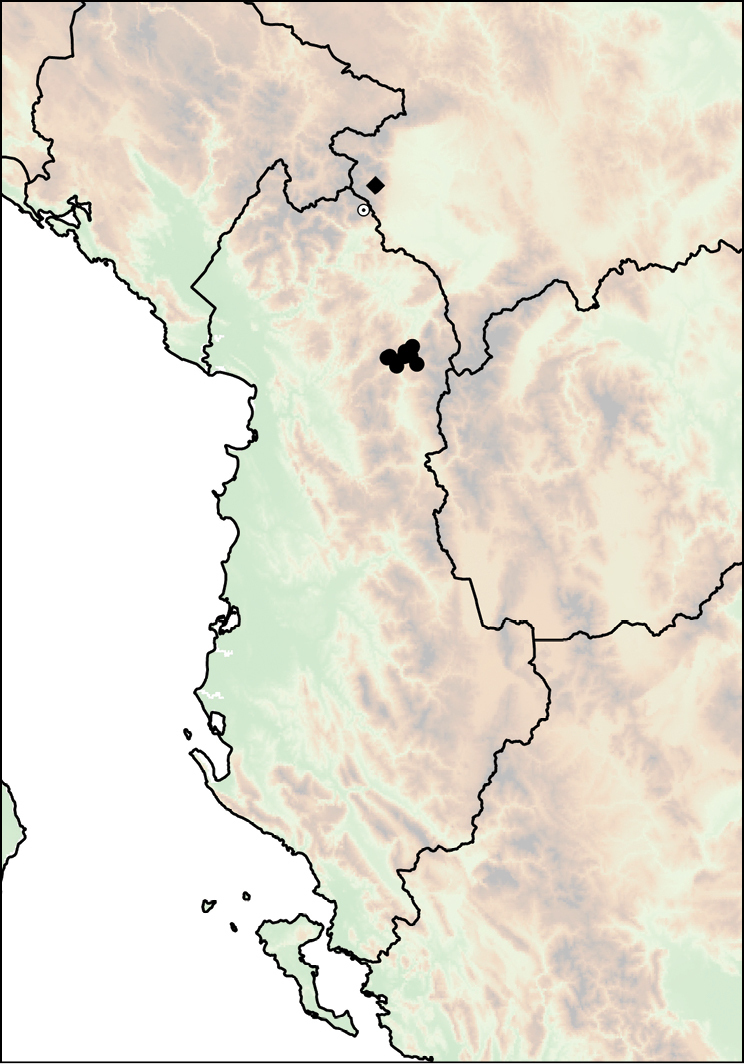
Distribution of *Montenegrina soosi* and *Montenegrina sporadica*. *Montenegrina soosi* (circle); *Montenegrina sporadica sporadica* (diamond); *Montenegrina sporadica tropojana* ssp. n. (empty circle with dot).

This taxon is distributed in the catchment area of the Drin i Zi (Black Drin) between the Mirditë and Mt. Gjalica e Lumës (Fig. 32).

##### Remarks.

As its shell does not reveal a clear affiliation to any other taxa, *Montenegrina soosi* is tentatively regarded as a distinct species. In the Lapavë Gorge it occurs syntopically with the nominotypical form of *Montenegrina skipetarica*.

#### Montenegrina sporadica

Taxon classificationAnimaliaStylommatophoraClausiliidae

Nordsieck, 1974

##### Diagnosis.

Shell small, light corneous. Lower whorls smooth, upper ones smooth to indistinctly wrinkled-costate. Neck inflexed, striate. Basal and peripheral crests weak. Peristome attached, ovoid to somewhat angular, with slightly swollen margin. In front view lamella inferior moderately emerged, medium-bent subcolumellaris visible or hidden. Lunella dorsolateral to dorsolateral-lateral. Basalis absent or weak and not fused to the lunella, subclaustralis absent. Anterior plica superior mostly missing, if present separate from the lunella complex. The clausilium plate is partly visible through the aperture. Differs from *Montenegrina janinensis* by the stronger sculpture, weaker-bent lamella subcolumellaris, and the lack of anterior plica superior.

#### Montenegrina sporadica sporadica

Taxon classificationAnimaliaStylommatophoraClausiliidae

Nordsieck, 1974

Fig. 28J

Montenegrina janinensis sporadica Nordsieck, 1974: 151–152, plate 5, fig. 29. – Zilch 1981: 129, plate 13, fig. 28. – Nordsieck 2009: 75.

##### Diagnosis.

Lower whorls smooth, upper ones indistinctly wrinkled-costate. Lamellae superior and spiralis slightly overlap. In front view lamella subcolumellaris visible. Lunella dorsolateral, not fused to the weak basalis. Subclaustralis, sulcalis and anterior plica superior absent.

##### Dimensions

(in mm). H_s_: 12.8–14.9 (holotype 13.5), W_s_: 3.2–3.7 (holotype 3.6).

##### Type locality.

Kosovo, valley of the Bistrica (= Dečanska Bistrica) upstream of the Dečani Monastery, left bank.

##### Type material.

Type locality, leg. Nordsieck, 11.viii.1970, holotype (SMF 227678), paratypes (SMF 227679, SMNS-N 4925).

##### Distribution.

Bistrica Valley, south of the Mt. Streocka, eastern part of the Prokletije. Known only from the type locality (Fig. 32).

#### Montenegrina sporadica tropojana ssp. n.

Taxon classificationAnimaliaStylommatophoraClausiliidae

http://zoobank.org/0E484600-6245-4220-82C6-17582464215E

Fig. 28K

##### Diagnosis.

Small, elongate subspecies with weakly inflexed neck, retracted lamella subcolumellaris, missing basalis and subclaustralis.

##### Description.

The small, elongate, light brownish-corneous shell has 9½ to 11 whorls. The lower three whorls are nearly of the same width. The entire surface of the shell is smooth, except the finely striate-costate neck. The neck is weakly inflexed, the basal and peripheral crests are also wealky developed. The light brownish, simple peristome is ovoid to somewhat angular, broadly attached, its upper columellar margin is missing. The lamellae superior and spiralis do not overlap. The moderately emerged terminal part of the lamella inferior descends in a straight line. In front view the medium-bent lamella subcolumellaris is not visible. The plica principalis is often fused to the superior. The broad lunella is dorsolateral-lateral to lateral. The plicae basalis and subclaustralis are absent. The sulcalis is strong. The anterior part of the plica superior is missing or weak, separate from the lunella complex. The clausilium plate is barely visible through the aperture.

##### Dimensions

(in mm). Holotype H_s_: 13.9, W_s_: 3.4, H_a_: 3.2, W_a_: 2.5; paratypes (NHMW 111225, n = 12): H_s_: 13.0–16.0 (mean 14.9, S.D. 0.94), W_s_: 3.1–3.8 (mean 3.4, S.D. 0.22), H_a_: 3.0–3.6, W_a_: 2.2–3.7.

##### Differential diagnosis.

The new subspecies differs from *Montenegrina sporadica sporadica* by its more elongate and smoother shell, retracted lamella subcolumellaris, deeper lunella, and the occasional presence of the anterior plica superior.

##### Type locality.

Albania, Tropojë District, gorge of the Përroi i Tropojës, ca. 14 km N of Tropojë, 970 m, 42.4740°N, 20.1520°E.

##### Type material.

Type locality, leg. DA, ZF, JG, 26.vi.2014, holotype (NHMW 111224), paratypes (NHMW 111225/4+9rf, HNHM 99493/9, GR/10, SZ/2); ca. 13 km N of Tropojë, 930 m, 42.4692°N, 20.1554°E, leg. DA, ZF, JG, 26.vi.2014, paratypes (NHMW 111226/4+26aj, HNHM 99494/3, SZ/1); Gash region, upper valley of the Përroi i Tropojës, 1100 m, 42.4749°N, 20.1518°E, leg. ZB, Lunk, DP, Schmidt, 6.viii.2009, paratype (HNHM 99495/1).

##### Etymology.

The new subspecies is named after the Tropojë Stream, in the gorge of which the type locality is located.

##### Distribution.

Mt. Shkëlzen in the eastern part of the Prokletije in northeastern Albania. Known only from the limestone gorge of the Tropojë Stream (Fig. 32).

#### Montenegrina stankovici

Taxon classificationAnimaliaStylommatophoraClausiliidae

(Urbański, 1960)

Fig. 28I

Delima (Delima [Heteroptycha]) stankovići Urbański, 1960: 51–54, plate Ia–c, fig. 1.
Montenegrina stankovici – Nordsieck 1969: 259. (genital anatomy) – Nordsieck 1974: 135, plate 6, fig. 36. – Zilch 1981: 132. – Nordsieck 2009: 73.

##### Diagnosis.

Shell small to medium, conical, light corneous. Lower whorls smooth, upper ones finely, indistinctly costate. Neck weakly inflexed, finely striate, without recognizable crests. Peristome attached, ovoid, its simple margin is missing at the upper columellar side. Lamellae superior and spiralis do not or only barely overlap. In front view lamella inferior moderately emerged, almost straight descending subcolumellaris mostly not visible. Lunella dorsal to dorsolateral, with weak and diffuse lower part. Plica principalis short, all other plicae absent. Clausilium plate narrow, entirely visible through the aperture.

##### Dimensions

(in mm). H_s_: 13.9–16.6, W_s_: 4.0–4.5 (Nordsieck 1974).

##### Type locality.

Macedonia, Ohrid District, near Sveti Naum Monastery at the SE shore of the Ohrid Lake.

##### Type material.

Not known.

##### Other material.

Type locality, 40.914°N, 20.7406°E, leg. A. Fehér, T. Fehér, ZF, LT, 9.viii.2014 (NHMW 110430/MN/0060); same locality, leg. ZF, Kónya, 10.ix.1994 (HNHM 90879); same locality, leg. ZE, ZF, AH, 6.iv.2004 (HNHM 94328); N of Trpejca, 700 m, 40.9656°N, 20.7858°E, leg. ZE, ZF, AH, 6.iv.2004 (HNHM 94444); same locality, leg. A. Fehér, T. Fehér, ZF, LT, 21.viii.2011 (HNHM 99581); same locality, leg. A. Fehér, T. Fehér, ZF, LT, 9.viii.2014 (NHMW 110430/MN/0059); same locality, leg. ZE, ZF, JG, 29.vi.2015 (NHMW 110430/MN/0063); Trpejca, southern bay, 700 m, 40.9574°N, 20.7778°E, leg. ZF, EH, KJ, HS, 16.x.2014 (NHMW 110430/MN/0062); Ohrid, N foot of the castle hill, 700 m, 41.1147°N, 20.7876°E, leg. A. Fehér, T. Fehér, ZF, LT, 10.viii.2014 (NHMW 110430/MN/0061); ca. 2.5 km S of Peštani, 41.0°N, 20.8°E, leg. LP, PS, AS, 16.vii.1972 (HNHM 36729); Ohrid, Sveti Jovan Kaneo Monastery, leg. LP, PS, AS, 17.vii.1972 (HNHM 36730).

##### Distribution.

**Figure 33.**
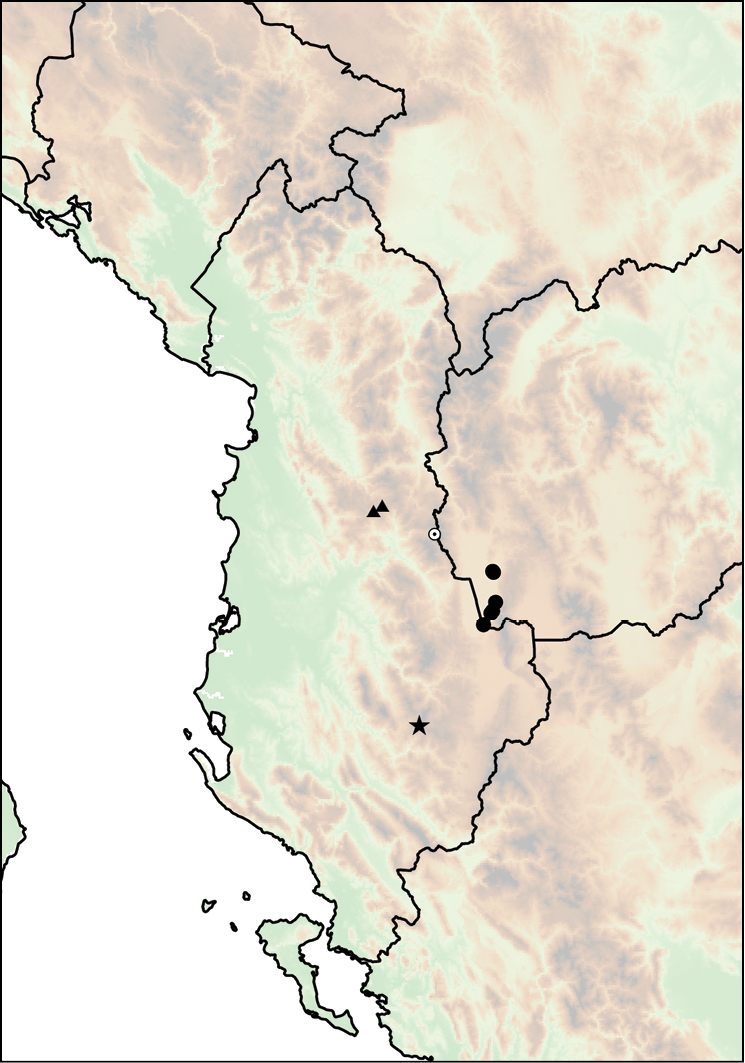
Distribution of *Montenegrina stankovici* and *Montenegrina sturanyana*. *Montenegrina stankovici* (circle); *Montenegrina sturanyana sturanyana* ssp. n. (empty circle with dot); *Montenegrina sturanyana gropana* ssp. n. (triangle); *Montenegrina sturanyana ostrovicensis* ssp. n. (star).

Eastern shore of the Ohrid Lake (Fig. 33).

##### Remarks.

Inhabits limestone cliffs along the shore of the Ohrid Lake, always very close to the water surface. This is the only known *Montenegrina* taxon that prefers such type of habitat. Occasionally it occurs together with, or in the vicinity of, *Montenegrina dofleini pinteri* or *Montenegrina perstriata ochridensis*, but in a strict sense is not syntopic with these. Apparent spatial segregation can be observed on a fine scale: its congeners prefer cliff regions farther from the water surface. This kind of niche partitioning is unique within the genus.

#### Montenegrina sturanyana sp. n.

Taxon classificationAnimaliaStylommatophoraClausiliidae

http://zoobank.org/28674C8F-6124-45D9-B2B4-4ECFE74CF88B

##### Diagnosis.

Shell small, glossy, light corneous. All whorls smooth. Neck weakly inflexed, striate to striate-costate. Basal crest weak to well developed, peripheral crest absent to recognizable. Peristome attached, ovoid to somewhat angular, its margin wide and swollen. In front view lamella inferior is weakly emerged, medium-bent subcolumellaris hidden or only barely visible. Lunella broad, dorsolateral to dorsolateral-lateral. Plica superior often fused to the principalis and can reach farther forward than its contact with the lunella. Basalis absent to short and separate from the lunella. Subclaustralis absent to residual, sulcalis of variable strength. Anterior plica superior absent or weak and not fused to the lunella complex. The clausilium plate is not or only barely visible through the aperture. Differs from *Montenegrina tomorosi* Brandt, 1961 by its slender stature, stronger basal crest, deeper lamella subcolumellaris and weaker subclaustralis, and from all other *Montenegrina* species by the combination of the dorsolateral to lateral lunella and strongly reduced basal plicae.

#### Montenegrina sturanyana sturanyana ssp. n.

Taxon classificationAnimaliaStylommatophoraClausiliidae

http://zoobank.org/241A20A6-5B5B-4B91-AE1D-A10F80FAFE3F

Fig. 34K

##### Diagnosis.

Small subspecies with non-overlapping lamellae superior and spiralis, deep-ending subcolumellaris, missing basalis and anterior plica superior.

##### Description.

The small, light corneous shell is comprised of 9½ to 10 whorls, of which the last three are of nearly the same width. The weakly inflexed neck is finely striate, otherwise the entire shell surface is smooth. The basal crest is weak, the peripheral one is not recognizable. The ovoid peristome is attached, its margin is wide and swollen. The lamellae superior and spiralis do not overlap. In front view the straight descending end of the lamella inferior is moderately emerged, the medium-bent subcolumellaris is not visible. The lunella is broad, dorsolateral-lateral. The plica superior occasionally reaches farther forward than its fusion with the lunella. Of the lower plicae the basalis is absent, the subclaustralis is residual, the sulcalis is well developed. The anterior plica superior is missing. The clausilium plate is not visible through the aperture.

##### Dimensions

(in mm). Holotype H_s_: 14.7, W_s_: 3.8, H_a_: 3.5, W_a_: 2.8; paratypes (HNHM 99506, HNHM99507, n = 5): H_s_: 14.6–15.2 (mean 14.8, S.D. 0.20), W_s_: 3.7–4.0 (mean 3.9, S.D. 0.10), H_a_: 3.3–3.6, W_a_: 2.3–2.9.

**Figure 34.**
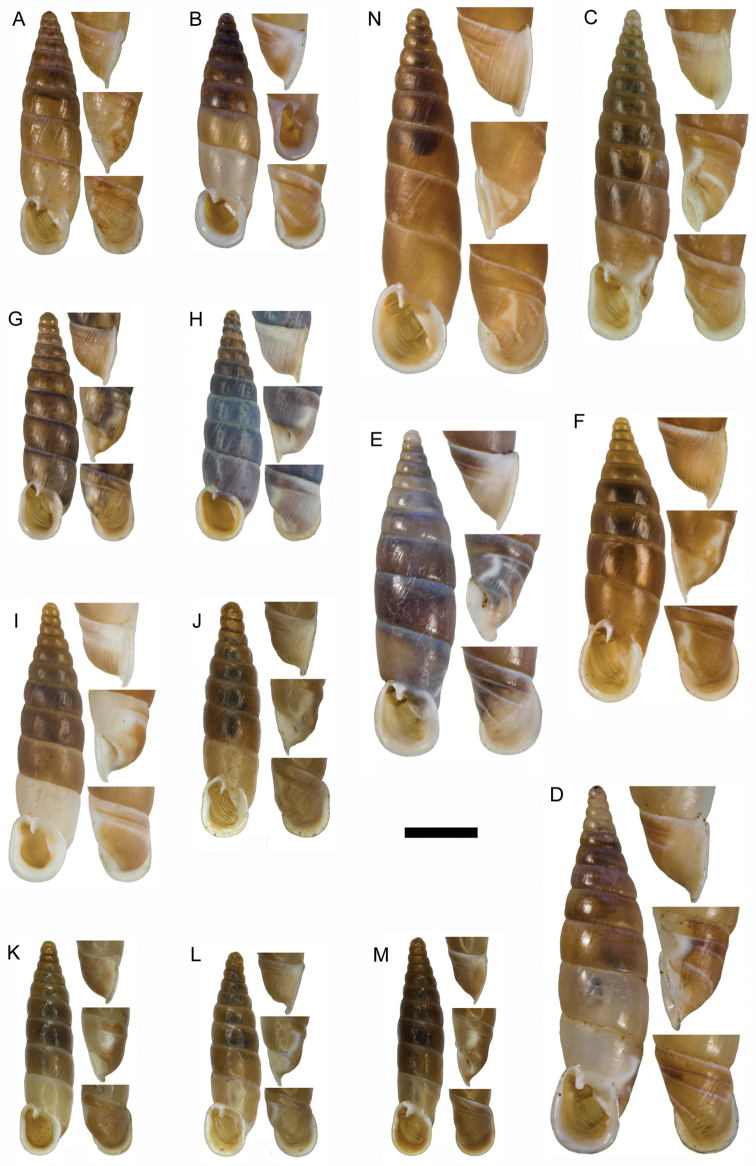
**A** *Montenegrina subcristata* (Pfeiffer, 1848), lectotype of *brunnea*, SMF 176308 **B** *Montenegrina subcristata* (Pfeiffer, 1848), holotype of *sublabiata*, SMF 176327 **C** *Montenegrina subcristata* (Pfeiffer, 1848), Popovo Cave near Njeguš, NHMW 38413 **D** *Montenegrina subcristata* (Pfeiffer, 1848), lectotype of *interior*, SMF 176323 **E** *Montenegrina subcristata* (Pfeiffer, 1848), lectotype of *wohlberedti*, SMF 176328 **F** *Montenegrina timeae* Erőss & Szekeres, 2006, holotype, HNHM 94855 **G** *Montenegrina tomorosi tomorosi* Brandt, 1961, holotype, SMF 163940 **H** *Montenegrina tomorosi coerulescens* Nordsieck, 1996, holotype, NHMW 89274 **I** *Montenegrina tomorosi ampla* Fehér & Szekeres, 2006, holotype, HNHM 94865 **J** *Montenegrina tomorosi hunyadii* ssp. n., holotype, HNHM 99510 **K** *Montenegrina sturanyana sturanyana* ssp. n., holotype, HNHM 99505 **L** *Montenegrina sturanyana ostrovicensis* ssp. n., holotype, HNHM 99500 **M** *Montenegrina sturanyana gropana* ssp. n., holotype, HNHM 99502 **N** *Montenegrina zilchi* Nordsieck, 1974, holotype, SMF 227690. Scale bar: 5 mm.

##### Differential diagnosis.

The new taxon differs from *Montenegrina sturanyana gropana* by its less elongate shell and the absence of the basalis, from *Montenegrina sturanyana ostrovicensis* by its deeper lunella, and from both subspecies by its finer neck sculpture, non-overlapping lamellae superior and spiralis, and missing anterior plica superior.

##### Type locality.

Albania, Librazhd District, Jablanica Mts, on Mt. Lapa, 5 km E of Qarishtë, 1960 m, 41.2505°N, 20.5034°E.

##### Type material.

Type locality, leg. ZB, DP, Vojtkó, 4.vii.2008, holotype (HNHM 99505), paratypes (HNHM 99506/3+1a, SZ/1); 5.4 km E of Qarishtë, 2010 m, 41.2498°N, 20.5044°E, leg. ZB, DP, Vojtkó, 4.vii.2008 (HNHM 99507/2).

##### Etymology.

The new species is dedicated to Rudolf Sturany (1867–1935), Austrian zoologist, former curator of the Mollusc Collection at NHMW, in appreciation of his pioneering contribution to the research of the Balkan fauna.

##### Distribution.

Western side of the Jablanica Mts in central eastern Albania. Known from two nearby localities (Fig. 33).

#### Montenegrina sturanyana gropana ssp. n.

Taxon classificationAnimaliaStylommatophoraClausiliidae

http://zoobank.org/2E6C1E2C-2A43-4A62-8C22-A1C4277B2E81

Fig. 34M

##### Diagnosis.

Small, slender subspecies with often overlapping lamellae superior and spiralis, and a basalis separate from the lunella complex.

##### Description.

The small, slender, light corneous shell consists of 9½ to 11½ whorls. The entire surface is smooth, except for the weakly inflexed neck that is finely striate-costate. The basal crest is well developed, the peripheral one is recognizable. The peristome is somewhat angular, attached, its margin is wide and swollen. The lamellae superior and spiralis often overlap. In front view the straight descending lamella inferior is moderately emerged. The retracted, medium-bent lamella subcolumellaris is only barely visible through the aperture. The broad, dorsolateral-lateral to lateral lunella is not fused to the short basalis. The plica superior can reach forward somewhat farther than its attachment to the lunella. The subclaustralis and sulcalis are residual. The anterior plica superior is absent, or weak, parallel to the principalis, separate from the lunella complex. The clausilium plate cannot be viewed through the aperture.

##### Dimensions

(in mm). Holotype H_s_: 14.9, W_s_: 3.6, H_a_: 3.7, W_a_: 2.8; paratypes (HNHM 99503, n = 12): H_s_: 13.2–16.3 (mean 14.7, S.D. 1.00), W_s_: 3.0–3.7 (mean 3.4, S.D. 0.24), H_a_: 3.0–3.7, W_a_: 2.3–2.9.

##### Differential diagnosis.

The new subspecies can be distinguished from *Montenegrina sturanyana sturanyana* by the presence of an anterior plica superior, from *Montenegrina sturanyana ostrovicensis* by its deeper lunella, and from both taxa by the slender shell and the presence of the basalis.

##### Type locality.

Albania, Tiranë District, Gropa Mts, Bizë, gorge of the Kaprol Stream near a military camp, 1250 m, 41.3392°N, 20.1989°E.

##### Type material.

Type locality, leg. ZF, TK, DM, 20.vi.2012, holotype (HNHM 99502), paratypes (HNHM 99503/75+7a+13aj, NHMW 111230/5); Bulqizë District, Çermenikë Mts, between Bizë and Ballenjë, near a cave, 1370 m, 41.3604°N, 20.2412°E, leg. ZF, TK, DM, 20.vi.2012, paratypes (HNHM 99504/25+11a+3aj, NHMW 111229/4, SZ/2).

##### Etymology.

The new taxon is named after the Gropa Mts in Central Albania.

##### Distribution.

Gropa-Bizë-Martanesh mountain group in Central Albania. Known from two nearby sites around the village Bizë in Central Albania (Fig. 33).

#### Montenegrina sturanyana ostrovicensis ssp. n.

Taxon classificationAnimaliaStylommatophoraClausiliidae

http://zoobank.org/7B143772-E043-4889-8727-823073D4D9F0

Fig. 34L

##### Diagnosis.

Small subspecies with often overlapping lamellae superior and spiralis, dorsolateral lunella, and missing basalis.

##### Description.

The small, light corneous shell is comprised of 8½ to 10 whorls, of which the last three are of comparable width. Except for the finely striate-costate neck the entire shell is smooth. The neck is weakly inflexed, the basal and peripheral crests are recognizable. The ovoid to somewhat angular peristome is attached, its margin is wide and swollen. The lamellae superior and spiralis often overlap. In front view the straight descending lamella inferior is weakly emerged. Through the aperture the medium-bent subcolumellaris can only be seen at an angle. The broad lunella is dorsolateral. The plica superior can reach forward farther than its fusion with the lunella. The basalis is absent, the subclaustralis is also absent or residual. The sulcalis is well developed. The anterior part of the plica superior is missing or weak, parallel to the principalis, separate from the lunella complex. The parietal edge of the clausilium plate is visible through the aperture.

##### Dimensions

(in mm). Holotype H_s_: 14.0, W_s_: 3.8, H_a_: 3.7, W_a_: 3.0; paratypes (HNHM 99501, n = 12): H_s_: 11.9–16.6 (mean 14.0, S.D. 1.27), W_s_: 3.7–4.4 (mean 4.0, S.D. 0.21), H_a_: 3.0–4.0, W_a_: 2.8–3.3.

##### Differential diagnosis.

The new subspecies differs from *Montenegrina sturanyana sturanyana* by the presence of the anterior plica superior, from *Montenegrina sturanyana gropana* by its less elongate shell and missing basalis, whereas from both of these by its less deeply positioned lunella.

##### Type locality.

Albania, Skrapar District, Mali i Ostrovicës, Maja e Faqekuqit, summit region, 2340 m, 40.5325°N, 20.4256°E.

##### Type material.

Type locality, leg. ZF, AH, TH, DM, 21.viii.2006, holotype (HNHM 99500), paratypes (HNHM 99501/32, NHMW 111228/4, HU/34, SZ/3).

##### Etymology.

The new taxon is named after the Ostrovica Mts.

##### Distribution.

Ostrovica Mts in Southern Albania. Known only from the type locality (Fig. 33).

#### Montenegrina subcristata

Taxon classificationAnimaliaStylommatophoraClausiliidae

(Pfeiffer, 1848)

Fig. 34A–E

Clausilia subcristata Pfeiffer, 1848: 438. – Küster 1844-1862: 39–40, plate 4, figs 10–13. – Schmidt 1868: 70.
Clausilia cattaroënsis (partim) – Walderdorff 1864: 509.
Clausilia cattaroensis var. minor Boettger, 1877b: 66.
Clausilia (Herilla) klecaki (sic!) Westerlund, 1881: 55.
Clausilia subcristata Clausilia (Delima) subcristata – Westerlund 1884: 54. – Clessin 1885: 182. – Wohlberedt 1907: 551–552. – Wohlberedt 1909: 675, plate 14, figs 158–161.
Clausilia (Delima) kleciaki – Westerlund 1884: 54.
Clausilia (Delima) wohlberedti Möllendorff, 1899: 169–170.
Clausilia (Delima) wohlberedti var. – Möllendorff 1899: 170.
Delima wohlberedti var. sublabiata v. Möll. – Wohlberedt 1901: 198, 206. (*nomen nudum*)
Clausilia (Delima) kleciaki var. brunnea Boettger 1907 in Wohlberedt 1907: 553. – Wohlberedt 1909: 676–677.
Clausilia (Delima) subcristata var. interior Boettger in Wohlberedt 1907: 552. – Wohlberedt 1909: 675, plate 14, fig. 162–163.
Clausilia (Delima) subcristata var. sublabiata Wohlberedt 1907: 553. – Wohlberedt 1909: 676.
Clausilia (Delima) subcristata f. minor – Wohlberedt 1907: 552.
Clausilia (Delima) subcristata wohlberedti – Wohlberedt 1907: 552–553. – Wohlberedt 1909: 675–676, plate 14, figs 164–169.
Delima (Delima) cattaroensis – Sturany and Wagner 1915: 73.
Delima (Delima) cattaroensis kleciaki – Sturany and Wagner 1915: 73.
Delima (Albanodelima) subcristata – Wagner 1924: 118. – Wagner 1925: 67, plate 2, fig. 23. (genital anatomy).
Delima (Albanodelima) kleciaki – Wagner 1924: 118. – Wagner 1925: 67, plate 3, fig. 26. (genital anatomy).
Delima (Albanodelima) subcristata wohlberedti – Wagner 1924: 119.
Delima (Albanodelima) subcristata interior – Wagner 1924: 119.
Clausilia laxa – Wagner 1924: 119.
Montenegrina subcristata – Nordsieck 1969: 259. (genital anatomy).
Montenegrina subcristata subcristata – Zilch 1981: 131, plate 13, fig. 19. – Nordsieck 2009: 73.
Montenegrina subcristata wohlberedti – Zilch 1981: 131, plate 13, figs 20–22. – Nordsieck 2009: 73.

##### Diagnosis.

Shell small to large, often ventricose, light corneous. All whorls smooth. Neck weakly inflexed, finely striate to moderately costate. Basal crest strong, peripheral crest well recognizable. Peristome attached, ovoid to angular, with somewhat swollen margin. Lamellae superior and spiralis do not or only barely overlap. In front view lamella inferior well emerged, medium-bent subcolumellaris mostly visible. Lunella lateral to ventrolateral, separate from the long to very long basalis. Subclaustralis is straight continuation of the lunella. Sulcalis strong. Long and strong anterior plica superior diverges from the principalis, remains separate from the lunella complex.

##### Dimensions

(in mm). H_s_: 13.7–25.3 (lectotype *brunnea* 17.0, lectotype *interior* 25.3, holotype *sublabiata* 16.5, lectotype *wohlberedti* 22.8), W_s_: 4.2–6.7 (lectotype *brunnea* 4.5, lectotype *interior* 6.7, holotype *sublabiata* 4.8, lectotype *wohlberedti* 5.8) (SMF 176323, SMF 176328, SMF 176308, SMF 176327, NHMW 110.430/MN/0138, NHMW 110.430/MN/0144, NHMW 110.430/MN/0145, HNMW 110430/MN/0119, NHMW 110430/MN/0157, HNHM 99489, NHMW 110.430/MN/0140, NHMW 38413, NHMW 110.430/MN/0143).

##### Type locality.

“in montibus Montenegrinis, 3000’ supra mare” (Pfeiffer 1848), more precisely by the collector (Küster 1844-1862): “Grenzgebirge Montenegro’s gegen Cattaro” = Montenegro, E of the Bay of Kotor, ca. 900 m [*subcristata*]; “Albanien” [*minor*]; Niksič (due to subsequent lectotype designation) [*interior*]; “Dalmatia ad Cattaro” [*kleciaki*]; “Sabljach” = Montenegro, Žabljak Crnojevića (due to subsequent lectotype designation) [*wohlberedti*]; “Wirbasar” = Montenegro, Virpazar (in: Möllendorff 1899) [*sublabiata*]; “Skadar kastel” = Albania, Shkodër fortress (due to subsequent lectotype designation) [*brunnea*].

##### Type material.

Niksič, ex Boettger, ex. Wohlberedt, 1906, lectotype [*interior*] (SMF 176323), paralectotypes [*interior*] (SMF 176324/5); “zw. Rijeka und Komarno”, ex Boettger, ex Wohlberedt 1906, paralectotype [*interior*] (SMF 176325); “Rijeka Quelle”, ex Boettger, ex Wohlberedt 1906, paralectotype [*interior*] (SMF 176326); “Sabljack”, ex Möllendorff, ex Wohlberedt, lectotype [*wohlberedti*] (SMF 176328), paralectotypes [*wohlberedti*] (SMF 176329/11); “Festung Skutari”, ex Boettger, ex Nägele, lectotype [*brunnea*] (SMF 176308), paralectotypes [*brunnea*] (SMF 176309/6); “Tarabos-Gebirge, Skutari”, ex Boettger, ex Wohlberedt 1906, paralectotypes [*brunnea*] (SMF 176310/6); Mikulić, ex Boettger, ex Wohlberedt 1906, paralectotype [*brunnea*] (SMF 176311); “Dalmatien, Praesieka”, ex Boettger, paralectotype [*brunnea*] (SMF 176312); “Virbazar”, ex Möllendorff, ex Wohlberedt, holotype [*sublabiata*] (SMF 176327).

##### Other material.

Montenegro, Zatrijebac, leg. Sturany, 24.iv.1905 (NHMW 43362); “Mužura Planina bei Dulcigno” (= Ulcinj), leg. Winneguth, 1904 (NHMW 41258); Rumija Mts, Đuravci, 340 m, 42.1677°N, 19.1959°E, leg. ZF, TK, DM, 16.vi.2012 (HNHM 99484); 12 km S of Virpazar, along the road to Petrovac, 570 m, 42.2188°N, 19.0246°E, leg. LD, ZF, JK, DM, 14.x.2008 (HNHM 99537); N of Tuđemili along the Bar to Virpazar road, 410 m, 42.1410°N, 19.1356°E, leg. MD, EH, KJ, HS, 7.vii.2015 (NHMW 110430/MN/0156); same locality, leg. ZE, ZF, 19.iv.2000 (HNHM 83481); near the Sutorman Pass, 760 m, 42.1568°N, 19.0996°E, leg. MD, EH, KJ, HS, 7.vii.2015 (NHMW 110430/MN/0157); Sutorman Pass, 42.1558°N, 19.1025°E, leg. TN, 1.vi.2014 (HNHM 99647); 9 km S of Virpazar, along the road to Bar, 440 m, 42.1912°N, 19.1081°E, leg. LD, ZF, JK, DM, 14.x.2008 (HNHM 99648); Virpazar, fortress, 42.244°N, 19.093°E, leg. ZE, ZF, 19.iv.2000 (HNHM 95414); Virpazar, 42.2460°N, 19.0917°E, leg. TD, ZE, ZF, 25.v.2015 (NHMW 110.430/MN/0122); 1 km E of Godinje, 40 m, 42.2205°N, 19.1241°E, leg. TD, ZE, ZF, 25.v.2015 (NHMW 110.430/MN/0123); 3.5 km SW of Virpazar, near Gluhi do, 42.2256°N, 19.0659°E, leg. TD, ZE, ZF, 25.v.2015 (NHMW 110.430/MN/0124); 0.5 km N of Virpazar, 42.2513°N, 19.0873°E, leg. TD, ZE, ZF, 25.v.2015 (NHMW 110.430/MN/0125); 1.3 km N of Virpazar, 42.2521°N, 19.0875°E, leg. TD, ZE, ZF, 25.v.2015 (NHMW 110.430/MN/0126); 3.5 km N of Virpazar, Vidikovac Observation Point, 150 m, 42.2547°N, 19.0896°E, leg. TD, ZE, ZF, 25.v.2015 (NHMW 110.430/MN/0127); Poseljani, 150 m, 42.3058°N, 19.0481°E, leg. TD, ZE, ZF, 25.v.2015 (NHMW 110.430/MN/0128); Začir, Pečina u Pećkom brdu, leg. LP, PS, AS, 20.vii.1972 (HNHM 36936); Komarno, near the Jabukov do Pečina, leg. ZE, ZF, 19.iv.2000 (HNHM 95418); Obodska Pećina, W of Rijeka Crnojevića, 150 m, 42.3523°N, 19.0047°E, leg. TD, ZE, ZF, 25.v.2015 (NHMW 110.430/MN/0129); 6.5 km NW of Rijeka Crnojevića, along the road to Cetinje, 310 m, 42.3651°N, 18.9924°E, leg. TD, ZE, ZF, 26.v.2015 (NHMW 110.430/MN/0130); “Rijeka Brücke”, leg. Bischoff, ex Klemm, viii.1934 (NHMW 21644); Ulići junction at the Podgorica to Cetinje road, leg. Kiss, LP, 9.vii.1985 (HNHM 43124); 1 km E of the Ulići junction, 470 m, 42.3815°N, 19.0084°E, leg. TD, ZE, ZF, 26.v.2015 (NHMW 110.430/MN/0131); Dobrska Župa junction, 250 m, 42.3865°N, 19.0571°E, leg. TD, ZE, ZF, 26.v.2015 (NHMW 110.430/MN/0132); 3 km from Barutan toward Rijeka Crnojevica, leg. LP, PS, AS, 20.vii.1972 (HNHM 36723); Cetinje, Donji Kraj District, 690 m, 42.4005°N, 18.9152°E, leg. TD, ZE, ZF, 26.v.2015 (NHMW 110.430/MN/0133); Mt. Lovćen, Njeguši, Popova Pečina, 42.4340°N, 18.8189°E, leg. LP, PS, AS, 23.vii.1972 (HNHM 36732, HNHM 37764); Njeguši, 910 m, 42.4267°N, 18.8314°E, leg. ZB, DP, 12.x.2003 (HNHM 94951); Njeguši, near the church, 880 m, 42.4316°N, 18.8126°E, leg. TD, ZE, ZF, 29.v.2015 (NHMW 110.430/MN/0149); Popova Pečina near Njeguši, leg. Sturany, 30.v.1903 (NHMW 38413); Nikšić District, W of Zagorak, 280 m, 42.6306°N, 19.0085°E, leg. ZB, DP, 7.x.2003 (HNHM 94911); Zeta Valley, ca. 2 km S of Nikšić along the road to Podgorica, 620 m, 42.7319°N, 18.9382°E, leg. TD, ZE, ZF, 30.v.2015 (NHMW 110.430/MN/0150); Podgorica District, Vitoja Spring, 42.3252°N, 19.3623°E, leg. AR, NR, PR, x.2013 (HNMW 110430/MN/0119); same locality, leg. MD, EH, KJ, HS, 8.vii.2015 (NHMW 110430/MN/0158); 2 km W of Božaj, 42.31°N, 19.37°E, leg. ZE, ZF, 30.vi.1996 (HNHM 91032); “Titograd” (= Podgorica), leg. Jakucs, 1966 (HNHM 95285); Podgorica, Mt. Ljubovic, eastern side, 42.4306°N, 19.2544°E, leg. MD, EH, KJ, HS, 8.vii.2015 (NHMW 110430/MN/0160); Šipčanik, 60 m, 42.3707°N, 19.3139°E, leg. TD, ZE, ZF, 25.v.2015 (NHMW 110.430/MN/0120); Žabljak Crnojevića, fortress, 50 m, 42.3172°N, 19.1567°E, leg. TD, ZE, ZF, 25.v.2015 (NHMW 110.430/MN/0121); Bar District, Dobra Voda, Tunel Ujtin, leg. Kiss, LP, 8.vii.1985 (HNHM 43121); 1 km S of Zaljevo, at Tunel Cafe, 90 m, 42.0572°N, 19.1278°E, leg. TD, ZE, ZF, 26.v.2015 (NHMW 110.430/MN/0138); 2 km S of Buljarica, 70 m, 42.1906°N, 18.9898°E, leg. TD, ZE, ZF, 28.v.2015 (NHMW 10.430/MN/0145); same locality, leg. ZE, ZF, 29.vi.1996 (HNHM 90880); Ulcinj District, Šasko Lake, 41.9768°N, 19.3387°E, leg. ZF, TK, DM, 16.vi.2012 (HNHM 99489); 1 km N of Gornja Kležna, 110 m, 42.0040°N, 19.2630°E, leg. TD, ZE, ZF, 27.v.2015 (NHMW 110.430/MN/0140); same locality, leg. PJ, TK, Magos, GP, 26.v.2013 (HNHM 99535); 5 km N of Zoganje, 80 m, 41.9794°N, 19.2591°E, leg. TD, ZE, ZF, 27.v.2015 (NHMW 110.430/MN/0139); 4 km S of Arbnež, 350 m, 42.0582°N, 19.3535°E, leg. TD, ZE, ZF, 28.v.2015 (NHMW 110.430/MN/0144); Albania, Selca (= Selcë), ex Fuchs (NHMW 111238); Maranaj north of Skutari, leg. Dabović (NHMW 27733); Shkodër District, Mt. Tarabosh, Vidhgar, 42.0572°N, 19.3962°E, leg. ZB, DP, 14.v.2013 (HNHM 99478); Vallas, 190 m, 42.0506°N, 19.4162°E, leg. TD, ZE, ZF, 27.v.2015 (NHMW 110.430/MN/0141); above Vallas, S of the Tarabosh Summit, 300 m, 42.0524°N, 19.4182°E, leg. TD, ZE, ZF, 27.v.2015 (NHMW 110.430/MN/0142); Shkodër, Rozafa Hill, S side, 42.0451°N, 19.4902°E, leg. TD, ZE, ZF, 28.v.2015 (NHMW 110.430/MN/0143); same locality, leg. ZB, ZF, CN, DP, 14.viii.2004 (HNHM 94509); same locality, leg. ZB, DP, 14.v.2013 (HNHM 99649); Zusi near Shkodër, 42.0386°N, 19.4811°E, leg. AR, NR, PR, v.2015 (NHMW 110.430/MN/0151); Lezhë District, Torovicë, 41.9000°N, 19.5130°E, leg. ZE, ZF, JK, DM, 21.x.2002 (HNHM 91612).

##### Distribution.

**Figure 35.**
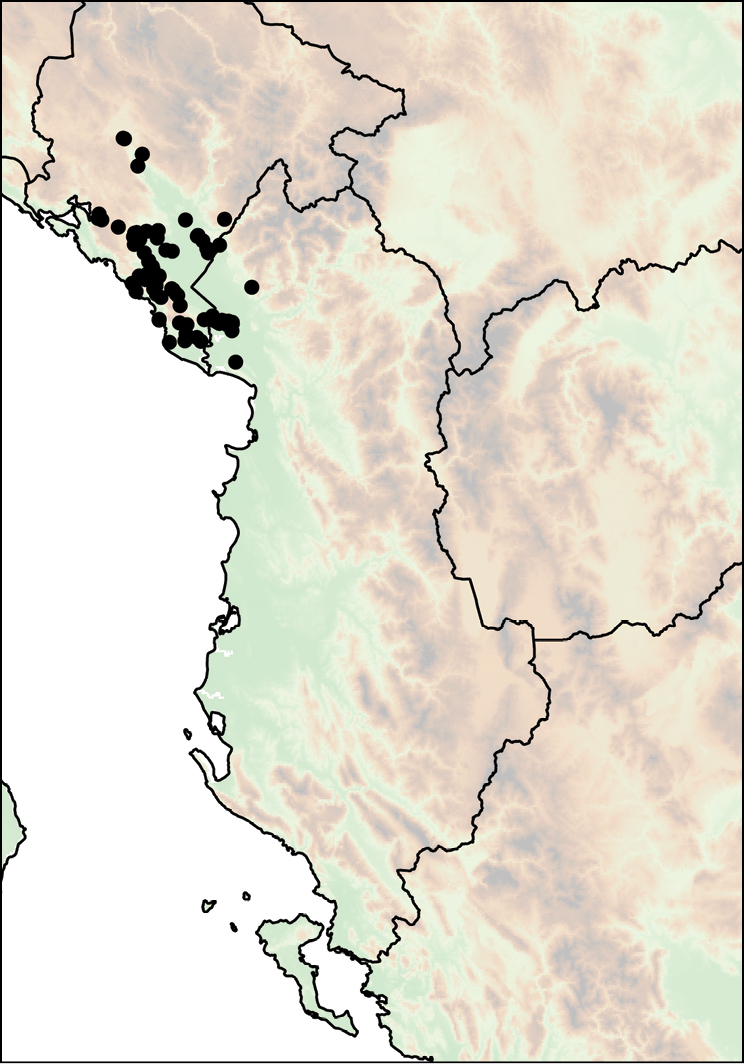
Distribution of *Montenegrina subcristata*.

Western Montenegro and northwestern Albania (Fig. 35). This species has a wide range and by far the highest number of known localities within the genus. It occurs primarily in the region of the Skutari Lake, and is particularly frequent along its northern and western shores, as well as in the area between the lake and the Adriatic coast. To the northwest it reaches Cetinje and the Njeguš Plateau, and in the north it has isolated occurrences to the Zeta valley around Niksič. Its southernmost occurrence was found near Torovicë (in Albania), whereas the easternmost record is probably from the Mt. Maranaj near Shkodër. Wohlberedt’s (1907) records from farther east, namely the Prokletije Mts (Medun, Zatrijebac, Selcë, between Kolašin and Andrijevica) could not be confirmed by recent field studies (Fehér et al. 2004, Murányi et al. 2011), therefore they cannot be accepted without reservation.

##### Remarks.

In the description the type locality of *subcristata* was only vaguely defined by Pfeiffer (1848). However, Küster’s (1844-1862) description provides important clues, mentioning that the material originated from the border area of Montenegro (with that of the Habsburg Monarchy), a site somewhere along the road from Kotor (to Cetinje) at an altitude of 3000 feet (ca. 900 m). Based on these data the type locality of the nominotypical subspecies can be in the vicinity of Njeguši, E of the Bay of Kotor.

Plate 4 of Küster’s monograph with figures of *subcristata* was published in 1847, whereas pages 39–40 with the description only in 1950. The plates contained no names, only accompanying sheets (wrappers) were issued along with the plates, so that subscribers and librarians would know which species were figured. These printed sheets were not meant to provide permanent scientific records. Due to the publication of Plate 4 and its wrapper, Pfeiffer could refer to *subcristata* in 1848, and attribute its authorship to Küster. However, ICZN Art. 8.1.1 specifies that for the availability of scientific names only the publication dates of text pages are relevant, and thus the wrappers are not acceptable as published work. Accordingly, Pfeiffer (1848) used the name first in proper form, therefore he should be considered the author (Welter-Schultes 1999: 165).

In 1899 Möllendorff gave description of a variety from Virpazar, but without a name. Later Wohlberedt (1901) mentioned the name *sublabiata* Möllendorff, without providing a description or indication according to ICZN Art. 12 (*nomen nudum*). Later Wohlberedt (1909) published the name *sublabiata* together with Möllendorff’s (1899) description, but he also published these, in literal Serbian translation, two years earlier (Wohlberedt 1907). Therefore, 1907 should be regarded as the year of publication and Wohlberedt as the author. This little-known Wohlberedt paper in Cyrillic script was probably overlooked or misunderstood by Zilch (1981), who referred to its *sublabiata* as *nomen nudum*.

In his 1907 and 1909 papers Wohlberedt clearly indicated that the descriptions of the new clausiliids are Boettger’s contributions, therefore the authorships of *interior* and *brunnea* are Boettger’s.

Westerlund (1881) described Clausilia (Herilla) klecaki, with this spelling. Later (Westerlund 1884) he used this name as *kleciaki*, and subsequently all authors adopted, probably incorrectly, this spelling form. Based on the original description, *kleciaki* is identical with the typical *subcristata*. By contrast, Sturany and Wagner used the name *kleciaki* in a different way (Sturany 1905, Sturany and Wagner 1915, Wagner 1924), for the form that was described by Boettger as *brunnea* (see also: Nordsieck 1972).

##### Intraspecific variability.

Nordsieck (2007, 2009) accepted only two valid subspecies, namely *s. subcristata* and *s. wohlberedti*. These differ primarily in the position of the lunella: *subcristata* having it ventrolaterally, whereas *wohlberedti* laterally. In this view, *minor*, *interior* and *kleciaki* are synonyms of the nominotypical form, whereas *sublabiata* and *brunnea* are synonyms of *wohlberedti*. The examined populations were highly variable in the size and shape of the shell, as well as in the position and shape of the lunella, allowing no clear delineation of two (or more) morphotypes. Moreover, considerable morphological heterogeneity could sometimes be observed within the same museum lots, or between lots from the same localities. Therefore, with the present knowledge, we find most appropriate classifying this diverse species without subspecific division.

#### Montenegrina timeae

Taxon classificationAnimaliaStylommatophoraClausiliidae

Erőss & Szekeres, 2006

Fig. 34F

Montenegrina irmengardis timeae Erőss & Szekeres, 2006 in Erőss et al. 2006: 194–196, fig. 15.
Montenegrina skipetarica timeae – Nordsieck 2009: 73.

##### Diagnosis.

Shell medium to large, ventricose, brownish-corneous. Whorls flat, smooth and glossy. Neck weakly inflexed, densely, irregularly striate-costate. Basal crest strong, peripheral crest barely recognizable. Peristome attached, ovoid, with somewhat swollen margin. Lamellae superior and spiralis overlap. In front view lamella inferior moderately emerged, medium-bent subcolumellaris not visible. Lunella dorsolateral to dorsolateral-lateral, fused to the basalis. Lunella complex with the basalis forms a lambda-like structure. Plica superior often fused to the principalis. Subclaustralis shorter than the basalis, sulcalis residual. Anterior plica superior absent to well developed, never connected to the lunella complex. Clausilium plate barely visible through the aperture. Distinguishable from *Montenegrina skipetarica* by the distinctly glossy shell surface, weaker-bent lamella subcolumellaris, shorter subclaustralis, and the less visible clausilium.

##### Dimensions

(in mm). H_s_: 15.0–22.0 (holotype 19.7), W_s_: 4.5–5.9 (holotype 5.4).

##### Type locality.

Albania, Mat District, 2 km S of Fshat, toward Gurri i Bardhë, SE of Burrel, along the Klos to Elbasan road, gorge of the Mat River, left side, 610 m, 41.4718°N, 20.0967°E.

##### Type material.

Type locality, leg. ZE, ZF, JK, DM, 26.x.2002, holotype (HNHM 94855), paratypes (NHMUK 20050222/2, HNC 63186/2, HNHM 94856/126, NHMW 103283/2, RMNH 100315/2, SMF 328087/2, NMBE 22597/3).

##### Other material.

Type locality, leg. ZF, TN, EM, 15.iv.2014 (HNHM 99009); 6.7 km NE of Gurri i Bardhë, along the Klos to Elbasan road, 650 m, 41.4685°N, 20.0904°E, leg. ZF, TN, EM, 15.iv.2014 (HNHM 99018); same locality, leg. ZF, JK, DM, 9.x.2004 (HNHM 94857); 5.5 km N of Gurri i Bardhë, 740 m, 41.4710°N, 20.0779°E, leg. ZF, TN, EM, 15.iv.2014 (HNHM 99020); same locality, le g. ZF, JK, DM, 9.x.2004 (HNHM 94858); Ura e Vashës, in the gorge of the Lumi i Matit, at its confluence with the Përroi i Gurri i Bardhit, 350 m, 41.4677°N, 20.1048°E, leg. ZF, TN, EM, 15.iv.2014 (HNHM 99011).

##### Distribution.

**Figure 36.**
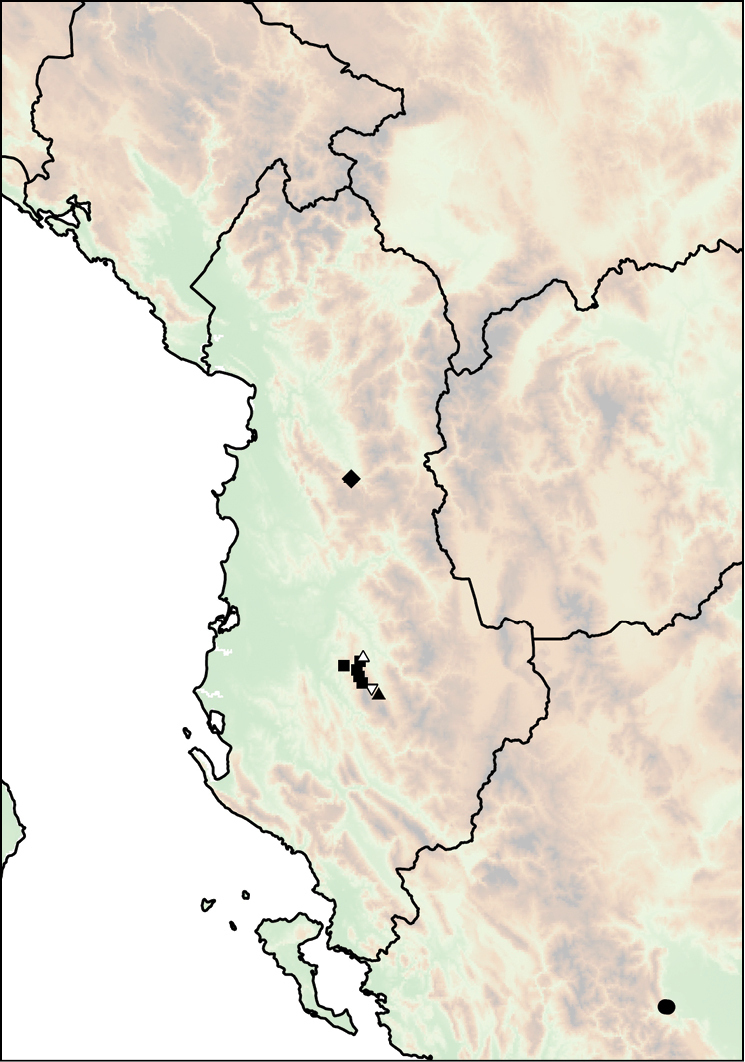
Distribution of *Montenegrina timeae*; *Montenegrina tomorosi* and *Montenegrina zilchi*. *Montenegrina timeae* (diamond); *Montenegrina tomorosi ampla* (triangle); *Montenegrina tomorosi coerulescens* (square) *Montenegrina tomorosi hunyadii* ssp. n. (empty triangle); *Montenegrina tomorosi tomorosi* (empty inverted triangle); *Montenegrina zilchi* (circle).

Northern part of the Gropa Mts in Central Albania. Known from a few localities around Gurri i Bardhë (near Klos) (Fig. 36).

##### Remarks.

Originally *Montenegrina timeae* was described as a subspecies of *Montenegrina skipetarica*, which has a similar, lambda-like lunella complex.

This species, as usual in this genus, was found on cliffs at all but one site. At Ura e Vashës, where *Montenegrina helvola magna* inhabits the rock surface at the entrance of the Mat gorge, a few specimens of *Montenegrina timeae* were found under stones in the riverbed gravel. This seemed to be a temporary colonisation by specimens drifted away by flood water, thus it should not be a basis for far-reaching conclusions regarding the habitat preferences of the species.

#### Montenegrina tomorosi

Taxon classificationAnimaliaStylommatophoraClausiliidae

Brandt, 1961

##### Diagnosis.

Shell very small to medium, light to brownish-corneous. All whorls smooth or very finely, indistinctly wrinkled. Neck variably inflexed, wrinkled-costate. Peristome attached, rounded to somewhat angular, with simple to strongly swollen margin. Lamellae superior and spiralis do not overlap. In front view lamella inferior barely to moderately emerged, weakly-bent subcolumellaris mostly hidden. Lunella broad, dorsolateral to lateral. Plica superior fused to the principalis. Basalis absent to weak and separate from the lunella. Subclaustralis absent to weak, sulcalis residual to well developed. Anterior plica superior absent to long, free or connected to the lunella complex. Clausilium plate not to partly visible through the aperture.

#### Montenegrina tomorosi tomorosi

Taxon classificationAnimaliaStylommatophoraClausiliidae

Brandt, 1961

Fig. 34G

Montenegrina (Montenegrina) janinensis tomorosi Brandt, 1961: 3–4, plate 1, fig. 2.
Montenegrina janinensis tomorosi – Zilch 1981: 129, plate 14, fig. 29. – Nordsieck 2009: 75.

##### Diagnosis.

Shell small to medium, light corneous. All whorls smooth. Neck weakly inflexed, densely wrinkled-costate. Basal and peripheral crests weak. Peristome ovoid to somewhat angular, with swollen margin. Lamellae superior and spiralis do not overlap. In front view lamella inferior moderately emerged, subcolumellaris not visible. Lunella dorsolateral. Basalis weak, mostly separate from the lunella. Subclaustralis absent to residual, sulcalis present. Anterior plica superior absent or weak, not connected to the lunella complex. Clausilium plate barely visible through the aperture. Distinguishable from *Montenegrina janinensis* by the non-overlapping lamellae superior and spiralis, as well as the weaker-bent and deeper ending lamella subcolumellaris.

##### Dimensions

(in mm). H_s_: 16.1–17.8 mm, W: 3.6–4.1 mm (penultimate whorl) up to 4.6 mm (at the basis) (Brandt 1961).

##### Type locality.

“Kutniak im Tomoros-Gebirge in Südalbanien, in 1400 m … vor einem Höhleneingang” = Albania, Tomorr Mts, Mt. Kulmak, in front of a cave entrance, 1400 m.

##### Type material.

Type locality, leg. Winkler, ? v.1931, holotype (SMF 163940), paratypes (SMF 163941/3, SMF 201612/8, SMF 265703/2, SMF 265704/5, ZMH 26303/5, ZMH 91631/8, ZSM 20013687, NHMW 111236/10, NHMW-E 30320/pl., NHMW-K 9369/pl., NHMW-K 45964/5).

##### Other material.

Albania, Tomorr Mts, vicinity of the “Teqe”, leg. Winkler (NHMW-E 43234/10, NHMW-K 9370/pl.); “Kutniak, Tomora-Gebirge”, ex Zilch (HNHM 36720); Skrapar District, Tomorr Mts, 400 m N of the Maja e Kulmakut, 2150 m, 40.5896°N, 20.2414°E, leg. ZB, ZF, CN, DP, 9.viii.2004 (HNHM 94922); 2.5 km NW of the Maja e Kulmakut, 1610 m, 40.5952°N, 20.2214°E, leg. ZB, ZF, CN, DP, 9.viii.2004 (HNHM 94921); small gorge 1 km NW of the Maja e Kulmakut, 2070 m, 40.5913°N, 20.2385°E, leg. ZB, ZF, CN, DP, 9.viii.2004 (HNHM 94923); Ujanik (4 km W of Gjerbës), gorge of the Përroi i Ujanikut, 970 m, 40.6328°N, 20.2161°E, leg. ZF, AH, TH, DM, 23.viii.2006 (HNHM 99541).

##### Distribution.

Tomorr Mts in southern Albania. This taxon occurs in the southern chain of the Tomorr Mts, to the north and west of the Kulmak Summit (Fig. 36).

##### Remarks.

In older collection labels the Tomorr Mts is often given as “Tomoros”. This is not to be confused with the main summit of the Galičica Mts, west of the Ohrid Lake, which is sometimes also mentioned as Tomoros (see *Montenegrina dofleini dofleini*).

#### Montenegrina tomorosi ampla

Taxon classificationAnimaliaStylommatophoraClausiliidae

Fehér & Szekeres, 2006

Fig. 34I

Montenegrina janinensis ampla Fehér & Szekeres, 2006 in Erőss et al. 2006: 196, fig. 16. – Nordsieck 2009: 75.

##### Diagnosis.

Shell medium, light corneous. All whorls smooth. Neck weakly inflexed, densely and finely striate-costate. Basal and peripheral crests weak. Peristome rounded to somewhat angular, with strongly swollen, reflexed margin. Lamellae superior and spiralis do not overlap. In front view lamella inferior moderately emerged, subcolumellaris mostly not visible. Lunella dorsolateral to lateral. Basalis mostly separate from the lunella. Subclaustralis short, sulcalis very close and often connected to the lamella subcolumellaris. Anterior plica superior close and parallel to the principalis, fused to the lunella complex. Clausilium plate not visible through the aperture.

##### Dimensions

(in mm). H_s_: 15.0–20.2 (holotype 19.2), W_s_: 3.8–4.9 (holotype 4.7).

##### Type locality.

Albania, Skrapar District, Tomorr Mts, karst sinkhole 1 km S of the Maja e Ramiës, 1850 m, 40.5686°N, 20.2585°E.

##### Type material.

Type locality, leg. ZB, ZF, CN, DP, 9.viii.2004, holotype (HNHM 94865), paratypes (NHMUK 20050223, HNC 63187, HNHM 94866/97, NHMW 103284, RMNH 100318, SMF 328088).

##### Other material.

Albania, Tomorr Mts, sinkhole east of the Maja e Ramiës, 2050 m, 40.5775°N, 20.2582°E, leg. ZB, ZF, CN, DP, 9.viii.2004 (HNHM 94867).

##### Distribution.

Tomorr Mts in southern Albania. This species was found in the southern chain of the Tomorr, within a restricted range around the Ramië Summit (Fig. 36).

#### Montenegrina tomorosi coerulescens

Taxon classificationAnimaliaStylommatophoraClausiliidae

Nordsieck, 1996

Fig. 34H

Montenegrina janinensis coerulescens Nordsieck, 1996: 10, plate 3, fig. 4. – Nordsieck 2009: 75.

##### Diagnosis.

Shell small, violet-brown, often with a whitish tint. All whorls smooth. Neck weakly inflexed, densely costate. Basal and peripheral crests weak. Peristome ovoid to somewhat angular, with swollen margin. Lamella superior weak, distant from the spiralis. In front view lamella inferior barely emerged, subcolumellaris mostly not visible. Lunella dorsolateral to dorsolateral-lateral. Basalis, subclaustralis and anterior plica superior absent, sulcalis present. Clausilium plate not or only barely visible through the aperture.

##### Dimensions

(in mm). H_s_: 13.9–16.8 (holotype 16.1), W_s_: 3.7–4.4 (holotype 4.2).

##### Type locality.

Albania, Tomorr Mts, eastern slope of the Çuka Partizan, 1850 m.

##### Type material.

Type locality, leg. HS, 16.viii.1992, holotype (NHMW 89274), paratypes (NHMW 87346/pl., SMNS-N 10035).

##### Other material.

Albania, “Weg zwischen Kloster Teqe und Tomor, 2200 m”, leg. Winkler (NHMW-E 16489/1); Periferi Berat, Tomorr Mts, Maja e Tomorrit, summit region, near the Bektashi shrine (teqe), 2270 m, 40.6306°N, 20.1662°E, leg. ZB, ZF, CN, DP, 10.viii.2004 (HNHM 94916); 700 m N of the Maja e Tomorrit along the ridge, 2370 m, 40.6411°N, 20.1575°E, leg. ZB, ZF, CN, DP, 11.viii.2004 (HNHM 94915); same locality, leg. ZF, AH, TH, DM, 23.viii.2006 (HNHM 99626); 1 km N of the Maja e Tomorrit along the ridge, 2230 m, 40.6440°N, 20.1590°E, leg. ZB, ZF, CN, DP, 10.viii.2004 (HNHM 94920); 6 km N of the Maja e Tomorrit, 2090 m, W of the ridge, 40.6889°N, 20.1461°E, leg. ZB, ZF, CN, DP, 11.viii.2004 (HNHM 94909); ca. 7 km NW of Terovë, NE of the Çuka Partizan, 1865–1950 m, 40.7164°N, 20.1567°E, leg. ZF, AH, TH, DM, 25.viii.2006 (HNHM 99627); 5 km from Tomorr Village towards the ridge, 1780 m, 40.6880°N, 20.1399°E, leg. ZB, ZF, CN, DP, 11.viii.2004 (HNHM 94908); 4.5 km from Tomorr village towards the ridge, 1520 m, 40.6883°N, 20.1341°E, leg. ZB, ZF, CN, DP, 11.viii.2004 (HNHM 94907); 1.5 km S of Tomorr Village, towards the ridge, 980 m, 40.6788°N, 20.1108°E, leg. ZB, ZF, CN, DP, 11.viii.2004 (HNHM 94913).

##### Distribution.

Tomorr Mts in southern Albania. This taxon occurs on the northern chain of this mountain, around and between the Tomorr and Partizan Summits (Fig. 36).

#### Montenegrina tomorosi hunyadii ssp. n.

Taxon classificationAnimaliaStylommatophoraClausiliidae

http://zoobank.org/3E153078-DEFA-40B7-8601-3B5A2353BE60

Fig. 34J

##### Diagnosis.

Small subspecies with smooth whorls, inflexed neck, and well developed basal and peripheral crests.

##### Description.

The small, light corneous shell consists of 8½ to 9½ whorls. The surface is smooth, only at the neck is wrinkled-costate. The neck is inflexed, the basal and peripheral crests are well developed. The ovoid to somewhat angular peristome is attached, its margin is slightly swollen. The lamellae superior and spiralis usually do not overlap. In front view the lamella inferior is moderately emerged, the weakly-bent subcolumellaris is mostly not visible. The broad, dorsolateral lunella is fused, via the plica superior, to the plica principalis. The basalis is weak, not connected to the lunella. The subclaustralis is absent, the sulcalis is well developed. The anterior plica superior of variable strength is separate from the lunella complex. The clausilium plate is partly visible through the aperture.

##### Dimensions

(in mm). Holotype H_s_: 12.1, W_s_: 3.4, H_a_: 3.2, W_a_: 2.4; paratypes (HNHM 99511, n = 12): H_s_: 10.8–13.1 (mean 12.2, S.D. 0.69), W_s_: 3.0–3.6 (mean 3.3, S.D. 0.18), H_a_: 2.8–3.5, W_a_: 2.2–2.6.

##### Differential diagnosis.

Differs from all other *Montenegrina tomorosi* subspecies by its smaller, translucent shell, as well as the stronger neck inflection and crests.

##### Type locality.

Albania, Skrapar District, Tomorr Mts, ca. 1.5 km W of Terovë, dry gorge, 850 m, 40.7127°N, 20.1839°E.

##### Type material.

Type locality, leg. ZF, AH, TH, DM, 24.viii.2006, holotype (HNHM 99510), paratypes (HNHM 99511/97, NHMW 111232/5, HU/97, SZ/8).

##### Etymology.

The new taxon is named after András Hunyadi, malacologist, who accompanied the first author on several Balkan field trips, including the one that led to the discovery of this subspecies.

##### Distribution.

Tomorr Mts in southern Albania. Known only from the type locality, in the eastern slope of the Tomorr (Fig. 36).

#### Montenegrina zilchi

Taxon classificationAnimaliaStylommatophoraClausiliidae

Nordsieck, 1974

Fig. 34N

Montenegrina zilchi Nordsieck, 1974: 156, plate 6, fig. 39. – Zilch 1981: 132, plate 15, fig. 43. – Nordsieck 2009: 73.

##### Diagnosis.

Shell large, brownish-corneous. All whorls smooth. Neck not or very weakly inflexed, densely striate. Basal crest weak, peripheral crest not recognizable. Peristome attached, ovoid, with somewhat swollen margin. Lamellae superior and spiralis overlap. In front view lamella inferior well emerged, broadly-bent subcolumellaris visible. Lunella dorsal, fused to the long basalis. Subclaustralis short, sulcalis strong. Anterior plica superior separate from the lunella complex, accompanied by one or two short lower plicae. Forward-bending parietal edge of the clausilium plate occasionally visible in front view.

##### Dimensions

(in mm). H_s_: 19.4–26.1 (holotype 24.9), W_s_: 5.1–6.6 (holotype 5.8).

##### Type locality.

Greece, Thessaly, Pili near Trikala, medieval Turkish bridge.

##### Type material.

Type locality, leg. Nordsieck, 7–8.ix.1972, holotype (SMF 227690), paratypes (SMF 227691, SMNS-N 5940); same locality, leg. Fauer, 31.vii.1973, paratypes (SMNS-N 6711, ZMH 113582/pl.).

##### Other material.

Type locality, 39.4603°N, 21.6008°E, leg. ZE, ZF, JG, 22.vi.2013 (HNHM 99598).

##### Distribution.

Gorge of the Portaikos River at the western edge of the Thessaly Basin. Known only from the type locality (Fig. 36).

## Discussion

The widely accepted species and subspecies concepts of Ernst Mayr provide a good theoretical background for taxonomists (Haffer 1997, Mallet 2007, Winker 2010). In theory, the lack of hybridization between different morphs under natural conditions indicates that they represent distinct species. When a morphologically variable species comprises parapatric populations with geographically partitioned morphs, and there are abrupt transitions between well discernible morphotypes with only narrow hybrid zones, then the species is polytypic. Their subspecific morphs with the mentioned characteristics represent biologically meaningful units that are reasonable to distinguish as subspecies (Welter-Schultes 2010).

However, the practical application of species and subspecies categories in the classification of allopatrically distributed groups is hampered by considerable difficulties (Gittenberger 2007). In *Montenegrina*, there are only a few known cases when different morphs co-occur or meet in a contact zone (Table 1). In a rare exception, the syntopic occurrence of *Montenegrina perstriata ochridensis* and *Montenegrina nana barinai* ssp. n. showed that *Montenegrina perstriata*, in the sense of Nordsieck (2009), consists of more than one species.

**Table 1.** Co-occurrences or contacts of different *Montenegrina* morphs.

Co-occurring taxa	Type of coexistence	Locality	Habitat	Microhabitat	Notes
*Montenegrina minuscula* and *Montenegrina skipetarica skipetarica*	syntopic coocurrence	3 km W of the Qafa e Murrës, near the Shkëmb i Skanderbeut, 970 m, 41.6465°N, 20.1898°E	gorge of a river (Lumi i Varoshit)	On exposed rock surface	
*Montenegrina skipetarica skipetarica* and *Montenegrina soosi*	syntopic coocurrence	2.5 km N of Bushtricë, Ura e Lapavës, 600 m, 41.8949°N, 20.4169°E	gorge of a creek (Pr i. Vaut të Cajës)	On exposed rock surface	
*Montenegrina grammica grammica* and *Montenegrina skipetarica pindica*	syntopic cooccurrence	Gramos Mts, W of Epano Arena summit, 2000 m, 40.3090°N, 20.8981°E	steep cliffs on rocky grassland	On exposed rock surface and among stones at the foot of the cliffs	The former one seems to be common and widespread in the whole summit region whereas the latter one was found to be within a narrow range
*Montenegrina grammica erosszoltani* and *Montenegrina skipetarica puskasi*	syntopic cooccurrence	Mali i Dejës, above the headwaters of the Lumi i Varoshit, 1540 m, 41.6746°N, 20.2112°E	steep cliffs	On exposed rock surface	
*Montenegrina dofleini pinteri* and *Montenegrina stankovici*	cooccurrence but apparent spatial segregation on the fine scale	N of Trpejca, 700 m, 40.9656°N, 20.7858°E	lakeshore cliffs	On exposed rock surface	Fine scale spatial segregation is supposed to be the consequence of different micro-habitat preference. *Montenegrina stankovici* occurs very close to the rock surface, *Montenegrina dofleini pinteri* is found some meters higher and farther from the water
*Montenegrina helvola magna* and *Montenegrina timeae*	cooccurrence but apparent spatial segregation on the fine scale	Ura e Vashës, 41.4677°N, 20.1048°E	gorge of a river (Lumi i Matit)	On exposed rock surface/riverbed under stones	*Montenegrina helvola magna* lives on the rock surface at the gorge entrance, *Montenegrina timeae* was found under stones in the riverbed gravel deposit. Note that in all other sites, *Montenegrina timeae* was to live on rock surface as usual for this genus. This one seems to be a temporal occurrence of some drifted specimens which will be extirpated by the following flash flood
*Montenegrina dofleini wagneri* and *Montenegrina hiltrudae fusca*	syntopic cooccurrence	Thatë Mts, near the ‘Tower of the war’, 40.9136°N, 20.8419°E	steep cliffs on rocky grassland	On exposed rock surface and among/under stones in the rocky grassland	collected/observed by I. Dedov
*Montenegrina nana barinai* and *Montenegrina perstriata ochridensis*	syntopic cooccurrence	Maja e Temlishit, 41.514°N, 20.316°E	steep cliffs	On exposed rock surface	
*Montenegrina hiltrudae selcensis* and *Montenegrina laxa errans*	syntopic cooccurrence	Shkëmb i Selcës, 40.7480°N, 20.5219°E	gorge of a river (Lumi i Verbës)	On exposed rock surface	
*Montenegrina perstriata ochridensis* and *Montenegrina stankovici*	cooccurrence but apparent spatial segregation on the fine scale	ca. 2.5 km S of Peštani, 41.0°N, 20.8°E	lakeshore cliffs	On exposed rock surface	Fine scale spatial segregation is supposed to be the consequence of different micro-habitat preference. *Montenegrina stankovici* occurs very close to the rock surface, *Montenegrina perstriata ochridensis* was found farther from the water (P. Subai personal communication)
*Montenegrina cattaroensis antivaricostata* and *Montenegrina cattaroensis umbilicata*	parapatry with hybrid zone	3 km above Stari Bar, on the footpath to Veliki Mikulići, 42.097°N, 19.146°E	rock wall and boulders by the footpath	On exposed rock surface	along a transect of the footpath between Stari Bar and Veliki Mikulići, morphologically transitional specimens were found only within a narrow zone (<50 m)
*Montenegrina skipetarica gurelurensis* and *Montenegrina skipetarica puskasi*	parapatry with hybrid zone	1 km W of Cidhnë, along the footpath to Gurë-Lurë, 41.752°N, 20.261°E	rock wall by the footpath	On exposed rock surface	along a transect of the footpath between Cidhnë and Gurrë-Lurë, morphologically transitional specimens were found only within a narrow zone (<100 m)

In the absence of natural contact there is no way of directly verifying reproductive isolation and competitive interactions. In such cases there are only indirect ways of inferring taxonomic differences. Namely, by comparing the degree of differences between related sympatric species, between intergrading subspecies of widespread species, or between hybridizing populations of related species (Mayr and Ashlock 1991: 104-105, Coyne and Orr 2004: 45, Haffer 1997). This makes the expert decision, whether allopatric morphs represent distinct subspecies within a polytypic species or only morphologically divergent populations of a monotypic taxon, inevitably subjective.

A further complication is that the actual classification of a group can also depend on the extent of its exploration. In poorly explored groups the complexity and variability range of the populations/morphs is only superficially known. Newly discovered populations often represent unknown morphotypes that are usually described under new names. New taxa are established as long as the new morphotypes seem clearly distinguishable and geographically well separated. However, more detailed studies can reveal continuous spatial gradients of several traits or entangling of the distribution areas, and thus can necessitate the merging of former taxa. At a certain exploration level a new type of classification may become necessary, one that involves substantially fewer taxa than the earlier approach. Such was the case, for instance, with Cretan *Albinaria*, where a novel classification concept resulted in massive synonymization of the formerly accepted subspecies (Welter-Schultes 2010).

Though Welter-Schultes (2012: 217) suggests the extension of the same ‘lumper’ approach to other rock-dwelling clausiliid genera, in particular *Alopia*, *Bulgarica* Boettger, 1877 and *Montenegrina*, the current taxonomy of rock-dwelling Balkan alopiinids still follows he traditional ‘splitter’ way (Nordsieck 2007, Bank 2013). Apparently, there is no single model that could be fully suitable for the classification of all these genera. For instance, in contrast to the Cretan *Albinaria*, in the case of *Charpentieria itala* (Martens, 1824) genetic relationships strongly supported the suitability of the polytypic species concept (Scheel and Hausdorf 2012).

Whereas taxonomic decisions unavoidably contain arbitrary elements, the nature of intra-generic heterogeneity provides guidance as to which of the approaches is to be used. The lumper approach is well applicable for species that have numerous populations within a geographically uninterrupted range, and shows high inter-, as well as intra-population diversity. By contrast, the splitter approach (or “traditional way”, as termed by Welter-Schultes 2010) is best suited for species that have spatially well separated, and by stable character state combinations unambiguously distinguishable populations, with high inter- but low intra-population variability.

Despite a substantial increase of occurrence data from Albania and the neighbouring regions, most of the known *Montenegrina* taxa still seem to show distinct, spatially structured distribution. That is, they occur sporadically, within narrow, geographically clustered ranges, which are well isolated from each other. These conditions justify their classification according to the polytypic species concept. However, *Montenegrina subcristata*, a species having a large number of populations within a geographically uninterrupted range, and showing high inter-, as well as intra-population diversity, seems to be best classified without subspecific division.

Therefore, at least at the current level of knowledge, we find the traditional method to be the most suitable way of classification, in which all taxa are recognized that are reliably distinguishable from the others. Accordingly, the present classification of *Montenegrina* contains 29 species and 106 subspecies, making it the second most speciose alopiinid genus after *Albinaria*.
